# FEDRANN: effective long-read overlap detection based on dimensionality reduction and approximate nearest neighbors

**DOI:** 10.1093/gigascience/giag048

**Published:** 2026-05-08

**Authors:** Jia-Yuan Zhang, Changjiu Miao, Teng Qiu, Junyi He, Wenqi Cao, Wei Lin, Xiaoshuang Xia, Lei He, Chunlei Yang, Yuhui Sun, Tao Zeng, Yuxiang Li, Xun Xu, Yijun Ruan, Yuliang Dong

**Affiliations:** Life Sciences Institute, Zhejiang University, 866 Yuhangtang Road, Xihu District, Hangzhou 310058, China; State Key Laboratory of Genome and Multi-omics Technologies, BGI Research, 202 Zhenzhong Road, Xihu District, Hangzhou 310030, China; BGI Research, 202 Zhenzhong Road, Xihu District, Hangzhou 310030, China; BGI Hangzhou CycloneSEQ Technology Co. Ltd., 203 Zhenzhong Road, Xihu District, Hangzhou 310030, China; BGI Hangzhou CycloneSEQ Technology Co. Ltd., 203 Zhenzhong Road, Xihu District, Hangzhou 310030, China; BGI Research, 202 Zhenzhong Road, Xihu District, Hangzhou 310030, China; BGI Hangzhou CycloneSEQ Technology Co. Ltd., 203 Zhenzhong Road, Xihu District, Hangzhou 310030, China; Life Sciences Institute, Zhejiang University, 866 Yuhangtang Road, Xihu District, Hangzhou 310058, China; State Key Laboratory of Genome and Multi-omics Technologies, BGI Research, 202 Zhenzhong Road, Xihu District, Hangzhou 310030, China; BGI Research, 202 Zhenzhong Road, Xihu District, Hangzhou 310030, China; BGI Hangzhou CycloneSEQ Technology Co. Ltd., 203 Zhenzhong Road, Xihu District, Hangzhou 310030, China; Life Sciences Institute, Zhejiang University, 866 Yuhangtang Road, Xihu District, Hangzhou 310058, China; State Key Laboratory of Genome and Multi-omics Technologies, BGI Research, 202 Zhenzhong Road, Xihu District, Hangzhou 310030, China; BGI Research, 202 Zhenzhong Road, Xihu District, Hangzhou 310030, China; College of Life Sciences, Zhejiang University, 866 Yuhangtang Road, Xihu District, Hangzhou 310058, China; BGI Research, 59 Keji 3rd Road, Donghu New Technology Development Zone, Wuhan 430074, China; BGI Research, 59 Keji 3rd Road, Donghu New Technology Development Zone, Wuhan 430074, China; BGI Hangzhou CycloneSEQ Technology Co. Ltd., 203 Zhenzhong Road, Xihu District, Hangzhou 310030, China; State Key Laboratory of Genome and Multi-omics Technologies, BGI Research, 9 Yunhua Road, Yantian District, Shenzhen 518083, China; State Key Laboratory of Genome and Multi-omics Technologies, BGI Research, 9 Yunhua Road, Yantian District, Shenzhen 518083, China; BGI Research, 59 Keji 3rd Road, Donghu New Technology Development Zone, Wuhan 430074, China; State Key Laboratory of Genome and Multi-omics Technologies, BGI Research, 9 Yunhua Road, Yantian District, Shenzhen 518083, China; Guangdong Provincial Key Laboratory of Genome Read and Write, BGI Research, 9 Yunhua Road, Yantian District, Shenzhen 518083, China; Life Sciences Institute, Zhejiang University, 866 Yuhangtang Road, Xihu District, Hangzhou 310058, China; BGI Research, 202 Zhenzhong Road, Xihu District, Hangzhou 310030, China; BGI Research, 9 Yunhua Road, Yantian District, Shenzhen 518083, China

**Keywords:** *de novo* assembly, overlap-layout-consensus, locality-sensitive hashing, dimensionality reduction, k-nearest neighbors

## Abstract

Overlap detection is a key step in *de novo* genome assembly pipelines based on the overlap-layout-consensus paradigm. Existing methods for overlap detection either rely on heuristic seed-and-extension strategies or locality-sensitive hashing (LSH), both of which struggle to handle repetitive genomic regions and the computational burden of large-scale datasets. Here, we present FEDRANN, a novel strategy for overlap graph construction that integrates feature extraction, dimensionality reduction (DR), and approximate nearest neighbor (ANN) search. We find the pipeline combining inverse document frequency (IDF) transformation, sparse random projection (SRP), and NNDescent enables accurate detection of overlaps across diverse datasets. We developed an efficient open-source implementation of this pipeline named Fedrann (https://github.com/jzhang-dev/fedrann). Through systematic benchmarking on real long-read sequencing data, we demonstrate that Fedrann produces overlap graphs comparable to or better than those generated by existing state-of-the-art tools, including MECAT2, minimap2, and wtdbg2, while maintaining competitive runtime. By integrating Fedrann into the Shasta assembler, we successfully reconstructed human whole genomes, achieving high assembly contiguity and quality. Despite being implemented primarily in Python, Fedrann achieves performance parity with tools written in compiled languages by leveraging C-accelerated numerical libraries and optimized batch-based matrix operations. Our results suggest that the combination of DR and ANN techniques offers a robust, scalable framework for accurate overlap detection in long-read assembly and broader sequence similarity search tasks.

## Introduction

*De novo* assembly reconstructs a genome from scratch using overlapping sequencing reads without a reference, which is crucial for studying novel genomes, uncovering structural variations, and exploring genetic diversity [1]. The advancement of long-read sequencing technologies, such as PacBio, Oxford Nanopore Technologies and BGI CycloneSEQ [2] platforms, has significantly improved *de novo* assembly by generating reads spanning repetitive regions and complex structural variants [3, 4]. Most long-read *de novo* assembly methods rely on the overlap-layout-consensus (OLC) approach, which aligns reads based on overlaps and iteratively refines contigs to reconstruct high-quality genomes [5–7]. A key step in the OLC approach is overlap detection, which involves finding overlaps from a large collection of sequencing reads, where the existence of an overlap between a pair of reads is typically determined by their sequence similarity. Overlap detection faces two primary challenges: the computational burden of processing large amount of sequences (millions of reads for human-sized genomes), which demands efficient algorithms and substantial memory resources, and the inherent complexity caused by repetitive genomic sequences that create ambiguous overlaps [8]. These repeats often lead to fragmented or erroneous connections in the overlap graph, significantly complicating the assembly process and potentially introducing misassemblies. This issue is further exacerbated by sequencing errors, which introduce spurious overlaps and masking true overlaps. Overcoming these obstacles is crucial for accurate genome reconstruction.

Existing methods for overlap detection can be roughly categorized into two classes: seed-and-extension and locality-sensitive hashing (LSH) methods. The seed-and-extension strategy first identifies small exact or near-exact matches (seeds) between the query sequence and the target sequences. Seed matches are then filtered based on local density or other criteria. For each retained seed, the algorithm attempts to extend the alignment in both directions using dynamic programming (DP) to build a more comprehensive alignment. Finally, target sequences with the best alignments (according to certain heuristic criteria) are identified as overlapping sequences. Overlap detection based on seed-and-extension is widely adopted by both general-purpose sequence alignment tools such as BLAST [9], DALIGN [10], and minimap2 [11], as well as *de novo* assembly-oriented tools such as MECAT [12], wtdbg2 [13], PECAT [7], and xRead [14].

As seed-and-extension methods only perform time-consuming DP-based alignment on high-potential regions that contain many seed matches, they scale well to large databases and long query sequences. However, the seed matching and filtering steps are heuristic in their nature, and are prone to returning target sequences that are locally but not globally similar to the query sequence (false positives) [15]. In addition, these methods often depend on many parameters such as minimum seed density, which require careful tuning to achieve a good trade-off between accuracy and computational efficiency.

LSH-based methods, in particular MinHash, have also been widely used in overlap detection. MinHash was originally developed for text mining tasks, e.g. finding similar news articles [16]. In MinHash, each sequence is first encoded as an unordered set of unique tokens, which are typically *k*-mers (subsequences of fixed-length *k*). The MinHash algorithm then applies multiple independent hash functions and records the minimum hash value of each set under each hash function. The resulting “sketch” serves as a compact approximation of the full set. Overlapping sequences will have more similar sketches compared to unrelated sequences. Popular *de novo assembly* tools that implement variants of MinHash include MHAP [17], Canu [5], and Shasta [6]. In addition, the overlap detection tool BLEND [18] uses another LSH-based method named SimHash to generate sketches for input sequences.

The LSH strategy replaces the time-consuming pairwise alignment step with rapid sketch comparison and therefore can potentially scale to large genomes while maintaining computational efficiency. For example, Shasta [6] is able to perform *de novo* assembly of the human genome within 6 h, of which only 3% is spent on the MinHash step. However, a key limitation of this strategy is that highly repetitive tokens, such as *k*-mers from low-complexity genomic regions, can dominate the sketch, overshadowing more informative, unique tokens, affecting assembly quality in these regions [5]. Another challenge of this strategy is the difficulty in identifying overlaps between sequences that vary greatly in their lengths using their respective sketches [6, 19, 20].

To address the limitations of existing approaches for overlap detection, we developed a new strategy inspired by common single-cell sequencing workflows. Our approach, hereby referred to as FEDRANN, comprises three main steps: feature extraction (FE), dimensionality reduction (DR), and approximate nearest neighbor (ANN) search. We first designed a benchmarking pipeline to evaluate overlap graph quality using both simulated and real long-read sequencing data. Using this pipeline, we systematically compared various combinations of feature extraction, DR, and ANN methods across datasets of differing sizes and complexities. We then implemented the best-performing method, which involved inverse document frequency (IDF) transformation, sparse random projection (SRP), and ANN searching using NNdescent, as a integrated overlap detection tool named Fedrann. We benchmarked Fedrann against established tools, including minimap2, MHAP, MECAT2, wtdbg2, xRead, and BLEND. Our results demonstrate that Fedrann constructs accurate overlap graphs across genomes of varying sizes and complexities while maintaining competitive execution times. By integrating Fedrann into the Shasta assembly framework, we successfully reconstructed human whole genomes with high contiguity and accuracy. These results were comparable to, and in specific cases surpassed, the performance of the original Shasta assembler, highlighting the practical utility and potential of Fedrann for large-scale genomic assembly applications.

## Results

### Overview of the FEDRANN workflow

**Figure 1:**
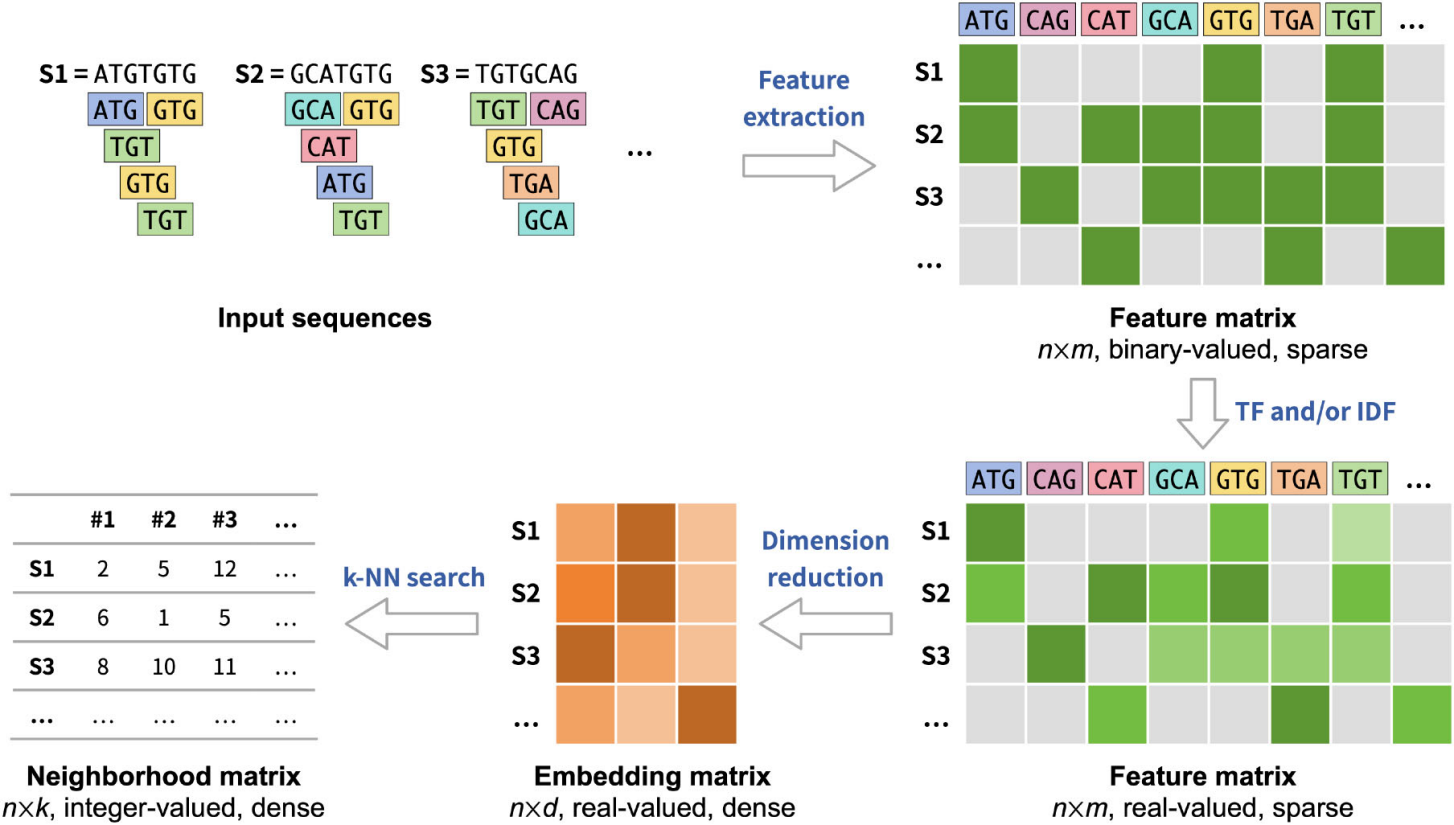
Diagrammatic representation of the proposed algorithm for sequence similarity search. The algorithm is comprised of three key steps: (1) encoding the input sequences into a high-dimensional sparse feature matrix, with optional TF and/or IDF weighting; (2) applying a dimensionality reduction technique to embed the sequences into a space of lower dimensionality; (3) identifying sequence similarity through the use of a k-nearest neighbor search algorithm.

Many text-mining algorithms, such as MinHash and SimHash [21], employ the bag-of-words model, which discards the ordering of tokens within a sequence and focuses solely on the presence or absence of each token, effectively encoding the sequence database as a high-dimensional sparse matrix [22]. We observed that, once sequences are encoded in this manner, overlap detection becomes analogous to performing a nearest-neighbor search in single-cell sequencing data, where both tasks rely on identifying similar items based on some similarity or distance metric in a high-dimensional sparse space (i.e., the sequence $\times$ token matrix or the cell $\times$ gene matrix). Based on this analogy, we hypothesized that dimensionality reduction and approximate nearest-neighbor (ANN) methods, which are widely used in single-cell data analysis, could also be applied to overlap detection with appropriate adaptations. Accordingly, we developed a new approach for overlap detection (Fig. 1) consisting of three main steps: feature extraction, DR, and ANN search, as described below.

**Feature extraction**. Given a dataset of *n* sequences, we uniformly sampled an alphabet of *m* k-mers and encoded the dataset as a high-dimensional matrix *X* with *n* rows and *m* columns, where each entry $X[i, j]$ represents the weight of *k*-mer *j* in sequence *i*. By default, the weights were binary, indicating the presence or absence of a *k*-mer. Optionally, term frequency (TF) and/or IDF transformations could be applied to adjust the weight of each *k*-mer based on its frequency across the dataset. Since each sequence typically contains only a small subset of all possible *k*-mers, the resulting matrix is sparse.

**Dimensionality reduction**. Although it is possible to directly apply nearest-neighbor algorithms to the high-dimensional feature matrix, doing so is often computationally expensive due to the large number of dimensions. To mitigate this, we applied dimensionality reduction techniques to project each high-dimensional feature vector into a lower-dimensional space, while aiming to preserve the relative distances between sequences.

**ANN search**. Since exact *k*-nearest neighbor (k-NN) search has quadratic time complexity, it becomes infeasible for large genomic sequencing datasets with millions of reads. To address this, we employed approximate k-NN algorithms to identify the most similar sequences for each query sequence in the dataset. These candidate overlaps were then used to define edges in the resulting overlap graph.

Many overlap detection tools, such as minimap2 and MHAP, aim to identify all pairs of overlapping reads in a dataset. Instead, fedrann employs a *k*-nearest neighbors (k-NN) search to retrieve only the top *k* most similar reads for each query read. These nearest neighbors are expected to exhibit the longest overlaps with the query. This approach aligns with the concept of the *best overlap graph* [5, 23], which is grounded in the observation that full enumeration of overlapping read pairs is unnecessary, as shorter overlaps are typically redundant and pruned during graph refinement. For instance, the Shasta assembler retains by default only the top six edges per vertex in its overlap graph [6]. Restricting the number of edges in the overlap graph is advantageous, as it significantly reduces the complexity of subsequent graph layout computations.

**Figure 2:**
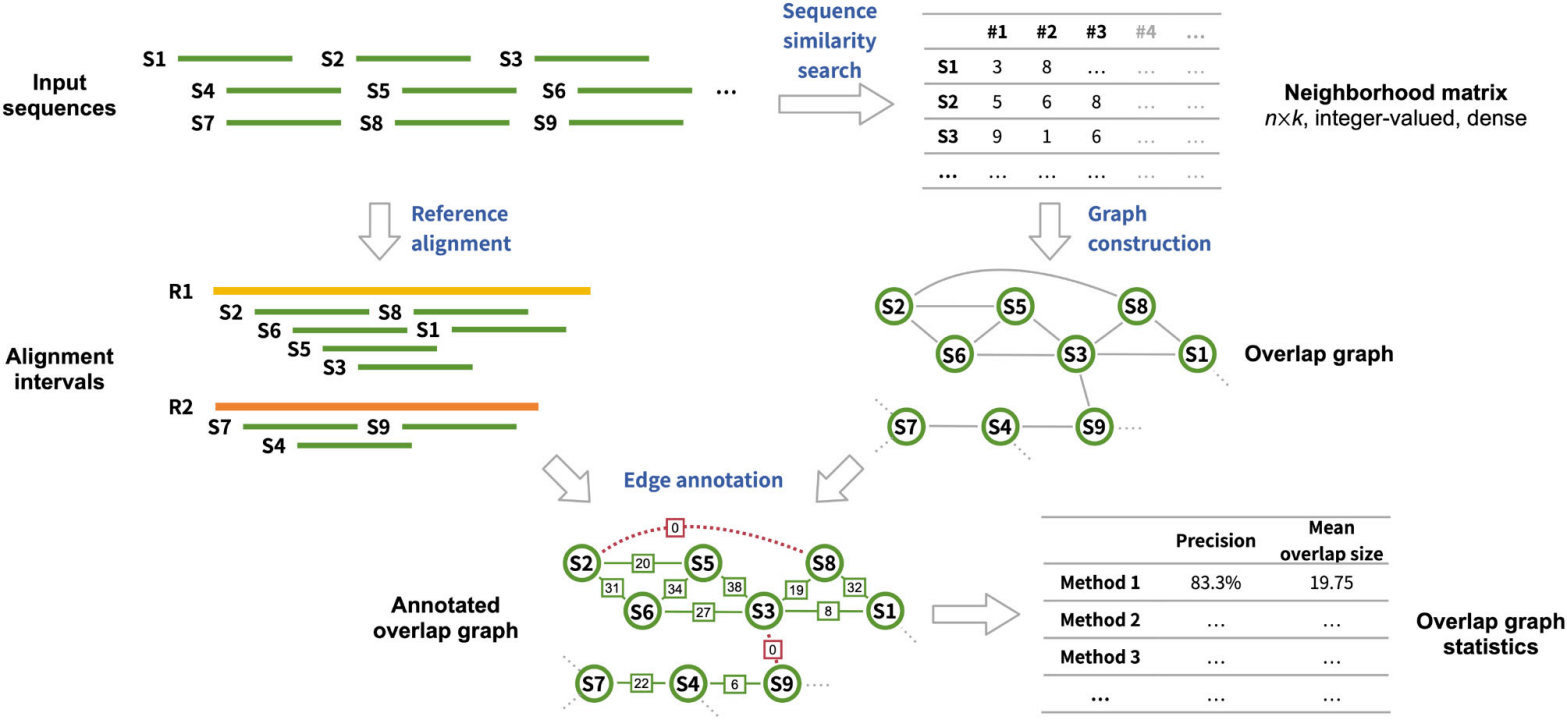
Assessment of sequence similarity search algorithms. For the analysis of each algorithm, an overlap graph is constructed by linking each query sequence to the top *k* sequences identified as most similar by the algorithm. This overlap graph is subsequently assessed against a reference graph, which is obtained by aligning the input sequences to a reference genome to ascertain the accuracy and overlap size of each edge. The algorithm’s performance is evaluated based on precision and mean overlap size.

To evaluate the performance of specific combinations of feature extraction, dimensionality reduction, and *k*-nearest neighbors (k-NN) search methods, we constructed overlap graphs for multiple long-read sequencing datasets and compared them against reference graphs derived from corresponding reference genomes (Fig. 2). The datasets included sequencing reads from Oxford Nanopore Technologies (ONT), PacBio HiFi, and CycloneSEQ, all sampled from three repeat-rich regions of the human genome: the *HLA* immunogene cluster, the *IGK* immunoglobulin $\kappa$-light chain locus, and chromosome 22 (Supplementary Table S1). The HLA and IGK regions each contain over 40% repetitive elements and are of notable medical relevance [24], while chromosome 22 encompasses large heterochromatic segments. For each method combination, performance was quantified by the error rate of the resulting overlap graph, defined as the proportion of incorrect edges among all edges.

### IDF transformation improves overlap detection accuracy

We first evaluated the impact of different weighting schemes (TF-IDF, TF, IDF, and raw TF) during feature extraction, and compared similarity metrics (Euclidean distance and cosine distance) used in k-NN search. As the sizes of the three benchmarking regions were relatively small (3.92–5.75 Mb), we were able to use k-d tree based exact k-NN search without dimensionality reduction to inspect the upper limit of the FEDRANN strategy.

**Figure 3:**
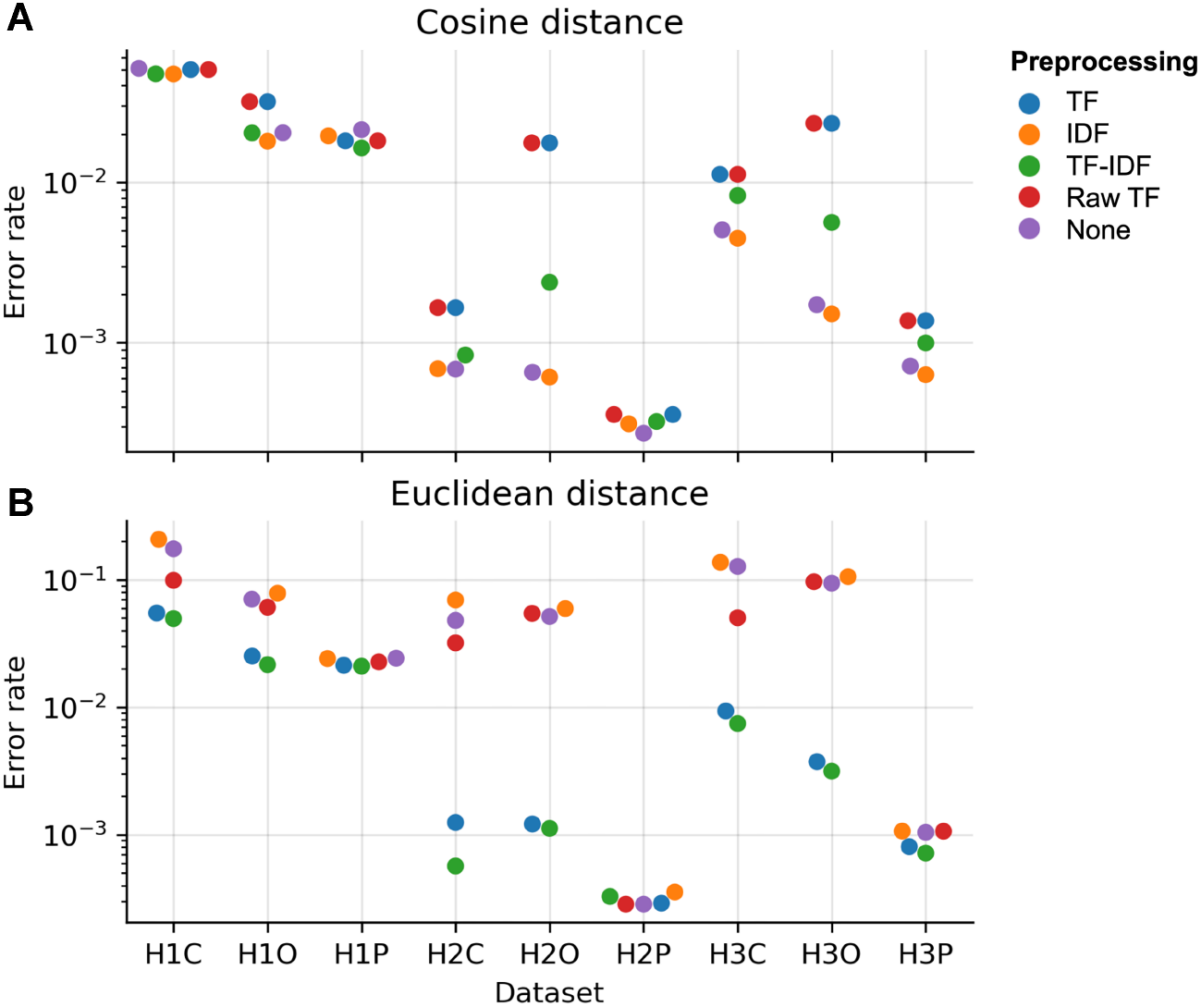
Assessment of preprocessing methods and similarity metric for overlap detection. (A, B) Overlap detection error rate of pipelines combining various preprocessing methods for feature extraction (TF, IDF, TF-IDF, Raw TF, and no preprocessing) and exact k-NN search based on cosine (A) or Euclidean (B) distance. Top six neighbors according to each distance metric were used to construct overlap graphs for evaluation. Abbreviations: TF, term frequency; IDF, inverse document frequency.

Our results showed that using cosine distance for ENN search resulted in significantly higher accuracy compared to Euclidean distance (Figs. 3, Supplementary Figs. S1 and S2). This is likely because Euclidean distance is sensitive to vector magnitude, while cosine distance captures only the angular difference between vectors, making it more robust to variations in sequence length. As a result, overlapping sequences of different lengths may still exhibit low cosine distances but high Euclidean distances, leading to better overlap detection when cosine similarity is used.

We evaluated the impact of different weighting schemes (TF, IDF, IDF, and raw TF) on overlap graph quality. For baseline comparison, we also constructed a binary feature matrix that only records the presence (1) or absence (0) of *k-*mers in each read, discarding all frequency and weighting information. While TF-IDF has been used in assemblers like Canu [5] to help resolve repetitive sequences, our experiments indicated that IDF alone, rather than TF or TF-IDF, was more effective in improving graph quality. When cosine distance was used, applying IDF weighting led to better performance than both TF-IDF and unweighted representations, whereas TF alone and raw TF degraded performance relative to the unweighted baseline (Figs. 3, Supplementary Figs. S1 and S2). These findings suggest that high-frequency *k*-mers—such as those originating from transposable elements, short tandem repeats, or other repetitive regions—are a major source of error in overlap graph construction. By down-weighting these features, IDF enhances the ability to distinguish between true overlaps. In contrast, TF and raw TF emphasize frequent features and may counteract the benefits of IDF, thereby reducing accuracy.

We plotted the overlap graph error rate as a function of the number of overlap candidates (*k*). As expected, the error rate increased with larger values of *k*, reflecting the greater difficulty in identifying larger numbers of correct overlaps. Notably, the combination of IDF transformation and cosine distance consistently outperformed other method combinations in terms of accuracy. Based on this observation, we selected the IDF–cosine distance combination for subsequent analyses, including evaluating the effects of read length, coverage depth, and sequencing accuracy, as well as for selecting DR and ANN search methods, as described below.

### Longer reads, deeper coverage, and higher accuracy improve overlap detection

We systematically evaluated the impact of read length, coverage depth, and sequencing accuracy to overlap detection by generating simulated datasets with PBSIM3 for human chromosome 22, covering read lengths of 10–30 kb, accuracy of 91–99%, and depths of 10×–50×. We found that longer reads and deeper coverage had a large positive effect on overlap graph accuracy (Supplementary Fig. S3). We hypothesize that this was because longer reads and deeper coverage both lead to longer overlaps between adjacent reads, facilitating overlap detection. Meanwhile, improved sequencing accuracy only had a moderate positive effect on overlap graph accuracy. For example, at 30× coverage and mean read length 20 kb, increasing sequencing accuracy from 91 to 99% only reduced error rate from 8.34 to 5.55% for human chromosome 22. This observation suggested that our FEDRANN strategy is relatively robust to sequencing errors.

### SRP enables scalable and accurate dimensionality reduction

DR techniques are widely used in single-cell sequencing analyses to reduce noise, accelerate computation, and facilitate data visualization. To evaluate their utility in overlap graph construction, we tested a range of DR methods, including linear approaches such as principal component analysis (PCA) [25] and SRP, as well as non-linear methods including Uniform Manifold Approximation and Projection (UMAP) [26], Spectral Embedding, and scBimapping. We also evaluated SimHash [27], a LSH–based method related to random projection, which has been widely used in text mining and recently applied to biological sequence analysis [18, 28].

**Figure 4:**
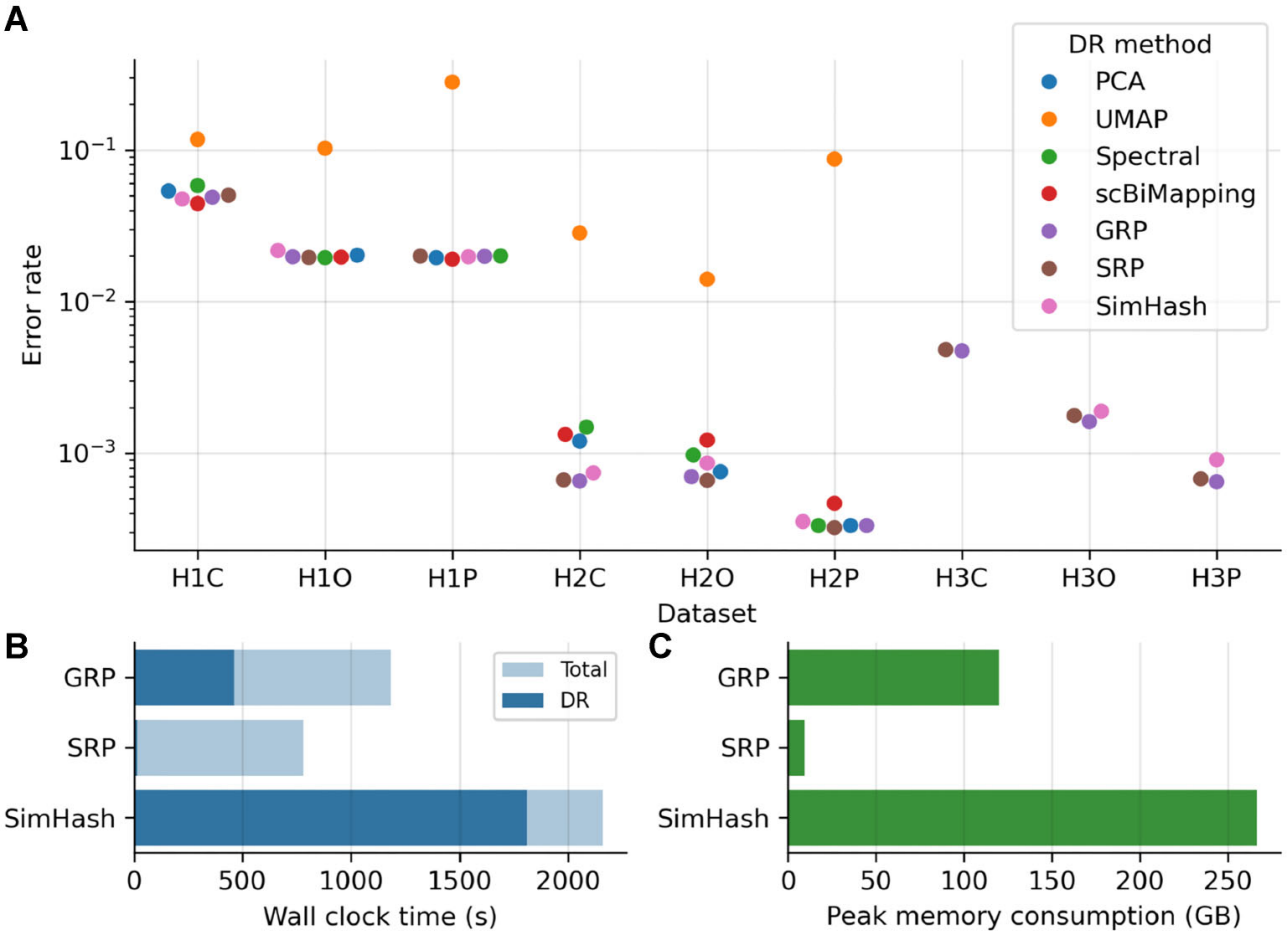
Assessment of DR methods. (A) Overlap detection error rate of various DR methods. IDF preprocessing was used in feature extraction. Exact Hamming distance (for SimHash) or cosine distance (for other methods) was used in k-NN search. Top six neighbors according to each distance metric were used to construct overlap graphs for evaluation. Missing dots indicate that the corresponding methods failed to generate results under predefined computational constraints (wall clock time $\le$ 6 h and peak memory $\le$ 900 GB). (B, C) Wall clock time (B) and peak memory consumption (C) of various DR methods in dataset H3P. Methods that failed to generate results are not shown. Abbreviations: PCA, principal component analyses; UMAP, Uniform Manifold Approximation and Projection; Spectral, spectral embedding; GRP, Gaussian random projection; SRP, sparse random projection; DR, dimensionality reduction.

For each method, we first constructed the input feature matrix using IDF weighting, then applied the DR method, and finally computed exact *k*-nearest neighbors (ENN) from the resulting low-dimensional embeddings (Fig. 1). We found that all tested DR methods except UMAP were able to generate accurate overlap graphs for smaller datasets (Fig. 4A), suggesting that they preserved the relative pairwise distances between sequences sufficiently well. However, when applied to larger datasets, most methods failed to complete within the predefined resource constraints (wall clock time $\le$ 6 h; peak memory $\le$ 1 TB) (UMAP), or the matrix size surpassed the maximum constraints of the methods (PCA, Spectral, and scBiMapping), resulting in dimensionality reduction failure. The only methods that successfully scaled to the largest datasets, H3C, H3O, and H3P, were SRP, GRP, and SimHash. Time and memory profiling on dataset H3P further confirmed that SRP was the fastest method and had the lowest memory usage among those tested (Fig. 4B). Based on this robust balance between distance preservation and computational efficiency, SRP was selected as our DR method for further benchmarking.

The accuracy of *k*-NN search after DR typically depends on the embedding dimension, as higher dimensions allow more information from the original feature matrix to be retained. To characterize this relationship, we varied the embedding dimension for each dataset using SRP. The results revealed that increasing the embedding dimension enhances the “feature resolution” of the reads, enabling the ANN algorithm to more effectively resolve ambiguities in repetitive or highly similar genomic regions (Supplementary Figs. S4 and S5). To better understand this trend, we examined the correlation between pairwise cosine distances before and after SRP in dataset H3C. The results showed a strong positive correlation that steadily improved with increasing dimensions, with the Pearson $R^2$ rising from 0.72 at 100 dimensions to 0.99 at 3,000 dimensions (Supplementary Fig. S6). This indicates that higher-dimensional projections achieve near-perfect structural fidelity to the original high-dimensional data. Furthermore, we analyzed the correlation between post-reduction cosine distances and actual overlap sizes. As dimensions increased, the $R^2$ significantly improved from 0.43 to 0.83, demonstrating that higher dimensions allow the embedding space to more accurately represent the underlying genomic relationships (Supplementary Fig. S6). However, the observed “diminishing returns” (Supplementary Figs. S4) suggest an informational saturation point; for most datasets, 1,000 dimensions are sufficient to capture the essential variance of the *k*-mer distribution. Beyond this threshold, additional dimensions primarily capture redundant information or noise, offering marginal precision gains at the expense of higher memory and computational overhead (Supplementary Figs. S13).

### NNDescent offers the best trade-off between speed and accuracy among ANN methods

In addition to dimensionality reduction, another strategy to accelerate k-NN search is to use approximate k-NN (ANN) algorithms, which leverage indexing structures or heuristics to reduce the computational cost of neighbor retrieval. We evaluated five ANN approaches that are widely used in single-cell sequencing and information retrieval applications: NNDescent [27], hierarchical navigable small world (HNSW) [29], product quantization (PQ) [30], inverted file index with product quantization (IVF-PQ) [30], and random projection forest (RPF) [31]. All ANN methods were applied to low-dimensional embeddings generated by SRP.

**Figure 5:**
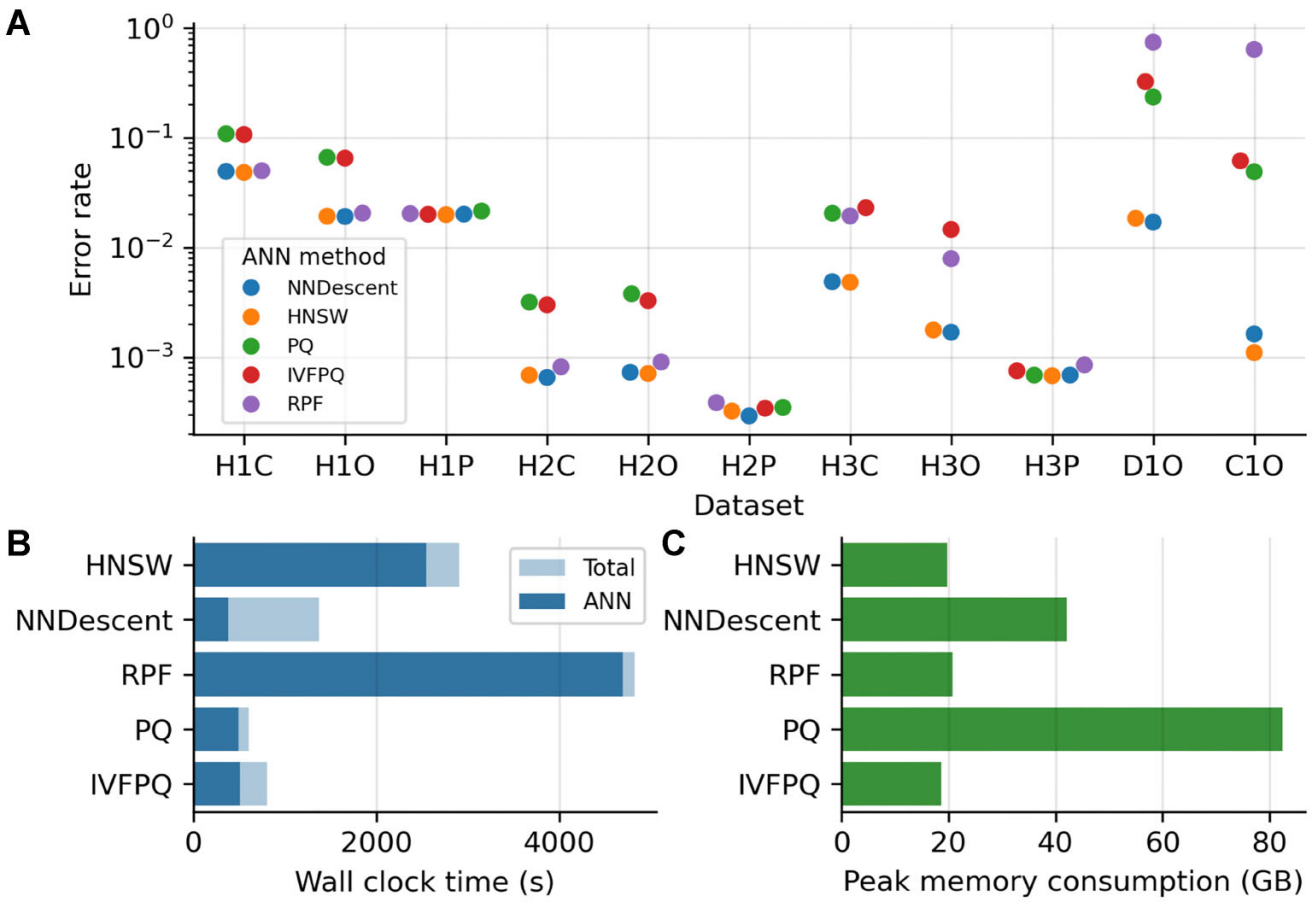
Assessment of ANN methods. (A) Overlap detection error rate of various ANN methods. IDF preprocessing was used in feature extraction. Feature matrices were reduced to 1,000 dimensions using SRP prior to ANN search. Cosine distance was used in ANN search. Top six neighbors were used to construct overlap graphs for evaluation. Missing dots indicate that the corresponding methods failed to generate results under predefined computational constraints (wall clock time $\le$ 6 h and peak memory $\le$ 900 GB). (B, C) Wall clock time (B) and peak memory consumption (C) of selected ANN methods in dataset D1O. Abbreviations: ANN, approximate *k*-nearest neighbors; HNSW, hierarchical navigable small world; PQ, product quantization; IVFPQ, inverted file with product quantization; RPF, random projection forest.

Among these methods, NNDescent and HNSW achieved high precision when used to construct overlap graphs, whereas PQ, IVF-PQ, and RPF performed less favorably in terms of accuracy (Fig. 5A). In terms of computational efficiency, NNDescent and IVF-PQ were the fastest, while RPF consumed the least memory (Fig. 5B–C). Across all configurations, the ANN step emerged as the computational bottleneck in the FEDRANN pipeline, often requiring significantly more time than the SRP step (Fig. 5B), although both steps had comparable peak memory usage (Figs. 4C and 5C). Consequently, NNDescent was selected as the optimal ANN method for our workflow, as it offered the best trade-off between speed and accuracy for scalable overlap graph construction.

### Efficient implementation of the IDF-SRP-NNDescent pipeline

The experimental results established the combination of IDF, sign-random projections (SRP), and NNDescent as a novel framework for constructing overlap graphs from genomic sequencing data. To transition this theoretical finding into a functional application, we developed Fedrann, an overlap detection tool optimized for processing large-scale, long-read datasets (Supplementary Fig. S7; see *Methods*).

Fedrann utilizes a memory-aware architecture designed to circumvent the memory bottlenecks typically associated with high-dimensional feature matrices. By avoiding full matrix materialization, the tool enables scalable analysis of human-scale genomic data. We implemented batch-wise processing for the computationally intensive upstream stages, including feature construction, weighting, and dimensionality reduction. In this workflow, sparse *k*-mer feature matrices are generated on demand and transformed incrementally in fixed-size batches. This ensures that intermediate data structures remain compact and transient. Consequently, peak memory consumption is governed by the batch size and the sampled *k*-mer alphabet rather than the total read count, providing a significant advantage over methods reliant on global feature matrices.

During the pre-processing stage, we leverage Jellyfish [32] to construct the *k*-mer library and filter low-frequency *k*-mers based on their occurrence statistics. Jellyfish outputs the total number of occurrences (i.e. collection frequency, CF [33]) of each *k*-mer across the sequence dataset. Our empirical analysis demonstrates that, on a logarithmic scale, CF is nearly identical to DF—the number of reads containing a specific *k*-mer—with an $R^2$ value reaching 0.9990 (see Supplementary Fig. S8). Based on this observation, we implemented inverse collection frequency (ICF) as a high-performance surrogate for the conventional IDF. This approach allows us to directly utilize pre-computed Jellyfish statistics for effective feature weighting, thereby bypassing the need for an additional, computationally intensive *k*-mer counting step.

An additional implementation optimization is the fusion of linear transformations. Because IDF reweighting and DR are both linear operations, they are combined into a single weighted projection matrix that is reused across all batches. This design eliminates intermediate feature transformations, reduces memory traffic, and improves computational efficiency, particularly under parallel execution. After DR, ANN search is performed on the full embedding matrix using pyNNDescent [34], which is itself highly optimized for efficiency and scalability. Together, these design choices enable Fedrann to scale efficiently to large long-read datasets while maintaining manageable computational and memory demands.

### Fedrann enables high-precision, time-efficient overlap detection on large genomes

We benchmarked Fedrann against a set of established overlap detection tools: minimap2 (in all-vs-all mode), MECAT2, MHAP, BLEND, wtdbg2, and xRead. Benchmarking was performed on real whole-genome sequencing (WGS) datasets from three species with differing genome sizes and complexities: *Caenorhabditis elegans, Drosophila melanogaster*, and *Homo sapiens* (Supplementary Table S1).

The overlap detection tools benchmarked in this study exhibit distinct trade-offs between precision and recall. Most tools—specifically minimap2, MECAT2, MHAP, BLEND, and wtdbg2—are designed to identify all potential overlapping pairs, generally prioritizing high recall. In contrast, xRead returns only a subset of the top overlap candidates per read, sacrificing recall for enhanced precision [14]. Fedrann follows a similar strategy by returning the top *k* candidates for each read, though higher recall can be achieved by increasing the value of *k*. To evaluate these differences, we performed a precision-recall analysis using the H3C dataset (Supplementary Fig. S9). To ensure a fair comparison, we post-processed the outputs of all tools to retain only the top *k* candidates per read, calculating precision and recall across a range of *k* values. Our analysis revealed that when *k* is lower than the expected number of true overlaps per read (a function of coverage depth and read length), the recall for most tools (excluding xRead) increases linearly with *k* while precision remains relatively stable. In this regime, MECAT2 and Fedrann demonstrated the highest precision. Once *k* exceeds the actual number of true overlaps, recall plateaus while precision begins to decline. This drop in precision was most pronounced for Fedrann, which is expected as the algorithm is configured to output exactly *k* candidates, whereas other tools employ internal thresholds to filter low-confidence overlaps. Stratifying target reads by overlap length confirmed that Fedrann prioritizes the detection of longer overlaps, with shorter overlaps being identified at larger *k* values (Supplementary Fig. S10).

Real-world *de novo* assembly applications present unique requirements and challenges. For example, longer overlaps are generally more valuable than shorter ones, as the latter are frequently pruned as transitive edges during graph simplification. Furthermore, an assembly graph must maintain high continuity rather than being fragmented into isolated subgraphs. Finally, the overlap detection tool must scale efficiently to large genomic datasets containing millions of reads. To evaluate performance under these demanding conditions, we benchmarked the tools on human WGS datasets using four key metrics: error rate ($1 - \mathrm{precision}$), mean overlap size, the number of correct overlap candidates per read (#COC), and the number of connected components (#CC). Mean overlap size assesses the ability to identify the longest, most informative overlaps for each read. Higher #COC values indicate superior per-read connectivity, whereas lower #CC values reflect reduced fragmentation and higher global contiguity. Additionally, we recorded the computational resource usage, including execution time and peak memory consumption, for each tool.

**Figure 6:**
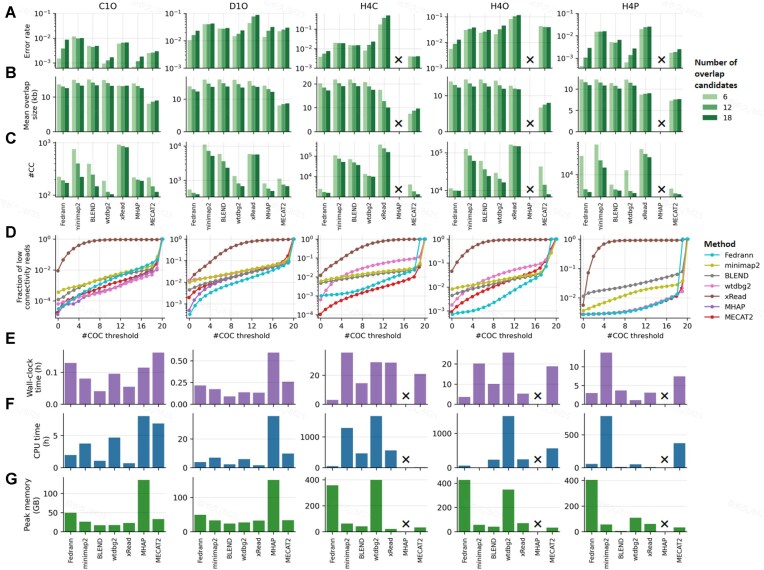
Benchmarking of overlap detection tools. (A–C) Overlap detection error rate (A), mean overlap size (B), and #CC (C) of various overlap detection tools in five whole-genome datasets (columns). Top 6, 12, or 18 overlap candidates (colors) according to cosine distance (for Fedrann) or overlap size (for other methods) were used to construct overlap graphs for evaluation. (D) Fraction of low-connectivity reads plotted against #COC threshold for various overlap detection tools. (E–G) Wall-clock time (E), CPU time (F), and peak memory (G) usage of various overlap detection tools. In (A–C) and (E–G), black crosses indicate that the corresponding methods failed to generate results under predefined computational constraints (wall clock time $\le$ 72 h and peak memory $\le$ 900 GB). These methods were not included in (D). Abbreviations: #CC, number of connected components; #COC, number of correct overlap candidates.

A memory limit of 950 GB was enforced during benchmarking. Under this constraint, MHAP failed to complete on all three human datasets (H4C, H4O, and H4P), and xRead failed on H4C. All other tools completed successfully (Fig. 6). Fedrann achieved the highest accuracy on four out of five datasets (Fig. 6A); the exception was H4C, where MECAT2 performed slightly better. wtdbg2 also showed strong accuracy, consistently ranking second or third across datasets. In terms of mean overlap size, MECAT2 consistently ranked lowest, while other tools performed comparably (Fig. 6B). For graph contiguity, Fedrann, wtdbg2, MHAP, and MECAT2 generally outperformed the other methods, with no clear single leader (Fig. 6C). Analysis of low-connectivity reads under varying #COC thresholds showed that xRead produced a large number of poorly connected reads—for instance, in dataset H4P, over 50% of reads had fewer than five correct edges. Fedrann ranked first or second across all datasets (Fig. 6D).

In terms of computational cost, all tools completed the smaller datasets (C1O and D1O) within 1 h and with moderate memory usage (<60 GB), except MHAP, which consumed up to 150 GB (Fig. 6E–G). In the context of the larger human datasets (H4C, H4O, H4P), where computational efficiency is paramount, Fedrann demonstrated superior performance. It achieved the shortest execution times on the H4C (3.02 h) and H4O (3.58 h) datasets. On the H4P dataset, Fedrann was the second fastest tool (2.97 h), trailing only wtdbg2. Notably, while wtdbg2 required over 20 h to process the H4C and H4O datasets, its execution time significantly decreased to 1.1 h for the H4P dataset. The memory efficiency of the evaluated tools varied significantly across the human genome datasets. Minimap2, BLEND, xRead, and MECAT2 maintained relatively low memory footprints, all requiring less than 100 GB. In contrast, Fedrann exhibited higher memory demands, with peak usage ranging between 350 and 450 GB. The memory consumption of wtdbg2 was comparable to Fedrann on the H4C and H4O datasets; however, it demonstrated significantly higher efficiency on the H4P dataset, where its peak memory usage dropped to approximately 100 GB.

In summary, Fedrann, wtdbg2, and MECAT2 emerged as the best-performing tools when considering both graph quality and computational efficiency. Fedrann offered a balanced trade-off across accuracy, overlap size, graph contiguity, and speed, though it incurred a high memory footprint. wtdbg2 was accurate and memory-efficient but lagged in contiguity, connectivity, and speed (except for HiFi data). MECAT2 was both accurate and efficient in memory usage, but failed to capture some of the longest overlaps. Overall, our results highlight Fedrann as a robust and competitive new tool for long-read overlap detection.

### The Fedrann-Shasta pipeline generates high-quality human whole-genome assembly contigs

To demonstrate the practical utility of Fedrann within end-to-end overlap-layout-consensus (OLC) frameworks, we developed an integrated assembly pipeline by modifying the Shasta assembler [6]. While Shasta natively employs a MinHash variant for overlap discovery, our modified version ingests Fedrann’s output to construct the initial overlap graph. Using this Fedrann-Shasta pipeline, we performed *de novo* assembly of the human genome using the H4C and H4O datasets. The results were benchmarked against assemblies generated by the unmodified Shasta assembler using otherwise identical parameters (Supplementary Table S6).

For the H4C dataset (raw read N50 of 40.67 kb), the Fedrann-Shasta assembly achieved a contig N50 of 44.45 Mb, with the longest contig spanning 110.3 Mb (Supplementary Fig. S11). The assembly demonstrated high genomic fidelity with a BUSCO completeness of 98.10%. Notably, chromosomes 3, 4, 12, and 20 were assembled nearly to completion, with discontinuities appearing only at centromeric and telomeric regions (Supplementary Fig. S11). These metrics are highly comparable to those of the native Shasta assembly (N50 45.07 Mb, longest contig 137.99 Mb, BUSCO 98.30%). In contrast, on the H4O dataset (shorter raw-read N50 of 23.06 kb), Fedrann-Shasta significantly outperformed the native assembler, yielding a contig N50 of 17.07 Mb—a three-fold increase over the 5.73 Mb N50 produced by Shasta. *K*-mer based phred-scale quality values (QV) for the H4O assemblies ( 37.5) were slightly higher than those of the H4C assemblies ( 35.7) and remained consistent regardless of the assembly method used.

To assess the impact of Fedrann on resolving complex, repeat-rich regions, we analyzed the HLA region within both H4C assemblies (Supplementary Fig. S12). Both pipelines partitioned the HLA region into two contigs with a breakpoint at roughly the same location. However, the native Shasta assembly exhibited a collapse of a  40 kb repeat unit, incorrectly merging two copies into one. This misassembly was correctly resolved in the Fedrann-Shasta assembly, suggesting that Fedrann’s high-accuracy overlap detection may improve assembly quality in repetitive genomic contexts.

This proof-of-concept study confirms that Fedrann is a robust alternative for overlap detection in large-scale *de novo* assembly, capable of producing high-quality human genome reconstructions. While these results are promising, further engineering of the alignment refinement stage may continue to enhance overall assembly contiguity and accuracy.

### Application notes

We provide the following recommendations for users applying Fedrann to large-scale genomic datasets. The software utilizes a transparent parameter space where most variables exhibit predictable linear trade-offs: increasing parameter values generally enhances predictive accuracy at the cost of increased memory consumption and execution time. For human genome-scale applications, our parameter sensitivity analysis (Supplementary Fig. S13A) suggests that a configuration of 1,000 dimensions for dimensionality reduction, 600 trees for NNDescent, and a 15% *k*-mer sampling fraction provides an optimal balance between accuracy and computational footprint. Critically, *k*-mer size selection is governed by the specific error profiles and sequence characteristics of the sequencing platform rather than computational scaling alone. For optimal performance, we recommend the following empirical defaults based on our whole-genome evaluations: $k=31$ for PacBio HiFi, $k=13$ for ONT, and $k=11$ for CycloneSEQ (Supplementary Fig. S13B). Detailed instructions on optimizing Fedrann parameters are included in Supplementary Note 1.

For human genome applications, Fedrann typically requires a memory footprint of 300–500 GB. In environments where this capacity is unavailable, the pipeline remains functional on machines with lower RAM (e.g., 256 GB) by leveraging system swap space (Supplementary Table S7). At a 256 GB limit using HDD-based swap, Fedrann successfully completed the task in 15.69 h, producing results identical to the unconstrained baseline (3.02 h), despite both configurations utilizing 64 threads. Notably, the use of high-speed Solid State Drives (SSDs) for the swap partition significantly accelerated the computation; the SSD-based configuration finished in 14.87 h using only 32 threads, effectively outperforming the 64-thread HDD execution. While relying on swap space substantially increases total execution time due to disk I/O bottlenecks, it provides a viable pathway for users to process human whole-genome datasets on systems with limited physical memory. Detailed instructions on running Fedrann with limited physical memory are included in Supplementary Note 2.

## Discussion

We proposed a novel strategy for overlap graph construction that integrates DR and ANN techniques. We conducted comprehensive evaluations on both simulated and real long-read sequencing datasets. Among the tested combinations, the pipeline consisting of IDF transformation, SRP, and NNDescent achieved the best trade-off between accuracy and computational efficiency. Based on these findings, we developed an efficient, open-source, and user-friendly implementation named Fedrann. Benchmarking results showed that Fedrann generated overlap graphs that were comparable to or better than those produced by state-of-the-art tools such as MECAT2 and wtdbg2, in terms of both overlap accuracy and contiguity for large genomes, without sacrificing computational speed.

Although the concept of treating overlap detection as a special case of k-NN search has been suggested in previous studies [6, 23], ANN search methods have not been directly applied to overlap identification. This omission is likely due to computational constraints: feature matrices derived from the human genome often contain millions of sequences, each represented by millions or even billions of features, posing a significant challenge even for state-of-the-art ANN algorithms such as HNSW and NNDescent. Common dimensionality reduction techniques, including PCA and UMAP, also fail to scale effectively to matrices of this magnitude. In this study, we identify SRP as a highly scalable dimensionality reduction method that preserves pairwise sequence distances (Figs. 4 and Supplementary Fig. S6), enabling accurate and efficient overlap detection via NNDescent, thereby unlocking new possibilities for overlap detection in the assembly of large genomes.

The current implementation of Fedrann is written primarily in Python and builds upon general-purpose numerical libraries rather than domain-specific sequence processing frameworks. Nevertheless, Fedrann achieves competitive performance, constructing overlap graphs for the human genome within 4 h, faster than many state-of-the-art overlap detection tools implemented in compiled languages such as C and C++. This efficiency largely stems from formulating overlap detection as a sequence of matrix operations, enabling extensive use of highly optimized dense and sparse linear algebra routines provided by NumPy and SciPy. Representing sequence data in matrix form allows Fedrann to benefit from vectorization, cache-friendly memory access, and parallel execution, mitigating much of the overhead typically associated with high-level languages. SRP plays a key role in this design by offering both algorithmic simplicity and computational efficiency: it is data-independent, inexpensive to compute, and naturally compatible with incremental, batch-wise processing, making it particularly well suited to large-scale overlap detection. Despite these strengths, the overall memory consumption of Fedrann remains higher than that of most existing overlap detection tools benchmarked, highlighting an important direction for future optimization through more memory-efficient data representations, shared-memory designs, or alternative implementations of ANN methods.

Beyond computational performance, another strength of using matrix-based representations is the clarity and modularity of the pipeline. Most parameters in Fedrann have well-defined meanings and predictable effects on the output, typically reflecting trade-offs between overlap detection accuracy and resource usage, without being tightly coupled to a specific sequencing platform. For instance, increasing the embedding dimension in SRP generally improves accuracy at the cost of additional computation. This platform-agnostic design allows Fedrann to be easily adapted to emerging sequencing technologies with minimal parameter tuning.

To showcase the practical utility of Fedrann in a full assembly workflow, we developed the Fedrann-Shasta pipeline to generate high-quality human *de novo* assemblies, illustrating the feasibility of modular frameworks that utilize Fedrann for overlap detection. In many existing assemblers, intermediate results such as overlap graphs are encapsulated in opaque, in-memory data structures. While this design is often motivated by computational efficiency, it creates a tight coupling between pipeline stages that limits transparency and reusability. Consequently, incorporating Fedrann into existing tools is non-trivial, requiring deep expertise in undocumented internal structures. Because this integration is currently constrained by the internal architecture of established tools, we consider our current Fedrann-Shasta results a successful proof-of-concept rather than the upper limit of Fedrann’s potential. The lack of modularity in existing tools often forces developers to build new assemblers from the ground up as sequencing technologies emerge. We hope that modular tools like Fedrann, which expose transparent and interpretable data structures, will lower these barriers and facilitate the development of more adaptable next-generation assembly algorithms.

Finally, we note that overlap graph construction can be regarded as a special case of the broader sequence similarity search (SSS) problem, where the objective is to retrieve the most similar sequences for each query from a large reference database. The use of DR and ANN methods for SSS has been explored in previous studies. For instance, the Rust library annembed [35] combines LSH with HNSW graphs to perform metagenomic binning, a task that clusters contigs or reads from metagenomic sequencing into bins ideally representing individual microbial genomes. Our results demonstrate FEDRANN as a robust and scalable framework for sequence similarity detection, with potential applicability to a variety of SSS tasks, including metagenomic binning. Another promising application is taxonomic classification in metagenomics, where query sequences (reads or contigs) are classified against a static, pre-indexed database of microbial reference genomes. Unlike overlap detection or binning, which typically operate on all input sequences jointly, classification often requires rapid querying of individual sequences against the database. The IDF-SRP-NNDescent pipeline investigated here is well-suited for such classification tasks. Both IDF and SRP are linear, independent transformations, while NNDescent supports incremental and efficient nearest neighbor search. Nonetheless, further development and empirical validation are needed to assess the performance of FEDRANN in metagenomic binning, taxonomic classification, and other long-read sequence analysis applications.

## Methods

### Fedrann implementation

We implemented the IDF–SRP–NNDescent pipeline as a standalone command-line tool for overlap detection, named Fedrann (Supplementary Fig. S7). Fedrann accepts FASTA or FASTQ files as input and identifies a fixed number of candidate overlaps (default: $k=20$) for each sequence. The tool generates a tab-separated values file characterizing sequence overlaps through the reporting of sequence indices, distance metrics, and neighbor ranks.

The implementation is primarily written in Python and leverages C-accelerated numerical libraries for efficiency, including optimized sparse and dense matrix operations provided by NumPy [36] and SciPy [37]. Together with extensive parallelization and memory-aware design choices, these optimizations enable scalable application to human-scale long-read sequencing datasets.

#### Feature extraction

The pipeline utilizes Jellyfish for efficient *k-*mer counting and filtering, retaining only *k-*mers whose multiplicities exceed a predefined threshold to remove noise arising from sequencing errors. From the filtered set, a *k*-mer alphabet is constructed by uniformly sampling a predefined fraction, typically between 5 and 20%.

Although the DF of a *k-*mer is conventionally defined as the number of reads containing that *k-*mer, we found no existing tools capable of computing this quantity efficiently at scale. Fedrann therefore adopts the total count (i.e. collection frequency, CF) of a *k-*mer within the dataset as a computationally efficient approximation of its DF, enabling direct use of Jellyfish’s high-performance *k-*mer counting. The implementation relies on Jellyfish’s canonical *k-*mer representation, in which the count of each *k-*mer reflects the combined frequency of the sequence and its reverse complement. Under this approximation, the IDF of *k-*mer *j* in dataset *D* is defined as


\begin{eqnarray*}
idf(j, D) = \log \frac{\vert N \vert }{c_j},
\end{eqnarray*}

where $\vert N \vert$ denotes the total number of reads in *D* and $c_j$ is the total count of *k-*mer *j* in the dataset.

To construct the feature matrix, a custom multi-threaded C++ program is used to locate each selected *k-*mer within every read, as well as within its reverse complement. The resulting *k-*mer–read associations are written to a compact binary file, which is subsequently loaded in batches to assemble a sparse feature matrix on demand. Rather than explicitly materializing this high-dimensional matrix, each sparse batch is streamed directly into the downstream dimensionality reduction stage.

#### Dimensionality reduction

Leveraging the fact that both IDF weighting and SRP are linear operations, the implementation fuses these steps by pre-multiplying the SRP projection matrix with the IDF weight vector. Concretely, an SRP matrix $\mathbf {R} \in \mathbb {R}^{n \times m}$ is first generated, where *n* denotes the number of sampled *k-*mers and *m* is the target reduced dimension, and is then multiplied by the IDF weight vector of size $n \times 1$ to form an integrated weighted projection matrix. This intermediate matrix is stored in shared memory, enabling concurrent access by multiple child processes without redundant data copying.

During processing, sparse feature batches of size $b \times n$ (default: $b=100{,}000$ reads), loaded from the binary file, are independently transformed via matrix multiplication with the shared weighted projection matrix. The resulting low-dimensional embeddings are written to a shared dense output array. This high-concurrency, incremental architecture eliminates the need to materialize a monolithic feature matrix, thereby avoiding memory bottlenecks and ensuring a manageable memory footprint while enabling efficient processing of human-scale genomic datasets.

#### Approximate nearest neighbor search

Approximate nearest neighbor search is performed on the resulting embedding matrix using the PyNNDescent library [34], which efficiently constructs a neighborhood graph in a highly parallelized manner. For each sequence, the algorithm retrieves its top-*k* nearest neighbors (default: $k=20$) along with the corresponding distance scores. The resulting neighborhood graph is then converted into a candidate overlap table and written to disk as the final output of Fedrann.

### Fedrann-Shasta pipeline

The Fedrann-Shasta pipeline is an integrated framework for long-read genome assembly that couples Fedrann’s overlap detection with a modified version of the Shasta assembler [6]. In this configuration, Fedrann functions as the upstream discovery module, generating the initial set of candidate overlaps that are subsequently refined by Shasta.

To facilitate this integration, we implemented a dedicated entry point within the Shasta source code that bypasses the native MinHash-based discovery stage. A custom loading function ingests the Fedrann output and performs the following operations:

- **ID mapping**: Translates read names to Shasta’s internal read identifiers.
- **Parsing and deduplication**: Processes the candidate list to eliminate redundant read pairs.
- **Candidate injection**: Populates Shasta’s alignment candidate container with oriented read pair entries.

Once injected, Shasta computes marker-based alignments for each candidate pair and applies its standard quality criteria. The overlap graph (referred to as the “read graph” in Shasta terminology) is then constructed from these validated alignments, where vertices represent oriented reads and edges represent accepted overlaps. Consistent with the original Shasta design, only the best *k* alignments per read are retained to maintain graph efficiency. All subsequent assembly stages—including marker graph construction, graph simplification, and consensus generation—are performed using the unmodified Shasta pipeline.

### Sequencing datasets

To evaluate various overlap detection methods, we used genomic sequencing data from three model species: *Homo sapiens, Caenorhabditis elegans*, and *Drosophila melanogaster*. The corresponding reference genomes were obtained from the following sources: the complete human T2T-CHM13 assembly v2.0 [3], the *Caenorhabditis elegans* WBcel235 assembly (WormBase release WS285; NCBI Assembly ID: GCA_000002985.3), and the *Drosophila melanogaster* Release 6 genome assembly (NCBI Assembly ID: GCF_000001215.4).

Simulated sequencing datasets with varying read lengths, error rates, and coverage depths were generated using PBSIM3 [38], based on the ERRHMM-ONT-HQ model. Real sequencing datasets from Oxford Nanopore Technologies (ONT) and PacBio HiFi platforms were obtained from publicly available databases (Supplementary Table S1). ONT R10 sequencing data for HG002 was obtained from the Oxford Nanopore Technologies EPI2ME repository [39]. ONT R9 sequencing data for *C. elegans* (SRR10028111) and *D. melanogaster* (SRR13070625) were retrieved from the NCBI Sequence Read Archive (SRA). PacBio HiFi sequencing data for the HG002 sample was downloaded from the Genome in a Bottle (GIAB) project [40]. CycloneSEQ G400-ER sequencing data for HG002 was generated in-house using genomic DNA extracted from the HG002 lymphoid cell line following standard library preparation and sequencing protocols.

Three genomic regions from the human genome were used for selecting appropriate methods for feature extraction, DR and k-NN search, including the *HLA* immunogene cluster, the *IGK* immunoglobulin $\kappa$-light chain locus, and chromosome 22 (Supplementary Table S1). For each region, real sequencing data were mapped to the corresponding reference genome using minimap2 with the following parameters: -k 19 -w 5 -A 3 -B 2 -m 250 –secondary=no, and reads belonging to the given region were extracted. Whole-genome sequencing data for *C. elegans* (ONT R10), *D. melanogaster* (ONT R10), and *H. sapiens* (ONT R10, PacBio HiFi, and CycloneSEQ G400-ER) were used for benchmarking overlap detection tools.

### Overlap graph construction and evaluation

For a dataset of *n* sequencing reads, an undirected overlap graph $G = (V, E)$ was constructed from pairs of reads identified as overlapping. Each vertex in the graph represented an oriented read—either in its forward or reverse-complement orientation—resulting in a total of $2n$ vertices. An edge $\lbrace u, v\rbrace$ was added to the graph if read *u* was among the *k* nearest neighbors of read *v*, or vice versa, indicating that the two reads likely originated from overlapping genomic regions. The total number of edges in the graph ranged from $kn$ to $2kn$, depending on the degree of mutuality among nearest-neighbor relationships (i.e., whether overlaps were reciprocal or one-sided).

For each benchmarking dataset, overlap graphs constructed using nearest neighbors identified by a specific method were evaluated against a reference graph $G^{\prime } = (V, E^{\prime })$ built from the same dataset. The reference graph shared the same set of vertices *V* as the overlap graphs. For real sequencing data, the reference edges $E^{\prime }$ were defined based on the alignment positions of reads in the reference genome. Sequencing reads were filtered based on the following criteria to remove ambiguously aligned reads: (1) read length $\ge$ 5 kb; (2) aligned fraction $\ge$ 50%; (3) mapping quality $\ge$ 30. For simulated data, the edges $E^{\prime }$ were determined according to the genomic intervals from which each read was simulated, as reported by PBSIM3.

For each edge $\lbrace u, v\rbrace$ identified in overlap graph $G = (V, E)$, if the same edge exist in the reference graph $G^{\prime }$, this edge was considered correct. Otherwise, this edge was considered incorrect. We used four metrics to quantitatively evaluate the quality of an overlap graph: error rate, mean overlap size, the number of correct overlap candidates (#COC) and the number of connected components (#CC). The error rate was defined as the number of incorrect edges divided by the number of total edges. Mean overlap size was defined as the arithmetic mean of overlap size of all edges. The overlap size of incorrect edges was considered zero for this calculation. #COC was defined as the degree of each node after removing any incorrect edges. If a node does not have any correct edges, it is referred to as a singleton. #CC was defined as the number of connected components of the overlap graph after removing any incorrect edges.

### Benchmarking overlap detection tools

We benchmarked Fedrann (v0.5.4) against six state-of-the-art tools: minimap2 (v2.24), xRead (v1.0.0), BLEND (v1.0.0), MHAP (v2.1.1), MECAT2 (v20190314), and wtdbg2 (v2.5). All alignments were filtered using two criteria: (1) $\ge$ 100 matched bases and (2) $\ge$ 10% alignment identity. Notably, for minimap2 PAF files (which produced exceptionally large outputs exceeding 4TB for human whole-genome sequencing read alignments), we increased the identity threshold to 30% to reduce computational overhead. All overlap detection tools were benchmarked using 64 threads on a dual-socket Linux server equipped with AMD EPYC 9654 processors (192 physical cores/384 threads total, 2.4 GHz base clock) and 2 TB of DDR5 RAM. Refer to Supplementary Tables S4 and S5 for the specific parameters used for each tool.

### Whole genome assembly and evaluation

To evaluate the practical utility of our approach in large-scale genomic workflows, we performed whole-genome assembly benchmarks on the H4C and H4O datasets, comparing the performance of the integrated Fedrann-Shasta pipeline against the standalone Shasta assembler. For the Fedrann-Shasta implementation, we utilized Fedrann (v0.5.4) and specified the number of nearest neighbors (*k*) for each read as $k=30$ for the H4C dataset and $k=50$ for the H4O dataset. In both the integrated pipeline and standalone runs, we employed Shasta using the –config Nanopore-May2022 parameter to ensure a consistent assembly parameters. The resulting assemblies were rigorously evaluated across multiple dimensions: QUAST v5.3.0 [41] was used to calculate NGA50 values for structural contiguity, while yak v0.1-r69-dirty [42] was utilized to estimate *k-*mer based Consensus Quality (QV) and *k-*mer completeness for base-level accuracy. Biological completeness was assessed using BUSCO v5.8.0 [43] against the primates_odb10 lineage database. Dot plots were generated using the Lakeview library [44].

## Availability of source code and requirements

- Project name: Fedrann
- Project homepage: https://github.com/jzhang-dev/FEDRANN
- License: GPL-3.0
- Operating system(s): Linux (Ubuntu 20.04 or later)
- Package management: Docker
- Programming language: Python, C++
- Hardware requirements: CPU with AVX2 support; minimum 32GB RAM (256GB+ recommended for human genome datasets)
- RRID: SCR_027416
- Integrated pipeline: Fedrann–Shasta pipeline: https://github.com/jzhang-dev/Fedrann-Shasta
- Reproducible workflows for benchmarking and evaluation: https://github.com/jzhang-dev/kNN-overlap-finder

## Supplementary Material

giag048_Supplemental_File

giag048_Authors_Response_To_Reviewer_Comments_original_submission

giag048_GIGA-D-25-00332_original_submission

giag048_GIGA-D-25-00332_revision_1

giag048_Reviewer_1_Report_original_submission Reviewer 1 -- 10/10/2025

giag048_Reviewer_2_Report_original_submission Reviewer 2 -- 11/24/2025

giag048_Reviewer_2_Report_revision_1 Reviewer 2 -- 2/12/2026

## Data Availability

The CycloneSEQ G400-ER sequencing data generated in this study is available via the publicly available database CNGBdb (accession number CNX1236558). These data have also been deposited in the National Center for Biotechnology Information (NCBI) databases. The BioProject accession number is PRJNA1321476. The raw sequence reads are available in the SRA under the accession number SRR35291479. The associated BioSample accession is SAMN51209978. The simulated datasets and numerical data underlying the figures and supplementary materials are available in the GigaScience GigaDB database [45].

## Additional files

**Supplementary Fig. S1**: Error rate of preprocessing methods and distance metrics for overlap detection. Overlap detection error rate of various preprocessing methods combined with Euclidean or cosine distance in various datasets. Each dot represents top-k neighbors error rate. No dimensionality reduction or ANN methods were used in k-NN search. Each dot represents the overlap detection error rate for a graph constructed using top-k nearest neighbors.

**Supplementary Fig. S2**: Error rate distribution of preprocessing methods and distance metrics for overlap dectection. Overlap detection error rate distribution in overlap detection across datasets (H1C-H3C, H1O-H3O, H1P-H3P) using different text preprocessing methods (TF, IDF, TF-IDF, Raw TF, None) with cosine and Euclidean distance metrics. No dimensionality reduction or ANN methods were used in k-NN search. Boxplots show quartile ranges with whiskers indicating 1.5×IQR, and circles denote outliers.

**Supplementary Fig. S3**: Assessment of simulated sequence reads. (a, b) Heatmap showing overlap detection error rates for simulated sequencing reads from the human IGK region (a) and chromosome 22 (b). Each cell representing error rates under different combinations of sequencing depth (10×, 30×, 50×), accuracy (91%–99%), and read length (10–30 kb). IDF preprocessing was used in feature extraction. Exact cosine distance was used in k-NN search. Top six neighbors according to each distance metric were used to construct overlap graphs for evaluation. The heatmap used a red-to-green color gradient, where red indicates higher error rates and green indicates lower error rates.

**Supplementary Fig. S4**: Assessment of embedding dimensions. Overlap detection error rate of various embedding dimensions. IDF preprocessing was used in feature extraction. Cosine distance was used as metric in k-NN search. Feature matrices were reduced to different dimensions using SRP prior to ENN search. Top six neighbors were used to construct overlap graphs for evaluation.

**Supplementary Fig. S5**: Assessment of embedding dimensions (Detail). Overlap detection error rate of various embedding dimensions. IDF preprocessing was used in feature extraction. Cosine distance was used as metric in k-NN search. Feature matrices were reduced to different dimensions using SRP prior to ENN search. Each dot represents the overlap detection error rate for a graph constructed using top-k nearest neighbors.

**Supplementary Fig. S6**: Effect of embedding dimension to distance preservation. (a) Correlation between distance metrics before/after dimensionality reduction at three embedding dimensions (100, 500, 3000) for dataset H3P. (b) Correlation between cosine distance (after dimensionality reduction) and overlap size at three embedding dimensions (100, 500, 3000) for dataset H3P.

**Supplementary Fig. S7**: Schematic overview of the Fedrann workflow This flowchart summarizes the main stages of the Fedrann pipeline. The pipeline comprises four operational steps implementing three core algorithmic components: feature extraction (steps 1–3), dimensionality reduction (precomputed in step 2 and applied in step 3), and ANN search (step 4). Fedrann employs ICF as a practical approximation to IDF, leveraging Jellyfish for efficient *k* counting.

**Supplementary Fig. S8**: Correlation between *k* collection frequency and document frequency. The relationship between the $\log _{10}$-transformed collection frequency and document frequency of *K*-mers in H4C dataset. The red line represents the linear regression fit.

**Supplementary Fig. S9**: Precision-recall analysis of various overlap detection tools on the H3C dataset. (a, b) Recall (a) and precision (b) of various overlap detection tools plotted against the number of overlap candidates (*k*). Recall values are calculated as the mean per-read recall across all reads. (c) Distribution of the number of overlapping reads (neighbor count) in the H3C dataset. In (a)–(c), the vertical dashed gray line represents the mode of the neighbor count across all reads in the dataset.

**Supplementary Fig. S10**: Recall of Fedrann stratified by overlap length on the H3C dataset. Recall of Fedrann plotted against the number of nearest neighbors (*k*) for various overlap length intervals. Each colored curve represents the recall rate achieved for a specific range of overlap lengths.

**Supplementary Fig. S11**: *De novo* assembly based on the Fedrann-Shasta pipeline (a) Alignment of Fedrann-Shasta assembly to the HG002 reference genome. Darker colors represent longer contigs. (b) Cumulative fraction of bases (*y*-axis) plotted against each minimum contig length threshold (*x*-axis) for the Fedrann-Shasta assembly.

**Supplementary Fig. S12**: Synteny and collinearity analysis of the HLA region assembly. (a, b) Dot plots showing the collinearity of the Fedrann-Shasta (a) and standard Shasta (b) assemblies (*y*-axis) against the T2T-CHM13 reference genome (*x*-axis). The horizontal dashed gray lines indicate the boundaries between two adjacent contigs. (c, d) Magnified views of the regions in (a) and (b), respectively, highlighting a complex segmental duplication locus (chr6: 31.75–32.05 Mb).

**Supplementary Fig. S13**: Impact of various parameters to Fedrann performance. (a) Evaluation of computational efficiency parameters. The plots illustrate the impact of *K* frequency threshold, *K* sample fraction, embedding dimension and number of trees on precision (line plots), wall-clock time (blue bars), and peak memory (red bars). (b) Performance across *k* sizes for different sequencing platforms. The benchmarking was conducted on genome-wide datasets from CycloneSEQ (H4C), ONT (H4O), and PacBio (H4P) platforms.

**Supplementary Table S1**: Summary of real datasets used in this study.

**Supplementary Table S2**: Summary of dimensionality reduction methods used in this study. Custom Python implementation of SimHash was used as no existing libraries suitable for analyzing biological sequences were found.

**Supplementary Table S3**: Summary of k-nearest neighbor search methods used in this study.

**Supplementary Table S4**: Fedrann parameters used for benchmarking.

**Supplementary Table S5**: Command-line arguments of various tools used for benchmarking.

**Supplementary Table S6**: *De novo* assembly statistics of Fedrann-Shasta and Shasta.

**Supplementary Table S7**: Performance of Fedrann under various CPU and memory limitations. Benchmarks were conducted on dataset H4C within Docker containers to simulate hardware constraints. RAM: random access memory. Prec.: precision. HDD: hard disk drive. SSD: solid state drive.
