# Supplementary material for: FEDRANN: effective long-read overlap detection based on dimensionality reduction and approximate nearest neighbors
Source: Gigascience. 2026 May 8;15:giag048. doi: 10.1093/gigascience/giag048 (PMC13201080; doi:10.1093/gigascience/giag048)
Supplement: giag048_Supplemental_File [file giag048_supplemental_file.pdf]

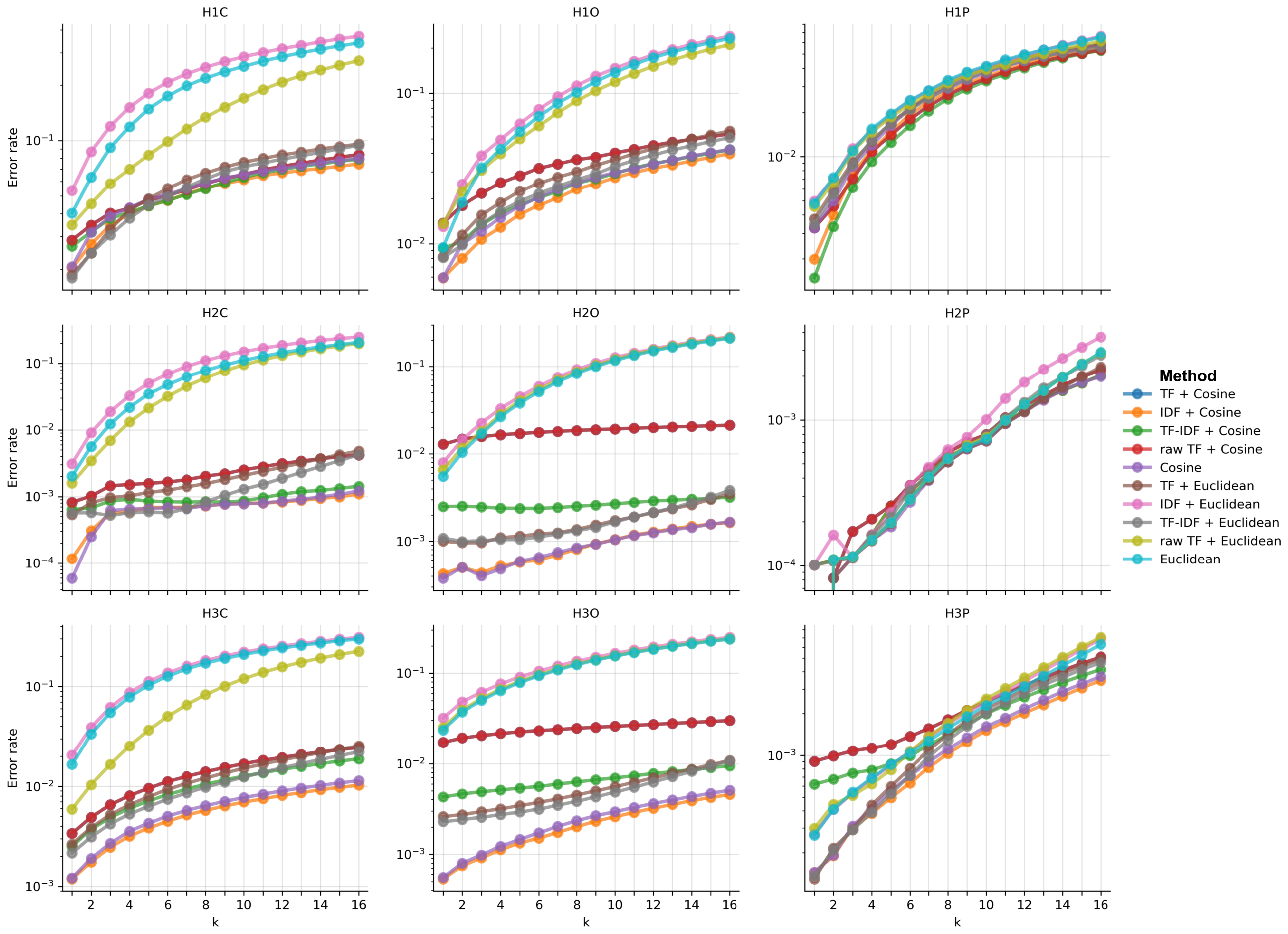

Figure S1. Error rate of preprocessing methods and distance metrics for overlap detection.
Overlap detection error rate of various preprocessing methods combined with Euclidean or cosine distance in various datasets. Each dot represents top-k neighbors error rate. No dimensionality reduction or ANN methods were used in k-NN search. Each dot represents the overlap detection error rate for a graph constructed using top-k nearest neighbors.

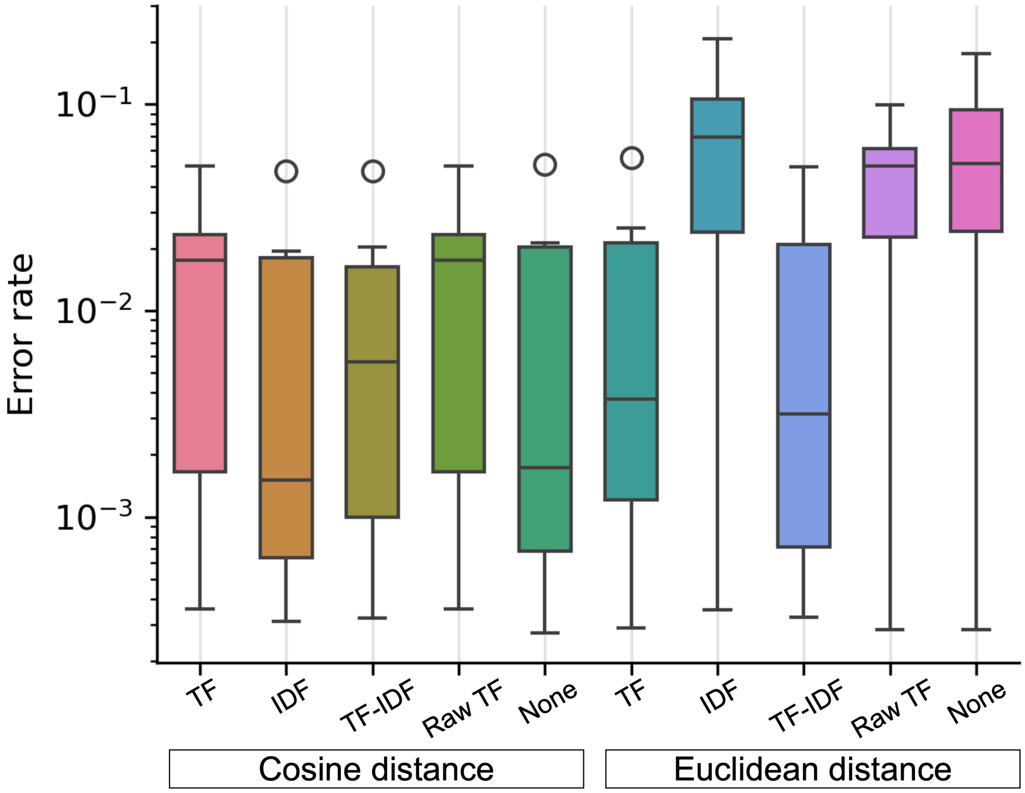

Figure S2. Error rate distribution of preprocessing methods and distance metrics for overlap dectection.
Overlap detection error rate distribution in overlap detection across datasets (H1C-H3C, H1O-H3O, H1P-H3P) using different text preprocessing methods (TF, IDF, TF-IDF, Raw TF, None) with cosine and Euclidean distance metrics. No dimensionality reduction or ANN methods were used in k-NN search. Boxplots show quartile ranges with whiskers indicating 1.5×IQR, and circles denote outliers.

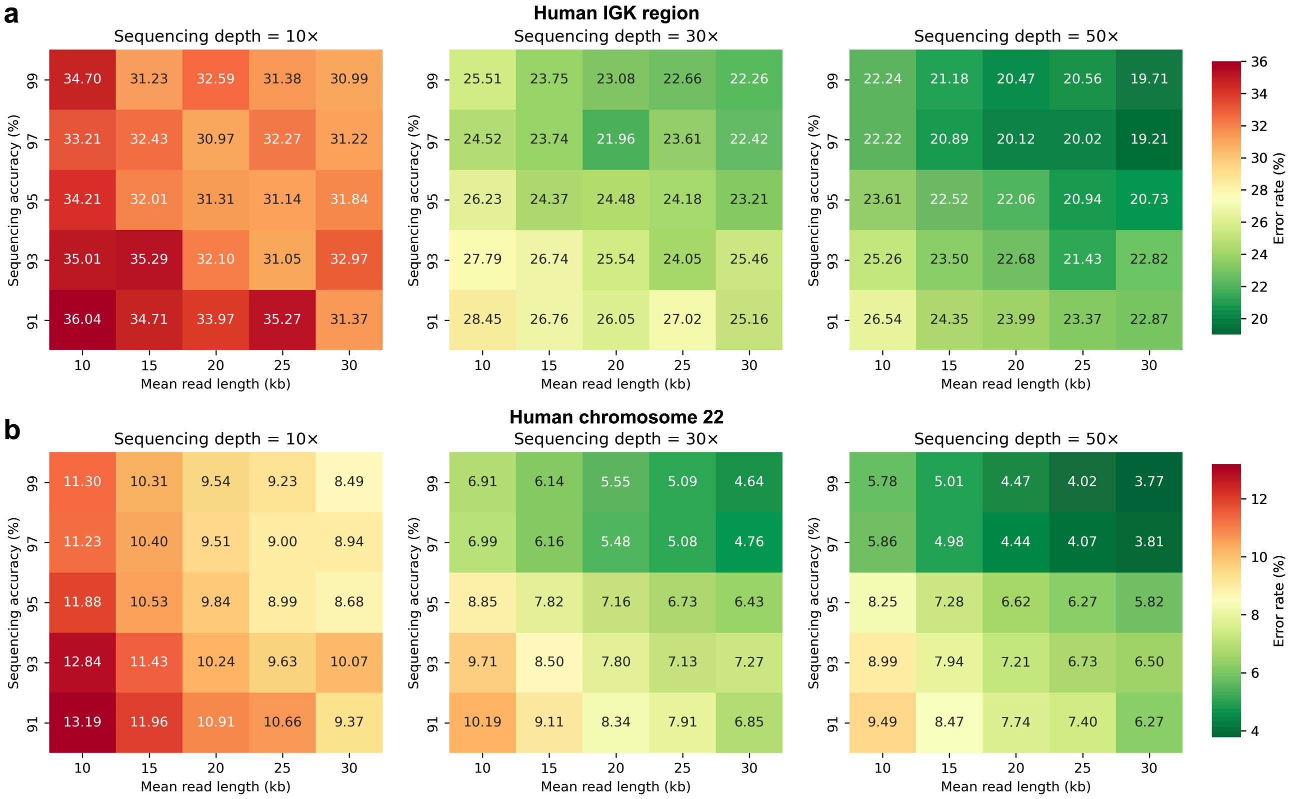

Figure S3. Assessment of simulated sequence reads.

(a-b) Heatmap showing overlap detection error rates for simulated sequencing reads from the human IGK region **(a)** and chromosome 22 **(b)**. Each cell representing error rates under different combinations of sequencing depth (10×, 30×, 50×), accuracy (91%–99%), and read length (10–30 kb). IDF preprocessing was used in feature extraction. Exact cosine distance was used in k-NN search. Top six neighbors according to each distance metric were used to construct overlap graphs for evaluation. The heatmap used a red-to-green color gradient, where red indicates higher error rates and green indicates lower error rates.

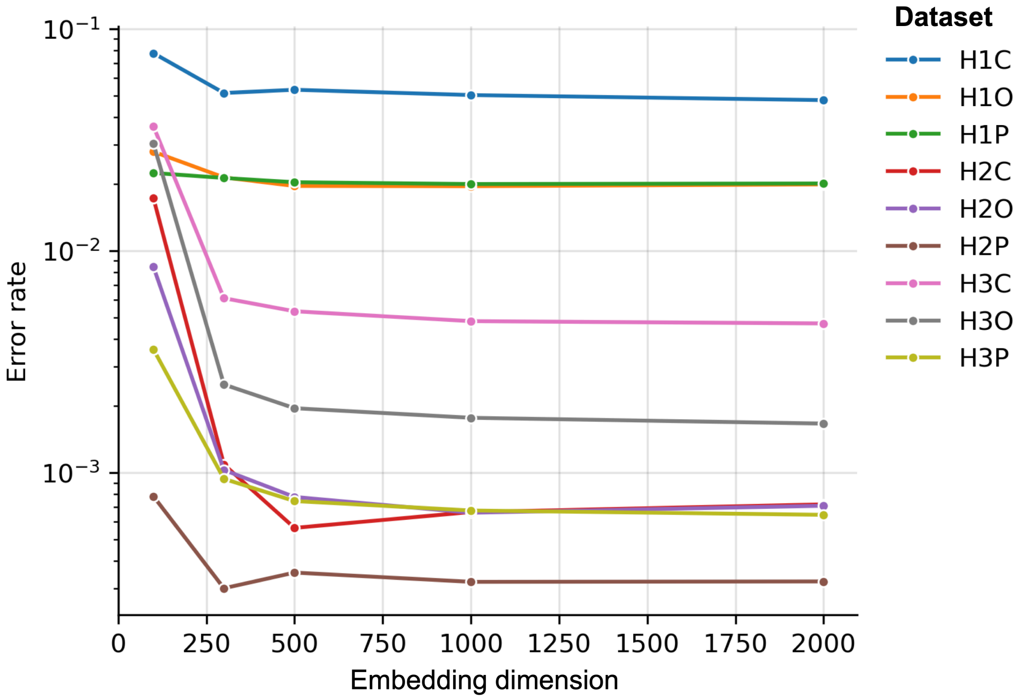

Figure S4. Assessment of embedding dimensions.
Overlap detection error rate of various embedding dimensions. IDF preprocessing was used in feature extraction. Cosine distance was used as metric in k-NN search. Feature matrices were reduced to different dimensions using Sparse Random Projection prior to ENN search. Top six neighbors were used to construct overlap graphs for evaluation.

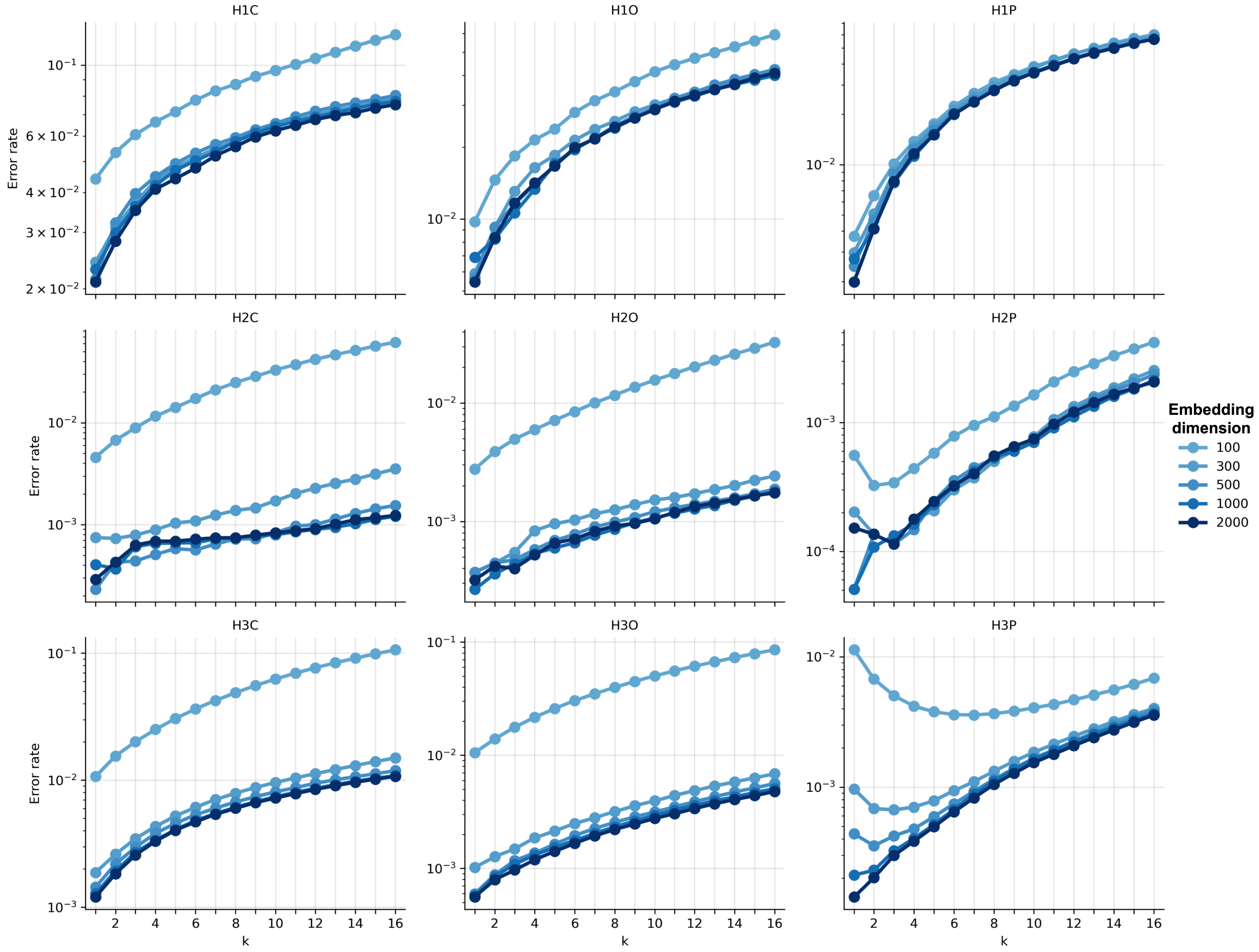

Figure S5. Assessment of embedding dimensions (Detail).
Overlap detection error rate of various embedding dimensions. IDF preprocessing was used in feature extraction. Cosine distance was used as metric in k-NN search. Feature matrices were reduced to different dimensions using Sparse Random Projection prior to ENN search. Each dot represents the overlap detection error rate for a graph constructed using top-k nearest neighbors.

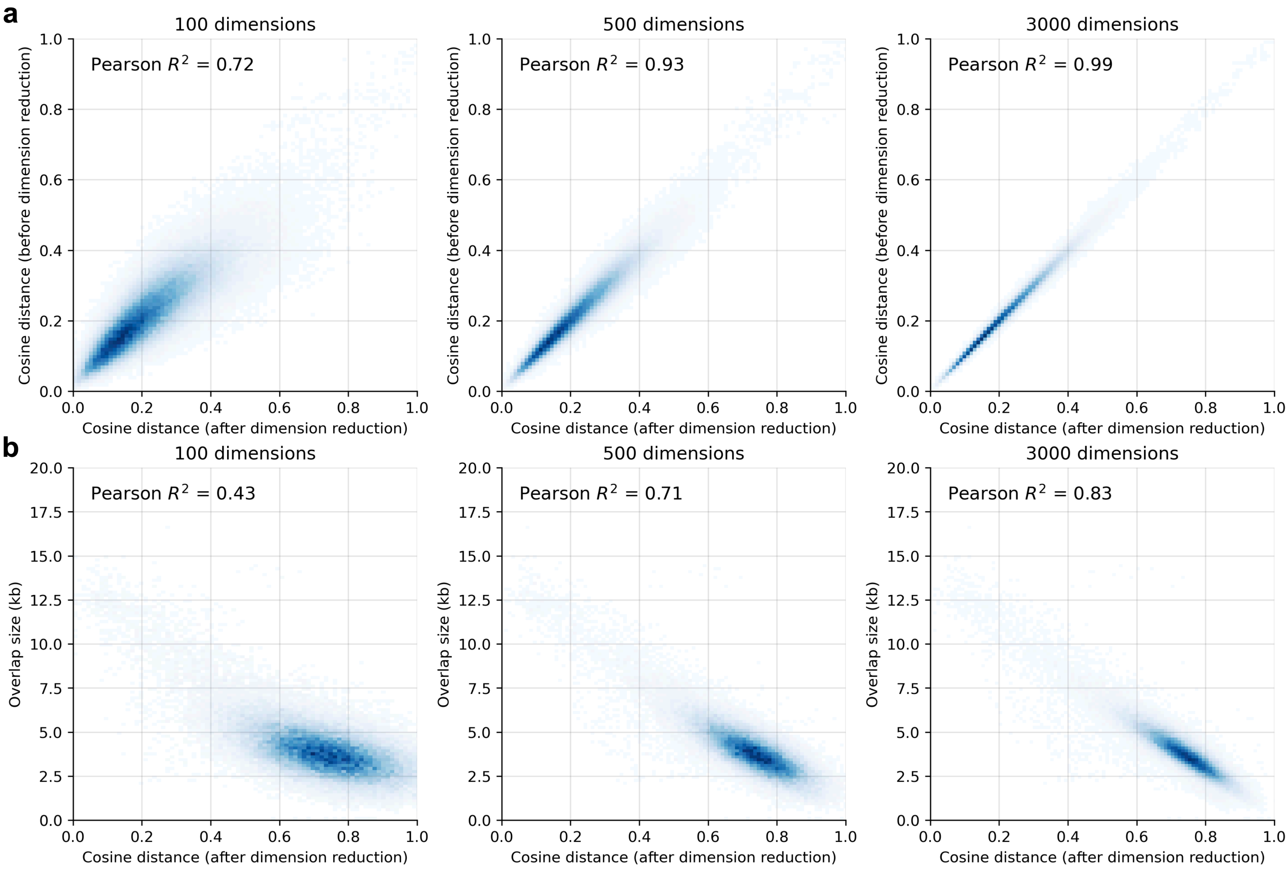

Figure S6. Effect of embedding dimension to distance preservation.
(a) Correlation between distance metrics before/after dimensionality reduction at three embedding dimensions (100,500,3000) for dataset H3P. **(b)** Correlation between cosine distance (after dimensionality reduction) and overlap size at three embedding dimensions (100,500,3000) for dataset H3P.

```
                    INPUT
                      |
                      v
            +-----------------------------------+
            |  FASTQ/FASTA Sequencing Reads     |
            |  (e.g., reads.fasta.gz)           |
            +-----------------------------------+
                      |
                      v
==============================================================
|           STEP 1: K-mer Counting and Sampling              |
==============================================================
                      |
            +-----------------+------------------+
            |                                    |
            v                                    v
+----------------------+           +-------------------------+
|  Jellyfish Count     |           |  Sample k-mers          |
|  - Count all k-mers  |  -------> |  - Sample fraction      |
|  - Filter by min     |           |  - Generate library     |
|    multiplicity      |           |  - Forward + Reverse    |
+----------------------+           +-------------------------+
                                                |
                                                v
                                  +-------------------------+
                                  |  kmer_searcher          |
                                  |  - Find k-mer positions |
                                  |  - Build sparse vectors |
                                  +-------------------------+
                                                |
                                                v
==============================================================
|   STEP 2: Dimensionality Reduction Matrix Precomputation   |
==============================================================
                      |
            +-----------------+------------------+
            |                                    |
            v                                    v
+----------------------+           +-------------------------+
|  Compute IDF Weights |           |  Generate Random        |
|  - Calculate inverse |           |  Projection Matrix      |
|    document freq     |  -------> |  - Sparse Random        |
|  - Weight k-mers by  |           |    Projection (SRP)     |
|    informativeness   |           |  - n_components × n_feat|
+----------------------+           +-------------------------+
                                                |
                                                v
                                  +-------------------------+
                                  |  Precompute Matrix      |
                                  |  (SRP_transpose × ICF)  |
                                  +-------------------------+
                                                |
                                                v
==============================================================
|           STEP 3: Feature Matrix Generation                |
==============================================================
                      |
            +-----------------+------------------+
            |                                    |
            v                                    v
+----------------------+           +-------------------------+
|  Parse k-mer Indices |           |  Apply Precompute       |
|  - Read binary file  |  -------> |  Matrix                 |
|  - Build sparse row  |           |  - Matrix multiplication|
|  - Both strands      |           |  - Get embeddings       |
+----------------------+           +-------------------------+
                                                |
                                                v
                                  +-------------------------+
                                  |  Embedding Matrix       |
                                  |  (n_reads × n_dim)      |
                                  |  [Optional: Save]       |
                                  +-------------------------+
                                                |
                                                v
==============================================================
|      STEP 4: Approximate Nearest Neighbor Search           |
==============================================================
                  |
                  v
        +-----------------------------+
        |  NNDescent Algorithm        |
        |  - Build k-NN graph index   |
        |  - Graph-based search       |
        |  - Cosine distance metric   |
        +-----------------------------+
                  |
                  v
        +-----------------------------+
        |  Neighbor Indices &         |
        |  Distance Matrix            |
        +-----------------------------+
                  |
                  v
==============================================================
|                       OUTPUT FILES                         |
==============================================================
                             |
     +-----------------------+-----------------------+
     |                                               |
     v                                               v
+----------------------+                +----------------------+
| overlaps.tsv         |                | feature_matrix.npz   |
|                      |                | (optional)           |
| - query_name         |                |                      |
| - query_orientation  |                | - Embedding vectors  |
| - target_name        |                | - Sparse format      |
| - target_orientation |                |                      |
| - neighbor_rank      |                +----------------------+
| - distance           |
+----------------------+
```

Figure S7. Schematic overview of the Fedrann workflow

This flowchart summarizes the main stages of the Fedrann pipeline. The pipeline comprises four operational steps implementing three core algorithmic components: feature extraction (steps 1–3), dimensionality reduction (precomputed in step 2 and applied in step 3), and approximate nearest neighbor (ANN) search (step 4). Fedrann employs inverse collection frequency (ICF) as a practical approximation to inverse document frequency (IDF), leveraging Jellyfish for efficient k-mer counting.

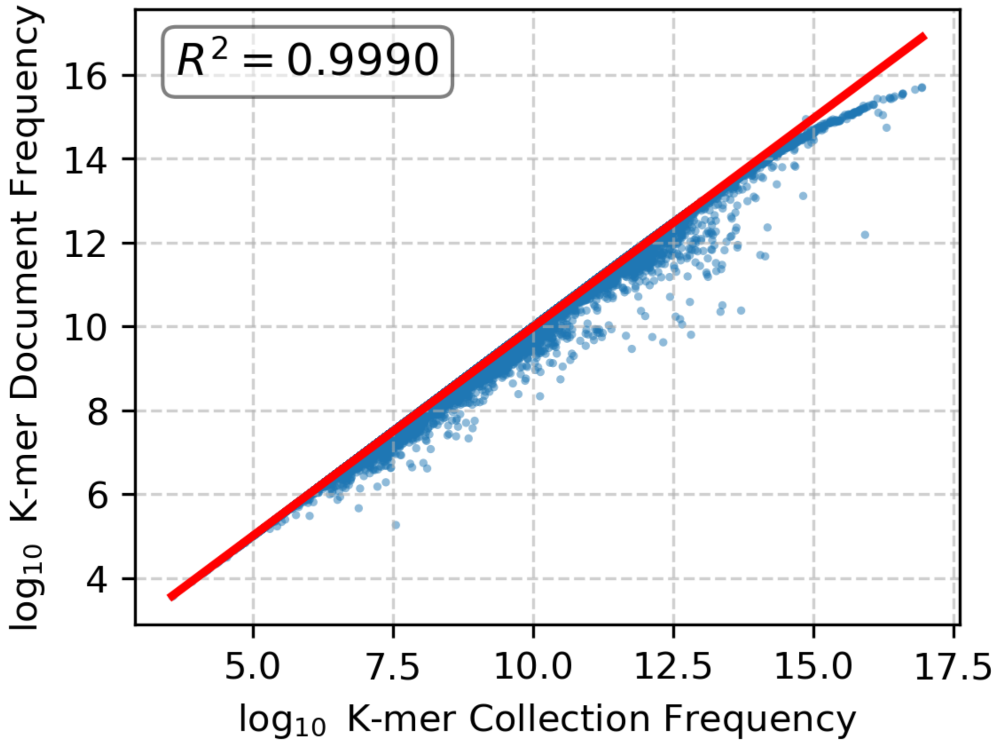

Figure S8. Correlation between k-mer collection frequency and document frequency.
The relationship between the $\log_{10}$-transformed collection frequency and document frequency of K-mers in H4C dataset. The red line represents the linear regression fit.

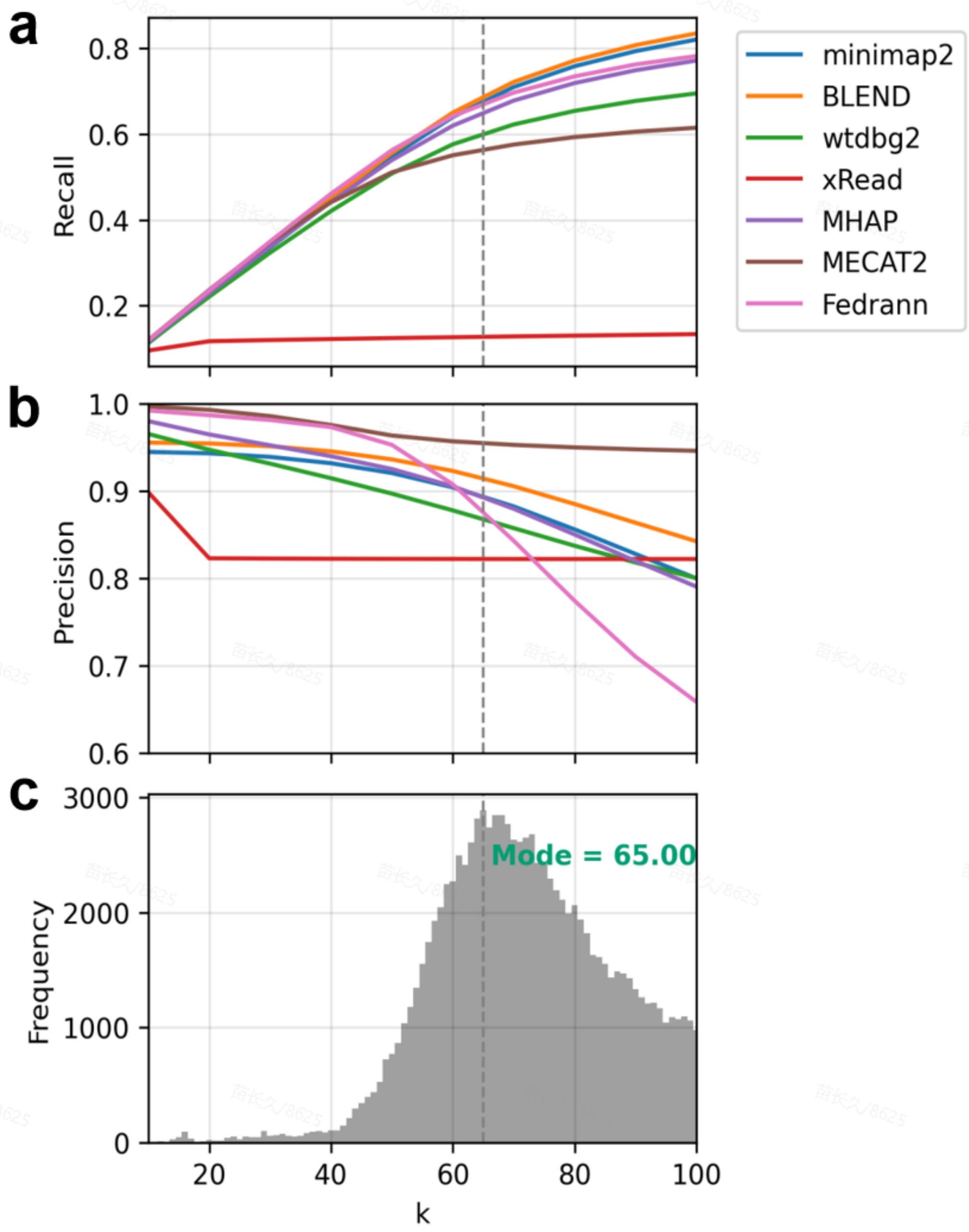

Figure S9. Precision-recall analysis of various overlap detection tools on the H3C dataset.
(a-b) Recall (a) and precision (b) of various overlap detection tools plotted against the number of overlap candidates (k). Recall values are calculated as the mean per-read recall across all reads. (c) Distribution of the number of overlapping reads (neighbor count) in the H3C dataset. In (a)-(c), the vertical dashed gray line represents the mode of the neighbor count across all reads in the dataset.

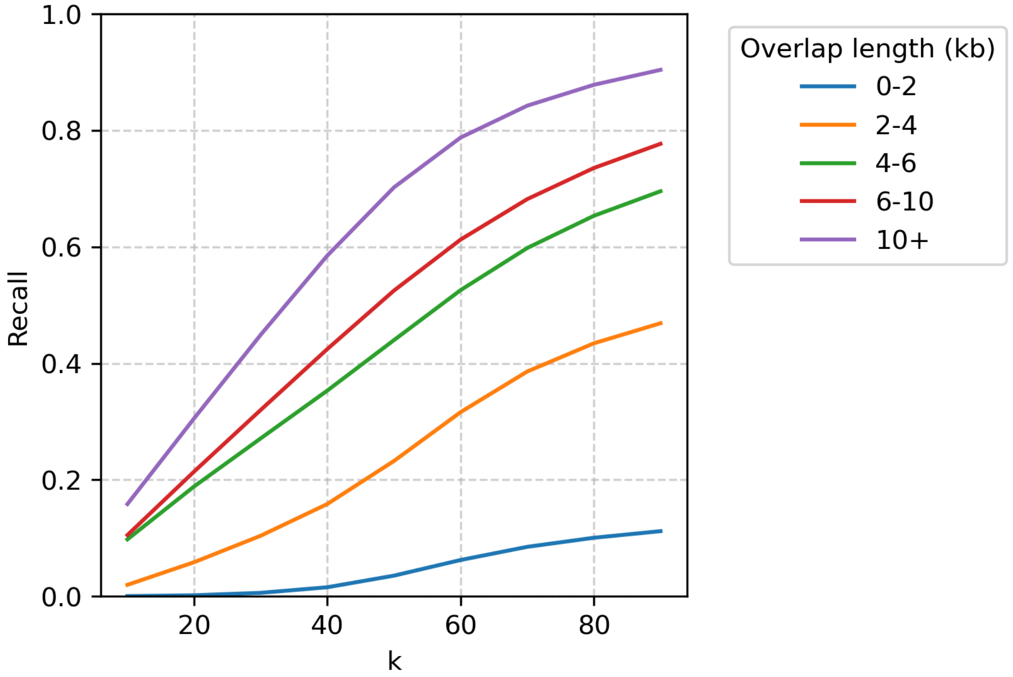

Figure S10. Recall of Fedrann stratified by overlap length on the H3C dataset.
Recall of Fedrann plotted against the number of nearest neighbors (k) for various overlap length intervals. Each colored curve represents the recall rate achieved for a specific range of overlap lengths.

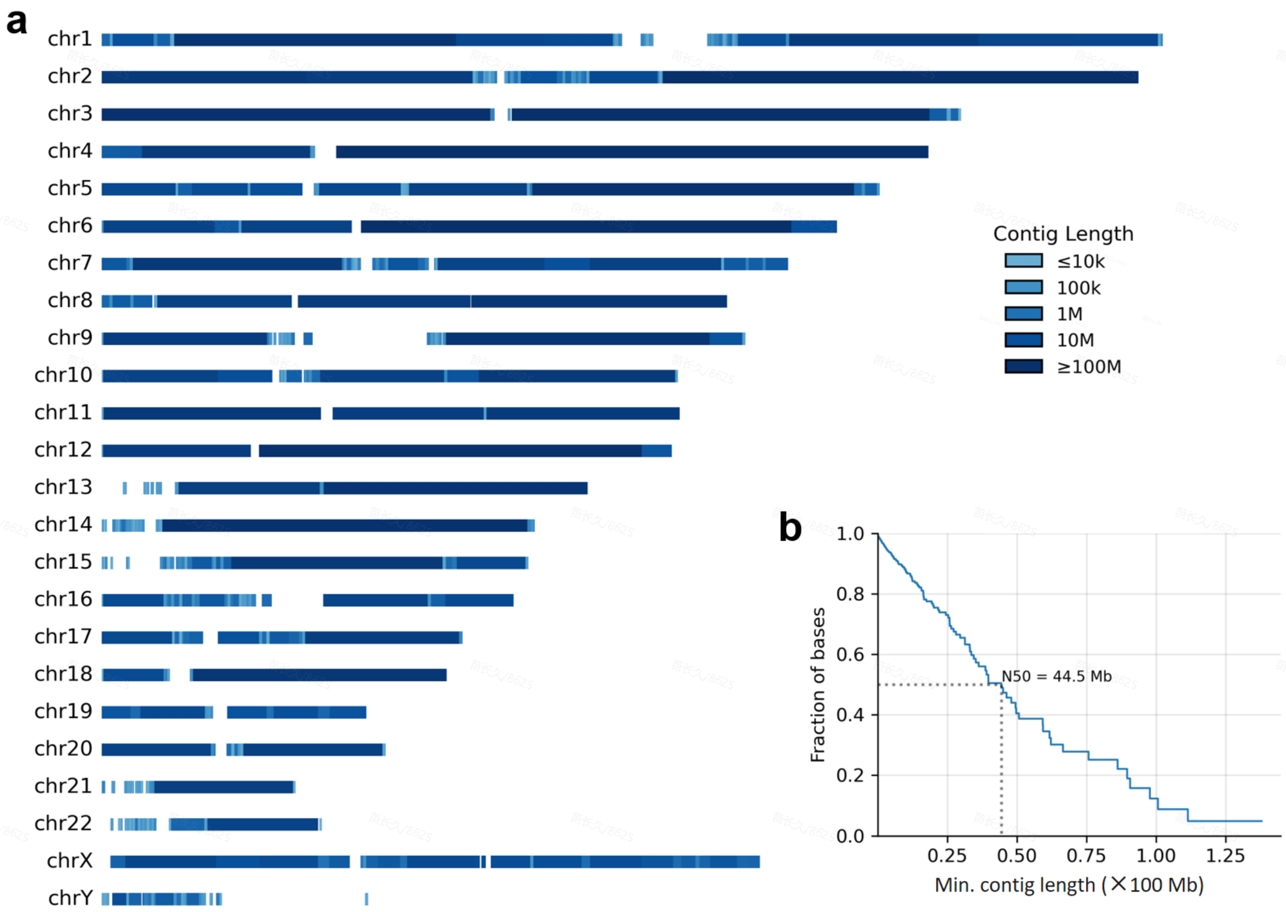

Figure S11. *De novo* assembly based on the Fedrann-Shasta pipeline
(a) Alignment of Fedrann-Shasta assembly to the HG002 reference genome. Darker colors represent longer contigs. **(b)** Cumulative fraction of bases (y axis) plotted against each minimum contig length threshold (x axis) for the Fedrann-Shasta assembly.

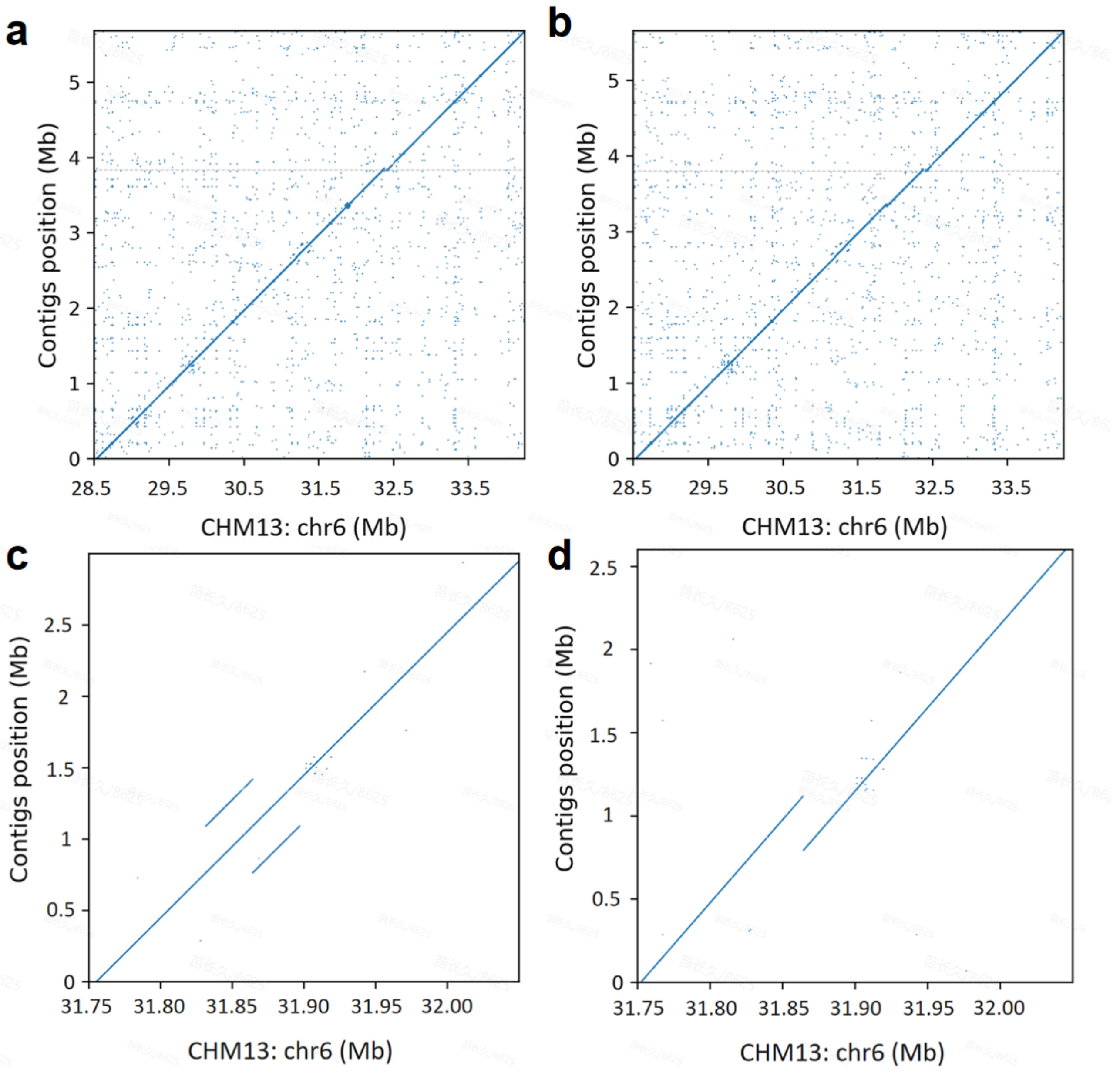

Figure S12. Synteny and collinearity analysis of the HLA region assembly.
(a-b) Dot plots showing the collinearity of the Fedrann-Shasta (a) and standard Shasta (b) assemblies (y-axis) against the T2T-CHM13 reference genome (x-axis). The horizontal dashed grey lines indicate the boundaries between two adjacent contigs. **(c-d)** Magnified views of the regions in (a) and (b), respectively, highlighting a complex segmental duplication locus (chr6: 31.75–32.05 Mb).

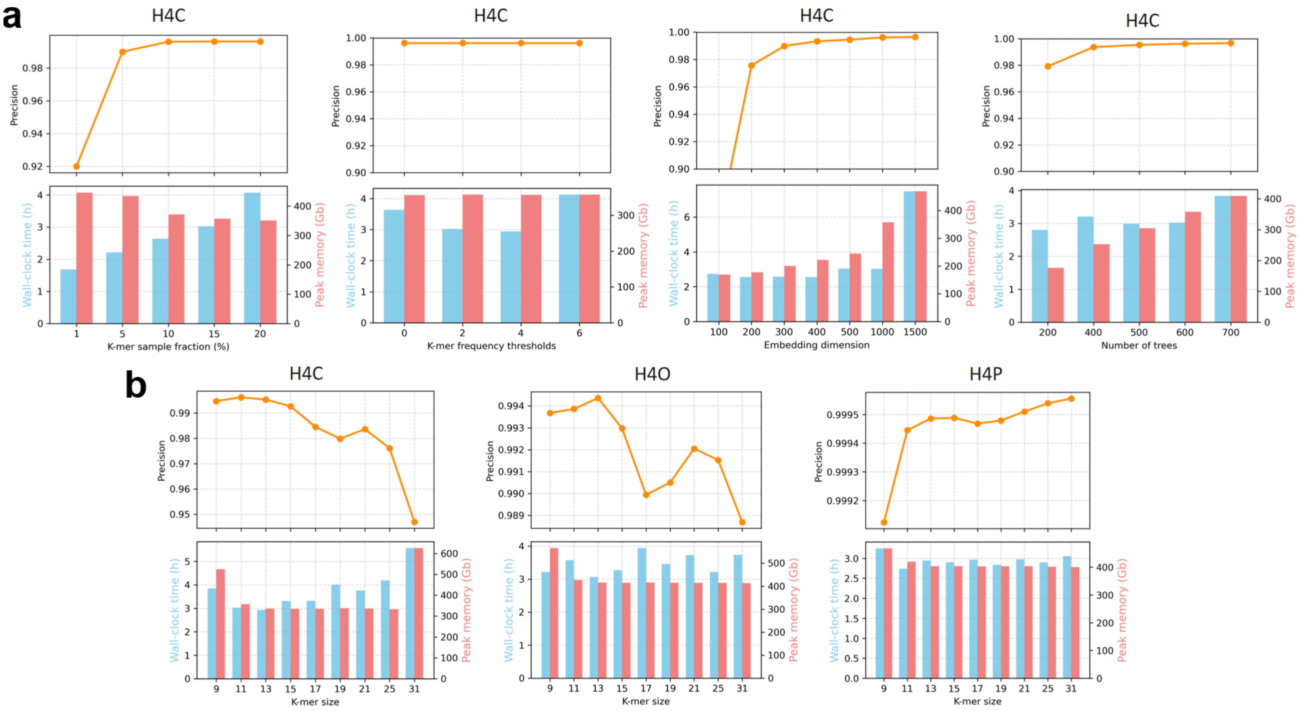

Figure S13. Impact of various parameters to Fedrann performance.
(a) Evaluation of computational efficiency parameters. The plots illustrate the impact of K-mer frequency threshold, K-mer sample fraction, embedding dimension and number of trees on precision (line plots), wall-clock time (blue bars), and peak memory (red bars). (b) Performance across k-mer sizes for different sequencing platforms. The benchmarking was conducted on genome-wide datasets from CycloneSEQ (H4C), ONT (H4O), and PacBio (H4P) platforms.

Supplementary tables

Table S1. Summary of real datasets used in this study.

Dataset	Sample	Region	Platform	Reference size (Mb)	Read count	N50 (kb)
H1C	*H. sapiens* (HG002)	IGK	CycloneSEQ G400-ER	3.92	2,506	41.91
H1O	*H. sapiens* (HG002)	IGK	ONT R10	3.92	2,701	22.97
H1P	*H. sapiens* (HG002)	IGK	PacBio HiFi	3.92	2,624	13.48
H2C	*H. sapiens* (HG002)	HLA	CycloneSEQ G400-ER	5.75	11,176	40.67
H2O	*H. sapiens* (HG002)	HLA	ONT R10	5.75	12,576	23.16
H2P	*H. sapiens* (HG002)	HLA	PacBio HiFi	5.75	12,872	13.42
H3C	*H. sapiens* (HG002)	Chromosome 22	CycloneSEQ G400-ER	51.32	76,601	41.25
H3O	*H. sapiens* (HG002)	Chromosome 22	ONT R10	51.32	81,891	22.78
H3P	*H. sapiens* (HG002)	Chromosome 22	PacBio HiFi	51.32	85,993	13.43
H4C	*H. sapiens* (HG002)	whole genome	CycloneSEQ G400-ER	3,117.29	5,127,109	40.67
H4O	*H. sapiens* (HG002)	whole genome	ONT R10	3,117.29	6,226,490	23.06
H4P	*H. sapiens* (HG002)	whole genome	PacBio HiFi	3,117.29	6,061,187	13.48
C1O	*C. elegans* (strain N2)	whole genome	ONT R9	100.29	221,513	19.02
D1O	*D. melanogaster* (BDGP genome strain)	whole genome	ONT R9	143.73	281,881	23.92

Table S2. Summary of dimensionality reduction methods used in this study. Custom Python implementation of SimHash was used as no existing libraries suitable for analyzing biological sequences were found.

Method	Implementation	Version
scBiMapping	`scBiMapping`	v0.1.0
UMAP	`umap-learn`	v0.5.3
PCA	`sklearn.decomposition.PCA`	v1.2.2
Gaussian Random Projection	`sklearn.random_projection.GaussianRandomProjection`	v1.2.2
Sparse Random Projection	`sklearn.random_projection.SparseRandomProjection`	v1.2.2
Spectral Embedding	`snapatac2.tl.spectral`	v2.1.0
SimHash	Custom Python implementation	–

Table S3. Summary of k-nearest neighbor search methods used in this study.

Method	Implementation	Version	Parameter
brute-force search	`sklearn.neighbors.NearestNeighbors`	v1.2.2	n_jobs = 64
HNSW	`hnswlib`	v0.7.0	M = 512, ef_construction = 200, set_ef = 50, set_num_threads = 64
PQ	`faiss`	v1.8.0	omp_set_num_threads = 64, index param = "PQ128x8"
IVF-PQ	`faiss`	v1.8.0	omp_set_num_threads = 64, M = 128, nlist = 1024, nbits_per_idx = 8, nprobe = 300
RPF	`rpforest`	v1.6	leaf_size = 50, no_trees = 100
NNDescent	`pynndescent`	v0.5.12	n_jobs = 64, n_trees = 600, leaf_size = 200, index_n_neighbors = 50

Table S4. Fedrann parameters used for benchmarking.

Dataset	Feature extraction	Dimensionality reduction	ANN search
H4C	k-mer size = 11; k-mer sample fraction = 0.15	SRP (embedding dimension = 1000)	NNDescent (number of trees = 600)
H4O	k-mer size = 13; k-mer sample fraction = 0.15	SRP (embedding dimension = 1000)	NNDescent (number of trees = 600)
H4P	k-mer size = 31; k-mer sample fraction = 0.15	SRP (embedding dimension = 1000)	NNDescent (number of trees = 600)
C1O	k-mer size = 21; k-mer sample fraction = 0.10	SRP (embedding dimension = 1000)	NNDescent (number of trees = 600)
D1O	k-mer size = 21; k-mer sample fraction = 0.15	SRP (embedding dimension = 2000)	NNDescent (number of trees = 600)

Table S5. Command-line arguments of various tools used for benchmarking.

Tool	Dataset platform	Arguments
xRead	CycloneSEQ/ONT	-k 19 -w 40 -p 2 -t 64
xRead	PacBio	-k 19 -w 40 -p 2 -t 64
MECAT2	CycloneSEQ/ONT/PacBio	-t 64
BLEND	CycloneSEQ/ONT	-x ava-ont -t 64
BLEND	PacBio	-x ava-hifi -t 64
wtdbg2	CycloneSEQ/ONT	-p 0 -k 15 -AS 2 -s 0.05 -t 64
wtdbg2	PacBio	-p 21 -k 0 -AS 4 -K 0.05 -s 0.5 -L 1000 -t 64
minimap2	CycloneSEQ/ONT	-x ava-ont -t 64
minimap2	PacBio	-x ava-pb -t 64
MHAP	CycloneSEQ/ONT/PacBio	–repeat-weight 0.9 –repeat-idf-scale 10 -k 16 –store-full-id –num-hashes 768 –num-min-matches 20 –threshold 0.73 –filter-threshold 0.0000001 –ordered-sketch-size 1536 –ordered-kmer-size 12 –min-olap-length 500

Table S6. *De novo* assembly statistics of Fedrann-Shasta and Shasta

Method	Fedrann-Shasta	Shasta	Fedrann-Shasta	Shasta
**Dataset**	**H4C**	**H4C**	**H4O**	**H4O**
Total assembly length (Gb)	2.84	2.85	2.81	2.80
N50 (Mb)	44.45	45.07	17.07	5.73
NGA50 (Mb)	8.00	8.23	5.71	3.46
Largest contig length (Mb)	138.00	137.99	97.89	29.09
K-mer QV	35.74	35.71	37.51	37.58
K-mer completeness (%)	95.56	95.75	95.42	94.94
BUSCO completeness (%)	98.10	98.30	97.80	97.00

Table S7. Performance of Fedrann under various CPU and memory limitations.
Benchmarks were conducted on dataset H4C within Docker containers to simulate hardware constraints. RAM: random access memory. Prec.: precision. HDD: hard disk drive. SSD: solid state drive.

Threads	Memory Limit	Swap Medium	Wall-clock Time (h)	Peak RAM (GB)	Prec. (k=6)	Prec. (k=12)	Prec. (k=18)
64	2 TB	HDD	3.02	357.45	99.62%	99.44%	99.25%
64	256 GB	HDD	15.69	250.73	99.62%	99.44%	99.24%
32	230 GB	SSD	14.87	228.10	99.62%	99.44%	99.25%
64	128 GB	HDD	Failed	—	—	—	—
