# Supplementary material for: FEDRANN: effective long-read overlap detection based on dimensionality reduction and approximate nearest neighbors
Source: Gigascience. 2026 May 8;15:giag048. doi: 10.1093/gigascience/giag048 (PMC13201080; doi:10.1093/gigascience/giag048)
Supplement: giag048_GIGA-D-25-00332_revision_1 [file giag048_giga-d-25-00332_revision_1.pdf]

GigaScience

FEDRANN: effective long-read overlap detection based on dimensionality reduction and approximate nearest neighbors

--Manuscript Draft--

**Manuscript Number:**	GIGA-D-25-00332R1
**Full Title:**	FEDRANN: effective long-read overlap detection based on dimensionality reduction and approximate nearest neighbors
**Article Type:**	Research
**Funding Information:**	National Key R&D Program of China (No.2024YFC3406300) — Not applicable<br>"Pioneer" and "Leading Goose" R&D Program of Zhejiang (No.2024C03004) — Not applicable
**Abstract:**	Overlap detection is a key step in de novo genome assembly pipelines based on the Overlap-Layout-Consensus (OLC) paradigm. Existing methods for overlap detection either rely on heuristic seed-and-extension strategies or locality-sensitive hashing (LSH), both of which struggle to handle repetitive genomic regions and the computational burden of large-scale datasets. Here, we present FEDRANN, a novel strategy for overlap graph construction that integrates feature extraction, dimensionality reduction (DR), and approximate nearest neighbor (ANN) search. We find the pipeline combining inverse document frequency (IDF) transformation, sparse random projection (SRP), and NNDescent enables accurate detection of overlaps across diverse datasets. We developed an efficient open-source implementation of this pipeline named Fedrann (https://github.com/jzhang-dev/fedrann). Through systematic benchmarking on real long-read sequencing data, we demonstrate that Fedrann produces overlap graphs comparable to or better than those generated by existing state-of-the-art tools, including MECAT2, minimap2, and wtdbg2, while maintaining competitive runtime. By integrating Fedrann into the Shasta assembler, we successfully reconstructed human whole genomes, achieving high assembly contiguity and quality. Despite being implemented primarily in Python, Fedrann achieves performance parity with tools written in compiled languages by leveraging C-accelerated numerical libraries and optimized batch-based matrix operations. Our results suggest that the combination of dimensionality reduction and ANN techniques offers a robust, scalable framework for accurate overlap detection in long-read assembly and broader sequence similarity search tasks.
**Corresponding Author:**	Yuliang Dong<br>BGI Research, Hangzhou; BGI Research, Shenzhen<br>Shenzhen, CHINA
**Corresponding Author Secondary Information:**	
**Corresponding Author's Institution:**	BGI Research, Hangzhou; BGI Research, Shenzhen
**Corresponding Author's Secondary Institution:**	
**First Author:**	Jia-Yuan Zhang
**First Author Secondary Information:**	
**Order of Authors:**	Jia-Yuan Zhang
	Changjiu Miao
	Teng Qiu
	Junyi He
	Wenqi Cao
	Wei Lin

Powered by Editorial Manager® and ProduXion Manager® from Aries Systems Corporation

	Xiaoshuang Xia
	Lei He
	Chunlei Yang
	Yuhui Sun
	Tao Zeng
	Yuxiang Li
	Xun Xu
	Yijun Ruan
	Yuliang Dong
**Order of Authors Secondary Information:**	
**Response to Reviewers:**	Please note that a professionally formatted version of our response has been uploaded as a separate PDF file titled "Response to reviewers' comments". > We kindly suggest referring to that PDF for a more convenient reading experience.<br><br>FEDRANN: effective long-read overlap detection based on dimensionality reduction and approximate nearest neighbors<br>Response to reviewers' comments<br><br>Reviewer #1<br>--<br><br>Comment 1.1<br><br>Summary<br>This paper presents FEDRANN, a novel approach to overlap detection in long-read genome assembly that combines feature extraction, dimensionality reduction (DR), and approximate nearest neighbor (ANN) search. The authors systematically evaluate a range of design choices and implement the best-performing pipeline (IDF-SRP-NNDescent) as an open-source tool, Fedrann. Benchmarking against state-of-the-art tools (minimap2, MECAT2, wtdbg2, BLEND, xRead, MHAP) shows that Fedrann achieves competitive or superior accuracy and overlap graph quality across multiple sequencing platforms (ONT, PacBio HiFi, CycloneSEQ), while maintaining reasonable runtime. The conceptual framing of overlap detection as a k-NN search problem is both innovative and potentially influential.<br>Major Strengths<br>1.Novel conceptual framework: Framing overlap detection as a k-NN search problem,<br>2.drawing analogies from single-cell analysis, is creative and opens new algorithmic possibilities for genome assembly.<br>3.Systematic evaluation: The authors carefully assess multiple feature extraction, DR, and ANN methods before converging on the optimal pipeline.<br>4.Strong empirical results: Fedrann demonstrates high accuracy and graph quality, often outperforming established tools while remaining runtime-efficient.<br><br>We thank the reviewer for these positive comments.<br>--<br><br>Comment 1.2<br><br>Major Concerns and Recommendations<br>1. Memory consumption is a critical limitation<br>Fedrann requires >700 GB RAM for human genome datasets, which makes the tool impractical for most research labs and cost-prohibitive for cloud use.<br>While acknowledged, this limitation is somewhat downplayed. In its current state, Fedrann may be restricted to only very high-resource environments.<br>Recommendation: Either (a) demonstrate initial results of memory-reduction strategies (e.g., shared memory, memory-mapped structures, GPU acceleration), or (b) more prominently highlight this as a key limitation restricting practical adoption.

Powered by Editorial Manager® and ProduXion Manager® from Aries Systems Corporation

We thank the reviewer for raising this important concern regarding memory consumption and practical usability. We agree that memory usage is a critical factor for the adoption of Fedrann and that this limitation required both technical improvements and clearer discussion in the manuscript.
In response, we have substantially refactored the Fedrann workflow to reduce memory usage. The revised implementation replaces the original monolithic feature matrix construction with an incremental, batch-based design. Feature construction and dimensionality reduction are now performed on fixed-size batches of reads. A key optimization is the fusion of IDF weighting and Sparse Random Projection into a single matrix multiplication, based on our observation that both operations are linear. The resulting weighted projection matrix is constructed once and shared across worker processes, eliminating intermediate feature matrices and reducing redundant memory usage. These changes accelerated computation and reduced the peak memory requirement for the H1C human genome dataset from over 700 GB to approximately 357 GB (about a 50% reduction), bringing Fedrann within the capabilities of standard high-memory compute nodes. As detailed in our response to Comment 2.2, the utilization of system swap space further enables Fedrann to process human genome datasets on systems with less than 256 GB physical memory, albeit at the cost of increased computational time.
We added Supplementary Figure S7 and a new section in the Results titled "Efficient implementation of the IDF-SRP-NNDescent pipeline" to describe the algorithmic and architectural optimizations developed for Fedrann.
We updated the “Fedrann enables high-precision, time-efficient overlap detection on large genomes” section in Results with the latest benchmarking results showing reduced time and memory usage of Fedrann.
We rewrote the “Fedrann implementation” section in Methods to provide details on the updated Fedrann architecture and workflow.
The Discussion section was revised to more clearly state the high memory requirement of the tool. We wrote: “Despite these strengths, the overall memory consumption of Fedrann remains higher than that of most existing overlap detection tools benchmarked, highlighting an important direction for future optimization through more memory-efficient data representations, shared-memory designs, or alternative implementations of ANN methods.”

Comment 1.3

2. Incomplete evaluation at the assembly pipeline level
The paper evaluates overlap graph quality but does not show results on final genome assemblies.
This is a critical gap: improved overlaps must ultimately lead to better assemblies (NGA50, BUSCO scores, base accuracy). Without this, the utility of Fedrann remains uncertain.
Recommendation: Include at least one end-to-end assembly experiment, comparing standard metrics with and without Fedrann overlaps. Even a single human or model genome dataset would provide crucial evidence.

We appreciate the reviewer’s suggestion to evaluate Fedrann at the assembly pipeline level. We fully agree that the ultimate measure of an overlap detector's utility is its impact on the quality of the final genome assembly, including metrics such as N50, BUSCO scores, and base accuracy.
In response, we developed the Fedrann-Shasta pipeline by modifying the Shasta assembler to ingest Fedrann’s output for overlap graph construction. We then performed de novo assembly of the human genome using datasets from CycloneSEQ (H4C) and Oxford Nanopore (H4O) and benchmarked the results against the native Shasta pipeline. Our results demonstrate that Fedrann yields high-quality assembly contigs with metrics that are comparable (H4C) or significantly better (H4O) than those of the standard Shasta pipeline.
Nonetheless, we noted that integrating external overlap results into existing assembly frameworks presents inherent technical hurdles. As discussed in the manuscript, many current assemblers rely on opaque, in-memory data structures that tightly couple the discovery and assembly stages. Because this integration is currently constrained by the internal architecture of established tools, we consider these results a successful proof-of-concept rather than the upper limit of Fedrann’s potential. We hope that

Fedrann’s transparent data structures will simplify such efforts for the community in the future. Furthermore, we are committed to developing a purpose-built assembler optimized specifically for Fedrann’s output, which will be the focus of our next manuscript.
We added Supplementary Figures S11, S12 and Supplementary Table S6 to describe the assembly results generated with the Fedrann-Shasta pipeline and compare them with the standard Shasta results. These results are summarized in a new "The Fedrann-Shasta pipeline generates high-quality human whole-genome assembly contigs" section in Results.
We expanded the Discussion to address the technical hurdles of integrating Fedrann into existing assembly frameworks and to emphasize its utility as a modular, transparent building block for future assembly algorithms.
We added two sections in the Methods describing the implementation of Fedrann-Shasta and the evaluation of de novo genomic assemblies, respectively.

Comment 1.4

3. Unclear treatment of k-mer size
The manuscript does not specify the k-mer length (k) used for feature extraction, even though k-mer size critically determines sensitivity/specificity of overlaps:
Smaller k (e.g., 15): more sensitive but prone to spurious matches.
Larger k (e.g., 31): more specific but may miss overlaps in shorter or error-prone reads.
The authors mention filtering low-frequency k-mers and sampling 10–15% of the alphabet, but do not justify frequency thresholds or sampling fractions.
Recommendation: Explicitly state the k-mer size used, justify parameter choices, and discuss their impact on overlap accuracy.

We fully agree with the reviewer that parameters such as k-mer size and sampling fractions are pivotal to the sensitivity and specificity of overlap detection. Fedrann utilizes a transparent parameter space where most variables exhibit predictable linear trade-offs: increasing parameter values generally enhances accuracy at the cost of increased memory consumption and execution time. We have updated the manuscript to provide comprehensive details and systematic guidance on parameter selection.
Supplementary Table S4 was been updated to include the specific k-mer sizes utilized for each dataset in this study.
We added Supplementary Figure S13 describing the impact of various parameters to Fedrann performance. These results were summarized in a new "Application notes" section in Results.
We added Supplementary Note 1 to provide detailed guidelines to assist users in selecting optimal parameters, including k-mer sizes and sampling fractions, based on their specific datasets.

Comment 1.5

Minor Comments
Figures S4–S6 (embedding dimension analysis) could be better explained in the main text with more intuitive interpretation.

We thank the reviewer for this insightful suggestion. We agree that providing a more intuitive explanation of the embedding dimension is crucial for understanding the algorithm's behavior.
We have significantly revised the relevant section to better articulate the trade-offs involved. Specifically, we now interpret the embedding dimension as a measure of "feature resolution"—where higher dimensions allow the system to better distinguish between similar but non-overlapping sequences. We have also introduced the concept of an "informational saturation point" to explain the diminishing returns observed in our sensitivity analysis, clarifying that 1,000 dimensions represent an optimal balance between structural fidelity and computational efficiency.
We rewrote the last two paragraphs of “Sparse random projection enables scalable and accurate dimensionality reduction” in Results to provide intuitive interpretation on the experimental results.

Powered by Editorial Manager® and ProduXion Manager® from Aries Systems Corporation

Benchmarking fairness: tools designed for high recall (e.g., minimap2, MECAT2) may be disadvantaged by post-processing into “top k” mode. Clarify this limitation in comparisons.

We appreciate the reviewer's insightful comment regarding benchmarking fairness. We acknowledge that tools like minimap2 and MECAT2 are inherently optimized for high recall by reporting all candidate overlaps above a certain quality threshold, whereas our approach (and other k-NN based methods) focuses on identifying the "top-k" most similar neighbors. However, we do not consider this a limitation of Fedrann or our benchmarking methodology. In overlap graph construction for de novo assembly, longer overlaps are generally more informative than shorter ones. Some assembly frameworks, such as Shasta, explicitly retain only the top k overlaps for each read during graph construction, while many other assemblers prune shorter overlaps as transitive edges during simplification. This biological context justifies a focus on identifying the most significant overlaps rather than all potential matches. In the revised manuscript, we have included additional results and discussion to examine these design differences in detail.
To ensure a more balanced view, we added Supplementary Figure S10, which demonstrates the changes in precision and recall for each benchmarked tool with respect to the value of k. This analysis provides transparency regarding the inherent design of Fedrann, illustrating that the tool is optimized for high-precision overlap detection and is unlikely to achieve exhaustive recall without a substantial sacrifice in precision.
We added a new paragraph in the "Fedrann enables high-precision, time-efficient overlap detection on large genomes" section in Results to explicitly contrast threshold-based and k-NN based overlap detection and highlight the impact of different design choices on benchmarking results.

Comment 1.6

Minor Corrections
Figure 1: Step numbering is currently (1), (3), (4). Step (2) is missing

We sincerely thank the reviewer for their meticulous attention to detail. We have corrected the step numbering of Figure 1 in the updated manuscript.
The caption of Figure 1 was updated to fix this issue.

Comment 1.7

Overall Recommendation
This paper presents a novel and promising framework for overlap detection with strong methodological rigor and empirical results. However, two major gaps — excessive memory usage and lack of assembly-level validation — must be addressed before the work can be considered fully convincing. In addition, clarity on basic parameters such as k-mer size is necessary for robustness.

We thank the reviewer for their thoughtful evaluation. We have addressed the concerns regarding memory consumption and assembly-level validation by implementing batch-processing optimizations and developing the Fedrann-Shasta pipeline for end-to-end benchmarking. We hope these revisions and the detailed responses provided above fully address your concerns and demonstrate the robustness of the Fedrann framework.

Reviewer #2

Comment 2.1

The authors present FEDRANN, a novel overlap detection framework that integrates feature extraction, sparse random projection-based dimensionality reduction, and

Powered by Editorial Manager® and ProduXion Manager® from Aries Systems Corporation

approximate nearest-neighbor searching via NNDescent. The manuscript is clearly written and supported by extensive benchmarking across multiple platforms (ONT, HiFi, CycloneSEQ) and genome scales. The work introduces a notably different paradigm from traditional seed-and-extend and MinHash/LSH-based methods, and the demonstrated improvements in repeat-rich genomic regions highlight the potential value of this approach for OLC-based assembly pipelines.

We thank the reviewer for these positive comments.

Comment 2.2

Below, I outline several concerns and suggestions for improvement:
FEDRANN requires more than 700 GB of RAM to process human datasets, which far exceeds the resource demands of the comparison tools. In practice, assembly groups typically operate under <256 GB per compute node, meaning the current implementation may be unusable for many users. Improving memory efficiency is a critical issue that must be addressed.

We thank the reviewer for this pragmatic observation regarding memory constraints. We have taken this feedback seriously and implemented both algorithmic and deployment-level solutions to ensure Fedrann is accessible to a broader range of users.
Algorithmic optimization: As detailed in our response to Comment 1.2, we have refactored the memory architecture, transitioning from a monolithic matrix construction to an incremental batch-processing workflow. By integrating shared-memory structures for the projection matrix, we have successfully halved the peak physical memory requirement for human genome H4C datasets from >700 GB to approximately 357 GB.
Support for 256 GB memory environments: Recognizing that 256 GB RAM is a common standard for bioinformatics compute nodes, we have validated a deployment strategy to accommodate these systems. Users can now process human whole-genome sequencing datasets on 256 GB machines by leveraging system swap space. Our benchmark tests confirm that while this mode incurs a performance penalty in terms of execution time, it does not impact the accuracy of the overlap detection or final assembly. Furthermore, we demonstrated that utilizing NVMe-based Solid State Drives (SSDs) for the swap partition significantly mitigates the latency typically associated with disk-swapping; for instance, a 32-thread SSD configuration outperformed a 64-thread HDD execution. This established workflow makes Fedrann a viable tool for memory-constrained environments without necessitating hardware upgrades.
We have significantly refactored the Fedrann workflow to optimize execution time and memory efficiency. The corresponding Results, Discussion, and Methods sections have been updated to reflect these improvements, as detailed in our response to Comment 1.2.
We added Supplementary Table S7, which provides benchmark results for Fedrann under various memory constraints. These tests confirm that Fedrann can successfully process human whole-genome datasets on 256 GB machines. These findings are now described in a new "Application notes" section within the Results.
We have added Supplementary Note 2, which provides a comprehensive technical guide for users on how to configure system swap space to run Fedrann in memory-limited environments.

Comment 2.3

The method involves multiple parameters that currently lack practical tuning guidance (e.g., read accuracy, kmer alphabet size, coverage depth, species complexity). The authors should provide a calibration guide with representative defaults for ONT/HiFi/CycloneSEQ and for genomes of different sizes, as well as runtime-accuracy trade-off curves for the SRP embedding dimension and ANN search settings.

We thank the reviewer for this constructive suggestion regarding the usability and transparency of our parameter settings. To better support the community in deploying our tool across various research scenarios, we have enhanced the manuscript by

providing a systematic calibration guide and empirical performance analysis. These additions offer data-driven guidance on balancing predictive accuracy with computational efficiency for different sequencing technologies and genome sizes.
We added Supplementary Note 1 to include a comprehensive parameter tuning guide. This guide provides recommendations for k-mer size, sampling fractions, and embedding dimensions, tailored specifically to different sequencing platforms (ONT, PacBio HiFi, and CycloneSEQ) and genomic complexities ranging from bacteria to humans.
Supplementary Table S4 has been updated to list the exact Fedrann parameter configurations used for every dataset in this study. These settings were empirically determined to achieve an optimal balance between precision and execution speed.
We have added runtime-accuracy trade-off curves for the Sparse Random Projection (SRP) embedding dimension and ANN search settings in Supplementary Figure S13. These results are now analyzed in the "Application notes" section of Results, providing users with a quantitative understanding of how parameter adjustments influence computational cost and sensitivity.

Comment 2.4

FEDRANN retrieves only the top-k neighbors per read, whereas most baseline tools attempt full overlap discovery. It remains unclear whether improved precision comes at the cost of substantial recall losses. Evaluation should include overlap recall plots and precision-recall analysis stratified by overlap length.

We sincerely appreciate the reviewer's constructive suggestion regarding the evaluation of recall and precision. We agree that a comprehensive understanding of the trade-off between the "top-k" retrieval strategy and exhaustive recall is essential for validating Fedrann’s effectiveness.
In response to your guidance, we performed two new sets of experiments using the H3C dataset. First, we conducted a precision-recall analysis across a range of k values to observe how reporting depth influences performance. We found that while threshold-based tools prioritize exhaustive discovery, Fedrann’s k-NN approach is optimized for high-precision retrieval of the most significant overlaps. While Fedrann can achieve recall comparable to exhaustive tools like minimap2 by increasing k, this results in considerable losses in precision because Fedrann is designed to report exactly k candidates, even for reads with fewer biological overlaps. Second, we stratified recall by overlap length, which confirmed that Fedrann successfully prioritizes longer, more informative overlaps. These are biologically critical for de novo assembly, as shorter overlaps are frequently pruned as transitive edges during graph simplification. We have updated the manuscript and supplementary materials to reflect these findings and provide transparency regarding the conditions under which different tools excel.
We added Supplementary Figure S9 to illustrates the dynamic changes in recall and precision for Fedrann and existing tools as k increases.
We added Supplementary Figure S10, presenting recall rates stratified by overlap length thresholds.
We added a dedicated paragraph in the "Fedrann enables high-precision, time-efficient overlap detection on large genomes" section in Results to contrast threshold-based and k-NN based detection. This section now explicitly describes how recall and precision respond to changes in k, providing users with clearer expectations.

Comment 2.5

Overlap precision and graph connectivity are useful proxies, but they do not guarantee better genome reconstruction. The evaluation should integrate FEDRANN into at least one complete OLC assembly workflow (e.g., WTDBG2, Canu, Shasta) and report standard assembly metrics (N50/NG50, BUSCO, QV, and structural variant detection). Assembly contiguity in complex immune loci (e.g., HLA, IGK) would further support claims of improved handling of repeats.

We appreciate the reviewer's suggestion to evaluate Fedrann within a complete assembly workflow and specifically to investigate complex immune loci. We agree that metrics like overlap precision are most valuable when they translate into improved

	genomic reconstruction.<br>In response, we developed the Fedrann-Shasta pipeline by modifying the Shasta assembler to ingest Fedrann's output for overlap graph construction. We performed de novo assembly of the human genome using CycloneSEQ (H4C) and Oxford Nanopore (H4O) datasets and benchmarked the results against the native Shasta pipeline. Our results demonstrate that Fedrann yields high-quality assembly contigs with metrics (N50, BUSCO, and QV) that are comparable to (H4C) or significantly better than (H4O) the standard Shasta pipeline.<br>Furthermore, we specifically analyzed the HLA region to evaluate Fedrann's performance in repeat-rich regions. While both pipelines partitioned the HLA region into two contigs, the native Shasta assembly exhibited a collapse of a ~40 kb repeat unit, incorrectly merging two copies. This misassembly was correctly resolved in the Fedrann-Shasta assembly. This suggests that Fedrann's high-accuracy overlap detection provides the specificity required to resolve complex genomic contexts that may be prone to collapse in traditional heuristic-based pipelines.<br>We added Supplementary Figures S11, S12 and Supplementary Table S6 to describe the assembly results generated with the Fedrann-Shasta pipeline and compare them with the standard Shasta results. These results are summarized in a new "The Fedrann-Shasta pipeline generates high-quality human whole-genome assembly contigs" section in Results.<br>We expanded the Discussion to address the technical hurdles of integrating Fedrann into existing assembly frameworks and to emphasize its utility as a modular, transparent building block for future assembly algorithms.<br>We added two sections in Methods describing the implementation of Fedrann-Shasta and de novo assembly evaluation, respectively.
**Additional Information:**	
**Question**	**Response**
Are you submitting this manuscript to a special series or article collection?	No
**Experimental design and statistics**<br><br>Full details of the experimental design and statistical methods used should be given in the Methods section, as detailed in our Minimum Standards Reporting Checklist. Information essential to interpreting the data presented should be made available in the figure legends.<br><br>Have you included all the information requested in your manuscript?	Yes
**Resources**<br><br>A description of all resources used, including antibodies, cell lines, animals and software tools, with enough information to allow them to be uniquely identified, should be included in the Methods section. Authors are strongly encouraged to cite Research Resource Identifiers (RRIDs) for antibodies, model	Yes

organisms and tools, where possible.<br><br>Have you included the information requested as detailed in our Minimum Standards Reporting Checklist?	
**Availability of data and materials**<br><br>All datasets and code on which the conclusions of the paper rely must be either included in your submission or deposited in publicly available repositories (where available and ethically appropriate), referencing such data using a unique identifier in the references and in the “Availability of Data and Materials” section of your manuscript.<br><br>Have you have met the above requirement as detailed in our Minimum Standards Reporting Checklist?	Yes
GigaScience has policies and guidelines in place for the use of generative AI-writing tools such as ChatGPT. If you have used such writing tools to assist with writing the manuscript this must be declared and cited in the text. Authors should not list AI-writing tools and other AI-assisted technologies as an author or co-author and should acknowledge that they are fully responsible for text generated or refined by AI-writing tools.<p><br><br>A summary of use (particularly in the introduction or among methods) needs to be included at the end of the paper, and the outputs should also be included as a supplementary file hosted in GigaDB or other open repositories. Please read our guidelines for more information. <p><br><br>By submitting to GigaScience, you are aware of the journal's AI-writing tools policy, and if you have declared use of such tools below, you have acknowledged this where appropriate in your manuscript and have made a summary of use and outputs available. <p><br><b>AI-assisted writing tools have been	No

used in the preparation of this manuscript?	

Powered by Editorial Manager® and ProduXion Manager® from Aries Systems Corporation

LaTeX - Main Document (TeX file) Click here to access/download;LaTeX - Main Document (TeX file);main.tex

```
This is pdfTeX, Version 3.141592653-2.6-1.40.26 (TeX Live 2024)
(preloaded format=pdflatex 2024.8.2)  12 MAR 2026 04:50
entering extended mode
 restricted \write18 enabled.
 %&-line parsing enabled.
**main.tex
(./main.tex
LaTeX2e <2024-06-01> patch level 2
L3 programming layer <2024-05-27>
(./oup-contemporary.cls
Document Class: oup-contemporary 2023/06/12, v1.2
(c:/texlive/2024/texmf-dist/tex/latex/base/article.cls
Document Class: article 2024/02/08 v1.4n Standard LaTeX document class
(c:/texlive/2024/texmf-dist/tex/latex/base/size10.clo
File: size10.clo 2024/02/08 v1.4n Standard LaTeX file (size option)
)
\c@part=\count194
\c@section=\count195
\c@subsection=\count196
\c@subsubsection=\count197
\c@paragraph=\count198
\c@subparagraph=\count199
\c@figure=\count266
\c@table=\count267
\abovecaptionskip=\skip49
\belowcaptionskip=\skip50
\bibindent=\dimen141
) (c:/texlive/2024/texmf-dist/tex/latex/base/inputenc.sty
Package: inputenc 2024/02/08 v1.3d Input encoding file
\inpenc@prehook=\toks17
\inpenc@posthook=\toks18
) (c:/texlive/2024/texmf-dist/tex/latex/base/fontenc.sty
Package: fontenc 2021/04/29 v2.0v Standard LaTeX package
) (c:/texlive/2024/texmf-dist/tex/generic/iftex/ifpdf.sty
Package: ifpdf 2019/10/25 v3.4 ifpdf legacy package. Use iftex instead.
(c:/texlive/2024/texmf-dist/tex/generic/iftex/iftex.sty
Package: iftex 2022/02/03 v1.0f TeX engine tests
)) (c:/texlive/2024/texmf-dist/tex/latex/microtype/microtype.sty
Package: microtype 2024/03/29 v3.1b Micro-typographical refinements (RS)
(c:/texlive/2024/texmf-dist/tex/latex/graphics/keyval.sty
Package: keyval 2022/05/29 v1.15 key=value parser (DPC)
\KV@toks@=\toks19
) (c:/texlive/2024/texmf-dist/tex/latex/etoolbox/etoolbox.sty
Package: etoolbox 2020/10/05 v2.5k e-TeX tools for LaTeX (JAW)
\etb@tempcnta=\count268
)
\MT@toks=\toks20
\MT@tempbox=\box52
\MT@count=\count269
LaTeX Info: Redefining \noprotrusionifhmode on input line 1061.
LaTeX Info: Redefining \leftprotrusion on input line 1062.
\MT@prot@toks=\toks21
LaTeX Info: Redefining \rightprotrusion on input line 1081.
LaTeX Info: Redefining \textls on input line 1392.
```

```
\MT@outer@kern=\dimen142
LaTeX Info: Redefining \textmicrotypecontext on input line 2013.
\MT@listname@count=\count270
(c:/texlive/2024/texmf-dist/tex/latex/microtype/microtype-pdftex.def
File: microtype-pdftex.def 2024/03/29 v3.1b Definitions specific to
pdftex (RS)

LaTeX Info: Redefining \lsstyle on input line 902.
LaTeX Info: Redefining \lslig on input line 902.
\MT@outer@space=\skip51
)
Package microtype Info: Loading configuration file microtype.cfg.
(c:/texlive/2024/texmf-dist/tex/latex/microtype/microtype.cfg
File: microtype.cfg 2024/03/29 v3.1b microtype main configuration file
(RS)
)) (c:/texlive/2024/texmf-dist/tex/latex/euler/euler.sty
Package: euler 1995/03/05 v2.5
Package: `euler' v2.5 <1995/03/05> (FJ and FMi)
LaTeX Font Info:    Redeclaring symbol font `letters' on input line 35.
LaTeX Font Info:    Encoding `OML' has changed to `U' for symbol font
(Font)              `letters' in the math version `normal' on input line
35.
LaTeX Font Info:    Overwriting symbol font `letters' in version `normal'
(Font)                  OML/cmm/m/it --> U/eur/m/n on input line 35.
LaTeX Font Info:    Encoding `OML' has changed to `U' for symbol font
(Font)              `letters' in the math version `bold' on input line
35.
LaTeX Font Info:    Overwriting symbol font `letters' in version `bold'
(Font)                  OML/cmm/b/it --> U/eur/m/n on input line 35.
LaTeX Font Info:    Overwriting symbol font `letters' in version `bold'
(Font)                  U/eur/m/n --> U/eur/b/n on input line 36.
LaTeX Font Info:    Redeclaring math symbol \Gamma on input line 47.
LaTeX Font Info:    Redeclaring math symbol \Delta on input line 48.
LaTeX Font Info:    Redeclaring math symbol \Theta on input line 49.
LaTeX Font Info:    Redeclaring math symbol \Lambda on input line 50.
LaTeX Font Info:    Redeclaring math symbol \Xi on input line 51.
LaTeX Font Info:    Redeclaring math symbol \Pi on input line 52.
LaTeX Font Info:    Redeclaring math symbol \Sigma on input line 53.
LaTeX Font Info:    Redeclaring math symbol \Upsilon on input line 54.
LaTeX Font Info:    Redeclaring math symbol \Phi on input line 55.
LaTeX Font Info:    Redeclaring math symbol \Psi on input line 56.
LaTeX Font Info:    Redeclaring math symbol \Omega on input line 57.
\symEulerFraktur=\mathgroup4
LaTeX Font Info:    Overwriting symbol font `EulerFraktur' in version
`bold'
(Font)                  U/euf/m/n --> U/euf/b/n on input line 63.
LaTeX Info: Redefining \oldstylenums on input line 85.
\symEulerScript=\mathgroup5
LaTeX Font Info:    Overwriting symbol font `EulerScript' in version
`bold'
(Font)                  U/eus/m/n --> U/eus/b/n on input line 93.
LaTeX Font Info:    Redeclaring math symbol \aleph on input line 97.
LaTeX Font Info:    Redeclaring math symbol \Re on input line 98.
LaTeX Font Info:    Redeclaring math symbol \Im on input line 99.
```

```
LaTeX Font Info:    Redeclaring math delimiter \vert on input line 101.
LaTeX Font Info:    Redeclaring math delimiter \backslash on input line
103.
LaTeX Font Info:    Redeclaring math symbol \neg on input line 106.
LaTeX Font Info:    Redeclaring math symbol \wedge on input line 108.
LaTeX Font Info:    Redeclaring math symbol \vee on input line 110.
LaTeX Font Info:    Redeclaring math symbol \setminus on input line 112.
LaTeX Font Info:    Redeclaring math symbol \sim on input line 113.
LaTeX Font Info:    Redeclaring math symbol \mid on input line 114.
LaTeX Font Info:    Redeclaring math delimiter \arrowvert on input line
116.
LaTeX Font Info:    Redeclaring math symbol \mathsection on input line
117.
\symEulerExtension=\mathgroup6
LaTeX Font Info:    Redeclaring math symbol \coprod on input line 125.
LaTeX Font Info:    Redeclaring math symbol \prod on input line 125.
LaTeX Font Info:    Redeclaring math symbol \sum on input line 125.
LaTeX Font Info:    Redeclaring math symbol \intop on input line 130.
LaTeX Font Info:    Redeclaring math symbol \ointop on input line 131.
LaTeX Font Info:    Redeclaring math symbol \braceld on input line 132.
LaTeX Font Info:    Redeclaring math symbol \bracerd on input line 133.
LaTeX Font Info:    Redeclaring math symbol \bracelu on input line 134.
LaTeX Font Info:    Redeclaring math symbol \braceru on input line 135.
LaTeX Font Info:    Redeclaring math symbol \infty on input line 136.
LaTeX Font Info:    Redeclaring math symbol \nearrow on input line 153.
LaTeX Font Info:    Redeclaring math symbol \searrow on input line 154.
LaTeX Font Info:    Redeclaring math symbol \nwarrow on input line 155.
LaTeX Font Info:    Redeclaring math symbol \swarrow on input line 156.
LaTeX Font Info:    Redeclaring math symbol \Leftrightarrow on input line
157.
LaTeX Font Info:    Redeclaring math symbol \Leftarrow on input line 158.
LaTeX Font Info:    Redeclaring math symbol \Rightarrow on input line
159.
LaTeX Font Info:    Redeclaring math symbol \leftrightarrow on input line
160.
LaTeX Font Info:    Redeclaring math symbol \leftarrow on input line 161.
LaTeX Font Info:    Redeclaring math symbol \rightarrow on input line
163.
LaTeX Font Info:    Redeclaring math delimiter \uparrow on input line
166.
LaTeX Font Info:    Redeclaring math delimiter \downarrow on input line
168.
LaTeX Font Info:    Redeclaring math delimiter \updownarrow on input line
170.
LaTeX Font Info:    Redeclaring math delimiter \Uparrow on input line
172.
LaTeX Font Info:    Redeclaring math delimiter \Downarrow on input line
174.
LaTeX Font Info:    Redeclaring math delimiter \Updownarrow on input line
176.
LaTeX Font Info:    Redeclaring math symbol \leftharpoonup on input line
177.
LaTeX Font Info:    Redeclaring math symbol \leftharpoondown on input
line 178.
```

```
LaTeX Font Info:    Redeclaring math symbol \rightharpoonup on input line
179.
LaTeX Font Info:    Redeclaring math symbol \rightharpoondown on input
line 180
.
LaTeX Font Info:    Redeclaring math delimiter \lbrace on input line 182.
LaTeX Font Info:    Redeclaring math delimiter \rbrace on input line 184.
\symcmmigroup=\mathgroup7
LaTeX Font Info:    Overwriting symbol font `cmmigroup' in version `bold'
(Font)                  OML/cmm/m/it --> OML/cmm/b/it on input line 200.
LaTeX Font Info:    Redeclaring math accent \vec on input line 201.
LaTeX Font Info:    Redeclaring math symbol \triangleleft on input line
202.
LaTeX Font Info:    Redeclaring math symbol \triangleright on input line
203.
LaTeX Font Info:    Redeclaring math symbol \star on input line 204.
LaTeX Font Info:    Redeclaring math symbol \lhook on input line 205.
LaTeX Font Info:    Redeclaring math symbol \rhook on input line 206.
LaTeX Font Info:    Redeclaring math symbol \flat on input line 207.
LaTeX Font Info:    Redeclaring math symbol \natural on input line 208.
LaTeX Font Info:    Redeclaring math symbol \sharp on input line 209.
LaTeX Font Info:    Redeclaring math symbol \smile on input line 210.
LaTeX Font Info:    Redeclaring math symbol \frown on input line 211.
LaTeX Font Info:    Redeclaring math accent \grave on input line 245.
LaTeX Font Info:    Redeclaring math accent \acute on input line 246.
LaTeX Font Info:    Redeclaring math accent \tilde on input line 247.
LaTeX Font Info:    Redeclaring math accent \ddot on input line 248.
LaTeX Font Info:    Redeclaring math accent \check on input line 249.
LaTeX Font Info:    Redeclaring math accent \breve on input line 250.
LaTeX Font Info:    Redeclaring math accent \bar on input line 251.
LaTeX Font Info:    Redeclaring math accent \dot on input line 252.
LaTeX Font Info:    Redeclaring math accent \hat on input line 254.
) (c:/texlive/2024/texmf-dist/tex/latex/merriweather/merriweather.sty
Package: merriweather 2022/09/20 (Bob Tennent) Supports
Merriweather(Sans) font
s for all LaTeX engines.
(c:/texlive/2024/texmf-dist/tex/generic/iftex/ifxetex.sty
Package: ifxetex 2019/10/25 v0.7 ifxetex legacy package. Use iftex
instead.
) (c:/texlive/2024/texmf-dist/tex/generic/iftex/ifluatex.sty
Package: ifluatex 2019/10/25 v1.5 ifluatex legacy package. Use iftex
instead.
) (c:/texlive/2024/texmf-dist/tex/latex/base/textcomp.sty
Package: textcomp 2024/04/24 v2.1b Standard LaTeX package
) (c:/texlive/2024/texmf-dist/tex/latex/xkeyval/xkeyval.sty
Package: xkeyval 2022/06/16 v2.9 package option processing (HA)
(c:/texlive/2024/texmf-dist/tex/generic/xkeyval/xkeyval.tex
(c:/texlive/2024/te
xmf-dist/tex/generic/xkeyval/xkvutils.tex
\XKV@toks=\toks22
\XKV@tempa@toks=\toks23
)
\XKV@depth=\count271
```

```
File: xkeyval.tex 2014/12/03 v2.7a key=value parser (HA)
)) (c:/texlive/2024/texmf-dist/tex/latex/base/fontenc.sty
Package: fontenc 2021/04/29 v2.0v Standard LaTeX package
) (c:/texlive/2024/texmf-dist/tex/latex/fontaxes/fontaxes.sty
Package: fontaxes 2020/07/21 v1.0e Font selection axes
LaTeX Info: Redefining \upshape on input line 29.
LaTeX Info: Redefining \itshape on input line 31.
LaTeX Info: Redefining \slshape on input line 33.
LaTeX Info: Redefining \swshape on input line 35.
LaTeX Info: Redefining \scshape on input line 37.
LaTeX Info: Redefining \sscshape on input line 39.
LaTeX Info: Redefining \ulcshape on input line 41.
LaTeX Info: Redefining \textsw on input line 47.
LaTeX Info: Redefining \textssc on input line 48.
LaTeX Info: Redefining \textulc on input line 49.
)) (c:/texlive/2024/texmf-dist/tex/latex/mathastext/mathastext.sty
Package: mathastext 2024/07/27 v1.4b Use the text font in math mode (JFB)

Package mathastext Info: Starting the math mode configuration.
\mst@exists@muskip=\muskip17
\mst@forall@muskip=\muskip18
\mst@prime@muskip=\muskip19
\mst@do@nonletters=\toks24
\mst@undo@nonletters=\toks25
\mst@do@easynonletters=\toks26
\mst@undo@easynonletters=\toks27
\symmtoperatorfont=\mathgroup8
\symmtletterfont=\mathgroup9
(   mathastext:   ) ! and ?
(   mathastext:   ) punctuation: , . : ; and \colon
LaTeX Info: Redefining \relbar on input line 1201.
LaTeX Info: Redefining \rightarrowfill on input line 1202.
LaTeX Info: Redefining \leftarrowfill on input line 1205.
(   mathastext:   ) + and =
LaTeX Info: Redefining \Relbar on input line 1298.
(   mathastext:   ) adding = ; and + to \nfss@catcodes
(   mathastext:   ) parentheses ( ) [ ] and slash /
(   mathastext:   ) alldelims: < > \backslash \setminus | \vert \mid \{
\}
LaTeX Font Info:    Redeclaring math symbol \setminus on input line 1364.
LaTeX Info: Redefining \models on input line 1383.
(   mathastext:   ) \# \mathdollar \% \&
(   mathastext:   ) \imath and \jmath
LaTeX Font Info:    Overwriting math alphabet `\Mathnormalbold' in
version `nor
mal'
(Font)                  T1/Merriwthr-OsF/b/it --> T1/Merriwthr-OsF/b/it
on inpu
t line 2863.
LaTeX Font Info:    Overwriting math alphabet `\Mathnormalbold' in
version `bol
d'
(Font)                  T1/Merriwthr-OsF/b/it --> T1/Merriwthr-OsF/b/it
on inpu
```

```
t line 2863.
LaTeX Font Info:    Overwriting symbol font `mtletterfont' in version
`normal'
(Font)                  T1/Merriwthr-OsF/m/it --> T1/Merriwthr-OsF/m/it
on inpu
t line 2863.
LaTeX Font Info:    Overwriting symbol font `mtletterfont' in version
`bold'
(Font)                  T1/Merriwthr-OsF/m/it --> T1/Merriwthr-OsF/b/it
on inpu
t line 2863.
LaTeX Font Info:    Overwriting symbol font `mtoperatorfont' in version
`normal
'
(Font)                  T1/Merriwthr-OsF/m/n --> T1/Merriwthr-OsF/m/n on
input
line 2863.
LaTeX Font Info:    Overwriting symbol font `mtoperatorfont' in version
`bold'
(Font)                  T1/Merriwthr-OsF/m/n --> T1/Merriwthr-OsF/b/n on
input
line 2863.
LaTeX Font Info:    Overwriting math alphabet `\Mathbf' in version
`normal'
(Font)                  T1/Merriwthr-OsF/b/n --> T1/Merriwthr-OsF/b/n on
input
line 2863.
LaTeX Font Info:    Overwriting math alphabet `\Mathbf' in version `bold'
(Font)                  T1/Merriwthr-OsF/b/n --> T1/Merriwthr-OsF/b/n on
input
line 2863.
LaTeX Font Info:    Overwriting math alphabet `\Mathit' in version
`normal'
(Font)                  T1/Merriwthr-OsF/m/it --> T1/Merriwthr-OsF/m/it
on inpu
t line 2863.
LaTeX Font Info:    Overwriting math alphabet `\Mathit' in version `bold'
(Font)                  T1/Merriwthr-OsF/m/it --> T1/Merriwthr-OsF/b/it
on inpu
t line 2863.
LaTeX Font Info:    Overwriting math alphabet `\Mathsf' in version
`normal'
(Font)                  T1/MerriwthrSans-OsF/m/n --> T1/MerriwthrSans-
OsF/m/n o
n input line 2863.
LaTeX Font Info:    Overwriting math alphabet `\Mathsf' in version `bold'
(Font)                  T1/MerriwthrSans-OsF/m/n --> T1/MerriwthrSans-
OsF/b/n o
n input line 2863.
LaTeX Font Info:    Overwriting math alphabet `\Mathtt' in version
`normal'
(Font)                  T1/lmtt/m/n --> T1/lmtt/m/n on input line 2863.
LaTeX Font Info:    Overwriting math alphabet `\Mathtt' in version `bold'
(Font)                  T1/lmtt/m/n --> T1/lmtt/b/n on input line 2863.
```

```
(   mathastext:   ) Latin letters in the `normal', resp. `bold',
(   mathastext:   ) math versions are now set up to use the fonts
(   mathastext:   ) T1/Merriwthr-OsF/m/it, resp. T1/Merriwthr-OsF/b/it.
(   mathastext:   ) Other characters (digits, ...) and \log-like names
will be
(   mathastext:   ) typeset with the n shape.
(   mathastext:   ) \hbar
(   mathastext:   ) minus as endash
(   mathastext:   ) The italic option is in effect.
(   mathastext:   ) \HUGE has been (re)-defined.
(   mathastext:   ) mathastext has declared larger sizes for subscripts.
(   mathastext:   ) To keep LaTeX defaults, use option
`defaultmathsizes'.

Package mathastext Info: Loading is complete.  You can now use
\Mathastext to
(mathastext)             modify the normal and bold math versions.  Use
it
(mathastext)             with optional argument or use \MTDeclareVersion
to
(mathastext)             declare additional math versions.
) (c:/texlive/2024/texmf-dist/tex/latex/relsize/relsize.sty
Package: relsize 2013/03/29 ver 4.1
) (c:/texlive/2024/texmf-dist/tex/latex/ragged2e/ragged2e.sty
Package: ragged2e 2023/06/22 v3.6 ragged2e Package
\CenteringLeftskip=\skip52
\RaggedLeftLeftskip=\skip53
\RaggedRightLeftskip=\skip54
\CenteringRightskip=\skip55
\RaggedLeftRightskip=\skip56
\RaggedRightRightskip=\skip57
\CenteringParfillskip=\skip58
\RaggedLeftParfillskip=\skip59
\RaggedRightParfillskip=\skip60
\JustifyingParfillskip=\skip61
\CenteringParindent=\skip62
\RaggedLeftParindent=\skip63
\RaggedRightParindent=\skip64
\JustifyingParindent=\skip65
) (c:/texlive/2024/texmf-dist/tex/latex/xcolor/xcolor.sty
Package: xcolor 2023/11/15 v3.01 LaTeX color extensions (UK)
(c:/texlive/2024/texmf-dist/tex/latex/graphics-cfg/color.cfg
File: color.cfg 2016/01/02 v1.6 sample color configuration
)
Package xcolor Info: Driver file: pdftex.def on input line 274.
(c:/texlive/2024/texmf-dist/tex/latex/graphics-def/pdftex.def
File: pdftex.def 2024/04/13 v1.2c Graphics/color driver for pdftex
) (c:/texlive/2024/texmf-dist/tex/latex/graphics/mathcolor.ltx)
Package xcolor Info: Model `cmy' substituted by `cmy0' on input line
1350.
Package xcolor Info: Model `hsb' substituted by `rgb' on input line 1354.
Package xcolor Info: Model `RGB' extended on input line 1366.
Package xcolor Info: Model `HTML' substituted by `rgb' on input line
1368.
```

```
Package xcolor Info: Model `Hsb' substituted by `hsb' on input line 1369.
Package xcolor Info: Model `tHsb' substituted by `hsb' on input line
1370.
Package xcolor Info: Model `HSB' substituted by `hsb' on input line 1371.
Package xcolor Info: Model `Gray' substituted by `gray' on input line
1372.
Package xcolor Info: Model `wave' substituted by `hsb' on input line
1373.
) (c:/texlive/2024/texmf-dist/tex/latex/colortbl/colortbl.sty
Package: colortbl 2024/07/06 v1.0i Color table columns (DPC)
(c:/texlive/2024/texmf-dist/tex/latex/tools/array.sty
Package: array 2024/06/14 v2.6d Tabular extension package (FMi)
\col@sep=\dimen143
\ar@mcellbox=\box53
\extrarowheight=\dimen144
\NC@list=\toks28
\extratabsurround=\skip66
\backup@length=\skip67
\ar@cellbox=\box54
)
\everycr=\toks29
\minrowclearance=\skip68
\rownum=\count272
) (c:/texlive/2024/texmf-dist/tex/latex/graphics/graphicx.sty
Package: graphicx 2021/09/16 v1.2d Enhanced LaTeX Graphics (DPC,SPQR)
(c:/texlive/2024/texmf-dist/tex/latex/graphics/graphics.sty
Package: graphics 2024/05/23 v1.4g Standard LaTeX Graphics (DPC,SPQR)
(c:/texlive/2024/texmf-dist/tex/latex/graphics/trig.sty
Package: trig 2023/12/02 v1.11 sin cos tan (DPC)
) (c:/texlive/2024/texmf-dist/tex/latex/graphics-cfg/graphics.cfg
File: graphics.cfg 2016/06/04 v1.11 sample graphics configuration
)
Package graphics Info: Driver file: pdftex.def on input line 106.
)
\Gin@req@height=\dimen145
\Gin@req@width=\dimen146
) (c:/texlive/2024/texmf-dist/tex/latex/xpatch/xpatch.sty
(c:/texlive/2024/texm
f-dist/tex/latex/l3kernel/expl3.sty
Package: expl3 2024-05-27 L3 programming layer (loader)
(c:/texlive/2024/texmf-dist/tex/latex/l3backend/l3backend-pdftex.def
File: l3backend-pdftex.def 2024-05-08 L3 backend support: PDF output
(pdfTeX)
\l__color_backend_stack_int=\count273
\l__pdf_internal_box=\box55
))
Package: xpatch 2020/03/25 v0.3a Extending etoolbox patching commands
(c:/texlive/2024/texmf-dist/tex/latex/l3packages/xparse/xparse.sty
Package: xparse 2024-05-08 L3 Experimental document command parser
)) (c:/texlive/2024/texmf-dist/tex/latex/environ/environ.sty
Package: environ 2014/05/04 v0.3 A new way to define environments
(c:/texlive/2024/texmf-dist/tex/latex/trimspaces/trimspaces.sty
Package: trimspaces 2009/09/17 v1.1 Trim spaces around a token list
)
```

```
\@envbody=\toks30
) (c:/texlive/2024/texmf-dist/tex/latex/lastpage/lastpage.sty
Package: lastpage 2024/07/07 v2.1c lastpage: 2.09 or 2e? (HMM)
(c:/texlive/2024/texmf-dist/tex/latex/lastpage/lastpage2e.sty
Package: lastpage2e 2024/07/07 v2.1c Decide which 2e lastpage version to
use (H
MM)
(c:/texlive/2024/texmf-dist/tex/latex/lastpage/lastpagemodern.sty
Package: lastpagemodern 2024-07-07 v2.1c Refers to last page's name (HMM;
JPG)
\c@lastpagecount=\count274
)
)) (c:/texlive/2024/texmf-dist/tex/latex/graphics/rotating.sty
Package: rotating 2016/08/11 v2.16d rotated objects in LaTeX
(c:/texlive/2024/texmf-dist/tex/latex/base/ifthen.sty
Package: ifthen 2024/03/16 v1.1e Standard LaTeX ifthen package (DPC)
)
\c@r@tfl@t=\count275
\rotFPtop=\skip69
\rotFPbot=\skip70
\rot@float@box=\box56
\rot@mess@toks=\toks31
) (c:/texlive/2024/texmf-dist/tex/latex/graphics/lscape.sty
Package: lscape 2020/05/28 v3.02 Landscape Pages (DPC)
) (c:/texlive/2024/texmf-dist/tex/latex/tools/afterpage.sty
Package: afterpage 2023/07/04 v1.08 After-Page Package (DPC)
\AP@output=\toks32
\AP@partial=\box57
\AP@footins=\box58
) (c:/texlive/2024/texmf-dist/tex/latex/textpos/textpos.sty
Package: textpos 2022/07/23 v1.10.1
Package textpos Info: choosing support for LaTeX3 on input line 60.
\TP@textbox=\box59
\TP@holdbox=\box60
\TPHorizModule=\dimen147
\TPVertModule=\dimen148
\TP@margin=\dimen149
\TP@absmargin=\dimen150
Grid set 16 x 16 = 37.34424pt x 52.81541pt
\TPboxrulesize=\dimen151
\TP@ox=\dimen152
\TP@oy=\dimen153
\TP@tbargs=\toks33
TextBlockOrigin set to 0pt x 0pt
) (c:/texlive/2024/texmf-dist/tex/latex/url/url.sty
\Urlmuskip=\muskip20
Package: url 2013/09/16  ver 3.4  Verb mode for urls, etc.
) (c:/texlive/2024/texmf-dist/tex/latex/newfloat/newfloat.sty
Package: newfloat 2023/10/01 v1.2 Defining new floating environments (AR)
Package newfloat Info: `rotating' package detected.
) (c:/texlive/2024/texmf-dist/tex/latex/mdframed/mdframed.sty
Package: mdframed 2013/07/01 1.9b: mdframed
(c:/texlive/2024/texmf-dist/tex/latex/kvoptions/kvoptions.sty
```

```
Package: kvoptions 2022-06-15 v3.15 Key value format for package options
(HO)
(c:/texlive/2024/texmf-dist/tex/generic/ltxcmds/ltxcmds.sty
Package: ltxcmds 2023-12-04 v1.26 LaTeX kernel commands for general use
(HO)
) (c:/texlive/2024/texmf-dist/tex/latex/kvsetkeys/kvsetkeys.sty
Package: kvsetkeys 2022-10-05 v1.19 Key value parser (HO)
)) (c:/texlive/2024/texmf-dist/tex/latex/zref/zref-abspage.sty
Package: zref-abspage 2023-09-14 v2.35 Module abspage for zref (HO)
(c:/texlive/2024/texmf-dist/tex/latex/zref/zref-base.sty
Package: zref-base 2023-09-14 v2.35 Module base for zref (HO)
(c:/texlive/2024/texmf-dist/tex/generic/infwarerr/infwarerr.sty
Package: infwarerr 2019/12/03 v1.5 Providing info/warning/error messages
(HO)
) (c:/texlive/2024/texmf-dist/tex/generic/kvdefinekeys/kvdefinekeys.sty
Package: kvdefinekeys 2019-12-19 v1.6 Define keys (HO)
) (c:/texlive/2024/texmf-dist/tex/generic/pdftexcmds/pdftexcmds.sty
Package: pdftexcmds 2020-06-27 v0.33 Utility functions of pdfTeX for
LuaTeX (HO
)
Package pdftexcmds Info: \pdf@primitive is available.
Package pdftexcmds Info: \pdf@ifprimitive is available.
Package pdftexcmds Info: \pdfdraftmode found.
) (c:/texlive/2024/texmf-dist/tex/generic/etexcmds/etexcmds.sty
Package: etexcmds 2019/12/15 v1.7 Avoid name clashes with e-TeX commands
(HO)
) (c:/texlive/2024/texmf-dist/tex/latex/auxhook/auxhook.sty
Package: auxhook 2019-12-17 v1.6 Hooks for auxiliary files (HO)
)
Package zref Info: New property list: main on input line 767.
Package zref Info: New property: default on input line 768.
Package zref Info: New property: page on input line 769.
)
\c@abspage=\count276
Package zref Info: New property: abspage on input line 67.
) (c:/texlive/2024/texmf-dist/tex/latex/needspace/needspace.sty
Package: needspace 2010/09/12 v1.3d reserve vertical space
)
\mdf@templength=\skip71
\c@mdf@globalstyle@cnt=\count277
\mdf@skipabove@length=\skip72
\mdf@skipbelow@length=\skip73
\mdf@leftmargin@length=\skip74
\mdf@rightmargin@length=\skip75
\mdf@innerleftmargin@length=\skip76
\mdf@innerrightmargin@length=\skip77
\mdf@innertopmargin@length=\skip78
\mdf@innerbottommargin@length=\skip79
\mdf@splittopskip@length=\skip80
\mdf@splitbottomskip@length=\skip81
\mdf@outermargin@length=\skip82
\mdf@innermargin@length=\skip83
\mdf@linewidth@length=\skip84
\mdf@innerlinewidth@length=\skip85
```

```
\mdf@middlelinewidth@length=\skip86
\mdf@outerlinewidth@length=\skip87
\mdf@roundcorner@length=\skip88
\mdf@footenotedistance@length=\skip89
\mdf@userdefinedwidth@length=\skip90
\mdf@needspace@length=\skip91
\mdf@frametitleaboveskip@length=\skip92
\mdf@frametitlebelowskip@length=\skip93
\mdf@frametitlerulewidth@length=\skip94
\mdf@frametitleleftmargin@length=\skip95
\mdf@frametitlerightmargin@length=\skip96
\mdf@shadowsize@length=\skip97
\mdf@extratopheight@length=\skip98
\mdf@subtitleabovelinewidth@length=\skip99
\mdf@subtitlebelowlinewidth@length=\skip100
\mdf@subtitleaboveskip@length=\skip101
\mdf@subtitlebelowskip@length=\skip102
\mdf@subtitleinneraboveskip@length=\skip103
\mdf@subtitleinnerbelowskip@length=\skip104
\mdf@subsubtitleabovelinewidth@length=\skip105
\mdf@subsubtitlebelowlinewidth@length=\skip106
\mdf@subsubtitleaboveskip@length=\skip107
\mdf@subsubtitlebelowskip@length=\skip108
\mdf@subsubtitleinneraboveskip@length=\skip109
\mdf@subsubtitleinnerbelowskip@length=\skip110
(c:/texlive/2024/texmf-dist/tex/latex/mdframed/md-frame-0.mdf
File: md-frame-0.mdf 2013/07/01\ 1.9b: md-frame-0
)
\mdf@frametitlebox=\box61
\mdf@footnotebox=\box62
\mdf@splitbox@one=\box63
\mdf@splitbox@two=\box64
\mdf@splitbox@save=\box65
\mdfsplitboxwidth=\skip111
\mdfsplitboxtotalwidth=\skip112
\mdfsplitboxheight=\skip113
\mdfsplitboxdepth=\skip114
\mdfsplitboxtotalheight=\skip115
\mdfframetitleboxwidth=\skip116
\mdfframetitleboxtotalwidth=\skip117
\mdfframetitleboxheight=\skip118
\mdfframetitleboxdepth=\skip119
\mdfframetitleboxtotalheight=\skip120
\mdffootnoteboxwidth=\skip121
\mdffootnoteboxtotalwidth=\skip122
\mdffootnoteboxheight=\skip123
\mdffootnoteboxdepth=\skip124
\mdffootnoteboxtotalheight=\skip125
\mdftotallinewidth=\skip126
\mdfboundingboxwidth=\skip127
\mdfboundingboxtotalwidth=\skip128
\mdfboundingboxheight=\skip129
\mdfboundingboxdepth=\skip130
\mdfboundingboxtotalheight=\skip131
```

```
\mdf@freevspace@length=\skip132
\mdf@horizontalwidthofbox@length=\skip133
\mdf@verticalmarginwhole@length=\skip134
\mdf@horizontalspaceofbox=\skip135
\mdfsubtitleheight=\skip136
\mdfsubsubtitleheight=\skip137
\c@mdfcountframes=\count278

****** mdframed patching \endmdf@trivlist

****** -- success******

\mdf@envdepth=\count279
\c@mdf@env@i=\count280
\c@mdf@env@ii=\count281
\c@mdf@zref@counter=\count282
Package zref Info: New property: mdf@pagevalue on input line 895.
) (c:/texlive/2024/texmf-dist/tex/latex/titlesec/titlesec.sty
Package: titlesec 2023/10/27 v2.16 Sectioning titles
\ttl@box=\box66
\beforetitleunit=\skip138
\aftertitleunit=\skip139
\ttl@plus=\dimen154
\ttl@minus=\dimen155
\ttl@toksa=\toks34
\titlewidth=\dimen156
\titlewidthlast=\dimen157
\titlewidthfirst=\dimen158
) (c:/texlive/2024/texmf-dist/tex/latex/koma-script/scrextend.sty
Package: scrextend 2023/07/07 v3.41 KOMA-Script package (extend other
classes w
ith features of KOMA-Script classes)
(c:/texlive/2024/texmf-dist/tex/latex/koma-script/scrkbase.sty
Package: scrkbase 2023/07/07 v3.41 KOMA-Script package (KOMA-Script-
dependent b
asics and keyval usage)
(c:/texlive/2024/texmf-dist/tex/latex/koma-script/scrbase.sty
Package: scrbase 2023/07/07 v3.41 KOMA-Script package (KOMA-Script-
independent
basics and keyval usage)
(c:/texlive/2024/texmf-dist/tex/latex/koma-script/scrlfile.sty
Package: scrlfile 2023/07/07 v3.41 KOMA-Script package (file load hooks)
(c:/texlive/2024/texmf-dist/tex/latex/koma-script/scrlfile-hook.sty
Package: scrlfile-hook 2023/07/07 v3.41 KOMA-Script package (using LaTeX
hooks)

(c:/texlive/2024/texmf-dist/tex/latex/koma-script/scrlogo.sty
Package: scrlogo 2023/07/07 v3.41 KOMA-Script package (logo)
)))
Applying: [2021/05/01] Usage of raw or classic option list on input line
252.
Already applied: [0000/00/00] Usage of raw or classic option list on
input line
 368.
```

```
))
Package scrextend Info: unexpected definition of `\@makefnmark'.
(scrextend)             Trying to patch it on input line 1762.
Package scrextend Info: patch seems to be successfull on input line 1762.
)

LaTeX Font Warning: Font shape `T1/cmr/m/n' in size <7.5> not available
(Font)              size <7> substituted on input line 69.

(c:/texlive/2024/texmf-dist/tex/latex/tools/calc.sty
Package: calc 2023/07/08 v4.3 Infix arithmetic (KKT,FJ)
\calc@Acount=\count283
\calc@Bcount=\count284
\calc@Adimen=\dimen159
\calc@Bdimen=\dimen160
\calc@Askip=\skip140
\calc@Bskip=\skip141
LaTeX Info: Redefining \setlength on input line 80.
LaTeX Info: Redefining \addtolength on input line 81.
\calc@Ccount=\count285
\calc@Cskip=\skip142
) (c:/texlive/2024/texmf-dist/tex/latex/geometry/geometry.sty
Package: geometry 2020/01/02 v5.9 Page Geometry
(c:/texlive/2024/texmf-dist/tex/generic/iftex/ifvtex.sty
Package: ifvtex 2019/10/25 v1.7 ifvtex legacy package. Use iftex instead.
)
\Gm@cnth=\count286
\Gm@cntv=\count287
\c@Gm@tempcnt=\count288
\Gm@bindingoffset=\dimen161
\Gm@wd@mp=\dimen162
\Gm@odd@mp=\dimen163
\Gm@even@mp=\dimen164
\Gm@layoutwidth=\dimen165
\Gm@layoutheight=\dimen166
\Gm@layouthoffset=\dimen167
\Gm@layoutvoffset=\dimen168
\Gm@dimlist=\toks35
) (c:/texlive/2024/texmf-dist/tex/latex/preprint/authblk.sty
Package: authblk 2001/02/27 1.3 (PWD)
\affilsep=\skip143
\@affilsep=\skip144
\c@Maxaffil=\count289
\c@authors=\count290
\c@affil=\count291
) (c:/texlive/2024/texmf-dist/tex/latex/footmisc/footmisc.sty
Package: footmisc 2023/07/05 v6.0f a miscellany of footnote facilities
\FN@temptoken=\toks36
\footnotemargin=\dimen169
\@outputbox@depth=\dimen170
Package footmisc Info: Declaring symbol style bringhurst on input line
696.
Package footmisc Info: Declaring symbol style chicago on input line 704.
Package footmisc Info: Declaring symbol style wiley on input line 713.
```

```
Package footmisc Info: Declaring symbol style lamport-robust on input
line 724.

Package footmisc Info: Declaring symbol style lamport* on input line 744.
Package footmisc Info: Declaring symbol style lamport*-robust on input
line 765
.
) (c:/texlive/2024/texmf-dist/tex/latex/fancyhdr/fancyhdr.sty
Package: fancyhdr 2024/07/23 v4.3.1 Extensive control of page headers and
foote
rs
\f@nch@headwidth=\skip145
\f@nch@O@elh=\skip146
\f@nch@O@erh=\skip147
\f@nch@O@olh=\skip148
\f@nch@O@orh=\skip149
\f@nch@O@elf=\skip150
\f@nch@O@erf=\skip151
\f@nch@O@olf=\skip152
\f@nch@O@orf=\skip153
) (c:/texlive/2024/texmf-dist/tex/generic/alphalph/alphalph.sty
Package: alphalph 2019/12/09 v2.6 Convert numbers to letters (HO)
(c:/texlive/2024/texmf-dist/tex/generic/intcalc/intcalc.sty
Package: intcalc 2019/12/15 v1.3 Expandable calculations with integers
(HO)
))
\c@authorfn=\count292
(c:/texlive/2024/texmf-dist/tex/latex/abstract/abstract.sty
Package: abstract 2009/06/08 v1.2a configurable abstracts
\abstitleskip=\skip154
\absleftindent=\skip155
\absrightindent=\skip156
\absparindent=\skip157
\absparsep=\skip158
)
Package newfloat Info: New float `keypoints' with options
`placement=t!,name=kp
t' on input line 274.
\c@keypoints=\count293
\newfloat@ftype=\count294
Package newfloat Info: float type `keypoints'=8 on input line 274.
(c:/texlive/2024/texmf-dist/tex/latex/enumitem/enumitem.sty
Package: enumitem 2019/06/20 v3.9 Customized lists
\labelindent=\skip159
\enit@outerparindent=\dimen171
\enit@toks=\toks37
\enit@inbox=\box67
\enit@count@id=\count295
\enitdp@description=\count296
) (c:/texlive/2024/texmf-dist/tex/latex/quoting/quoting.sty
Package: quoting 2014/01/28 v0.1c Consolidated environment for displayed
text
\quo@toppartop=\skip160
) (c:/texlive/2024/texmf-dist/tex/latex/sttools/stfloats.sty
```

```
Package: stfloats 2017/03/27 v3.3 Improve float mechanism and
baselineskip sett
ings
\@dblbotnum=\count297
\c@dblbotnumber=\count298
) (c:/texlive/2024/texmf-dist/tex/latex/booktabs/booktabs.sty
Package: booktabs 2020/01/12 v1.61803398 Publication quality tables
\heavyrulewidth=\dimen172
\lightrulewidth=\dimen173
\cmidrulewidth=\dimen174
\belowrulesep=\dimen175
\belowbottomsep=\dimen176
\aboverulesep=\dimen177
\abovetopsep=\dimen178
\cmidrulesep=\dimen179
\cmidrulekern=\dimen180
\defaultaddspace=\dimen181
\@cmidla=\count299
\@cmidlb=\count300
\@aboverulesep=\dimen182
\@belowrulesep=\dimen183
\@thisruleclass=\count301
\@lastruleclass=\count302
\@thisrulewidth=\dimen184
) (c:/texlive/2024/texmf-dist/tex/latex/tools/tabularx.sty
Package: tabularx 2023/12/11 v2.12a `tabularx' package (DPC)
\TX@col@width=\dimen185
\TX@old@table=\dimen186
\TX@old@col=\dimen187
\TX@target=\dimen188
\TX@delta=\dimen189
\TX@cols=\count303
\TX@ftn=\toks38
)
\enitdp@tablenotes=\count304
(c:/texlive/2024/texmf-dist/tex/latex/caption/caption.sty
Package: caption 2023/08/05 v3.6o Customizing captions (AR)
(c:/texlive/2024/texmf-dist/tex/latex/caption/caption3.sty
Package: caption3 2023/07/31 v2.4d caption3 kernel (AR)
\caption@tempdima=\dimen190
\captionmargin=\dimen191
\caption@leftmargin=\dimen192
\caption@rightmargin=\dimen193
\caption@width=\dimen194
\caption@indent=\dimen195
\caption@parindent=\dimen196
\caption@hangindent=\dimen197
Package caption Info: Standard document class detected.
)
\c@caption@flags=\count305
\c@continuedfloat=\count306
Package caption Info: rotating package is loaded.
Package caption Info: scrextend package is loaded.
\caption@addmargin@hsize=\dimen198
```

```
\caption@addmargin@linewidth=\dimen199
) (c:/texlive/2024/texmf-dist/tex/latex/natbib/natbib.sty
Package: natbib 2010/09/13 8.31b (PWD, AO)
\bibhang=\skip161
\bibsep=\skip162
LaTeX Info: Redefining \cite on input line 694.
\c@NAT@ctr=\count307
)) (c:/texlive/2024/texmf-dist/tex/latex/siunitx/siunitx.sty
Package: siunitx 2024-06-24 v3.3.19 A comprehensive (SI) units package
\l__siunitx_number_uncert_offset_int=\count308
\l__siunitx_number_exponent_fixed_int=\count309
\l__siunitx_number_min_decimal_int=\count310
\l__siunitx_number_min_integer_int=\count311
\l__siunitx_number_round_precision_int=\count312
\l__siunitx_number_lower_threshold_int=\count313
\l__siunitx_number_upper_threshold_int=\count314
\l__siunitx_number_group_first_int=\count315
\l__siunitx_number_group_size_int=\count316
\l__siunitx_number_group_minimum_int=\count317
\l__siunitx_angle_tmp_dim=\dimen256
\l__siunitx_angle_marker_box=\box68
\l__siunitx_angle_unit_box=\box69
\l__siunitx_compound_count_int=\count318
(c:/texlive/2024/texmf-dist/tex/latex/translations/translations.sty
Package: translations 2022/02/05 v1.12 internationalization of LaTeX2e
packages
 (CN)
) (c:/texlive/2024/texmf-dist/tex/latex/amsmath/amstext.sty
Package: amstext 2021/08/26 v2.01 AMS text
(c:/texlive/2024/texmf-dist/tex/latex/amsmath/amsgen.sty
File: amsgen.sty 1999/11/30 v2.0 generic functions
\@emptytoks=\toks39
\ex@=\dimen257
))
\l__siunitx_table_tmp_box=\box70
\l__siunitx_table_tmp_dim=\dimen258
\l__siunitx_table_column_width_dim=\dimen259
\l__siunitx_table_integer_box=\box71
\l__siunitx_table_decimal_box=\box72
\l__siunitx_table_uncert_box=\box73
\l__siunitx_table_before_box=\box74
\l__siunitx_table_after_box=\box75
\l__siunitx_table_before_dim=\dimen260
\l__siunitx_table_carry_dim=\dimen261
\l__siunitx_unit_tmp_int=\count319
\l__siunitx_unit_position_int=\count320
\l__siunitx_unit_total_int=\count321
) (c:/texlive/2024/texmf-dist/tex/latex/makecell/makecell.sty
Package: makecell 2009/08/03 V0.1e Managing of Tab Column Heads and Cells
\rotheadsize=\dimen262
\c@nlinenum=\count322
\TeXr@lab=\toks40
) (c:/texlive/2024/texmf-dist/tex/latex/chngcntr/chngcntr.sty
Package: chngcntr 2018/04/09 v1.1a change counter resetting
```

```
Package chngcntr Info: \counterwithout already defined.
(chngcntr)             Quitting chngcntr on input line 21.
) (c:/texlive/2024/texmf-dist/tex/latex/amsmath/amsmath.sty
Package: amsmath 2024/05/23 v2.17q AMS math features
\@mathmargin=\skip163
For additional information on amsmath, use the `?' option.
(c:/texlive/2024/texmf-dist/tex/latex/amsmath/amsbsy.sty
Package: amsbsy 1999/11/29 v1.2d Bold Symbols
\pmbraise@=\dimen263
) (c:/texlive/2024/texmf-dist/tex/latex/amsmath/amsopn.sty
Package: amsopn 2022/04/08 v2.04 operator names
)
\inf@bad=\count323
LaTeX Info: Redefining \frac on input line 233.
\uproot@=\count324
\leftroot@=\count325
LaTeX Info: Redefining \overline on input line 398.
LaTeX Info: Redefining \colon on input line 409.
\classnum@=\count326
\DOTSCASE@=\count327
LaTeX Info: Redefining \ldots on input line 495.
LaTeX Info: Redefining \dots on input line 498.
LaTeX Info: Redefining \cdots on input line 619.
\Mathstrutbox@=\box76
\strutbox@=\box77
LaTeX Info: Redefining \big on input line 721.
LaTeX Info: Redefining \Big on input line 722.
LaTeX Info: Redefining \bigg on input line 723.
LaTeX Info: Redefining \Bigg on input line 724.
\big@size=\dimen264
LaTeX Font Info:    Redeclaring font encoding OML on input line 742.
LaTeX Font Info:    Redeclaring font encoding OMS on input line 743.
\macc@depth=\count328
LaTeX Info: Redefining \bmod on input line 904.
LaTeX Info: Redefining \pmod on input line 909.
LaTeX Info: Redefining \smash on input line 939.
LaTeX Info: Redefining \relbar on input line 969.
LaTeX Info: Redefining \Relbar on input line 970.
\c@MaxMatrixCols=\count329
\dotsspace@=\muskip21
\c@parentequation=\count330
\dspbrk@lvl=\count331
\tag@help=\toks41
\row@=\count332
\column@=\count333
\maxfields@=\count334
\andhelp@=\toks42
\eqnshift@=\dimen265
\alignsep@=\dimen266
\tagshift@=\dimen267
\tagwidth@=\dimen268
\totwidth@=\dimen269
\lineht@=\dimen270
\@envbody=\toks43
```

```
\multlinegap=\skip164
\multlinetaggap=\skip165
\mathdisplay@stack=\toks44
LaTeX Info: Redefining \[ on input line 2953.
LaTeX Info: Redefining \] on input line 2954.
) (c:/texlive/2024/texmf-dist/tex/latex/amsfonts/amsfonts.sty
Package: amsfonts 2013/01/14 v3.01 Basic AMSFonts support
\symAMSa=\mathgroup10
\symAMSb=\mathgroup11
LaTeX Font Info:    Redeclaring math symbol \hbar on input line 98.
LaTeX Info: Redefining \frak on input line 111.
) (c:/texlive/2024/texmf-dist/tex/latex/hyperref/hyperref.sty
Package: hyperref 2024-07-10 v7.01j Hypertext links for LaTeX
(c:/texlive/2024/texmf-dist/tex/generic/pdfescape/pdfescape.sty
Package: pdfescape 2019/12/09 v1.15 Implements pdfTeX's escape features
(HO)
) (c:/texlive/2024/texmf-dist/tex/latex/hycolor/hycolor.sty
Package: hycolor 2020-01-27 v1.10 Color options for hyperref/bookmark
(HO)
) (c:/texlive/2024/texmf-dist/tex/latex/hyperref/nameref.sty
Package: nameref 2023-11-26 v2.56 Cross-referencing by name of section
(c:/texlive/2024/texmf-dist/tex/latex/refcount/refcount.sty
Package: refcount 2019/12/15 v3.6 Data extraction from label references
(HO)
) (c:/texlive/2024/texmf-
dist/tex/generic/gettitlestring/gettitlestring.sty
Package: gettitlestring 2019/12/15 v1.6 Cleanup title references (HO)
)
\c@section@level=\count335
) (c:/texlive/2024/texmf-dist/tex/generic/stringenc/stringenc.sty
Package: stringenc 2019/11/29 v1.12 Convert strings between diff.
encodings (HO
)
)
\@linkdim=\dimen271
\Hy@linkcounter=\count336
\Hy@pagecounter=\count337
(c:/texlive/2024/texmf-dist/tex/latex/hyperref/pd1enc.def
File: pd1enc.def 2024-07-10 v7.01j Hyperref: PDFDocEncoding definition
(HO)
Now handling font encoding PD1 ...
... no UTF-8 mapping file for font encoding PD1
)
\Hy@SavedSpaceFactor=\count338
(c:/texlive/2024/texmf-dist/tex/latex/hyperref/puenc.def
File: puenc.def 2024-07-10 v7.01j Hyperref: PDF Unicode definition (HO)
Now handling font encoding PU ...
... no UTF-8 mapping file for font encoding PU
)
Package hyperref Info: Option `colorlinks' set `true' on input line 4040.
Package hyperref Info: Hyper figures OFF on input line 4157.
Package hyperref Info: Link nesting OFF on input line 4162.
Package hyperref Info: Hyper index ON on input line 4165.
Package hyperref Info: Plain pages OFF on input line 4172.
```

```
Package hyperref Info: Backreferencing OFF on input line 4177.
Package hyperref Info: Implicit mode ON; LaTeX internals redefined.
Package hyperref Info: Bookmarks ON on input line 4424.
\c@Hy@tempcnt=\count339
LaTeX Info: Redefining \url on input line 4763.
\XeTeXLinkMargin=\dimen272
(c:/texlive/2024/texmf-dist/tex/generic/bitset/bitset.sty
Package: bitset 2019/12/09 v1.3 Handle bit-vector datatype (HO)
(c:/texlive/2024/texmf-dist/tex/generic/bigintcalc/bigintcalc.sty
Package: bigintcalc 2019/12/15 v1.5 Expandable calculations on big
integers (HO
)
))
\Fld@menulength=\count340
\Field@Width=\dimen273
\Fld@charsize=\dimen274
Package hyperref Info: Hyper figures OFF on input line 6042.
Package hyperref Info: Link nesting OFF on input line 6047.
Package hyperref Info: Hyper index ON on input line 6050.
Package hyperref Info: backreferencing OFF on input line 6057.
Package hyperref Info: Link coloring ON on input line 6060.
Package hyperref Info: Link coloring with OCG OFF on input line 6067.
Package hyperref Info: PDF/A mode OFF on input line 6072.
(c:/texlive/2024/texmf-dist/tex/latex/base/atbegshi-ltx.sty
Package: atbegshi-ltx 2021/01/10 v1.0c Emulation of the original atbegshi
package with kernel methods
)
\Hy@abspage=\count341
\c@Item=\count342
\c@Hfootnote=\count343
)
Package hyperref Info: Driver (autodetected): hpdftex.
(c:/texlive/2024/texmf-dist/tex/latex/hyperref/hpdftex.def
File: hpdftex.def 2024-07-10 v7.01j Hyperref driver for pdfTeX
(c:/texlive/2024/texmf-dist/tex/latex/base/atveryend-ltx.sty
Package: atveryend-ltx 2020/08/19 v1.0a Emulation of the original
atveryend pac
kage
with kernel methods
)
\HyAnn@Count=\count344
\Fld@listcount=\count345
\c@bookmark@seq@number=\count346
(c:/texlive/2024/texmf-dist/tex/latex/rerunfilecheck/rerunfilecheck.sty
Package: rerunfilecheck 2022-07-10 v1.10 Rerun checks for auxiliary files
(HO)
(c:/texlive/2024/texmf-dist/tex/generic/uniquecounter/uniquecounter.sty
Package: uniquecounter 2019/12/15 v1.4 Provide unlimited unique counter
(HO)
)
Package uniquecounter Info: New unique counter `rerunfilecheck' on input
line 2
85.
)
```

```
\Hy@SectionHShift=\skip166
)
Package translations Info: No language package found. I am going to use
`englis
h' as default language. on input line 84.
LaTeX Font Info:    Trying to load font information for T1+Merriwthr-OsF
on inp
ut line 84.
(c:/texlive/2024/texmf-dist/tex/latex/merriweather/T1Merriwthr-OsF.fd
File: T1Merriwthr-OsF.fd 2020/08/30 (autoinst) Font definitions for
T1/Merriwth
r-OsF.
)
LaTeX Font Info:    Font shape `T1/Merriwthr-OsF/m/n' will be
(Font)              scaled to size 7.5pt on input line 84.
(./main.aux)
\openout1 = `main.aux'.

LaTeX Font Info:    Checking defaults for OML/cmm/m/it on input line 84.
LaTeX Font Info:    ... okay on input line 84.
LaTeX Font Info:    Checking defaults for OMS/cmsy/m/n on input line 84.
LaTeX Font Info:    ... okay on input line 84.
LaTeX Font Info:    Checking defaults for OT1/cmr/m/n on input line 84.
LaTeX Font Info:    ... okay on input line 84.
LaTeX Font Info:    Checking defaults for T1/cmr/m/n on input line 84.
LaTeX Font Info:    ... okay on input line 84.
LaTeX Font Info:    Checking defaults for TS1/cmr/m/n on input line 84.
LaTeX Font Info:    ... okay on input line 84.
LaTeX Font Info:    Checking defaults for OMX/cmex/m/n on input line 84.
LaTeX Font Info:    ... okay on input line 84.
LaTeX Font Info:    Checking defaults for U/cmr/m/n on input line 84.
LaTeX Font Info:    ... okay on input line 84.
LaTeX Font Info:    Checking defaults for PD1/pdf/m/n on input line 84.
LaTeX Font Info:    ... okay on input line 84.
LaTeX Font Info:    Checking defaults for PU/pdf/m/n on input line 84.
LaTeX Font Info:    ... okay on input line 84.
LaTeX Info: Redefining \microtypecontext on input line 84.
Package microtype Info: Applying patch `item' on input line 84.
Package microtype Info: Applying patch `toc' on input line 84.
Package microtype Info: Applying patch `eqnum' on input line 84.
Package microtype Info: Applying patch `footnote' on input line 84.
Package microtype Info: Applying patch `verbatim' on input line 84.
Package microtype Info: Generating PDF output.
Package microtype Info: Character protrusion enabled (level 2).
Package microtype Info: Using default protrusion set `alltext'.
Package microtype Info: Automatic font expansion enabled (level 2),
(microtype)             stretch: 20, shrink: 20, step: 1, non-selected.
Package microtype Info: Using default expansion set `alltext-nott'.
LaTeX Info: Redefining \showhyphens on input line 84.
Package microtype Info: No adjustment of tracking.
Package microtype Info: No adjustment of interword spacing.
Package microtype Info: No adjustment of character kerning.
Package microtype Info: Loading generic protrusion settings for font
family
```

```
(microtype)                `Merriwthr-OsF' (encoding: T1).
(microtype)                For optimal results, create family-specific
settings.
(microtype)                See the microtype manual for details.
LaTeX Font Info:    Redeclaring symbol font `operators' on input line 84.
LaTeX Font Info:    Encoding `OT1' has changed to `T1' for symbol font
(Font)              `operators' in the math version `normal' on input
line 84.
LaTeX Font Info:    Overwriting symbol font `operators' in version
`normal'
(Font)                  OT1/cmr/m/n --> T1/Merriwthr-OsF/m/up on input
line 84.

LaTeX Font Info:    Encoding `OT1' has changed to `T1' for symbol font
(Font)              `operators' in the math version `bold' on input line
84.
LaTeX Font Info:    Overwriting symbol font `operators' in version `bold'
(Font)                  OT1/cmr/bx/n --> T1/Merriwthr-OsF/m/up on input
line 84
.
LaTeX Font Info:    Overwriting symbol font `operators' in version `bold'
(Font)                  T1/Merriwthr-OsF/m/up --> T1/Merriwthr-OsF/b/up
on inpu
t line 84.
LaTeX Font Info:    Redeclaring math alphabet \mathbf on input line 84.
LaTeX Font Info:    Overwriting math alphabet `\mathbf' in version
`normal'
(Font)                  OT1/cmr/bx/n --> T1/Merriwthr-OsF/b/up on input
line 84
.
LaTeX Font Info:    Overwriting math alphabet `\mathbf' in version `bold'
(Font)                  OT1/cmr/bx/n --> T1/Merriwthr-OsF/b/up on input
line 84
.
LaTeX Font Info:    Redeclaring math alphabet \mathsf on input line 84.
LaTeX Font Info:    Overwriting math alphabet `\mathsf' in version
`normal'
(Font)                  OT1/cmss/m/n --> T1/MerriwthrSans-OsF/m/up on
input lin
e 84.
LaTeX Font Info:    Overwriting math alphabet `\mathsf' in version `bold'
(Font)                  OT1/cmss/bx/n --> T1/MerriwthrSans-OsF/m/up on
input li
ne 84.
LaTeX Font Info:    Redeclaring math alphabet \mathit on input line 84.
LaTeX Font Info:    Overwriting math alphabet `\mathit' in version
`normal'
(Font)                  OT1/cmr/m/it --> T1/Merriwthr-OsF/m/it on input
line 84
.
LaTeX Font Info:    Overwriting math alphabet `\mathit' in version `bold'
(Font)                  OT1/cmr/bx/it --> T1/Merriwthr-OsF/m/it on input
line 8
4.
```

```
LaTeX Font Info:    Redeclaring math alphabet \mathtt on input line 84.
LaTeX Font Info:    Overwriting math alphabet `\mathtt' in version
`normal'
(Font)                  OT1/cmtt/m/n --> T1/lmtt/m/up on input line 84.
LaTeX Font Info:    Overwriting math alphabet `\mathtt' in version `bold'
(Font)                  OT1/cmtt/m/n --> T1/lmtt/m/up on input line 84.
LaTeX Font Info:    Overwriting math alphabet `\mathsf' in version `bold'
(Font)                  T1/MerriwthrSans-OsF/m/up --> T1/MerriwthrSans-
OsF/b/up
 on input line 84.
LaTeX Font Info:    Overwriting math alphabet `\mathit' in version `bold'
(Font)                  T1/Merriwthr-OsF/m/it --> T1/Merriwthr-OsF/b/it
on inpu
t line 84.
\c@mv@tabular=\count347
\c@mv@boldtabular=\count348
(c:/texlive/2024/texmf-dist/tex/context/base/mkii/supp-pdf.mkii
[Loading MPS to PDF converter (version 2006.09.02).]
\scratchcounter=\count349
\scratchdimen=\dimen275
\scratchbox=\box78
\nofMPsegments=\count350
\nofMParguments=\count351
\everyMPshowfont=\toks45
\MPscratchCnt=\count352
\MPscratchDim=\dimen276
\MPnumerator=\count353
\makeMPintoPDFobject=\count354
\everyMPtoPDFconversion=\toks46
) (c:/texlive/2024/texmf-dist/tex/latex/epstopdf-pkg/epstopdf-base.sty
Package: epstopdf-base 2020-01-24 v2.11 Base part for package epstopdf
Package epstopdf-base Info: Redefining graphics rule for `.eps' on input
line 4
85.
(c:/texlive/2024/texmf-dist/tex/latex/latexconfig/epstopdf-sys.cfg
File: epstopdf-sys.cfg 2010/07/13 v1.3 Configuration of (r)epstopdf for
TeX Liv
e
))
*geometry* driver: auto-detecting
*geometry* detected driver: pdftex
*geometry* verbose mode - [ preamble ] result:
* driver: pdftex
* paper: a4paper
* layout: <same size as paper>
* layoutoffset:(h,v)=(0.0pt,0.0pt)
* modes: includefoot twoside
* h-part:(L,W,R)=(54.64pt, 488.22787pt, 54.64pt)
* v-part:(T,H,B)=(66.0pt, 745.04684pt, 34.0pt)
* \paperwidth=597.50787pt
* \paperheight=845.04684pt
* \textwidth=488.22787pt
* \textheight=715.04684pt
* \oddsidemargin=-17.62999pt
```

```
* \evensidemargin=-17.62999pt
* \topmargin=-47.76999pt
* \headheight=17.5pt
* \headsep=24.0pt
* \topskip=10.0pt
* \footskip=30.0pt
* \marginparwidth=48.0pt
* \marginparsep=10.0pt
* \columnsep=18.0pt
* \skip\footins=22.0pt plus 2.0pt
* \hoffset=0.0pt
* \voffset=0.0pt
* \mag=1000
* \@twocolumntrue
* \@twosidetrue
* \@mparswitchtrue
* \@reversemarginfalse
* (1in=72.27pt=25.4mm, 1cm=28.453pt)

Package caption Info: Begin \AtBeginDocument code.
Package caption Info: hyperref package is loaded.
Package caption Info: End \AtBeginDocument code.

(c:/texlive/2024/texmf-dist/tex/latex/translations/translations-basic-
dictionar
y-english.trsl
File: translations-basic-dictionary-english.trsl (english translation
file `tra
nslations-basic-dictionary')
)
Package translations Info: loading dictionary `translations-basic-
dictionary' f
or `english'. on input line 84.
Package hyperref Info: Link coloring ON on input line 84.
(./main.out) (./main.out)
\@outlinefile=\write3
\openout3 = `main.out'.

\@gscitedetails=\box79
\@gscitedetailsheight=\skip167
\@gsheadbox=\box80
\@gsheadboxheight=\skip168
LaTeX Font Info:    Font shape `T1/Merriwthr-OsF/b/n' will be
(Font)              scaled to size 6.5pt on input line 84.
LaTeX Font Info:    Calculating math sizes for size <7.5> on input line
84.

LaTeX Font Warning: Font shape `T1/Merriwthr-OsF/m/up' undefined
(Font)              using `T1/Merriwthr-OsF/m/n' instead on input line
84.

LaTeX Font Info:    Font shape `T1/Merriwthr-OsF/m/up' will be
(Font)              scaled to size 6.24973pt on input line 84.
LaTeX Font Info:    Font shape `T1/Merriwthr-OsF/m/up' will be
```

```
(Font)              scaled to size 5.24997pt on input line 84.
LaTeX Font Info:    Trying to load font information for U+eur on input
line 84.

(c:/texlive/2024/texmf-dist/tex/latex/amsfonts/ueur.fd
File: ueur.fd 2013/01/14 v3.01 Euler Roman
) (c:/texlive/2024/texmf-dist/tex/latex/microtype/mt-eur.cfg
File: mt-eur.cfg 2006/07/31 v1.1 microtype config. file: AMS Euler Roman
(RS)
)

LaTeX Font Warning: Font shape `OMS/cmsy/m/n' in size <7.5> not available
(Font)              size <7> substituted on input line 84.

LaTeX Font Info:    Trying to load font information for U+euf on input
line 84.

(c:/texlive/2024/texmf-dist/tex/latex/amsfonts/ueuf.fd
File: ueuf.fd 2013/01/14 v3.01 Euler Fraktur
) (c:/texlive/2024/texmf-dist/tex/latex/microtype/mt-euf.cfg
File: mt-euf.cfg 2006/07/03 v1.1 microtype config. file: AMS Euler
Fraktur (RS)

)
LaTeX Font Info:    Trying to load font information for U+eus on input
line 84.

(c:/texlive/2024/texmf-dist/tex/latex/amsfonts/ueus.fd
File: ueus.fd 2013/01/14 v3.01 Euler Script
) (c:/texlive/2024/texmf-dist/tex/latex/microtype/mt-eus.cfg
File: mt-eus.cfg 2006/07/28 v1.2 microtype config. file: AMS Euler Script
(RS)
)
LaTeX Font Info:    Trying to load font information for U+euex on input
line 84
.
(c:/texlive/2024/texmf-dist/tex/latex/amsfonts/ueuex.fd
File: ueuex.fd 2013/01/14 v3.01 Euler extra symbols
)

LaTeX Font Warning: Font shape `OML/cmm/m/it' in size <7.5> not available
(Font)              size <7> substituted on input line 84.

LaTeX Font Info:    Font shape `T1/Merriwthr-OsF/m/n' will be
(Font)              scaled to size 6.24973pt on input line 84.
LaTeX Font Info:    Font shape `T1/Merriwthr-OsF/m/n' will be
(Font)              scaled to size 5.24997pt on input line 84.
LaTeX Font Info:    Font shape `T1/Merriwthr-OsF/m/it' will be
(Font)              scaled to size 7.5pt on input line 84.
LaTeX Font Info:    Font shape `T1/Merriwthr-OsF/m/it' will be
(Font)              scaled to size 6.24973pt on input line 84.
LaTeX Font Info:    Font shape `T1/Merriwthr-OsF/m/it' will be
(Font)              scaled to size 5.24997pt on input line 84.
```

```
LaTeX Font Info:    Trying to load font information for U+msa on input
line 84.

(c:/texlive/2024/texmf-dist/tex/latex/amsfonts/umsa.fd
File: umsa.fd 2013/01/14 v3.01 AMS symbols A
) (c:/texlive/2024/texmf-dist/tex/latex/microtype/mt-msa.cfg
File: mt-msa.cfg 2006/02/04 v1.1 microtype config. file: AMS symbols (a)
(RS)
)
LaTeX Font Info:    Trying to load font information for U+msb on input
line 84.

(c:/texlive/2024/texmf-dist/tex/latex/amsfonts/umsb.fd
File: umsb.fd 2013/01/14 v3.01 AMS symbols B
) (c:/texlive/2024/texmf-dist/tex/latex/microtype/mt-msb.cfg
File: mt-msb.cfg 2005/06/01 v1.0 microtype config. file: AMS symbols (b)
(RS)
)
LaTeX Font Info:    Font shape `T1/Merriwthr-OsF/m/n' will be
(Font)              scaled to size 8.0pt on input line 84.
LaTeX Font Info:    Font shape `T1/Merriwthr-OsF/m/it' will be
(Font)              scaled to size 8.0pt on input line 84.
LaTeX Font Info:    Font shape `T1/Merriwthr-OsF/b/it' will be
(Font)              scaled to size 8.0pt on input line 84.
LaTeX Font Info:    Font shape `T1/Merriwthr-OsF/b/n' will be
(Font)              scaled to size 8.0pt on input line 84.

LaTeX Warning: Reference `LastPage' on page 1 undefined on input line 84.

TextBlockOrigin set to 4pc+6.64pt x 4pc+6pt
LaTeX Font Info:    Font shape `T1/Merriwthr-OsF/m/n' will be
(Font)              scaled to size 14.0pt on input line 100.
LaTeX Font Info:    Font shape `T1/Merriwthr-OsF/m/n' will be
(Font)              scaled to size 8.99997pt on input line 100.
LaTeX Font Info:    Calculating math sizes for size <14> on input line
100.
LaTeX Font Info:    Font shape `T1/Merriwthr-OsF/m/up' will be
(Font)              scaled to size 14.0pt on input line 100.
LaTeX Font Info:    Font shape `T1/Merriwthr-OsF/m/up' will be
(Font)              scaled to size 11.66617pt on input line 100.
LaTeX Font Info:    Font shape `T1/Merriwthr-OsF/m/up' will be
(Font)              scaled to size 9.79996pt on input line 100.
LaTeX Font Info:    Font shape `T1/Merriwthr-OsF/m/n' will be
(Font)              scaled to size 11.66617pt on input line 100.
LaTeX Font Info:    Font shape `T1/Merriwthr-OsF/m/n' will be
(Font)              scaled to size 9.79996pt on input line 100.
LaTeX Font Info:    Font shape `T1/Merriwthr-OsF/m/it' will be
(Font)              scaled to size 14.0pt on input line 100.
LaTeX Font Info:    Font shape `T1/Merriwthr-OsF/m/it' will be
(Font)              scaled to size 11.66617pt on input line 100.
LaTeX Font Info:    Font shape `T1/Merriwthr-OsF/m/it' will be
(Font)              scaled to size 9.79996pt on input line 100.
LaTeX Font Info:    Font shape `T1/Merriwthr-OsF/b/n' will be
(Font)              scaled to size 18.0pt on input line 100.
```

```
LaTeX Font Info:    Font shape `T1/Merriwthr-OsF/m/n' will be
(Font)              scaled to size 13.0pt on input line 100.
LaTeX Font Info:    Calculating math sizes for size <13> on input line
100.
LaTeX Font Info:    Font shape `T1/Merriwthr-OsF/m/up' will be
(Font)              scaled to size 13.0pt on input line 100.
LaTeX Font Info:    Font shape `T1/Merriwthr-OsF/m/up' will be
(Font)              scaled to size 10.83287pt on input line 100.
LaTeX Font Info:    Font shape `T1/Merriwthr-OsF/m/up' will be
(Font)              scaled to size 9.09996pt on input line 100.

LaTeX Font Warning: Font shape `OMS/cmsy/m/n' in size <13> not available
(Font)              size <12> substituted on input line 100.


LaTeX Font Warning: Font shape `OML/cmm/m/it' in size <13> not available
(Font)              size <12> substituted on input line 100.

LaTeX Font Info:    Font shape `T1/Merriwthr-OsF/m/n' will be
(Font)              scaled to size 10.83287pt on input line 100.
LaTeX Font Info:    Font shape `T1/Merriwthr-OsF/m/n' will be
(Font)              scaled to size 9.09996pt on input line 100.
LaTeX Font Info:    Font shape `T1/Merriwthr-OsF/m/it' will be
(Font)              scaled to size 13.0pt on input line 100.
LaTeX Font Info:    Font shape `T1/Merriwthr-OsF/m/it' will be
(Font)              scaled to size 10.83287pt on input line 100.
LaTeX Font Info:    Font shape `T1/Merriwthr-OsF/m/it' will be
(Font)              scaled to size 9.09996pt on input line 100.
LaTeX Font Info:    Trying to load font information for TS1+Merriwthr-OsF
on in
put line 100.
(c:/texlive/2024/texmf-dist/tex/latex/merriweather/TS1Merriwthr-OsF.fd
File: TS1Merriwthr-OsF.fd 2020/08/30 (autoinst) Font definitions for
TS1/Merriw
thr-OsF.
)
LaTeX Font Info:    Font shape `TS1/Merriwthr-OsF/m/n' will be
(Font)              scaled to size 10.83287pt on input line 100.
Package microtype Info: Loading generic protrusion settings for font
family
(microtype)             `Merriwthr-OsF' (encoding: TS1).
(microtype)             For optimal results, create family-specific
settings.
(microtype)             See the microtype manual for details.
LaTeX Font Info:    Font shape `T1/Merriwthr-OsF/m/n' will be
(Font)              scaled to size 9.0pt on input line 100.
LaTeX Font Info:    Font shape `T1/Merriwthr-OsF/m/up' will be
(Font)              scaled to size 9.0pt on input line 100.
LaTeX Font Info:    Font shape `T1/Merriwthr-OsF/m/up' will be
(Font)              scaled to size 7.0pt on input line 100.
LaTeX Font Info:    Font shape `T1/Merriwthr-OsF/m/up' will be
(Font)              scaled to size 5.0pt on input line 100.
LaTeX Font Info:    Font shape `T1/Merriwthr-OsF/m/n' will be
(Font)              scaled to size 7.0pt on input line 100.
```

```
LaTeX Font Info:    Font shape `T1/Merriwthr-OsF/m/n' will be
(Font)              scaled to size 5.0pt on input line 100.
LaTeX Font Info:    Font shape `T1/Merriwthr-OsF/m/it' will be
(Font)              scaled to size 9.0pt on input line 100.
LaTeX Font Info:    Font shape `T1/Merriwthr-OsF/m/it' will be
(Font)              scaled to size 7.0pt on input line 100.
LaTeX Font Info:    Font shape `T1/Merriwthr-OsF/m/it' will be
(Font)              scaled to size 5.0pt on input line 100.
LaTeX Font Info:    Font shape `T1/Merriwthr-OsF/m/n' will be
(Font)              scaled to size 6.5pt on input line 100.
LaTeX Font Info:    Calculating math sizes for size <6.5> on input line
100.
LaTeX Font Info:    Font shape `T1/Merriwthr-OsF/m/up' will be
(Font)              scaled to size 6.5pt on input line 100.
LaTeX Font Info:    Font shape `T1/Merriwthr-OsF/m/up' will be
(Font)              scaled to size 5.41643pt on input line 100.
LaTeX Font Info:    Font shape `T1/Merriwthr-OsF/m/up' will be
(Font)              scaled to size 4.54997pt on input line 100.

LaTeX Font Warning: Font shape `OMS/cmsy/m/n' in size <6.5> not available
(Font)              size <6> substituted on input line 100.


LaTeX Font Warning: Font shape `OMS/cmsy/m/n' in size <5.41643> not
available
(Font)              size <5> substituted on input line 100.


LaTeX Font Warning: Font shape `OMS/cmsy/m/n' in size <4.54997> not
available
(Font)              size <5> substituted on input line 100.


LaTeX Font Warning: Font shape `OML/cmm/m/it' in size <6.5> not available
(Font)              size <6> substituted on input line 100.


LaTeX Font Warning: Font shape `OML/cmm/m/it' in size <5.41643> not
available
(Font)              size <5> substituted on input line 100.


LaTeX Font Warning: Font shape `OML/cmm/m/it' in size <4.54997> not
available
(Font)              size <5> substituted on input line 100.

LaTeX Font Info:    Font shape `T1/Merriwthr-OsF/m/n' will be
(Font)              scaled to size 5.41643pt on input line 100.
LaTeX Font Info:    Font shape `T1/Merriwthr-OsF/m/n' will be
(Font)              scaled to size 4.54997pt on input line 100.
LaTeX Font Info:    Font shape `T1/Merriwthr-OsF/m/it' will be
(Font)              scaled to size 6.5pt on input line 100.
LaTeX Font Info:    Font shape `T1/Merriwthr-OsF/m/it' will be
(Font)              scaled to size 5.41643pt on input line 100.
```

```
LaTeX Font Info:    Font shape `T1/Merriwthr-OsF/m/it' will be
(Font)              scaled to size 4.54997pt on input line 100.
LaTeX Font Info:    Font shape `TS1/Merriwthr-OsF/m/n' will be
(Font)              scaled to size 5.41643pt on input line 100.

Overfull \hbox (54.64pt too wide) in paragraph at lines 100--100
[][][]
 []

LaTeX Font Info:    Font shape `T1/Merriwthr-OsF/b/n' will be
(Font)              scaled to size 10.0pt on input line 100.
LaTeX Font Info:    Trying to load font information for T1+lmtt on input
line 1
00.
(c:/texlive/2024/texmf-dist/tex/latex/lm/t1lmtt.fd
File: t1lmtt.fd 2015/05/01 v1.6.1 Font defs for Latin Modern
)
Package microtype Info: Loading generic protrusion settings for font
family
(microtype)             `lmtt' (encoding: T1).
(microtype)             For optimal results, create family-specific
settings.
(microtype)             See the microtype manual for details.
LaTeX Font Info:    Font shape `T1/Merriwthr-OsF/m/up' will be
(Font)              scaled to size 8.0pt on input line 100.
LaTeX Font Info:    Font shape `T1/Merriwthr-OsF/m/up' will be
(Font)              scaled to size 6.0pt on input line 100.
LaTeX Font Info:    Font shape `T1/Merriwthr-OsF/m/n' will be
(Font)              scaled to size 6.0pt on input line 100.
LaTeX Font Info:    Font shape `T1/Merriwthr-OsF/m/it' will be
(Font)              scaled to size 6.0pt on input line 100.

Overfull \hbox (54.64pt too wide) in paragraph at lines 100--100
[][][]
 []


LaTeX Warning: Text page 1 contains only floats.


Overfull \vbox (8.01685pt too high) has occurred while \output is active
[]




LaTeX Warning: Text page 1 contains only floats.


Overfull \vbox (8.01685pt too high) has occurred while \output is active
[]
```

```
LaTeX Font Info:    Font shape `T1/Merriwthr-OsF/m/n' will be
(Font)              scaled to size 7.8pt on input line 100.
LaTeX Font Info:    Font shape `T1/Merriwthr-OsF/b/n' will be
(Font)              scaled to size 7.8pt on input line 100.
[1{c:/texlive/2024/texmf-
var/fonts/map/pdftex/updmap/pdftex.map}{c:/texlive/202
4/texmf-
dist/fonts/enc/dvips/merriweather/merriwthr_posqbl.enc}{c:/texlive/2024
/texmf-
dist/fonts/enc/dvips/merriweather/merriwthr_owzwzj.enc}{c:/texlive/2024/
texmf-dist/fonts/enc/dvips/lm/lm-ec.enc}


]
LaTeX Font Info:    Font shape `T1/Merriwthr-OsF/b/n' will be
(Font)              scaled to size 7.5pt on input line 104.

Package natbib Warning: Citation `sohn2018present' on page 2 undefined on
input
 line 104.


Package natbib Warning: Citation `zhang2024single' on page 2 undefined on
input
 line 104.


Package natbib Warning: Citation `nurk2022complete' on page 2 undefined
on inpu
t line 104.


Package natbib Warning: Citation `espinosa2024advancements' on page 2
undefined
 on input line 104.


Package natbib Warning: Citation `koren2017canu' on page 2 undefined on
input l
ine 104.


Package natbib Warning: Citation `shafin2020nanopore' on page 2 undefined
on in
put line 104.


Package natbib Warning: Citation `nie2024novo' on page 2 undefined on
input lin
e 104.


Package natbib Warning: Citation `rizzi2019overlap' on page 2 undefined
on inpu
```

```
t line 104.


Package natbib Warning: Citation `altschul1997gapped' on page 2 undefined
on in
put line 106.


Package natbib Warning: Citation `myers2014efficient' on page 2 undefined
on in
put line 106.


Package natbib Warning: Citation `li2018minimap2' on page 2 undefined on
input
line 106.


Package natbib Warning: Citation `xiao2017mecat' on page 2 undefined on
input l
ine 106.


Package natbib Warning: Citation `ruan2020fast' on page 2 undefined on
input li
ne 106.


Package natbib Warning: Citation `nie2024novo' on page 2 undefined on
input lin
e 106.


Package natbib Warning: Citation `kong2025xread' on page 2 undefined on
input l
ine 106.


Package natbib Warning: Citation `vinga2003alignment' on page 2 undefined
on in
put line 108.


Package natbib Warning: Citation `broder1997resemblance' on page 2
undefined on
 input line 112.

LaTeX Font Info:    Font shape `T1/Merriwthr-OsF/m/up' will be
(Font)              scaled to size 7.5pt on input line 112.

Package natbib Warning: Citation `berlin2015assembling' on page 2
undefined on
input line 112.
```

```
Package natbib Warning: Citation `koren2017canu' on page 2 undefined on
input l
ine 112.


Package natbib Warning: Citation `shafin2020nanopore' on page 2 undefined
on in
put line 112.


Package natbib Warning: Citation `firtina2023blend' on page 2 undefined
on inpu
t line 112.


Package natbib Warning: Citation `shafin2020nanopore' on page 2 undefined
on in
put line 114.


Package natbib Warning: Citation `koren2017canu' on page 2 undefined on
input l
ine 114.


Package natbib Warning: Citation `li2016minimap' on page 2 undefined on
input l
ine 114.


Package natbib Warning: Citation `shafin2020nanopore' on page 2 undefined
on in
put line 114.


Package natbib Warning: Citation `greenberg2023analysis' on page 2
undefined on
 input line 114.



LaTeX Font Info:    Font shape `T1/Merriwthr-OsF/b/n' will be
(Font)              scaled to size 8.5pt on input line 121.

LaTeX Warning: File `images/algorithm_overview.png' not found on input
line 126
.


! Package pdftex.def Error: File `images/algorithm_overview.png' not
found: usi
ng draft setting.
```

```
See the pdftex.def package documentation for explanation.
Type  H <return>  for immediate help.
 ...

l.126 ...textwidth]{images/algorithm_overview.png}

Try typing  <return>  to proceed.
If that doesn't work, type  X <return>  to quit.

LaTeX Font Info:    Font shape `T1/Merriwthr-OsF/b/n' will be
(Font)              scaled to size 6.0pt on input line 128.

Package natbib Warning: Citation `charikar2002similarity' on page 2
undefined o
n input line 133.


Package natbib Warning: Citation `ponsero2023comparison' on page 2
undefined on
 input line 133.



[2] [3]
LaTeX Font Info:    Font shape `T1/Merriwthr-OsF/m/sc' will be
(Font)              scaled to size 7.5pt on input line 141.

Package natbib Warning: Citation `miller2008aggressive' on page 4
undefined on
input line 141.


Package natbib Warning: Citation `koren2017canu' on page 4 undefined on
input l
ine 141.


Package natbib Warning: Citation `shafin2020nanopore' on page 4 undefined
on in
put line 141.


LaTeX Warning: File `images/evaluation_pipeline.png' not found on input
line 14
6.


! Package pdftex.def Error: File `images/evaluation_pipeline.png' not
found: us
ing draft setting.

See the pdftex.def package documentation for explanation.
Type  H <return>  for immediate help.
 ...
```

```
l.146 ...extwidth]{images/evaluation_pipeline.png}

Try typing  <return>  to proceed.
If that doesn't work, type  X <return>  to quit.


Package natbib Warning: Citation `zhang2021using' on page 4 undefined on
input
line 153.


LaTeX Warning: File `images/feature_extraction.png' not found on input
line 160
.


! Package pdftex.def Error: File `images/feature_extraction.png' not
found: usi
ng draft setting.

See the pdftex.def package documentation for explanation.
Type  H <return>  for immediate help.
 ...

l.160 ...linewidth]{images/feature_extraction.png}

Try typing  <return>  to proceed.
If that doesn't work, type  X <return>  to quit.


Package natbib Warning: Citation `koren2017canu' on page 4 undefined on
input l
ine 171.



LaTeX Font Info:    Font shape `TS1/Merriwthr-OsF/m/n' will be
(Font)              scaled to size 7.5pt on input line 179.

Package natbib Warning: Citation `dunteman1989principal' on page 4
undefined on
 input line 187.


Package natbib Warning: Citation `mcinnes2018umap' on page 4 undefined on
input
 line 187.


Package natbib Warning: Citation `dong2011efficient' on page 4 undefined
on inp
ut line 187.
```

```
Package natbib Warning: Citation `firtina2023blend' on page 4 undefined on inpu
t line 187.


Package natbib Warning: Citation `li2024dna2bit' on page 4 undefined on input l
ine 187.



[4{c:/texlive/2024/texmf-dist/fonts/enc/dvips/merriweather/merriwthr_xys2i2.enc
}] [5]


LaTeX Warning: File `images/dimenality_reduction_main.png' not found on input l
ine 196.


! Package pdftex.def Error: File `images/dimenality_reduction_main.png' not fou
nd: using draft setting.

See the pdftex.def package documentation for explanation.
Type  H <return>  for immediate help.
 ...

l.196 ...th]{images/dimenality_reduction_main.png}

Try typing  <return>  to proceed.
If that doesn't work, type  X <return>  to quit.


LaTeX Warning: File `images/ann_main.png' not found on input line 210.


! Package pdftex.def Error: File `images/ann_main.png' not found: using draft s
etting.

See the pdftex.def package documentation for explanation.
Type  H <return>  for immediate help.
 ...

l.210 ...cs[width=\linewidth]{images/ann_main.png}

Try typing  <return>  to proceed.
If that doesn't work, type  X <return>  to quit.
```

```
Package natbib Warning: Citation `dong2011efficient' on page 6 undefined
on inp
ut line 220.


Package natbib Warning: Citation `malkov2018efficient' on page 6
undefined on i
nput line 220.


Package natbib Warning: Citation `jegou2010product' on page 6 undefined
on inpu
t line 220.


Package natbib Warning: Citation `jegou2010product' on page 6 undefined
on inpu
t line 220.


Package natbib Warning: Citation `yan2019k' on page 6 undefined on input
line 2
20.


Underfull \vbox (badness 10000) has occurred while \output is active []



LaTeX Font Info:    Font shape `T1/Merriwthr-OsF/m/n' will be
(Font)              scaled to size 8.5pt on input line 224.
[6]
Underfull \hbox (badness 1067) in paragraph at lines 224--224
|\T1/Merriwthr-OsF/b/n/8.5 (+20) Efficient im-ple-men-ta-tion of the IDF-
SRP-NN
Descent
 []


Package natbib Warning: Citation `marccais2011jellyfish' on page 7
undefined on
 input line 230.


Package natbib Warning: Citation `manning2008introduction' on page 7
undefined
on input line 230.


Underfull \vbox (badness 1509) has occurred while \output is active []
```

```
Package natbib Warning: Citation `pynndescent' on page 7 undefined on
input lin
e 232.


LaTeX Warning: File `images/genome_benchmarking.png' not found on input
line 23
9.


! Package pdftex.def Error: File `images/genome_benchmarking.png' not
found: us
ing draft setting.

See the pdftex.def package documentation for explanation.
Type  H <return>  for immediate help.
 ...

l.239 ...extwidth]{images/genome_benchmarking.png}

Try typing  <return>  to proceed.
If that doesn't work, type  X <return>  to quit.


Package natbib Warning: Citation `kong2025xread' on page 7 undefined on
input l
ine 252.



[7] [8]

Package natbib Warning: Citation `shafin2020nanopore' on page 9 undefined
on in
put line 266.




[9]

Package natbib Warning: Citation `miller2008aggressive' on page 10
undefined on
 input line 289.


Package natbib Warning: Citation `shafin2020nanopore' on page 10
undefined on i
nput line 289.
```

```
Package natbib Warning: Citation `zhao2023towards' on page 10 undefined
on inpu
t line 297.


Package natbib Warning: Citation `harris2020array' on page 10 undefined
on inpu
t line 306.


Package natbib Warning: Citation `virtanen2020scipy' on page 10 undefined
on in
put line 306.

LaTeX Font Info:    Font shape `T1/Merriwthr-OsF/b/sl' in size <7.5> not
availa
ble
(Font)              Font shape `T1/Merriwthr-OsF/b/it' tried instead on
input l
ine 308.
LaTeX Font Info:    Font shape `T1/Merriwthr-OsF/b/it' will be
(Font)              scaled to size 7.5pt on input line 308.


[10]
LaTeX Font Info:    Font shape `T1/Merriwthr-OsF/b/n' will be
(Font)              scaled to size 6.24973pt on input line 322.
LaTeX Font Info:    Font shape `T1/Merriwthr-OsF/b/n' will be
(Font)              scaled to size 5.24997pt on input line 322.

Package natbib Warning: Citation `pynndescent' on page 11 undefined on
input li
ne 328.


Package natbib Warning: Citation `shafin2020nanopore' on page 11
undefined on i
nput line 333.




Package natbib Warning: Citation `nurk2022complete' on page 11 undefined
on inp
ut line 350.


Package natbib Warning: Citation `ono2022pbsim3' on page 11 undefined on
input
line 352.


Package natbib Warning: Citation `wright2020november' on page 11
undefined on i
```

```
nput line 352.


Package natbib Warning: Citation `zook2020robust' on page 11 undefined on
input
 line 352.



[11]

Package natbib Warning: Citation `gurevich2013quast' on page 12 undefined
on in
put line 369.


Package natbib Warning: Citation `li2020yak' on page 12 undefined on
input line
 369.


Package natbib Warning: Citation `simao2015busco' on page 12 undefined on
input
 line 369.


Package natbib Warning: Citation `jzhang2023lakeview' on page 12
undefined on i
nput line 369.




Package natbib Warning: Citation `zhang2026fedrann' on page 12 undefined
on inp
ut line 388.

! Misplaced alignment tab character &.
l.396 ...work was supported by the National Key R&
                                                  D Program of China (No.
20...
I can't figure out why you would want to use a tab mark
here. If you just want an ampersand, the remedy is
simple: Just type `I\&' now. But if some right brace
up above has ended a previous alignment prematurely,
you're probably due for more error messages, and you
might try typing `S' now just to see what is salvageable.

! Misplaced alignment tab character &.
l.396 ...00), the "Pioneer" and "Leading Goose" R&
                                                  D Program of Zhejiang
(No....
I can't figure out why you would want to use a tab mark
here. If you just want an ampersand, the remedy is
```

simple: Just type `I\&' now. But if some right brace
up above has ended a previous alignment prematurely,
you're probably due for more error messages, and you
might try typing `S' now just to see what is salvageable.

No file main.bbl.

[12]

[13

]
Underfull \hbox (badness 2922) in paragraph at lines 432--433
[]\T1/Merriwthr-OsF/b/n/7.5 (+20) High-complexity genomes \T1/Merriwthr-OsF/m/u
p/7.5 (+20) (e.g., hu-man, large poly-ploid
 []

[14

]

[15

]

[16

]

LaTeX Warning: File `images/preprocessing_error_rate.png' not found on input li
ne 561.

! Package pdftex.def Error: File `images/preprocessing_error_rate.png' not foun
d: using draft setting.

See the pdftex.def package documentation for explanation.
Type H <return> for immediate help.

```
 ...

l.561 ...dth]{images/preprocessing_error_rate.png}

Try typing  <return>  to proceed.
If that doesn't work, type  X <return>  to quit.

[17]

LaTeX Warning: File `images/preprocessing_box_plot.png' not found on
input line
 570.


! Package pdftex.def Error: File `images/preprocessing_box_plot.png' not
found:
 using draft setting.

See the pdftex.def package documentation for explanation.
Type  H <return>  for immediate help.
 ...

l.570 ...width]{images/preprocessing_box_plot.png}

Try typing  <return>  to proceed.
If that doesn't work, type  X <return>  to quit.

LaTeX Font Info:    Font shape `TS1/Merriwthr-OsF/m/n' will be
(Font)              scaled to size 6.0pt on input line 572.
[18]

LaTeX Warning: File `images/length_depth_accuracy.png' not found on input
line
581.


! Package pdftex.def Error: File `images/length_depth_accuracy.png' not
found:
using draft setting.

See the pdftex.def package documentation for explanation.
Type  H <return>  for immediate help.
 ...

l.581 ...twidth]{images/length_depth_accuracy.png}

Try typing  <return>  to proceed.
If that doesn't work, type  X <return>  to quit.

[19]

LaTeX Warning: File `images/embedding_dimension_error_rate.png' not found
on in
put line 593.
```

```
! Package pdftex.def Error: File
`images/embedding_dimension_error_rate.png' no
t found: using draft setting.

See the pdftex.def package documentation for explanation.
Type  H <return>  for immediate help.
 ...

l.593 ...mages/embedding_dimension_error_rate.png}

Try typing  <return>  to proceed.
If that doesn't work, type  X <return>  to quit.

[20]

LaTeX Warning: File `images/embedding_dimension_detail.png' not found on
input
line 603.


! Package pdftex.def Error: File `images/embedding_dimension_detail.png'
not fo
und: using draft setting.

See the pdftex.def package documentation for explanation.
Type  H <return>  for immediate help.
 ...

l.603 ...h]{images/embedding_dimension_detail.png}

Try typing  <return>  to proceed.
If that doesn't work, type  X <return>  to quit.

[21]

LaTeX Warning: File `images/distance_correlation.png' not found on input
line 6
14.


! Package pdftex.def Error: File `images/distance_correlation.png' not
found: u
sing draft setting.

See the pdftex.def package documentation for explanation.
Type  H <return>  for immediate help.
 ...

l.614 ...xtwidth]{images/distance_correlation.png}

Try typing  <return>  to proceed.
If that doesn't work, type  X <return>  to quit.
```

```
[22]

LaTeX Warning: File `images/fedrann_workflow.png' not found on input line
626.


! Package pdftex.def Error: File `images/fedrann_workflow.png' not found:
using
 draft setting.

See the pdftex.def package documentation for explanation.
Type  H <return>  for immediate help.
 ...

l.626 ...aspectratio]{images/fedrann_workflow.png}

Try typing  <return>  to proceed.
If that doesn't work, type  X <return>  to quit.

[23]

LaTeX Warning: File `images/CF_DF_correlation.png' not found on input
line 637.



! Package pdftex.def Error: File `images/CF_DF_correlation.png' not
found: usin
g draft setting.

See the pdftex.def package documentation for explanation.
Type  H <return>  for immediate help.
 ...

l.637 ...spectratio]{images/CF_DF_correlation.png}

Try typing  <return>  to proceed.
If that doesn't work, type  X <return>  to quit.

LaTeX Font Info:    Font shape `T1/Merriwthr-OsF/b/n' will be
(Font)              scaled to size 5.0pt on input line 639.
[24]

LaTeX Warning: File `images/recall-precision.png' not found on input line
648.


! Package pdftex.def Error: File `images/recall-precision.png' not found:
using
 draft setting.

See the pdftex.def package documentation for explanation.
Type  H <return>  for immediate help.
```

```
 ...

l.648 ...aspectratio]{images/recall-precision.png}

Try typing  <return>  to proceed.
If that doesn't work, type  X <return>  to quit.

[25]

LaTeX Warning: File `images/stratified_recall.png' not found on input
line 658.



! Package pdftex.def Error: File `images/stratified_recall.png' not
found: usin
g draft setting.

See the pdftex.def package documentation for explanation.
Type  H <return>  for immediate help.
 ...

l.658 ...spectratio]{images/stratified_recall.png}

Try typing  <return>  to proceed.
If that doesn't work, type  X <return>  to quit.

[26]

LaTeX Warning: File `images/Fedrann-Shasta.png' not found on input line
669.


! Package pdftex.def Error: File `images/Fedrann-Shasta.png' not found:
using d
raft setting.

See the pdftex.def package documentation for explanation.
Type  H <return>  for immediate help.
 ...

l.669 ...th=\textwidth]{images/Fedrann-Shasta.png}

Try typing  <return>  to proceed.
If that doesn't work, type  X <return>  to quit.

[27]

LaTeX Warning: File `images/HLA_dot_plot.png' not found on input line
680.


! Package pdftex.def Error: File `images/HLA_dot_plot.png' not found:
using dra
```

```
ft setting.

See the pdftex.def package documentation for explanation.
Type  H <return>  for immediate help.
 ...

l.680 ...idth=\textwidth]{images/HLA_dot_plot.png}

Try typing  <return>  to proceed.
If that doesn't work, type  X <return>  to quit.

[28]

LaTeX Warning: File `images/parameter_tuning.png' not found on input line
692.


! Package pdftex.def Error: File `images/parameter_tuning.png' not found:
using
 draft setting.

See the pdftex.def package documentation for explanation.
Type  H <return>  for immediate help.
 ...

l.692 ...aspectratio]{images/parameter_tuning.png}

Try typing  <return>  to proceed.
If that doesn't work, type  X <return>  to quit.

[29]



[30








]
LaTeX Font Info:    Font shape `T1/Merriwthr-OsF/b/n' will be
(Font)              scaled to size 7.0pt on input line 714.
```

```
Overfull \hbox (15.12355pt too wide) in alignment at lines 734--734
[][][][][][][]
 []

[31]
Underfull \hbox (badness 10000) in alignment at lines 754--754
[][][][][][][]
 []

[32]
Underfull \hbox (badness 10000) in alignment at lines 773--773
[][][][][][][]
 []

[33] [34] [35]
Underfull \hbox (badness 10000) in alignment at lines 840--840
[][][][][]
 []

[36]
Underfull \hbox (badness 10000) in alignment at lines 859--859
[][][][][][][][]
 []

[37])
! Emergency stop.
<*> main.tex

*** (job aborted, no legal \end found)


Here is how much of TeX's memory you used:
 26060 strings out of 473583
 515747 string characters out of 5732343
 1979908 words of memory out of 5000000
 47590 multiletter control sequences out of 15000+600000
 1969214 words of font info for 766 fonts, out of 8000000 for 9000
 1141 hyphenation exceptions out of 8191
 123i,17n,131p,2014b,1052s stack positions out of
10000i,1000n,20000p,200000b,200000s
!  ==> Fatal error occurred, no output PDF file produced!
```

Manuscript

Click here to access/download;Manuscript;revision2026-0312.pdf

PAPER

FEDRANN: effective long-read overlap detection based on dimensionality reduction and approximate nearest neighbors

Jia-Yuan Zhang[1, 2, 3, 4, †], Changjiu Miao[4, †], Teng Qiu[3], Junyi He[4], Wenqi Cao[1, 2, 3, 4], Wei Lin[1, 2, 3, 5], Xiaoshuang Xia[6], Lei He[6], Chunlei Yang[4], Yuhui Sun[7], Tao Zeng[7], Yuxiang Li[6], Xun Xu[7,8*], Yijun Ruan[1,*] and Yuliang Dong[3,9*]

[1]Life Sciences Institute, Zhejiang University, Hangzhou, Zhejiang 310058, China and [2]State Key Laboratory of Genome and Multi-omics Technologies, BGI Research, Hangzhou 310030, China and [3]BGI Research, Hangzhou 310030, China and [4]BGI Hangzhou CycloneSEQ Technology Co., Ltd, Hangzhou 310030 , China and [5]College of Life Sciences, Zhejiang University, Hangzhou, Zhejiang 310058 and [6]BGI Research, Wuhan 430074, China and [7]State Key Laboratory of Genome and Multi-omics Technologies, BGI Research, Shenzhen 518083, China and [8]Guangdong Provincial Key Laboratory of Genome Read and Write, BGI Research, Shenzhen 518083, China and [9]BGI Research, Shenzhen 518083, China

*Correspondence: Yuliang Dong (dongyuliang@genomics.cn), Yijun Ruan (yjruan@zju.edu.cn), Xun Xu (xuxun@genomics.cn)
†These authors contributed equally.

Abstract

Overlap detection is a key step in *de novo* genome assembly pipelines based on the Overlap-Layout-Consensus (OLC) paradigm. Existing methods for overlap detection either rely on heuristic seed-and-extension strategies or locality-sensitive hashing (LSH), both of which struggle to handle repetitive genomic regions and the computational burden of large-scale datasets. Here, we present FEDRANN, a novel strategy for overlap graph construction that integrates feature extraction, dimensionality reduction (DR), and approximate nearest neighbor (ANN) search. We find the pipeline combining inverse document frequency (IDF) transformation, sparse random projection (SRP), and NNDescent enables accurate detection of overlaps across diverse datasets. We developed an efficient open-source implementation of this pipeline named Fedrann (`https://github.com/jzhang-dev/fedrann`). Through systematic benchmarking on real long-read sequencing data, we demonstrate that Fedrann produces overlap graphs comparable to or better than those generated by existing state-of-the-art tools, including MECAT2, minimap2, and wtdbg2, while maintaining competitive runtime. By integrating Fedrann into the Shasta assembler, we successfully reconstructed human whole genomes, achieving high assembly contiguity and quality. Despite being implemented primarily in Python, Fedrann achieves performance parity with tools written in compiled languages by leveraging C-accelerated numerical libraries and optimized batch-based matrix operations. Our results suggest that the combination of dimensionality reduction and ANN techniques offers a robust, scalable framework for accurate overlap detection in long-read assembly and broader sequence similarity search tasks.

Key words:
de novo assembly, overlap-layout-consensus, locality-sensitive hashing, dimensionality reduction, k-nearest neighbors

Introduction

De novo assembly reconstructs a genome from scratch using overlapping sequencing reads without a reference, which is crucial for studying novel genomes, uncovering structural variations, and exploring genetic diversity [1]. The advancement of long-read sequencing technologies, such as PacBio, Oxford Nanopore Technologies and BGI CycloneSEQ [2] platforms, has significantly improved *de novo* assembly by generating reads spanning repetitive regions and complex structural variants [3, 4]. Most long-read *de novo* assembly methods rely on the Overlap-Layout-Consensus (OLC) approach, which aligns reads based on overlaps and iteratively refines contigs to reconstruct high-quality genomes [5, 6, 7]. A key step in the OLC approach is overlap detection, which involves finding overlaps from a large collection of sequencing reads, where the existence of an overlap between a pair of reads is typically determined by their sequence similarity. Overlap detection faces two primary challenges: the computational burden of processing large amount of sequences (millions of reads for human-sized genomes), which demands efficient algorithms and substantial memory resources, and the inherent complexity caused by repetitive genomic sequences that create ambiguous overlaps [8]. These repeats often lead to fragmented or erroneous connections in the overlap graph, significantly complicating the assembly process and potentially introducing misassemblies. This issue is further exacerbated by sequencing errors, which introduce spurious overlaps and masking true overlaps. Overcoming these obstacles is crucial for accurate genome reconstruction.

Existing methods for overlap detection can be roughly categorized into two classes: seed-and-extension and locality-sensitive hashing (LSH) methods. The seed-and-extension strategy first identifies small exact or near-exact matches (seeds) between the query sequence and the target sequences. Seed matches are then filtered based on local density or other criteria. For each retained seed, the algorithm attempts to extend the alignment in both directions using dynamic programming (DP) to build a more comprehensive alignment. Finally, target sequences with the best alignments (according to certain heuristic criteria) are identified as overlapping sequences. Overlap detection based on seed-and-extension is widely adopted by both general-purpose sequence alignment tools such as BLAST [9], DALIGN [10], and minimap2 [11], as well as *de novo* assembly-oriented tools such as MECAT [12], wtdbg2 [13], PECAT [7], and xRead [14].

As seed-and-extension methods only perform time-consuming DP-based alignment on high-potential regions that contain many seed matches, they scale well to large databases and long query sequences. However, the seed matching and filtering steps are heuristic in their nature, and are prone to returning target sequences that are locally but not globally similar to the query sequence (false positives) [15]. In addition, these methods often depend on many parameters such as minimum seed density, which require careful tuning to achieve a good trade-off between accuracy and computational efficiency.

LSH-based methods, in particular MinHash, have also been widely used in overlap detection. MinHash was originally developed for text mining tasks, e.g. finding similar news articles [16] In MinHash, each sequence is first encoded as an unordered set of unique tokens, which are typically k-mers (subsequences of fixed-length k). The MinHash algorithm then applies multiple independent hash functions and records the minimum hash value of each set under each hash function. The resulting "sketch" serves as a compact approximation of the full set. Overlapping sequences will have more similar sketches compared to unrelated sequences. Popular *de novo assembly* tools that implement variants of MinHash include MHAP [17], Canu [5], and Shasta [6]. In addition, the overlap detection tool BLEND [18] uses another LSH-based method named SimHash to generate sketches for input sequences.

The LSH strategy replaces the time-consuming pairwise alignment step with rapid sketch comparison and therefore can potentially scale to large genomes while maintaining computational efficiency. For example, Shasta [6] is able to perform *de novo* assembly of the human genome within 6 hours, of which only 3% is spent on the MinHash step. However, a key limitation of this strategy is that highly repetitive tokens, such as k-mers from low-complexity genomic regions, can dominate the sketch, overshadowing more informative, unique tokens, affecting assembly quality in these regions [5]. Another challenge of this strategy is the difficulty in identifying overlaps between sequences that vary greatly in their lengths using their respective sketches [19, 6, 20].

To address the limitations of existing approaches for overlap detection, we developed a new strategy inspired by common single-cell sequencing workflows. Our approach, hereby referred to as FEDRANN, comprises three main steps: feature extraction (FE), dimensionality reduction (DR), and approximate nearest neighbor (ANN) search. We first designed a benchmarking pipeline to evaluate overlap graph quality using both simulated and real long-read sequencing data. Using this pipeline, we systematically compared various combinations of feature extraction, DR, and ANN methods across datasets of differing sizes and complexities. We then implemented the best-performing method, which involved inverse document frequency (IDF) transformation, sparse random projection (SRP) and ANN searching using NNdescent, as a integrated overlap detection tool named Fedrann. We benchmarked Fedrann against established tools, including minimap2, MHAP, MECAT2, wtdbg2, xRead, and BLEND. Our results demonstrate that Fedrann constructs accurate overlap graphs across genomes of varying sizes and complexities while maintaining competitive execution times. By integrating Fedrann into the Shasta assembly framework, we successfully reconstructed human whole genomes with high contiguity and accuracy. These results were comparable to, and in specific cases surpassed, the performance of the original Shasta assembler, highlighting the practical utility and potential of Fedrann for large-scale genomic assembly applications.

Results

Overview of the FEDRANN workflow

Many text-mining algorithms, such as MinHash and SimHash [21], employ the Bag-of-Words (BoW) model, which discards the ordering of tokens within a sequence and focuses solely on the presence or absence of each token, effectively encoding the sequence database as a high-dimensional sparse matrix [22]. We observed that, once sequences are encoded in this manner, overlap detection becomes analogous to performing a nearest-neighbor search in single-cell sequencing data, where both tasks rely on identifying similar items based on some similarity or distance metric in a high-dimensional sparse space (i.e., the sequence $\times$ token matrix or the cell $\times$ gene matrix). Based on this analogy, we hypothesized that dimensionality reduction and approximate nearest-neighbor (ANN) methods, which are widely used in single-cell data analysis, could also be applied to overlap detection with appropriate adaptations. Accordingly, we developed a new approach for overlap detection (**Figure 1**) consisting of three main steps: feature extraction, dimensionality reduction (DR), and ANN search, as described below.

Compiled on: March 12, 2026.
Draft manuscript prepared by the authors.

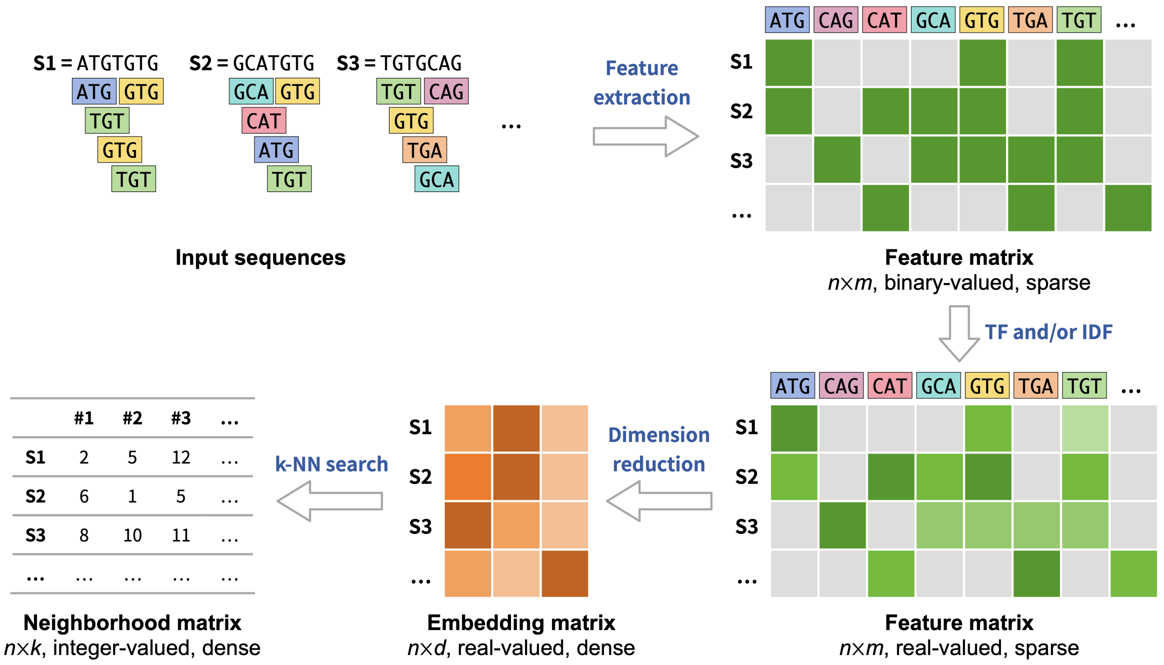

Figure 1. Diagrammatic representation of the proposed algorithm for sequence similarity search.
The algorithm is comprised of three key steps: (1) encoding the input sequences into a high-dimensional sparse feature matrix, with optional TF and/or IDF weighting; (2) applying a dimensionality reduction technique to embed the sequences into a space of lower dimensionality; (3) identifying sequence similarity through the use of a k-nearest neighbor search algorithm.

Feature extraction. Given a dataset of n sequences, we uniformly sampled an alphabet of m k-mers and encoded the dataset as a high-dimensional matrix X with n rows and m columns, where each entry $X[i, j]$ represents the weight of k-mer j in sequence i. By default, the weights were binary, indicating the presence or absence of a k-mer. Optionally, term frequency (TF) and/or inverse document frequency (IDF) transformations could be applied to adjust the weight of each k-mer based on its frequency across the dataset. Since each sequence typically contains only a small subset of all possible k-mers, the resulting matrix is sparse.

Dimensionality reduction. Although it is possible to directly apply nearest-neighbor algorithms to the high-dimensional feature matrix, doing so is often computationally expensive due to the large number of dimensions. To mitigate this, we applied dimensionality reduction techniques to project each high-dimensional feature vector into a lower-dimensional space, while aiming to preserve the relative distances between sequences.

ANN search. Since exact k-nearest neighbor (k-NN) search has quadratic time complexity, it becomes infeasible for large genomic sequencing datasets with millions of reads. To address this, we employed approximate k-NN algorithms to identify the most similar sequences for each query sequence in the dataset. These candidate overlaps were then used to define edges in the resulting overlap graph.

Many overlap detection tools, such as minimap2 and MHAP, aim to identify all pairs of overlapping reads in a dataset. Instead, FEDRANN employs a k-nearest neighbors (k-NN) search to retrieve only the top k most similar reads for each query read. These nearest neighbors are expected to exhibit the longest overlaps with the query. This approach aligns with the concept of the *best overlap graph* (BOG) [23, 5], which is grounded in the observation that full enumeration of overlapping read pairs is unnecessary, as shorter overlaps are typically redundant and pruned during graph refinement. For instance, the Shasta assembler retains by default only the top six edges per vertex in its overlap graph [6]. Restricting the number of edges in the overlap graph is advantageous, as it significantly reduces the complexity of subsequent graph layout computations.

To evaluate the performance of specific combinations of feature extraction, dimensionality reduction, and k-nearest neighbors (k-NN) search methods, we constructed overlap graphs for multiple long-read sequencing datasets and compared them against reference graphs derived from corresponding reference genomes (**Figure 2**). The datasets included sequencing reads from Oxford Nanopore Technologies (ONT), PacBio HiFi, and CycloneSEQ, all sampled from three repeat-rich regions of the human genome: the *HLA* immunogene cluster, the *IGK* immunoglobulin κ-light chain locus, and chromosome 22 (**Table S1**). The HLA and IGK regions each contain over 40% repetitive elements and are of notable medical relevance [24], while chromosome 22 encompasses large heterochromatic segments. For each method combination, performance was quantified by the error rate of the resulting overlap graph, defined as the proportion of incorrect edges among all edges.

Inverse document frequency (IDF) transformation improves overlap detection accuracy

We first evaluated the impact of different weighting schemes (TF-IDF, TF, IDF and raw TF) during feature extraction, and compared similarity metrics (Euclidean distance and cosine distance) used in k-NN search. As the sizes of the three benchmarking regions were relatively small (3.92–5.75 Mb), we were able to use k-d tree based exact k-NN search without dimensionality reduction to inspect the upper limit of the FEDRANN strategy.

Our results showed that using cosine distance for ENN search resulted in significantly higher accuracy compared to Euclidean distance (**Figures 3**, **S1**, and **S2**). This is likely because Euclidean distance is sensitive to vector magnitude, while cosine distance captures only the angular difference between vectors, making it more robust to variations in sequence length. As a result, overlapping sequences of different lengths may still exhibit low cosine distances but high Euclidean distances, leading to better overlap detection when cosine similarity is used.

We evaluated the impact of different weighting schemes (TF,

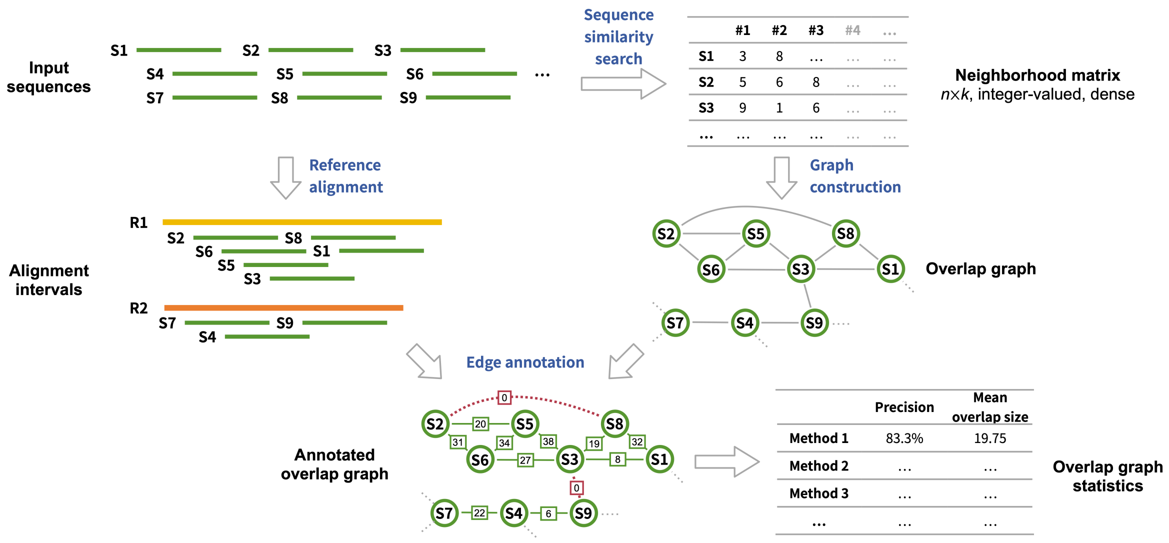

Figure 2. Assessment of sequence similarity search algorithms.
For the analysis of each algorithm, an overlap graph is constructed by linking each query sequence to the top k sequences identified as most similar by the algorithm. This overlap graph is subsequently assessed against a reference graph, which is obtained by aligning the input sequences to a reference genome to ascertain the accuracy and overlap size of each edge. The algorithm's performance is evaluated based on precision and mean overlap size.

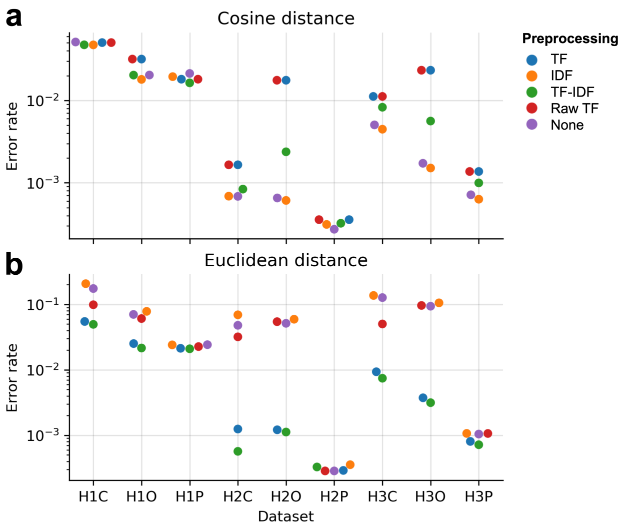

Figure 3. Assessment of preprocessing methods and similarity metric for overlap detection.
(a–b) Overlap detection error rate of pipelines combining various preprocessing methods for feature extraction (TF, IDF, TF-IDF, Raw TF, and no preprocessing) and exact k-NN search based on cosine **(a)** or Euclidean **(b)** distance. Top six neighbors according to each distance metric were used to construct overlap graphs for evaluation. Abbreviations: TF, term frequency; IDF, inverse document frequency.

IDF, IDF and raw TF) on overlap graph quality. For baseline comparison, we also constructed a binary feature matrix that only records the presence (1) or absence (0) of k-mers in each read, discarding all frequency and weighting information. While TF-IDF has been used in assemblers like Canu [5] to help resolve repetitive sequences, our experiments indicated that IDF alone, rather than TF or TF-IDF, was more effective in improving graph quality. When cosine distance was used, applying IDF weighting led to better performance than both TF-IDF and unweighted representations, whereas TF alone and raw TF degraded performance relative to the unweighted baseline (**Figures 3**, **S1**, and **S2**). These findings suggest that high-frequency k-mers—such as those originating from transposable elements, short tandem repeats, or other repetitive regions—are a major source of error in overlap graph construction. By down-weighting these features, IDF enhances the ability to distinguish between true overlaps. In contrast, TF and raw TF emphasizes frequent features and may counteract the benefits of IDF, thereby reducing accuracy.

We plotted the overlap graph error rate as a function of the number of overlap candidates (k). As expected, the error rate increased with larger values of k, reflecting the greater difficulty in identifying larger numbers of correct overlaps. Notably, the combination of IDF transformation and cosine distance consistently outperformed other method combinations in terms of accuracy. Based on this observation, we selected the IDF–cosine distance combination for subsequent analyses, including evaluating the effects of read length, coverage depth, and sequencing accuracy, as well as for selecting dimensionality reduction and ANN search methods, as described below.

Longer reads, deeper coverage, and higher accuracy improve overlap detection

We systematically evaluated the impact of read length, coverage depth, and sequencing accuracy to overlap detection by generating simulated datasets with PBSIM3 for human chromosome 22, covering read lengths of 10–30 kb, accuracy of 91%–99%, and depths of 10×–50×. We found that longer reads and deeper coverage had a large positive effect on overlap graph accuracy (**Figure S3**). We hypothesize that this was because longer reads and deeper coverage both lead to longer overlaps between adjacent reads, facilitating overlap detection. Meanwhile, improved sequencing accuracy only had a moderate positive effect on overlap graph accuracy. For example, at 30× coverage and mean read length 20 kb, increasing sequencing accuracy from 91% to 99% only reduced error rate from 8.34% to 5.55% for human chromosome 22. This observation suggested that our FEDRANN strategy is relatively robust to sequencing errors.

Sparse random projection enables scalable and accurate dimensionality reduction

Dimensionality reduction (DR) techniques are widely used in single-cell sequencing analyses to reduce noise, accelerate computation, and facilitate data visualization. To evaluate their utility in overlap graph construction, we tested a range of DR methods,

including linear approaches such as principal component analysis (PCA) [25] and sparse random projection (SRP), as well as non-linear methods including Uniform Manifold Approximation and Projection (UMAP) [26], Spectral Embedding, and scBimapping. We also evaluated SimHash [27], a locality-sensitive hashing (LSH)–based method related to random projection, which has been widely used in text mining and recently applied to biological sequence analysis [18, 28].

For each method, we first constructed the input feature matrix using IDF weighting, then applied the DR method, and finally computed exact *k*-nearest neighbors (ENN) from the resulting low-dimensional embeddings (**Figure 1**). We found that all tested DR methods except UMAP were able to generate accurate overlap graphs for smaller datasets (**Figure 4a**), suggesting that they preserved the relative pairwise distances between sequences sufficiently well. However, when applied to larger datasets, most methods failed to complete within the predefined resource constraints (wall clock time $\leq$ 6 hours; peak memory $\leq$ 1 TB) (UMAP), or the matrix size surpassed the maximum constraints of the methods (PCA, Spectral and scBiMapping), resulting in dimensionality reduction failure. The only methods that successfully scaled to the largest datasets, H3C, H3O and H3P, was SRP, GRP and SimHash. Time and memory profiling on dataset H3P further confirmed that SRP was the fastest method and had the lowest memory usage among those tested (**Figure 4b**). Based on this robust balance between distance preservation and computational efficiency, SRP was selected as our DR method for further benchmarking.

The accuracy of *k*-NN search after DR typically depends on the embedding dimension, as higher dimensions allow more information from the original feature matrix to be retained. To characterize this relationship, we varied the embedding dimension for each dataset using SRP. The results revealed that increasing the embedding dimension enhances the "feature resolution" of the reads, enabling the ANN algorithm to more effectively resolve ambiguities in repetitive or highly similar genomic regions (**Figures S4** and **S5**). To better understand this trend, we examined the correlation between pairwise cosine distances before and after SRP in dataset H3C. The results showed a strong positive correlation that steadily improved with increasing dimensions, with the Pearson R^2 rising from 0.72 at 100 dimensions to 0.99 at 3000 dimensions (**Figure S6**). This indicates that higher-dimensional projections achieve near-perfect structural fidelity to the original high-dimensional data. Furthermore, we analyzed the correlation between post-reduction cosine distances and actual overlap sizes. As dimensions increased, the R^2 significantly improved from 0.43 to 0.83, demonstrating that higher dimensions allow the embedding space to more accurately represent the underlying genomic relationships (**Figure S6**). However, the observed "diminishing returns" (**Figures S4**) suggest an informational saturation point; for most datasets, 1,000 dimensions are sufficient to capture the essential variance of the *k*-mer distribution. Beyond this threshold, additional dimensions primarily capture redundant information or noise, offering marginal precision gains at the expense of higher memory and computational overhead (**Figures S13**).

NNDescent offers the best trade-off between speed and accuracy among ANN methods

In addition to dimensionality reduction, another strategy to accelerate k-NN search is to use approximate k-NN (ANN) algorithms, which leverage indexing structures or heuristics to reduce the computational cost of neighbor retrieval. We evaluated five ANN approaches that are widely used in single-cell sequencing and information retrieval applications: NNDescent [27], Hierarchical Navigable Small World (HNSW) [29], product quantization (PQ) [30], inverted file index with product quantization (IVF-PQ) [30], and random projection forest (RPF) [31]. All ANN meth-

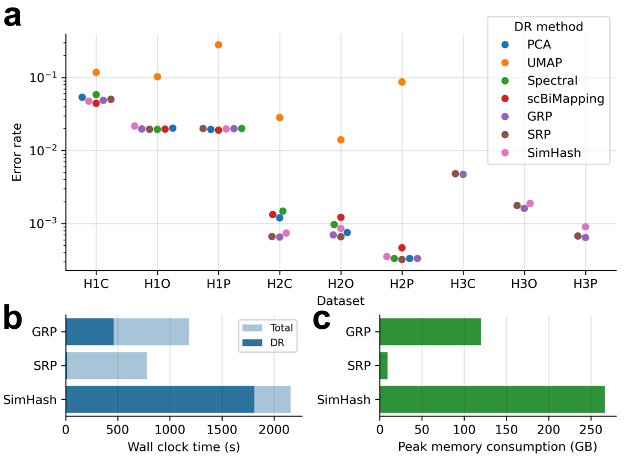

Figure 4. Assessment of dimensionality reduction methods.
(a) Overlap detection error rate of various dimensionality reduction methods. IDF preprocessing was used in feature extraction. Exact Hamming distance (for SimHash) or cosine distance (for other methods) was used in k-NN search. Top six neighbors according to each distance metric were used to construct overlap graphs for evaluation. Missing dots indicate that the corresponding methods failed to generate results under predefined computational constraints (wall clock time $\leq$ 6 h and peak memory $\leq$ 900 GB). **(b-c)** Wall clock time **(b)** and peak memory consumption **(c)** of various dimensionality reduction methods in dataset H3P. Methods that failed to generate results are not shown. Abbreviations: PCA, principal component analyses; UMAP, Uniform Manifold Approximation and Projection; Spectral, spectral embedding; GRP, Gaussian random projection, SRP: sparse random projection; DR, dimensionality reduction.

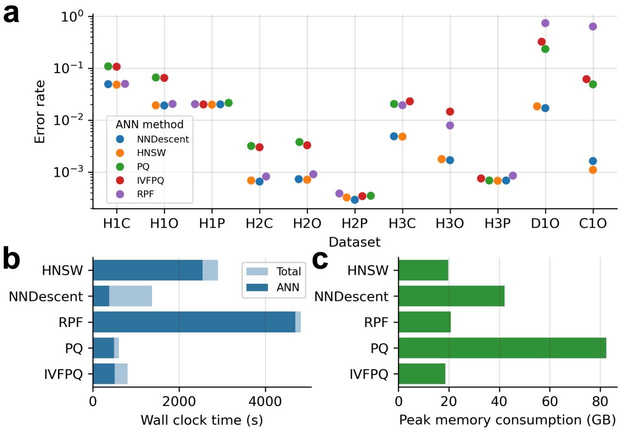

Figure 5. Assessment of ANN methods.
(a) Overlap detection error rate of various ANN methods. IDF preprocessing was used in feature extraction. Feature matrices were reduced to 1,000 dimensions using Sparse Random Projection prior to ANN search. Cosine distance was used in ANN search. Top six neighbors were used to construct overlap graphs for evaluation. Missing dots indicate that the corresponding methods failed to generate results under predefined computational constraints (wall clock time $\leq$ 6 h and peak memory $\leq$ 900 GB). **(b-c)** Wall clock time **(b)** and peak memory consumption **(c)** of selected ANN methods in dataset D1O. Abbreviations: ANN, approximate *k*-nearest neighbors; HNSW, Hierarchical Navigable Small World; PQ, Product Quantization; IVFPQ, Inverted File with Product Quantization; RPF, Random Projection Forest.

ods were applied to low-dimensional embeddings generated by sparse random projection (SRP).

Among these methods, NNDescent and HNSW achieved high precision when used to construct overlap graphs, whereas PQ, IVF-PQ and RPF performed less favorably in terms of accuracy (**Figure 5a**). In terms of computational efficiency, NNDescent and IVF-PQ were the fastest, while RPF consumed the least memory (**Figure 5b–c**). Across all configurations, the ANN step emerged as the computational bottleneck in the FEDRANN pipeline, often requiring significantly more time than the SRP step (**Figure 5b**),

although both steps had comparable peak memory usage (**Figures 4c** and **5c**). Consequently, NNDescent was selected as the optimal ANN method for our workflow, as it offered the best trade-off between speed and accuracy for scalable overlap graph construction.

Efficient implementation of the IDF-SRP-NNDescent pipeline

The experimental results established the combination of Inverse Document Frequency (IDF), Sign-Random Projections (SRP), and NNDescent as a novel framework for constructing overlap graphs from genomic sequencing data. To transition this theoretical finding into a functional application, we developed Fedrann, an overlap detection tool optimized for processing large-scale, long-read datasets (**Figure S7**; see **Methods**).

Fedrann utilizes a memory-aware architecture designed to circumvent the memory bottlenecks typically associated with high-dimensional feature matrices. By avoiding full matrix materialization, the tool enables scalable analysis of human-scale genomic data. We implemented batch-wise processing for the computationally intensive upstream stages, including feature construction, weighting, and dimensionality reduction. In this workflow, sparse *k*-mer feature matrices are generated on demand and transformed incrementally in fixed-size batches. This ensures that intermediate data structures remain compact and transient. Consequently, peak memory consumption is governed by the batch size and the sampled *k*-mer alphabet rather than the total read count, providing a significant advantage over methods reliant on global feature matrices.

During the pre-processing stage, we leverage Jellyfish [32] to construct the *k*-mer library and filter low-frequency *k*-mers based on their occurrence statistics. Jellyfish outputs the total number of occurrences (i.e. collection frequency, CF [33]) of each *k*-mer across the sequence dataset. Our empirical analysis demonstrates that, on a logarithmic scale, CF is nearly identical to document frequency (DF)—the number of reads containing a specific *k*-mer—with an R^2 value reaching 0.9990 (see Figure S8). Based on this observation, we implemented inverse collection frequency (ICF) as a high-performance surrogate for the conventional IDF. This approach allows us to directly utilize pre-computed Jellyfish statistics for effective feature weighting, thereby bypassing the need for an additional, computationally intensive *k*-mer counting step.

An additional implementation optimization is the fusion of linear transformations. Because IDF reweighting and dimensionality reduction are both linear operations, they are combined into a single weighted projection matrix that is reused across all batches. This design eliminates intermediate feature transformations, reduces memory traffic, and improves computational efficiency, particularly under parallel execution. After dimensionality reduction, approximate nearest neighbor search is performed on the full embedding matrix using pyNNDescent [34], which is itself highly optimized for efficiency and scalability. Together, these design choices enable Fedrann to scale efficiently to large long-read datasets while maintaining manageable computational and memory demands.

Fedrann enables high-precision, time-efficient overlap detection on large genomes

We benchmarked Fedrann against a set of established overlap detection tools: minimap2 (in all-vs-all mode), MECAT2, MHAP, BLEND, wtdbg2, and xRead. Benchmarking was performed on real whole-genome sequencing datasets from three species with differing genome sizes and complexities: *Caenorhabditis elegans*, *Drosophila melanogaster*, and *Homo sapiens* (**Table S1**).

The overlap detection tools benchmarked in this study exhibit distinct trade-offs between precision and recall. Most tools—specifically minimap2, MECAT2, MHAP, BLEND, and wtdbg2—are designed to identify all potential overlapping pairs, generally prioritizing high recall. In contrast, xRead returns only a subset of the top overlap candidates per read, sacrificing recall for enhanced precision [14]. Fedrann follows a similar strategy by returning the top *k* candidates for each read, though higher recall can be achieved by increasing the value of *k*. To evaluate these differences, we performed a precision-recall analysis using the H3C dataset (**Figure S9**). To ensure a fair comparison, we post-processed the outputs of all tools to retain only the top *k* candidates per read, calculating precision and recall across a range of *k* values. Our analysis revealed that when *k* is lower than the expected number of true overlaps per read (a function of coverage depth and read length), the recall for most tools (excluding xRead) increases linearly with *k* while precision remains relatively stable. In this regime, MECAT2 and Fedrann demonstrated the highest precision. Once *k* exceeds the actual number of true overlaps, recall plateaus while precision begins to decline. This drop in precision was most pronounced for Fedrann, which is expected as the algorithm is configured to output exactly *k* candidates, whereas other tools employ internal thresholds to filter low-confidence overlaps. Stratifying target reads by overlap length confirmed that Fedrann prioritizes the detection of longer overlaps, with shorter overlaps being identified at larger *k* values (**Figure S10**).

Real-world *de novo* assembly applications present unique requirements and challenges. For example, longer overlaps are generally more valuable than shorter ones, as the latter are frequently pruned as transitive edges during graph simplification. Furthermore, an assembly graph must maintain high continuity rather than being fragmented into isolated subgraphs. Finally, the overlap detection tool must scale efficiently to large genomic datasets containing millions of reads. To evaluate performance under these demanding conditions, we benchmarked the tools on human whole-genome sequencing (WGS) datasets using four key metrics: error rate (1 − precision), mean overlap size, the number of correct overlap candidates per read (#COC), and the number of connected components (#CC). Mean overlap size assesses the ability to identify the longest, most informative overlaps for each read. Higher #COC values indicate superior per-read connectivity, whereas lower #CC values reflect reduced fragmentation and higher global contiguity. Additionally, we recorded the computational resource usage, including execution time and peak memory consumption, for each tool.

A memory limit of 950 GB was enforced during benchmarking. Under this constraint, MHAP failed to complete on all three human datasets (H4C, H4O, and H4P), and xRead failed on H4C. All other tools completed successfully (**Figure 6**). Fedrann achieved the highest accuracy on four out of five datasets (**Figure 6a**); the exception was H4C, where MECAT2 performed slightly better. wtdbg2 also showed strong accuracy, consistently ranking second or third across datasets. In terms of mean overlap size, MECAT2 consistently ranked lowest, while other tools performed comparably (**Figure 6b**). For graph contiguity, Fedrann, wtdbg2, MHAP, and MECAT2 generally outperformed the other methods, with no clear single leader (**Figure 6c**). Analysis of low-connectivity reads under varying #COC thresholds showed that xRead produced a large number of poorly connected reads—for instance, in dataset H4P, over 50% of reads had fewer than five correct edges. Fedrann ranked first or second across all datasets (**Figure 6d**).

In terms of computational cost, all tools completed the smaller datasets (C10 and D10) within one hour and with moderate memory usage (<60 GB), except MHAP, which consumed up to 150 GB (**Figure 6e–g**). In the context of the larger human datasets (H4C, H4O, H4P), where computational efficiency is paramount, Fedrann demonstrated superior performance. It achieved the shortest execution times on the H4C (3.02 h) and H4O (3.58 h) datasets. On the H4P dataset, Fedrann was the second fastest tool (2.97 h), trailing only wtdbg2. Notably, while wtdbg2 required over 20 h to

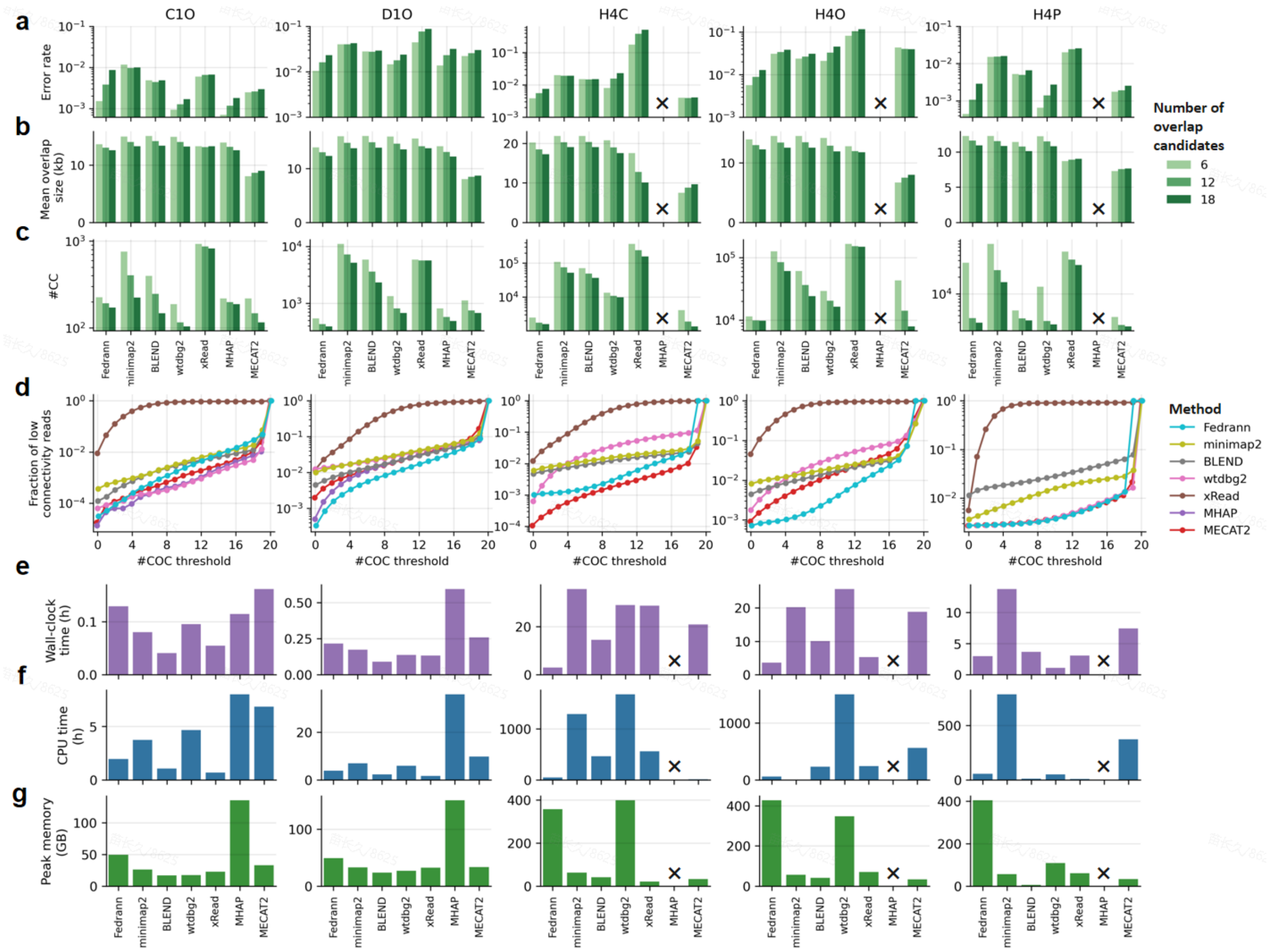

Figure 6. Benchmarking of overlap detection tools.
(a–c) Overlap detection error rate **(a)**, mean overlap size **(b)**, and #CC **(c)** of various overlap detection tools in five whole-genome datasets (columns). Top 6, 12, or 18 overlap candidates (colors) according to cosine distance (for Fedrann) or overlap size (for other methods) were used to construct overlap graphs for evaluation. **(d)** Fraction of low-connectivity reads plotted against #COC threshold for various overlap detection tools. **(e–g)** Wall-clock time **(e)**, CPU time **(f)**, and peak memory **(g)** usage of various overlap detection tools. In **(a–c)** and **(e–g)**, black crosses indicate that the corresponding methods failed to generate results under predefined computational constraints (wall clock time $\leq$ 72 h and peak memory $\leq$ 900 GB). These methods were not included in **(d)**. Abbreviations: #CC, number of connected components; #COC, number of correct overlap candidates.

process the H4C and H4O datasets, its execution time significantly decreased to 1.1 h for the H4P dataset. The memory efficiency of the evaluated tools varied significantly across the human genome datasets. Minimap2, BLEND, xRead, and MECAT2 maintained relatively low memory footprints, all requiring less than 100 GB. In contrast, Fedrann exhibited higher memory demands, with peak usage ranging between 350–450 GB. The memory consumption of wtdbg2 was comparable to Fedrann on the H4C and H4O datasets; however, it demonstrated significantly higher efficiency on the H4P dataset, where its peak memory usage dropped to approximately 100 GB.

In summary, Fedrann, wtdbg2, and MECAT2 emerged as the best-performing tools when considering both graph quality and computational efficiency. Fedrann offered a balanced trade-off across accuracy, overlap size, graph contiguity, and speed, though it incurred a high memory footprint. wtdbg2 was accurate and memory-efficient but lagged in contiguity, connectivity, and speed (except for HiFi data). MECAT2 was both accurate and efficient in memory usage, but failed to capture some of the longest overlaps. Overall, our results highlight Fedrann as a robust and competitive new tool for long-read overlap detection.

The Fedrann-Shasta pipeline generates high-quality human whole-genome assembly contigs

To demonstrate the practical utility of Fedrann within end-to-end Overlap-Layout-Consensus (OLC) frameworks, we developed an integrated assembly pipeline by modifying the Shasta assembler [6]. While Shasta natively employs a MinHash variant for overlap discovery, our modified version ingests Fedrann's output to construct the initial overlap graph. Using this Fedrann-Shasta pipeline, we performed *de novo* assembly of the human genome using the H4C and H4O datasets. The results were benchmarked against assemblies generated by the unmodified Shasta assembler using otherwise identical parameters (**Table S6**).

For the H4C dataset (raw read N50 of 40.67 kb), the Fedrann-Shasta assembly achieved a contig N50 of 44.45 Mb, with the longest contig spanning 110.3 Mb (**Figure S11**). The assembly demonstrated high genomic fidelity with a BUSCO completeness of 98.10%. Notably, chromosomes 3, 4, 12, and 20 were assembled nearly to completion, with discontinuities appearing only at centromeric and telomeric regions (**Figure S11**). These metrics are highly comparable to those of the native Shasta assembly (N50 45.07 Mb, longest contig 137.99 Mb, BUSCO 98.30%). In contrast, on the H4O dataset (shorter raw-read N50 of 23.06 kb), Fedrann-Shasta significantly outperformed the native assembler, yielding a contig N50 of 17.07 Mb—a three-fold increase over the 5.73 Mb N50 produced by Shasta. K-mer based phred-scale quality values (QV) for the H4O assemblies (37.5) were slightly higher than those of the H4C assemblies (35.7) and remained consistent regardless of the assembly method used.

To assess the impact of Fedrann on resolving complex, repeat-rich regions, we analyzed the HLA region within both H4C assemblies (**Figure S12**). Both pipelines partitioned the HLA region into two contigs with a breakpoint at roughly the same location. However, the native Shasta assembly exhibited a collapse of a 40 kb repeat unit, incorrectly merging two copies into one. This misassembly was correctly resolved in the Fedrann-Shasta assembly, suggesting that Fedrann's high-accuracy overlap detection may improve assembly quality in repetitive genomic contexts.

This proof-of-concept study confirms that Fedrann is a robust alternative for overlap detection in large-scale *de novo* assembly, capable of producing high-quality human genome reconstructions. While these results are promising, further engineering of the alignment refinement stage may continue to enhance overall assembly contiguity and accuracy.

Application notes

We provide the following recommendations for users applying Fedrann to large-scale genomic datasets. The software utilizes a transparent parameter space where most variables exhibit predictable linear trade-offs: increasing parameter values generally enhances predictive accuracy at the cost of increased memory consumption and execution time. For human genome-scale applications, our parameter sensitivity analysis (**Figure S13a**) suggests that a configuration of 1,000 dimensions for dimensionality reduction, 600 trees for NNDescent, and a 15% k-mer sampling fraction provides an optimal balance between accuracy and computational footprint. Critically, k-mer size selection is governed by the specific error profiles and sequence characteristics of the sequencing platform rather than computational scaling alone. For optimal performance, we recommend the following empirical defaults based on our whole-genome evaluations: $k = 31$ for PacBio HiFi, $k = 13$ for ONT, and $k = 11$ for CycloneSEQ (**Figure S13b**). Detailed instructions on optimizing Fedrann parameters are included in **Supplementary Note 1**.

For human genome applications, Fedrann typically requires a memory footprint of 300–500 GB. In environments where this capacity is unavailable, the pipeline remains functional on machines with lower RAM (e.g., 256 GB) by leveraging system swap space (**Table S7**). At a 256 GB limit using HDD-based swap, Fedrann successfully completed the task in 15.69 h, producing results identical to the unconstrained baseline (3.02 h), despite both configurations utilizing 64 threads. Notably, the use of high-speed Solid State Drives (SSDs) for the swap partition significantly accelerated the computation; the SSD-based configuration finished in 14.87 h using only 32 threads, effectively outperforming the 64-thread HDD execution. While relying on swap space substantially increases total execution time due to disk I/O bottlenecks, it provides a viable pathway for users to process human whole-genome datasets on systems with limited physical memory. Detailed instructions on running Fedrann with limited physical memory are included in **Supplementary Note 2**.

Discussion

We proposed a novel strategy for overlap graph construction that integrates dimensionality reduction (DR) and approximate nearest neighbor (ANN) techniques. We conducted comprehensive evaluations on both simulated and real long-read sequencing datasets. Among the tested combinations, the pipeline consisting of inverse document frequency (IDF) transformation, sparse random projection (SRP), and NNDescent achieved the best trade-off between accuracy and computational efficiency. Based on these findings, we developed an efficient, open-source, and user-friendly implementation named Fedrann. Benchmarking results showed that Fedrann generated overlap graphs that were comparable to or better than those produced by state-of-the-art tools such as MECAT2 and wtdbg2, in terms of both overlap accuracy and contiguity for large genomes, without sacrificing computational speed.

Although the concept of treating overlap detection as a special case of k-NN search has been suggested in previous studies [23, 6], ANN search methods have not been directly applied to overlap identification. This omission is likely due to computational constraints: feature matrices derived from the human genome often contain millions of sequences, each represented by millions or even billions of features, posing a significant challenge even for state-of-the-art ANN algorithms such as HNSW and NNDescent. Common dimensionality reduction techniques, including PCA and UMAP, also fail to scale effectively to matrices of this magnitude. In this study, we identify SRP as a highly scalable dimensionality reduction method that preserves pairwise sequence distances (Fig-

ures 4 and S6), enabling accurate and efficient overlap detection via NNDescent, thereby unlocking new possibilities for overlap detection in the assembly of large genomes.

The current implementation of Fedrann is written primarily in Python and builds upon general-purpose numerical libraries rather than domain-specific sequence processing frameworks. Nevertheless, Fedrann achieves competitive performance, constructing overlap graphs for the human genome within four hours, faster than many state-of-the-art overlap detection tools implemented in compiled languages such as C and C++. This efficiency largely stems from formulating overlap detection as a sequence of matrix operations, enabling extensive use of highly optimized dense and sparse linear algebra routines provided by `NumPy` and `SciPy`. Representing sequence data in matrix form allows Fedrann to benefit from vectorization, cache-friendly memory access, and parallel execution, mitigating much of the overhead typically associated with high-level languages. Sparse Random Projection (SRP) plays a key role in this design by offering both algorithmic simplicity and computational efficiency: it is data-independent, inexpensive to compute, and naturally compatible with incremental, batch-wise processing, making it particularly well suited to large-scale overlap detection. Despite these strengths, the overall memory consumption of Fedrann remains higher than that of most existing overlap detection tools benchmarked, highlighting an important direction for future optimization through more memory-efficient data representations, shared-memory designs, or alternative implementations of ANN methods.

Beyond computational performance, another strength of using matrix-based representations is the clarity and modularity of the pipeline. Most parameters in Fedrann have well-defined meanings and predictable effects on the output, typically reflecting trade-offs between overlap detection accuracy and resource usage, without being tightly coupled to a specific sequencing platform. For instance, increasing the embedding dimension in SRP generally improves accuracy at the cost of additional computation. This platform-agnostic design allows Fedrann to be easily adapted to emerging sequencing technologies with minimal parameter tuning.

To showcase the practical utility of Fedrann in a full assembly workflow, we developed the Fedrann-Shasta pipeline to generate high-quality human *de novo* assemblies, illustrating the feasibility of modular frameworks that utilize Fedrann for overlap detection. In many existing assemblers, intermediate results such as overlap graphs are encapsulated in opaque, in-memory data structures. While this design is often motivated by computational efficiency, it creates a tight coupling between pipeline stages that limits transparency and reusability. Consequently, incorporating Fedrann into existing tools is non-trivial, requiring deep expertise in undocumented internal structures. Because this integration is currently constrained by the internal architecture of established tools, we consider our current Fedrann-Shasta results a successful proof-of-concept rather than the upper limit of Fedrann's potential. The lack of modularity in existing tools often forces developers to build new assemblers from the ground up as sequencing technologies emerge. We hope that modular tools like Fedrann, which expose transparent and interpretable data structures, will lower these barriers and facilitate the development of more adaptable next-generation assembly algorithms.

Finally, we note that overlap graph construction can be regarded as a special case of the broader sequence similarity search (SSS) problem, where the objective is to retrieve the most similar sequences for each query from a large reference database. The use of dimensionality reduction (DR) and approximate nearest neighbor (ANN) methods for SSS has been explored in previous studies. For instance, the Rust library `annembed` [35] combines locality-sensitive hashing (LSH) with hierarchical navigable small world (HNSW) graphs to perform metagenomic binning, a task that clusters contigs or reads from metagenomic sequencing into bins ideally representing individual microbial genomes. Our results demonstrate FEDRANN as a robust and scalable framework for sequence similarity detection, with potential applicability to a variety of SSS tasks, including metagenomic binning. Another promising application is taxonomic classification in metagenomics, where query sequences (reads or contigs) are classified against a static, pre-indexed database of microbial reference genomes. Unlike overlap detection or binning, which typically operate on all input sequences jointly, classification often requires rapid querying of individual sequences against the database. The IDF-SRP-NNDescent pipeline investigated here is well-suited for such classification tasks. Both IDF and SRP are linear, independent transformations, while NNDescent supports incremental and efficient nearest neighbor search. Nonetheless, further development and empirical validation are needed to assess the performance of FEDRANN in metagenomic binning, taxonomic classification, and other long-read sequence analysis applications.

Methods

Fedrann implementation

We implemented the IDF–SRP–NNDescent pipeline as a standalone command-line tool for overlap detection, named Fedrann (Figure S7). Fedrann accepts FASTA or FASTQ files as input and identifies a fixed number of candidate overlaps (default: $k = 20$) for each sequence. The tool generates a tab-separated values (TSV) file characterizing sequence overlaps through the reporting of sequence indices, distance metrics, and neighbor ranks.

The implementation is primarily written in Python and leverages C-accelerated numerical libraries for efficiency, including optimized sparse and dense matrix operations provided by `NumPy` [36] and `SciPy` [37]. Together with extensive parallelization and memory-aware design choices, these optimizations enable scalable application to human-scale long-read sequencing datasets.

Feature extraction

The pipeline utilizes Jellyfish for efficient k-mer counting and filtering, retaining only k-mers whose multiplicities exceed a predefined threshold to remove noise arising from sequencing errors. From the filtered set, a k-mer alphabet is constructed by uniformly sampling a predefined fraction, typically between 5–20%.

Although the document frequency (DF) of a k-mer is conventionally defined as the number of reads containing that k-mer, we found no existing tools capable of computing this quantity efficiently at scale. Fedrann therefore adopts the total count (i.e. collection frequency, CF) of a k-mer within the dataset as a computationally efficient approximation of its DF, enabling direct use of Jellyfish's high-performance k-mer counting. The implementation relies on Jellyfish's canonical k-mer representation, in which the count of each k-mer reflects the combined frequency of the sequence and its reverse complement. Under this approximation, the inverse document frequency (IDF) of k-mer j in dataset D is defined as

$$idf(j, D) = \log \frac{|N|}{c_j},$$

where $|N|$ denotes the total number of reads in D and c_j is the total count of k-mer j in the dataset.

To construct the feature matrix, a custom multi-threaded C++ program is used to locate each selected k-mer within every read, as well as within its reverse complement. The resulting k-mer–read associations are written to a compact binary file, which is subsequently loaded in batches to assemble a sparse feature matrix on demand. Rather than explicitly materializing this high-dimensional matrix, each sparse batch is streamed directly into

the downstream dimensionality reduction stage.

Dimensionality reduction

Leveraging the fact that both IDF weighting and Sparse Random Projection (SRP) are linear operations, the implementation fuses these steps by pre-multiplying the SRP projection matrix with the IDF weight vector. Concretely, an SRP matrix $\mathbf{R} \in \mathbb{R}^{n \times m}$ is first generated, where n denotes the number of sampled k-mers and m is the target reduced dimension, and is then multiplied by the IDF weight vector of size $n \times 1$ to form an integrated weighted projection matrix. This intermediate matrix is stored in shared memory, enabling concurrent access by multiple child processes without redundant data copying.

During processing, sparse feature batches of size $b \times n$ (default: b = 100,000 reads), loaded from the binary file, are independently transformed via matrix multiplication with the shared weighted projection matrix. The resulting low-dimensional embeddings are written to a shared dense output array. This high-concurrency, incremental architecture eliminates the need to materialize a monolithic feature matrix, thereby avoiding memory bottlenecks and ensuring a manageable memory footprint while enabling efficient processing of human-scale genomic datasets.

Approximate nearest neighbor search

Approximate nearest neighbor search is performed on the resulting embedding matrix using the PyNNDescent library [34], which efficiently constructs a neighborhood graph in a highly parallelized manner. For each sequence, the algorithm retrieves its top-k nearest neighbors (default: $k = 20$) along with the corresponding distance scores. The resulting neighborhood graph is then converted into a candidate overlap table and written to disk as the final output of Fedrann.

Fedrann-Shasta pipeline

The Fedrann-Shasta pipeline is an integrated framework for long-read genome assembly that couples Fedrann's overlap detection with a modified version of the Shasta assembler [6]. In this configuration, Fedrann functions as the upstream discovery module, generating the initial set of candidate overlaps that are subsequently refined by Shasta.

To facilitate this integration, we implemented a dedicated entry point within the Shasta source code that bypasses the native MinHash-based discovery stage. A custom loading function ingests the Fedrann output and performs the following operations:

- **ID mapping**: Translates read names to Shasta's internal read identifiers.
- **Parsing and deduplication**: Processes the candidate list to eliminate redundant read pairs.
- **Candidate injection**: Populates Shasta's alignment candidate container with oriented read pair entries.

Once injected, Shasta computes marker-based alignments for each candidate pair and applies its standard quality criteria. The overlap graph (referred to as the "read graph" in Shasta terminology) is then constructed from these validated alignments, where vertices represent oriented reads and edges represent accepted overlaps. Consistent with the original Shasta design, only the best k alignments per read are retained to maintain graph efficiency. All subsequent assembly stages—including marker graph construction, graph simplification, and consensus generation—are performed using the unmodified Shasta pipeline.

Sequencing datasets

To evaluate various overlap detection methods, we used genomic sequencing data from three model species: *Homo sapiens*, *Caenorhabditis elegans*, and *Drosophila melanogaster*. The corresponding reference genomes were obtained from the following sources: the complete human T2T-CHM13 assembly v2.0 [3], the *Caenorhabditis elegans* WBcel235 assembly (WormBase release WS285; NCBI Assembly ID: GCA_000002985.3),and the *Drosophila melanogaster* Release 6 genome assembly (NCBI Assembly ID: GCF_000001215.4).

Simulated sequencing datasets with varying read lengths, error rates, and coverage depths were generated using PBSIM3 [38], based on the `ERRHMM-ONT-HQ` model. Real sequencing datasets from Oxford Nanopore Technologies (ONT), and PacBio HiFi platforms were obtained from publicly available databases (**Table S1**). ONT R10 sequencing data for HG002 was obtained from the Oxford Nanopore Technologies EPI2ME repository [39]. ONT R9 sequencing data for *C. elegans* (SRR10028111) and *D. melanogaster* (SRR13070625) were retrieved from the NCBI Sequence Read Archive (SRA). PacBio HiFi sequencing data for the HG002 sample was downloaded from the Genome in a Bottle (GIAB) project [40]. CycloneSEQ G400-ER sequencing data for HG002 was generated in-house using genomic DNA extracted from the HG002 lymphoid cell line following standard library preparation and sequencing protocols.

Three genomic regions from the human genome were used for selecting appropriate methods for feature extraction, dimensionality reduction and k-NN search, including the *HLA* immunogene cluster, the *IGK* immunoglobulin κ-light chain locus, and chromosome 22 (**Table S1**). For each region, real sequencing data were mapped to the corresponding reference genome using minimap2 with the following parameters: `-k 19 -w 5 -A 3 -B 2 -m 250 --secondary=no`, and reads belonging to the given region were extracted. Whole-genome sequencing data for *C. elegans* (ONT R10), *D. melanogaster* (ONT R10) and *H. sapiens* (ONT R10, PacBio HiFi, and CycloneSEQ G400-ER) were used for benchmarking overlap detection tools.

Overlap graph construction and evaluation

For a dataset of n sequencing reads, an undirected overlap graph $G = (V, E)$ was constructed from pairs of reads identified as overlapping. Each vertex in the graph represented an oriented read—either in its forward or reverse-complement orientation—resulting in a total of $2n$ vertices. An edge $\{u, v\}$ was added to the graph if read u was among the k nearest neighbors of read v, or vice versa, indicating that the two reads likely originated from overlapping genomic regions. The total number of edges in the graph ranged from kn to $2kn$, depending on the degree of mutuality among nearest-neighbor relationships (i.e., whether overlaps were reciprocal or one-sided).

For each benchmarking dataset, overlap graphs constructed using nearest neighbors identified by a specific method were evaluated against a reference graph $G' = (V, E')$ built from the same dataset. The reference graph shared the same set of vertices V as the overlap graphs. For real sequencing data, the reference edges E' were defined based on the alignment positions of reads in the reference genome. Sequencing reads were filtered based on the following criteria to remove ambiguously aligned reads: (1) read length $\geq$ 5 kb; (2) aligned fraction $\geq$ 50%; (3) mapping quality $\geq$ 30. For simulated data, the edges E' were determined according to the genomic intervals from which each read was simulated, as reported by PBSIM3.

For each edge $\{u, v\}$ identified in overlap graph $G = (V, E)$, if the same edge exist in the reference graph G', this edge was considered correct. Otherwise, this edge was considered incorrect. We

used four metrics to quantitatively evaluate the quality of an overlap graph: error rate, mean overlap size, the number of correct overlap candidates (#COC) and the number of connected components (#CC). The error rate was defined as the number of incorrect edges divided by the number of total edges. Mean overlap size was defined as the arithmetic mean of overlap size of all edges. The overlap size of incorrect edges were considered zero for this calculation. #COC was defined as the degree of each node after removing any incorrect edges. If a node does not have any correct edges, it was referred to as a singleton. #CC was defined as the number of connected components of the overlap graph after removing any incorrect edges.

Benchmarking overlap detection tools

We benchmarked Fedrann (v0.5.4) against six state-of-the-art tools: minimap2 (v2.24), xRead (v1.0.0), BLEND (v1.0.0), MHAP (v2.1.1), MECAT2 (v20190314), and wtdbg2 (v2.5). All alignments were filtered using two criteria: (1) $\geq$ 100 matched bases and (2) $\geq$ 10% alignment identity. Notably, for minimap2 PAF files (which produced exceptionally large outputs exceeding 4TB for human whole-genome sequencing read alignments), we increased the identity threshold to 30% to reduce computational overhead. All overlap detection tools were benchmarked using 64 threads on a dual-socket Linux server equipped with AMD EPYC 9654 processors (192 physical cores/384 threads total, 2.4 GHz base clock) and 2 TB of DDR5 RAM. Refer to **Table S4** and **S5** for the specific parameters used for each tool.

Whole genome assembly and evaluation

To evaluate the practical utility of our approach in large-scale genomic workflows, we performed whole-genome assembly benchmarks on the H4C and H40 datasets, comparing the performance of the integrated Fedrann-Shasta pipeline against the standalone Shasta assembler. For the Fedrann-Shasta implementation, we utilized Fedrann (v0.5.4) and specified the number of nearest neighbors (k) for each read as $k = 30$ for the H4C dataset and $k = 50$ for the H40 dataset. In both the integrated pipeline and standalone runs, we employed Shasta using the `--config Nanopore-May2022` parameter to ensure a consistent assembly parameters. The resulting assemblies were rigorously evaluated across multiple dimensions: QUAST v5.3.0 [41] was used to calculate NGA50 values for structural contiguity, while yak v0.1-r69-dirty [42] was utilized to estimate k-mer based Consensus Quality (QV) and k-mer completeness for base-level accuracy. Biological completeness was assessed using BUSCO v5.8.0 [43] against the primates_odb10 lineage database. Dot plots were generated using the Lakeview library [44].

Availability of Source Code and Requirements

Project name: Fedrann
Project homepage: https://github.com/jzhang-dev/FEDRANN
License: GPL-3.0
Operating system(s): Linux (Ubuntu 20.04 or later)
Package management: Docker
Programming language: Python, C++
Hardware requirements: CPU with AVX2 support; minimum 32GB RAM (256GB+ recommended for human genome datasets)
RRID: SCR_027416
Integrated pipeline: Fedrann–Shasta pipeline: https://github.com/jzhang-dev/Fedrann-Shasta
Reproducible workflows for benchmarking and evaluation: https://github.com/jzhang-dev/kNN-overlap-finder

Data Availability

The CycloneSEQ G400-ER sequencing data generated in this study is available via the publicly available database CNGBdb (accession number CNX1236558). These data have also been deposited in the National Center for Biotechnology Information (NCBI) databases. The BioProject accession number is PRJNA1321476. The raw sequence reads are available in the Sequence Read Archive (SRA) under the accession number SRR35291479. The associated BioSample accession is SAMN51209978.

The simulated datasets and numerical data underlying the figures and supplementary materials are available in the GigaScience GigaDB database [45].

Ethics statement

This study was conducted in adherence to ethical standards to ensure the responsible and respectful treatment of all data and materials. Ethical approval for this study was obtained from the Institutional Review Board of BGI (FT 20060, FT 17099).

Acknowledgments

This work was supported by the National Key RD Program of China (No. 2024YFC3406300), the "Pioneer" and "Leading Goose" RD Program of Zhejiang (No. 2024C03004), and the Zhejiang Province Postdoctoral Research Merit-based Funding Program (ZJ2025175).

Competing interests

The authors have submitted patent applications related to the methods or results presented in this manuscript. Prof. Xun Xu serves as an Editor-in-Chief for GigaScience. He was blinded from reviewing or making final decisions on the manuscript. The article was subject to the journal's standard procedures, with peer review handled independently of Prof. Xu.

References

1. Sohn Ji, Nam JW. The present and future of de novo whole-genome assembly. Briefings in Bioinformatics 2018;19(1):23–40. Doi: 10.1093/bib/bbw096.
2. Zhang JY, Zhang Y, Wang L, Guo F, Yun Q, Zeng T, et al. A single-molecule nanopore sequencing platform. bioRxiv 2024;p. 2024.08.01.605050. Doi: 10.1101/2024.08.19.608720.
3. Nurk S, Koren S, Rhie A, Rautiainen M, Bzikadze AV, Mikheenko A, et al. The complete sequence of a human genome. Science 2022;376(6588):44–53. Doi: 10.1126/science.abj6987.
4. Espinosa E, Bautista R, Larrosa R, Plata O. Advancements in long-read genome sequencing technologies and algorithms. Genomics 2024;116(2):110842. Doi: 10.1016/j.ygeno.2024.110842.
5. Koren S, Walenz BP, Berlin K, Miller JR, Bergman NH, Phillippy AM. Canu: scalable and accurate long-read assembly via adaptive k-mer weighting and repeat separation. Genome Research 2017;27(5):722–736. Doi: 10.1101/gr.215087.116.
6. Shafin K, Pesout T, Lorig-Roach R, Haukness M, Olsen HE, Bosworth C, et al. Nanopore sequencing and the Shasta toolkit enable efficient de novo assembly of eleven human genomes. Nature Biotechnology 2020;38(9):1044–1053. Doi: 10.1038/s41587-020-0503-6.
7. Nie F, Ni P, Huang N, Zhang J, Wang Z, Xiao C, et al. De novo diploid genome assembly using long noisy reads. Nature

Communications 2024;15(1):2964. Doi: 10.1038/s41467-024-47349-7.
8. Rizzi R, Beretta S, Patterson M, Pirola Y, Previtali M, Della Vedova G, et al. Overlap graphs and de Bruijn graphs: data structures for de novo genome assembly in the big data era. Quantitative Biology 2019;7:278–292. Doi: 10.1007/s40484-019-0181-x.
9. Altschul SF, Madden TL, Schäffer AA, Zhang J, Zhang Z, Miller W, et al. Gapped BLAST and PSI-BLAST: a new generation of protein database search programs. Nucleic Acids Research 1997;25(17):3389–3402. Doi: 10.1093/nar/25.17.3389.
10. Myers G. Efficient local alignment discovery amongst noisy long reads. In: International Workshop on Algorithms in Bioinformatics Springer; 2014. p. 52–67. Doi: 10.1007/978-3-662-44753-6_5.
11. Li H. Minimap2: pairwise alignment for nucleotide sequences. Bioinformatics 2018;34(18):3094–3100. Doi: 10.1093/bioinformatics/bty191.
12. Xiao CL, Chen Y, Xie SQ, Chen KN, Wang Y, Han Y, et al. MECAT: fast mapping, error correction, and de novo assembly for single-molecule sequencing reads. Nature Methods 2017;14(11):1072–1074. Doi: 10.1038/nmeth.4432.
13. Ruan J, Li H. Fast and accurate long-read assembly with wtdbg2. Nature Methods 2020;17(2):155–158. Doi: 10.1038/s41592-019-0669-3.
14. Kong T, Wang Y, Liu B. xRead: a coverage-guided approach for scalable construction of read overlapping graph. GigaScience 2025;14:giaf007. Doi: 10.1093/gigascience/giaf007.
15. Vinga S, Almeida J. Alignment-free sequence comparison—a review. Bioinformatics 2003;19(4):513–523. Doi: 10.1093/bioinformatics/btg005.
16. Broder AZ. On the resemblance and containment of documents. In: Proceedings. Compression and Complexity of SEQUENCES 1997 IEEE; 1997. p. 21–29. Doi: 10.1109/SEQUEN.1997.666900.
17. Berlin K, Koren S, Chin CS, Drake JP, Landolin JM, Phillippy AM. Assembling large genomes with single-molecule sequencing and locality-sensitive hashing. Nature Biotechnology 2015;33(6):623–630. Doi: 10.1038/nbt.3238.
18. Firtina C, Park J, Alser M, Kim JS, Cali DS, Shahroodi T, et al. BLEND: a fast, memory-efficient and accurate mechanism to find fuzzy seed matches in genome analysis. NAR Genomics and Bioinformatics 2023;5(1):lqad004. Doi: 10.1093/nargab/lqad004.
19. Li H. Minimap and miniasm: fast mapping and de novo assembly for noisy long sequences. Bioinformatics 2016;32(14):2103–2110. Doi: 10.1093/bioinformatics/btw152.
20. Greenberg GC. Analysis and applications of k-mer based methods in bioinformatics. PhD thesis, University of Illinois at Urbana-Champaign; 2023.
21. Charikar MS. Similarity estimation techniques from rounding algorithms. In: Proceedings of the 34th Annual ACM Symposium on Theory of Computing; 2002. p. 380–388. Doi: 10.1145/509907.509965.
22. Ponsero AJ, Miller M, Hurwitz BL. Comparison of k-mer-based de novo comparative metagenomic tools and approaches. Microbiome Research Reports 2023;2(4):27. Doi: 10.20517/mrr.2023.26.
23. Miller JR, Delcher AL, Koren S, Venter E, Walenz BP, Brownley A, et al. Aggressive assembly of pyrosequencing reads with mates. Bioinformatics 2008;24(24):2818–2824. Doi: 10.1093/bioinformatics/btn548.
24. Zhang JY, Roberts H, Flores DS, Cutler AJ, Brown AC, Whalley JP, et al. Using de novo assembly to identify structural variation of eight complex immune system gene regions. PLOS Computational Biology 2021;17(8):e1009254. Doi: 10.1371/journal.pcbi.1009254.
25. Dunteman GH. Principal components analysis, vol. 69. Sage Publications; 1989.
26. McInnes L, Healy J, Saul N, Großberger L. UMAP: Uniform manifold approximation and projection for dimension reduction. Journal of Open Source Software 2018;3(29):861. Doi: 10.21105/joss.00861.
27. Dong W, Moses C, Li K. Efficient k-nearest neighbor graph construction for generic similarity measures. In: Proceedings of the 20th International Conference on World Wide Web; 2011. p. 577–586. Doi: 10.1145/1963405.1963487.
28. Li J, Tian Y, Wang Y, Jin L. dna2bit: high performance genomic distance estimation software for microbial genome analysis. Frontiers in Microbiology 2024;15:1521181. Doi: 10.3389/fmicb.2024.1521181.
29. Malkov YA, Yashunin DA. Efficient and robust approximate nearest neighbor search using hierarchical navigable small world graphs. IEEE Transactions on Pattern Analysis and Machine Intelligence 2018;42(4):824–836. Doi: 10.1109/TPAMI.2018.2889473.
30. Jegou H, Douze M, Schmid C. Product quantization for nearest neighbor search. IEEE Transactions on Pattern Analysis and Machine Intelligence 2010;33(1):117–128. Doi: 10.1109/TPAMI.2010.57.
31. Yan D, Wang Y, Wang J, Wang H, Li Z. K-nearest neighbor search by random projection forests. IEEE Transactions on Big Data 2019;7(1):147–157. Doi: 10.1109/TBDATA.2019.2908178.
32. Marçais G, Kingsford C. A fast, lock-free approach for efficient parallel counting of occurrences of k-mers. Bioinformatics 2011;27(6):764–770. Doi: 10.1093/bioinformatics/btr011.
33. Manning CD, Raghavan P, Schütze H. Introduction to Information Retrieval. Cambridge, UK: Cambridge University Press; 2008.
34. McInnes L, Healy J, PyNNDescent: Fast Approximate Nearest Neighbor Descent; 2020. https://github.com/lmcinnes/pynndescent. GitHub repository.
35. Zhao X, Tian Y, Huang K, Zheng B, Zhou X. Towards efficient index construction and approximate nearest neighbor search in high-dimensional spaces. Proceedings of the VLDB Endowment 2023;16(8):1979–1991. Doi: 10.14778/3594512.3594527.
36. Harris CR, Millman KJ, Van Der Walt SJ, Gommers R, Virtanen P, Cournapeau D, et al. Array programming with NumPy. Nature 2020;585(7825):357–362. Doi: 10.1038/s41586-020-2649-2.
37. Virtanen P, Gommers R, Oliphant TE, Haberland M, Reddy T, Cournapeau D, et al. SciPy 1.0: fundamental algorithms for scientific computing in Python. Nature Methods 2020;17(3):261–272. Doi: 10.1038/s41592-019-0686-2.
38. Ono Y, Hamada M, Asai K. PBSIM3: a simulator for all types of PacBio and ONT long reads. NAR Genomics and Bioinformatics 2022;4(4):lqac092. Doi: 10.1093/nargab/lqac092.
39. Wright C, November 2020 GM24385 Dataset Release; 2020. https://labs.epi2me.io/gm24385_2020.11/.
40. Zook JM, Hansen NF, Olson ND, Chapman L, Mullikin JC, Xiao C, et al. A robust benchmark for detection of germline large deletions and insertions. Nature Biotechnology 2020;38(11):1347–1355. Doi: 10.1038/s41587-020-0538-8.
41. Gurevich A, Saveliev V, Vyahhi N, Tesler G. QUAST: quality assessment tool for genome assemblies. Bioinformatics 2013;29(8):1072–1075. Doi: 10.1093/bioinformatics/btt086.
42. Li H, yak: Yet another k-mer analyzer; 2020. https://github.com/lh3/yak. GitHub repository.
43. Simão FA, Waterhouse RM, Ioannidis P, Kriventseva EV, Zdobnov EM. BUSCO: assessing genome assembly and annotation completeness with single-copy orthologs. Bioinformatics 2015;31(19):3210–3212. Doi: 10.1093/bioinformatics/btv351.
44. Zhang JY, Lakeview: A Python library for creating publication-quality genomic visualisations; 2023. https://github.com/jzhang-dev/lakeview. GitHub repository.
45. Zhang J, Miao C, Qiu T, He J, Cao W, Lin W, et al., Support-

ing data for "FEDRANN: effective long-read overlap detection based on dimensionality reduction and approximate nearest neighbors". GigaScience Database; 2026. https://doi.org/10.5524/102809, doi: 10.5524/102809.

Supplementary Information

Supplementary Note 1: Parameter Optimization Guide

The performance of Fedrann in overlap detection is influenced by the interplay between sequencing data characteristics and algorithmic parameters. This guide provides a framework for users to optimize settings based on their specific research context.

Sequencing accuracy
: The selection of k-mer size (k) involves a fundamental trade-off between sensitivity and specificity. A single sequencing error (substitution or indel) renders all k overlapping k-mers covering that position mismatched.

- **Low-accuracy reads** (e.g., CycloneSEQ, ONT; ≈95–99% accuracy): We recommend using a smaller k (typically k = 11 to 13). Smaller k-mers are more resilient to errors, ensuring that overlapping read pairs still share a sufficient number of identical "anchors" to maintain a high similarity score in the embedding space.
- **High-accuracy reads** (e.g., PacBio HiFi; >99% accuracy): We recommend using a larger k (typically k = 19 to 31). High-fidelity reads allow for larger k-mers, which significantly enhances feature specificity and reduces the chance of accidental matches between unrelated genomic regions.

Genome complexity and repeats
Genome size and the density of repetitive elements directly impact the "collision rate" within the embedding space. Larger genomes generate more reads and a higher volume of distinct k-mer features; consequently, they are considerably more computationally intensive than smaller genomes and require higher embedding dimensions to maintain resolution.

- **High-complexity genomes** (e.g., human, large polyploid plants): To manage the vast feature space and resolve repetitive regions, users should increase the k-mer sampling fraction (e.g., 15%–20%) and the embedding dimension (e.g., 1,000–3,000). Higher dimensionality provides the necessary capacity to separate complex features in the latent space.
- **Simple genomes** (e.g., bacteria, yeast): For smaller, less repetitive genomes, the embedding dimension can be reduced (e.g., 500) to accelerate processing and minimize the memory footprint without sacrificing precision.

Coverage depth
We utilize k-mer frequency-based filtering to distinguish genuine genomic features from sequencing noise and ultra-high-frequency repeats.

- **Low-coverage datasets** ($<30\times$): The minimum k-mer frequency threshold should be set low (e.g., 2). Higher thresholds may mistakenly discard k-mers that appear infrequently due to low sampling depth, resulting in fragmented overlap graphs.
- **High-coverage datasets** ($>60\times$): The k-mer frequency threshold can be increased to aggressively filter out erroneous k-mers, thereby sharpening the signal-to-noise ratio in the feature matrix.

Supplementary Note 2: Running Fedrann on Limited Physical Memory

For large-scale applications such as human whole-genome assembly, Fedrann typically requires 300–500 GB of RAM. If physical memory is constrained, the pipeline can be successfully executed by leveraging system swap space. This section describes how to configure and validate swap space to maintain stability and avoid unexpected process termination due to out-of-memory (OOM) events.

To prevent the operating system from terminating the process, the total available memory (physical RAM plus swap) should be sufficient to cover Fedrann's peak memory usage. In practice, we recommend provisioning swap space such that:

- **RAM + swap ≥ 500 GB** for human whole-genome assemblies.
- Swap space should reside on a **high-speed SSD or NVMe device**, as swap access may be frequent during peak memory phases.

Verify existing swap and disk type
First, inspect the currently active swap devices or swap files:

```
swapon --show
```

Example output:

```
NAME       TYPE SIZE   USED PRIO
/swapfile  file 500G   120G   -2
```

This output reports the total swap size, current usage, and priority. If swap is already heavily utilized under normal conditions, additional swap space should be provisioned before running Fedrann.

Next, verify that the underlying storage device hosting the swap space is a solid-state drive (SSD) rather than a rotational hard disk drive (HDD):

```
lsblk -d -o NAME,ROTA,SIZE,MODEL
```

Example output:

```
NAME ROTA SIZE MODEL
nvme0n1 0  1.8T Samsung SSD 980 PRO
sda      1  8.0T ST8000DM004
```

A value of `0` in the `ROTA` column indicates a non-rotational device (SSD or NVMe). For acceptable performance, swap space should be placed exclusively on such devices (e.g., `nvme0n1`), as using rotational disks may lead to severe slowdowns or system unresponsiveness under heavy swapping.

Create and enable additional swap space
If the existing swap space is insufficient, create a new swap file on an SSD-backed filesystem. The example below creates a 500 GB swap file:

```
sudo fallocate -l 500G /swapfile
sudo chmod 600 /swapfile
sudo mkswap /swapfile
sudo swapon /swapfile
```

Expected output from `mkswap`:

```
Setting up swapspace version 1, size = 500 GiB
no label, UUID=xxxxxxxx-xxxx-xxxx-xxxx-xxxxxxxxxxxx
```

After enabling the swap file, re-check the swap status to confirm that it is active:

```
swapon --show
```

Example output:

```
NAME       TYPE SIZE   USED PRIO
/swapfile  file 500G   0B     -2
```

Ensure that the combined capacity of physical memory and swap space is sufficient to accommodate Fedrann's peak memory usage.

Adjust kernel swappiness
The Linux kernel parameter `vm.swappiness` controls how aggressively inactive memory pages are moved from RAM to swap. Increasing this value allows the kernel to utilize swap space earlier, which can help avoid abrupt out-of-memory (OOM) termination during transient memory spikes.

Check the current setting:

```
cat /proc/sys/vm/swappiness
```

For memory-intensive workloads such as Fedrann, we recommend:

- Setting `vm.swappiness` to at least **40**
- Using values in the range **60–100** when physical memory is highly constrained

Apply the change immediately:

```
sudo sysctl vm.swappiness=60
```

To make this configuration persistent across reboots, add the following line to `/etc/sysctl.conf`:

```
vm.swappiness=60
```

After completing these steps, Fedrann can be executed using the standard workflow. During execution, it is advisable to monitor swap utilization (e.g., via `free`, `vmstat`, or `htop`) to ensure that swap usage remains within acceptable limits.

Supplementary figures

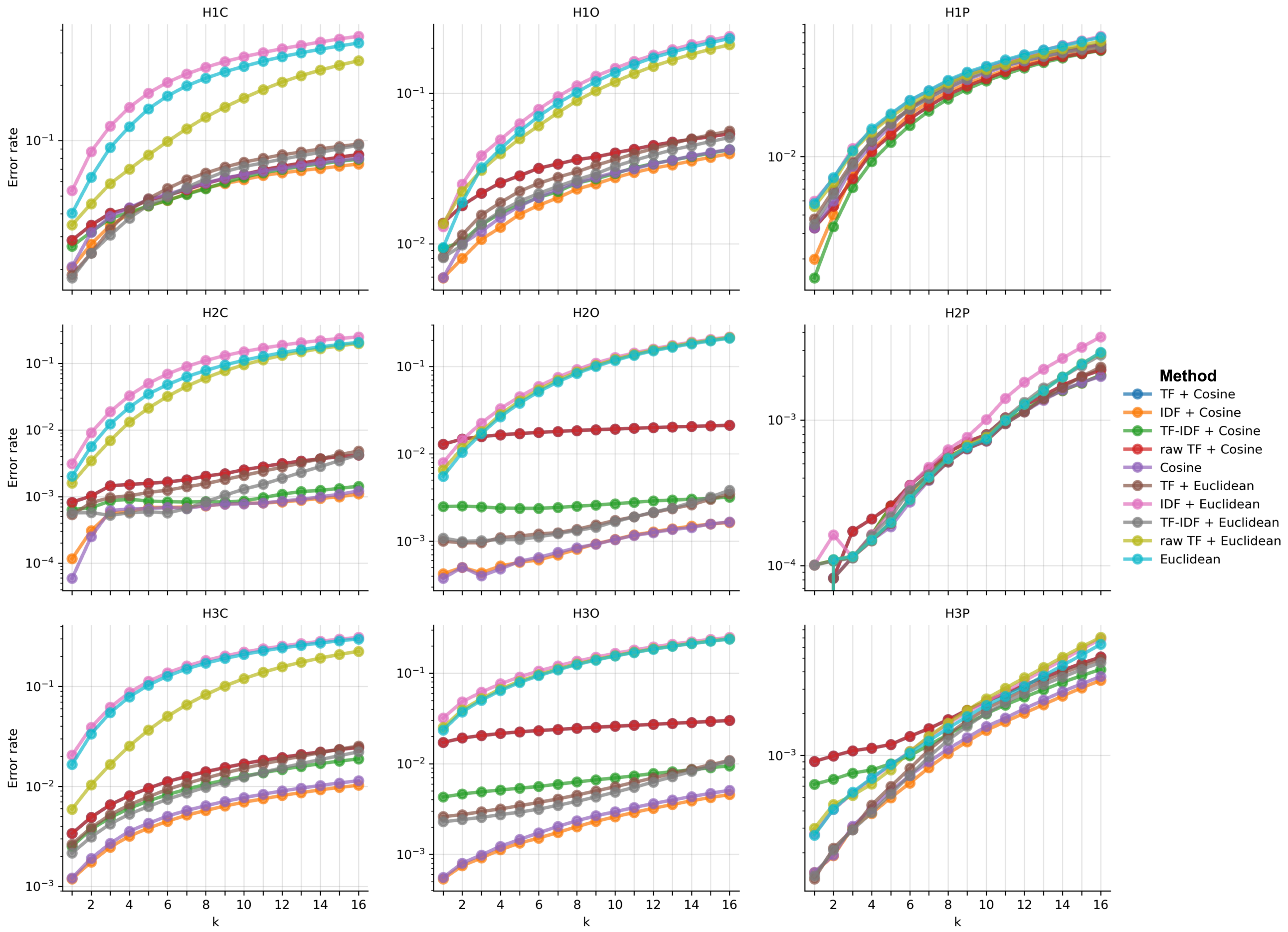

Figure S1. Error rate of preprocessing methods and distance metrics for overlap detection.
Overlap detection error rate of various preprocessing methods combined with Euclidean or cosine distance in various datasets. Each dot represents top-k neighbors error rate. No dimensionality reduction or ANN methods were used in k-NN search. Each dot represents the overlap detection error rate for a graph constructed using top-k nearest neighbors.

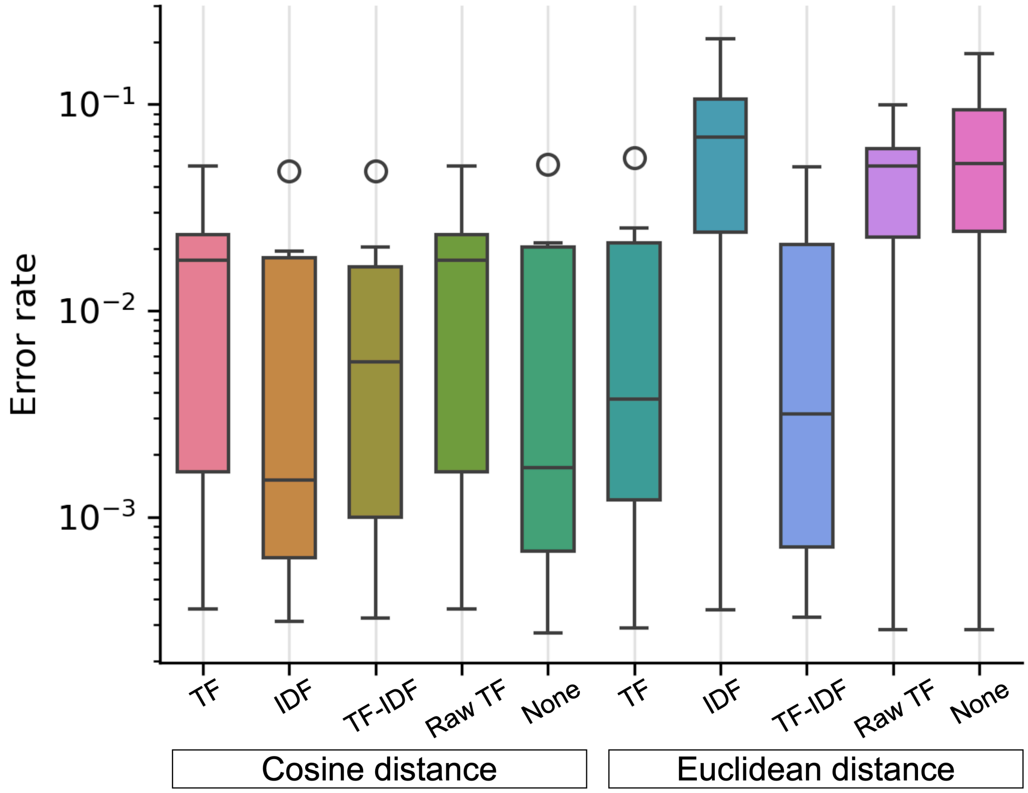

Figure S2. Error rate distribution of preprocessing methods and distance metrics for overlap dectection.
Overlap detection error rate distribution in overlap detection across datasets (H1C-H3C, H1O-H3O, H1P-H3P) using different text preprocessing methods (TF, IDF, TF-IDF, Raw TF, None) with cosine and Euclidean distance metrics. No dimensionality reduction or ANN methods were used in k-NN search. Boxplots show quartile ranges with whiskers indicating 1.5×IQR, and circles denote outliers.

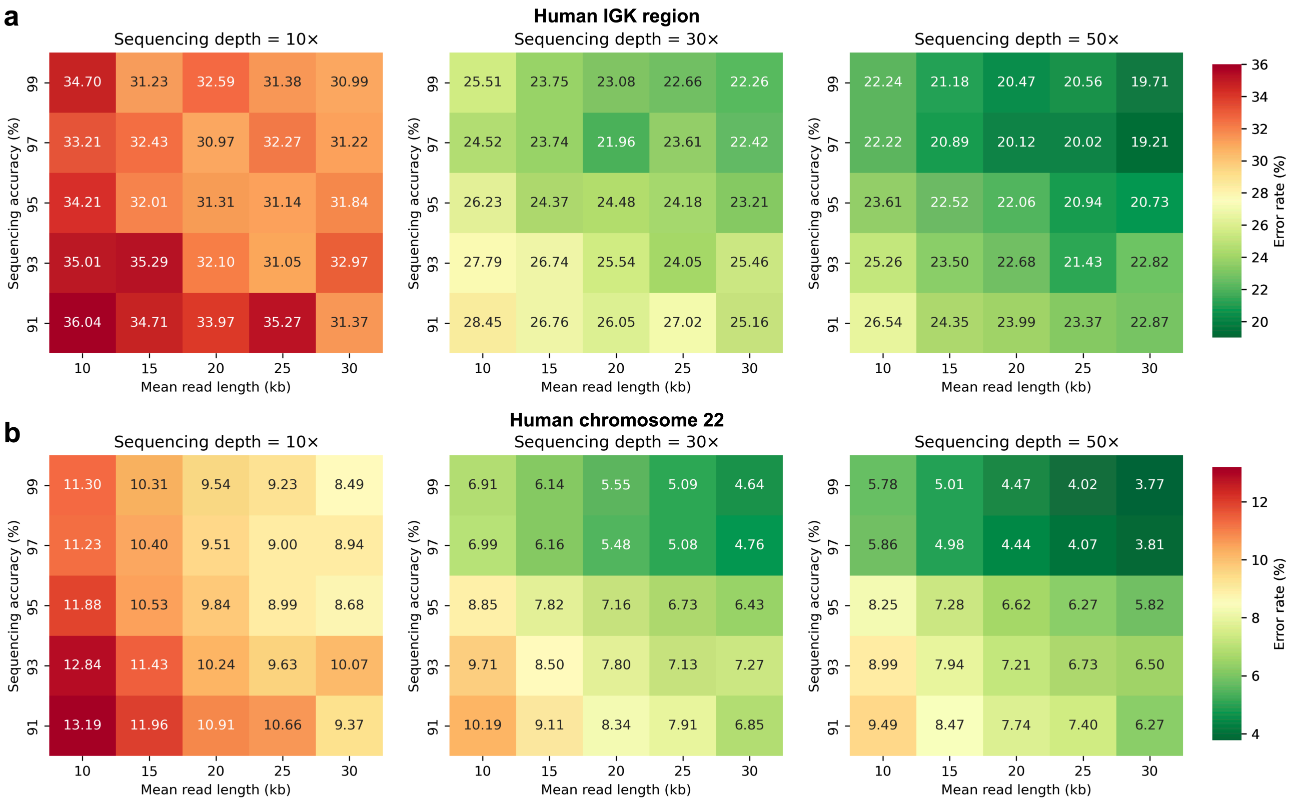

Figure S3. Assessment of simulated sequence reads.
(a–b) Heatmap showing overlap detection error rates for simulated sequencing reads from the human IGK region **(a)** and chromosome 22 **(b)**. Each cell representing error rates under different combinations of sequencing depth (10×, 30×, 50×), accuracy (91%–99%), and read length (10–30 kb). IDF preprocessing was used in feature extraction. Exact cosine distance was used in k-NN search. Top six neighbors according to each distance metric were used to construct overlap graphs for evaluation. The heatmap used a red-to-green color gradient, where red indicates higher error rates and green indicates lower error rates.

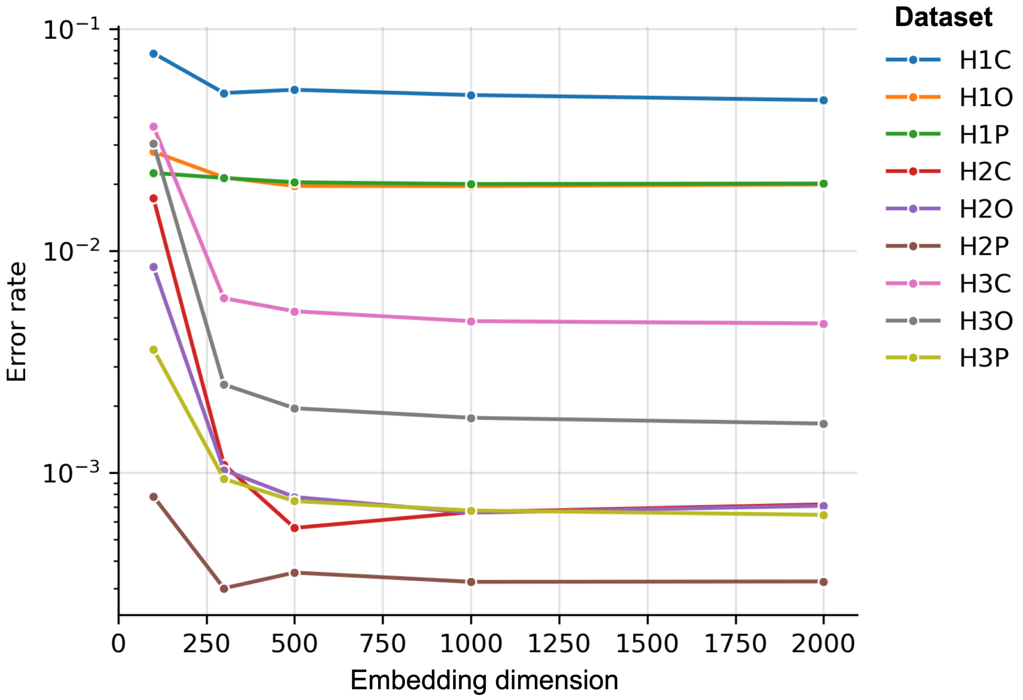

Figure S4. Assessment of embedding dimensions.
Overlap detection error rate of various embedding dimensions. IDF preprocessing was used in feature extraction. Cosine distance was used as metric in k-NN search. Feature matrices were reduced to different dimensions using Sparse Random Projection prior to ENN search. Top six neighbors were used to construct overlap graphs for evaluation.

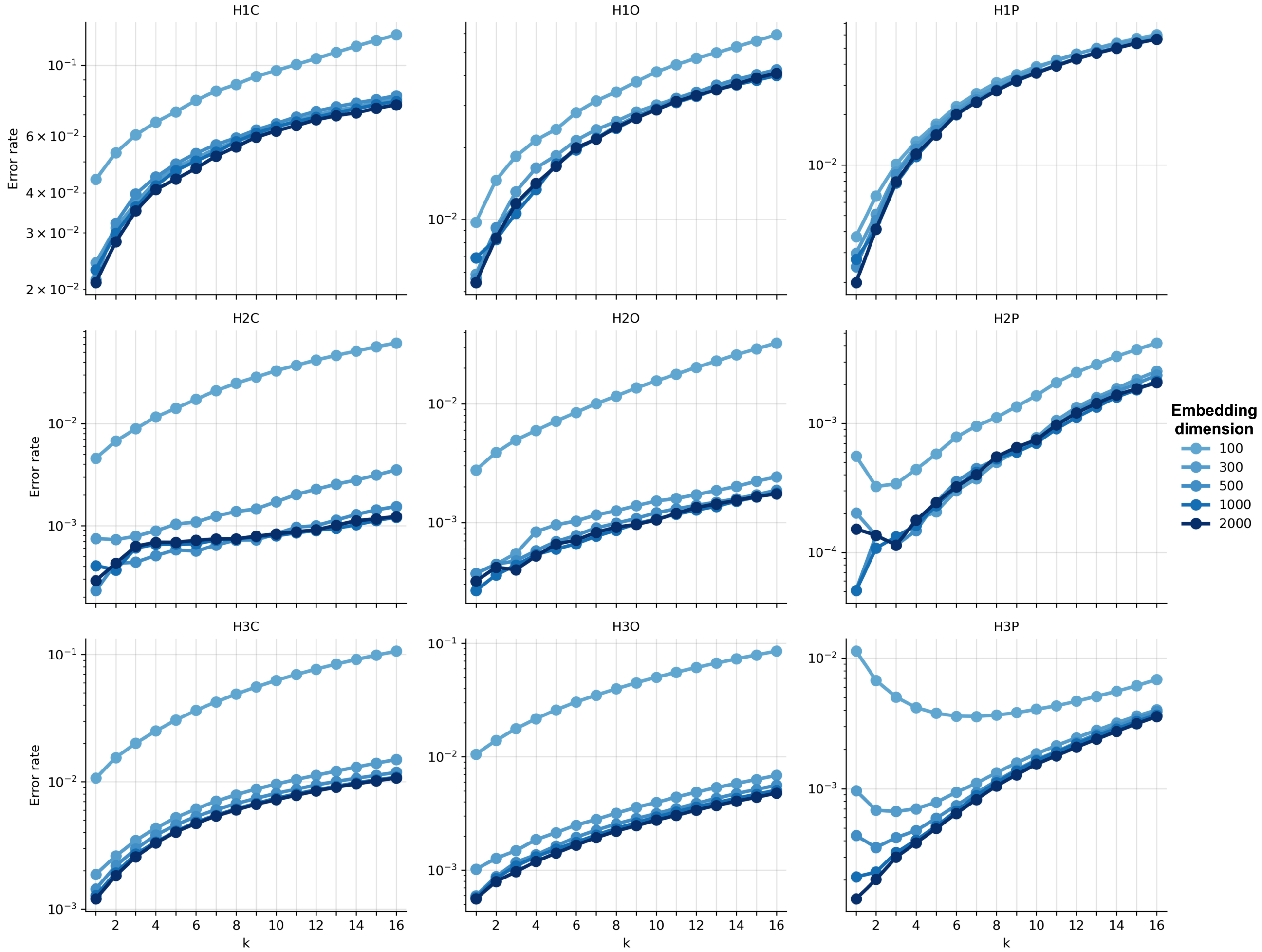

Figure S5. Assessment of embedding dimensions (Detail).
Overlap detection error rate of various embedding dimensions. IDF preprocessing was used in feature extraction. Cosine distance was used as metric in k-NN search. Feature matrices were reduced to different dimensions using Sparse Random Projection prior to ENN search. Each dot represents the overlap detection error rate for a graph constructed using top-k nearest neighbors.

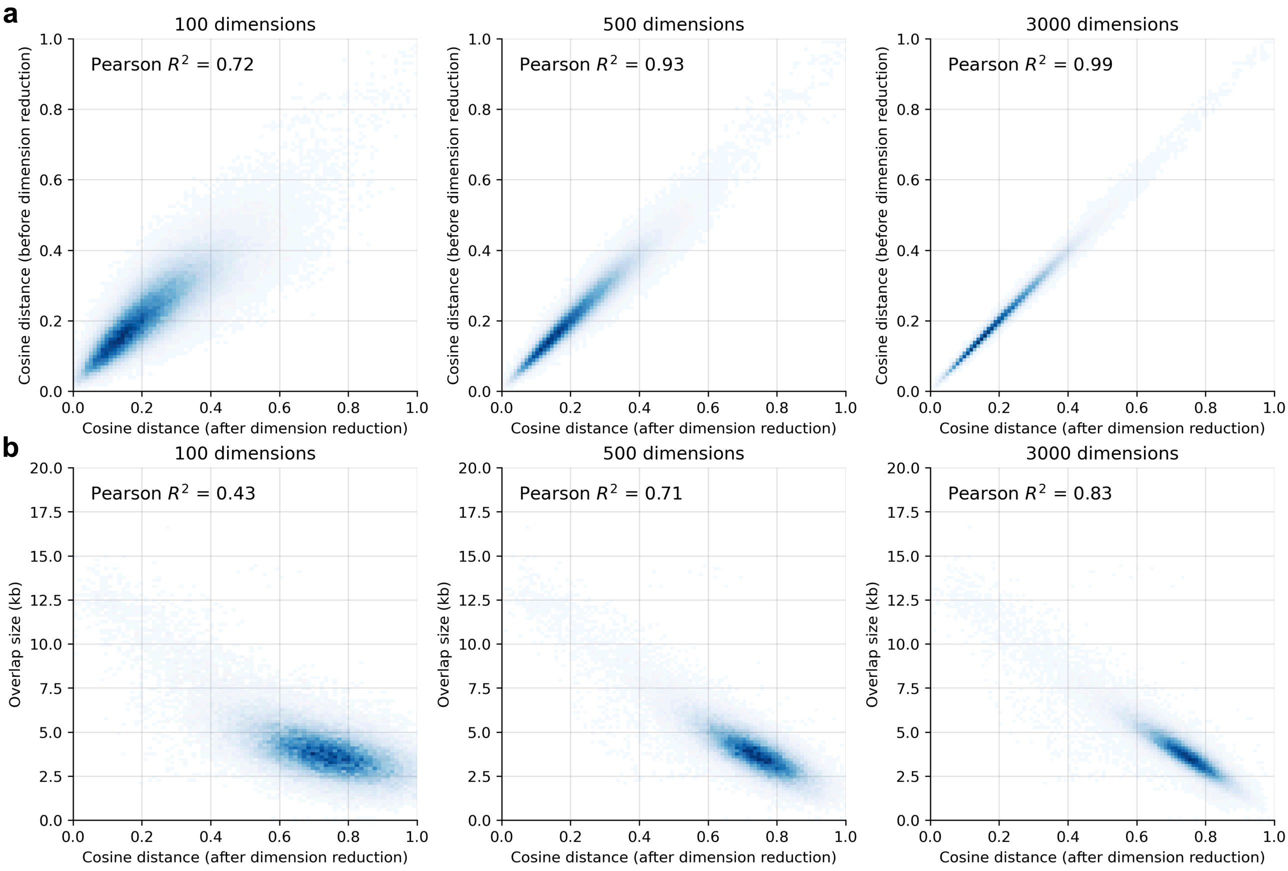

Figure S6. Effect of embedding dimension to distance preservation.
(a) Correlation between distance metrics before/after dimensionality reduction at three embedding dimensions (100,500,3000) for dataset H3P. **(b)** Correlation between cosine distance (after dimensionality reduction) and overlap size at three embedding dimensions (100,500,3000) for dataset H3P.

```
                       INPUT
                         |
                         v
              +-----------------------------------+
              |  FASTQ/FASTA Sequencing Reads    |
              |  (e.g., reads.fasta.gz)          |
              +-----------------------------------+
                         |
                         v
===============================================================
|          STEP 1: K-mer Counting and Sampling               |
===============================================================
                         |
              +-----------------+------------------+
              |                                    |
              v                                    v
+----------------------+              +-------------------------+
|  Jellyfish Count     |              |  Sample k-mers          |
|  - Count all k-mers  |   -------->  |  - Sample fraction      |
|  - Filter by min     |              |  - Generate library     |
|    multiplicity      |              |  - Forward + Reverse    |
+----------------------+              +-------------------------+
                                                 |
                                                 v
                                     +-------------------------+
                                     |  kmer_searcher          |
                                     |  - Find k-mer positions |
                                     |  - Build sparse vectors |
                                     +-------------------------+
                                                 |
                                                 v
===============================================================
|   STEP 2: Dimensionality Reduction Matrix Precomputation   |
===============================================================
                         |
              +-----------------+------------------+
              |                                    |
              v                                    v
+----------------------+              +-------------------------+
|  Compute IDF Weights |              |  Generate Random        |
|  - Calculate inverse |              |  Projection Matrix      |
|    document freq     |   -------->  |  - Sparse Random        |
|  - Weight k-mers by  |              |    Projection (SRP)     |
|    informativeness   |              |  - n_components × n_feat|
+----------------------+              +-------------------------+
                                                 |
                                                 v
                                     +-------------------------+
                                     |  Precompute Matrix      |
                                     |  (SRP_transpose × ICF)  |
                                     +-------------------------+
                                                 |
                                                 v
===============================================================
|          STEP 3: Feature Matrix Generation                 |
===============================================================
                         |
              +-----------------+------------------+
              |                                    |
              v                                    v
+----------------------+              +-------------------------+
|  Parse k-mer Indices |              |  Apply Precompute       |
|  - Read binary file  |   -------->  |  Matrix                 |
|  - Build sparse row  |              |  - Matrix multiplication|
|  - Both strands      |              |  - Get embeddings       |
+----------------------+              +-------------------------+
                                                 |
                                                 v
                                     +-------------------------+
                                     |  Embedding Matrix       |
                                     |  (n_reads × n_dim)      |
                                     |  [Optional: Save]       |
                                     +-------------------------+
                                                 |
                                                 v
===============================================================
|      STEP 4: Approximate Nearest Neighbor Search           |
===============================================================
                      |
                      v
          +-----------------------------+
          |  NNDescent Algorithm        |
          |  - Build k-NN graph index   |
          |  - Graph-based search       |
          |  - Cosine distance metric   |
          +-----------------------------+
                      |
                      v
          +-----------------------------+
          |  Neighbor Indices &         |
          |  Distance Matrix            |
          +-----------------------------+
                      |
                      v
===============================================================
|                        OUTPUT FILES                         |
===============================================================
                          |
       +------------------------+------------------------+
       |                                                 |
       v                                                 v
+----------------------+                  +----------------------+
| overlaps.tsv         |                  | feature_matrix.npz   |
|                      |                  | (optional)           |
| - query_name         |                  |                      |
| - query_orientation  |                  | - Embedding vectors  |
| - target_name        |                  | - Sparse format      |
| - target_orientation|                  |                      |
| - neighbor_rank      |                  +----------------------+
| - distance           |
+----------------------+
```

Figure S7. Schematic overview of the Fedrann workflow

This flowchart summarizes the main stages of the Fedrann pipeline. The pipeline comprises four operational steps implementing three core algorithmic components: feature extraction (steps 1–3), dimensionality reduction (precomputed in step 2 and applied in step 3), and approximate nearest neighbor (ANN) search (step 4). Fedrann employs inverse collection frequency (ICF) as a practical approximation to inverse document frequency (IDF), leveraging Jellyfish for efficient k-mer counting.

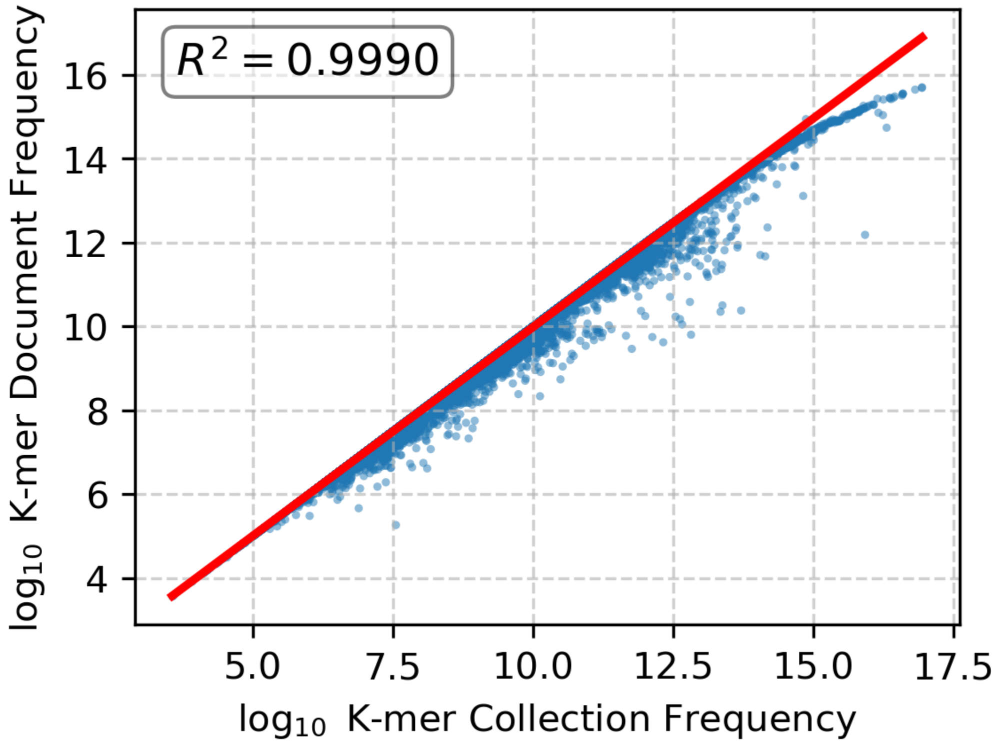

Figure S8. Correlation between k-mer collection frequency and document frequency.
The relationship between the $\log_{10}$-transformed collection frequency and document frequency of K-mers in H4C dataset. The red line represents the linear regression fit.

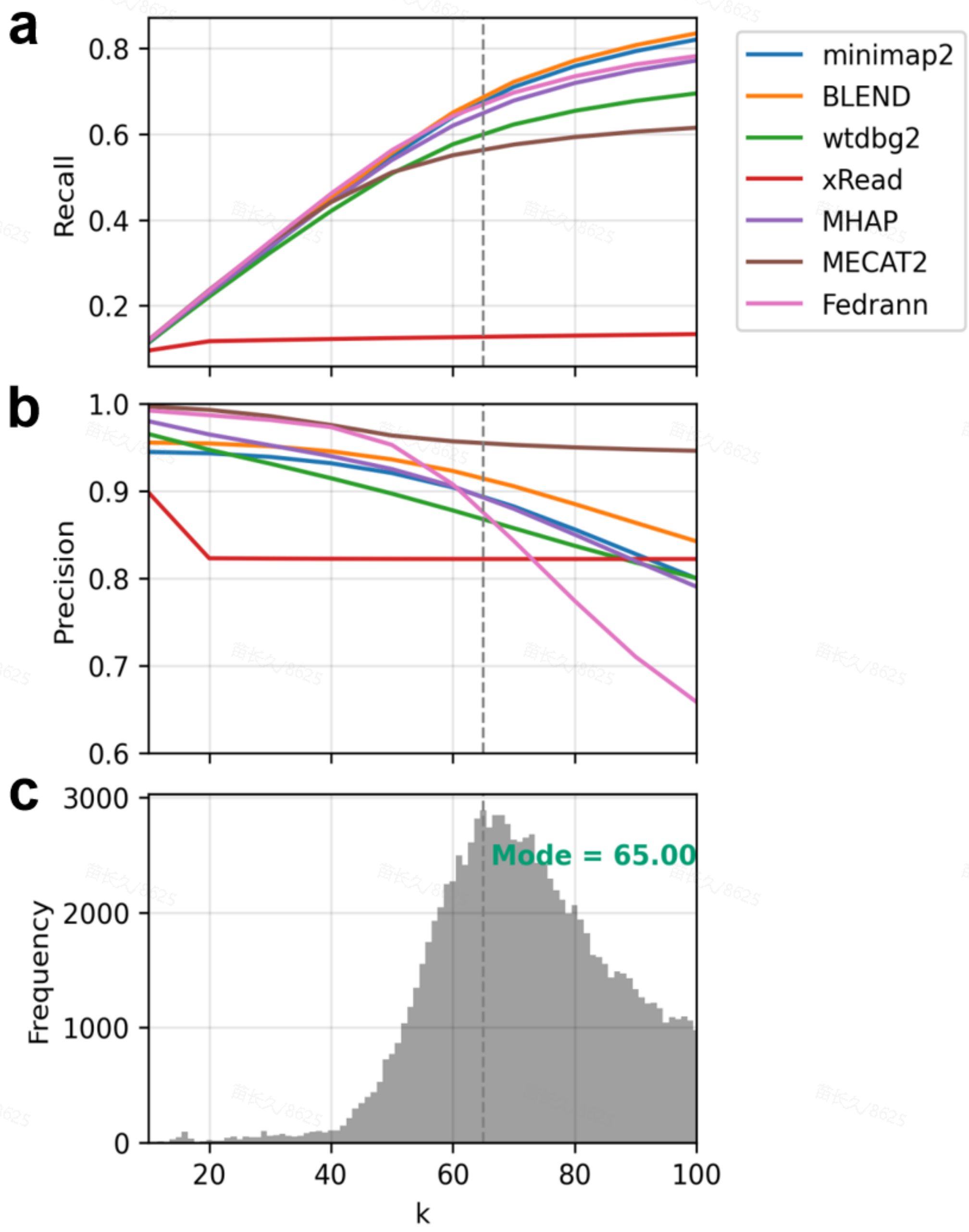

Figure S9. Precision-recall analysis of various overlap detection tools on the H3C dataset.
(a-b) Recall (a) and precision (b) of various overlap detection tools plotted against the number of overlap candidates (*k*). Recall values are calculated as the mean per-read recall across all reads. (c) Distribution of the number of overlapping reads (neighbor count) in the H3C dataset. In (a)-(c), the vertical dashed gray line represents the mode of the neighbor count across all reads in the dataset.

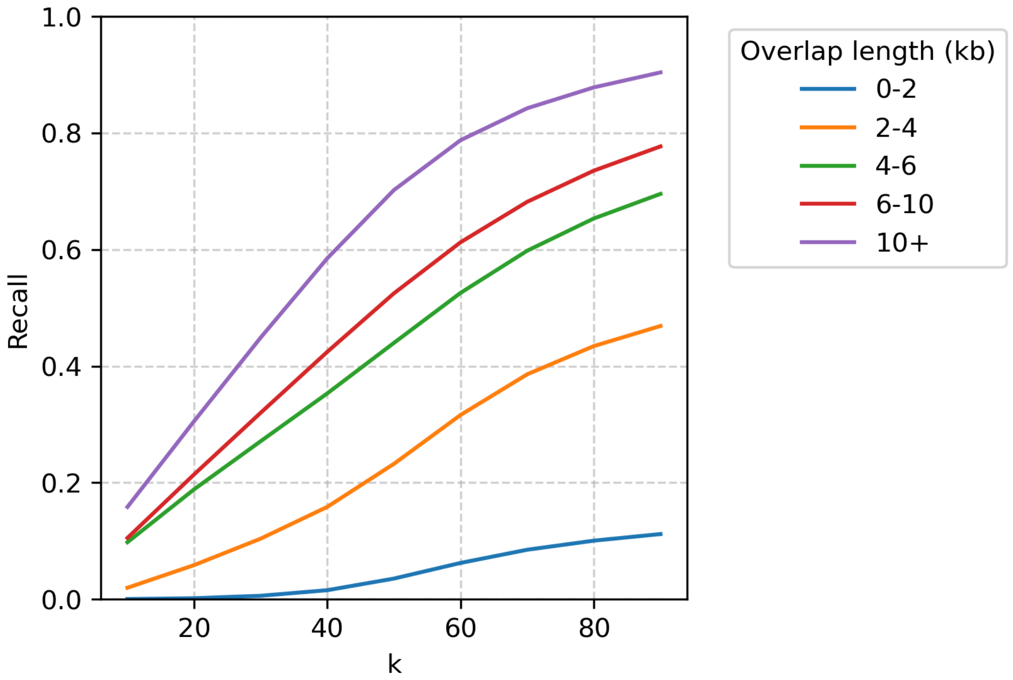

Figure S10. Recall of Fedrann stratified by overlap length on the H3C dataset.
Recall of Fedrann plotted against the number of nearest neighbors (k) for various overlap length intervals. Each colored curve represents the recall rate achieved for a specific range of overlap lengths.

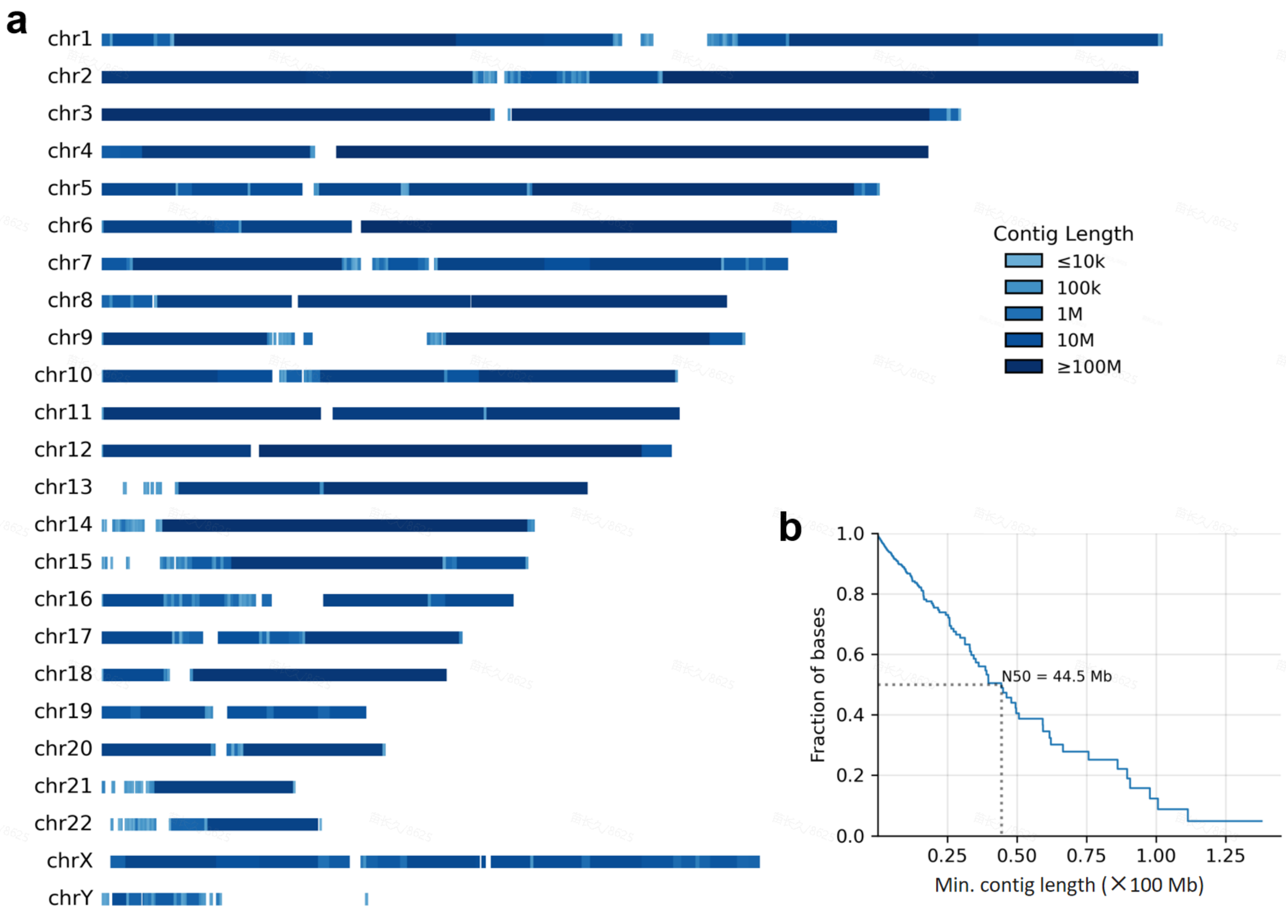

Figure S11. *De novo* assembly based on the Fedrann-Shasta pipeline
(a) Alignment of Fedrann-Shasta assembly to the HG002 reference genome. Darker colors represent longer contigs. **(b)** Cumulative fraction of bases (y axis) plotted against each minimum contig length threshold (x axis) for the Fedrann-Shasta assembly.

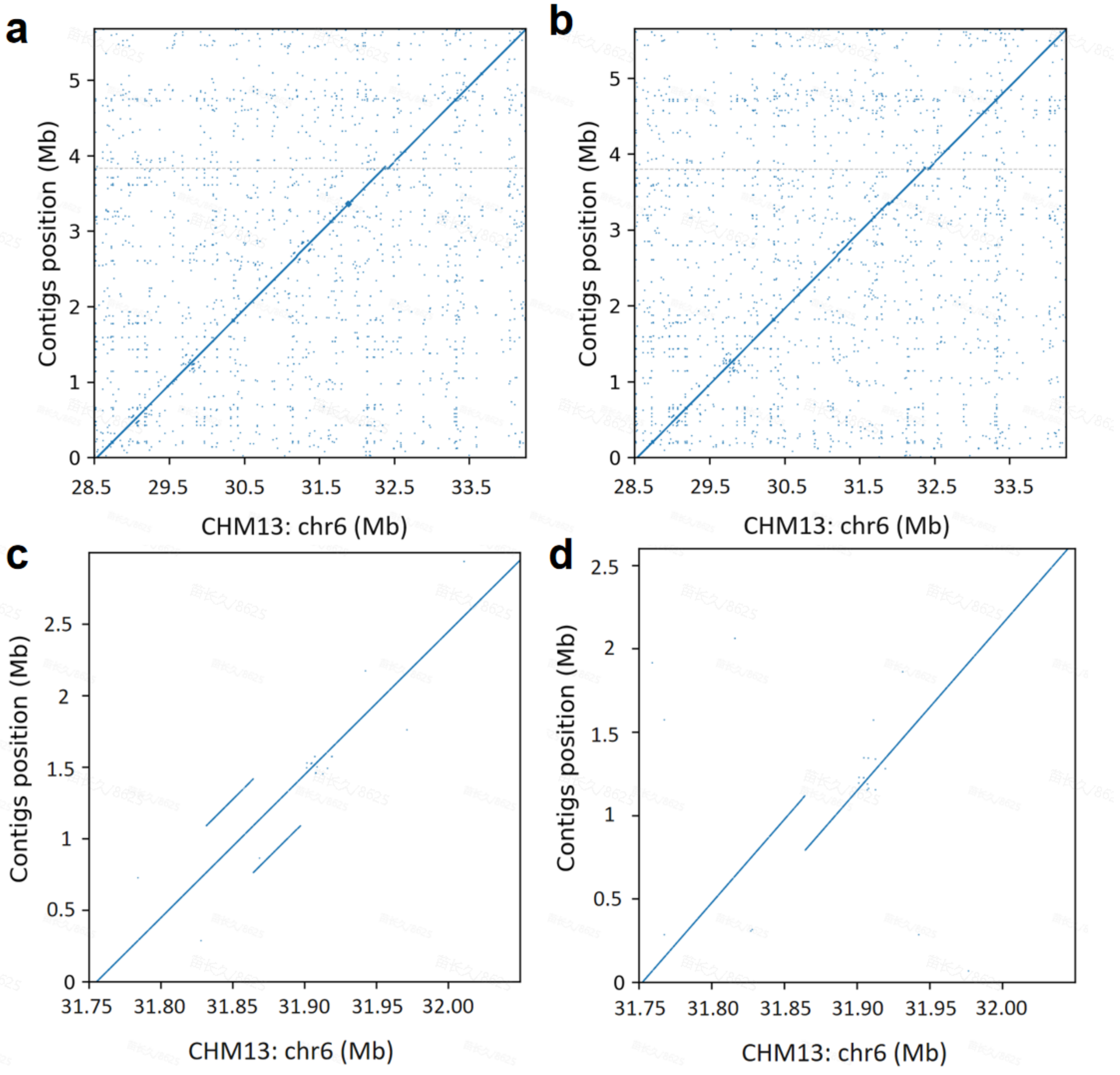

Figure S12. Synteny and collinearity analysis of the HLA region assembly.
(a–b) Dot plots showing the collinearity of the Fedrann-Shasta (a) and standard Shasta (b) assemblies (y-axis) against the T2T-CHM13 reference genome (x-axis). The horizontal dashed grey lines indicate the boundaries between two adjacent contigs. **(c–d)** Magnified views of the regions in (a) and (b), respectively, highlighting a complex segmental duplication locus (chr6: 31.75–32.05 Mb).

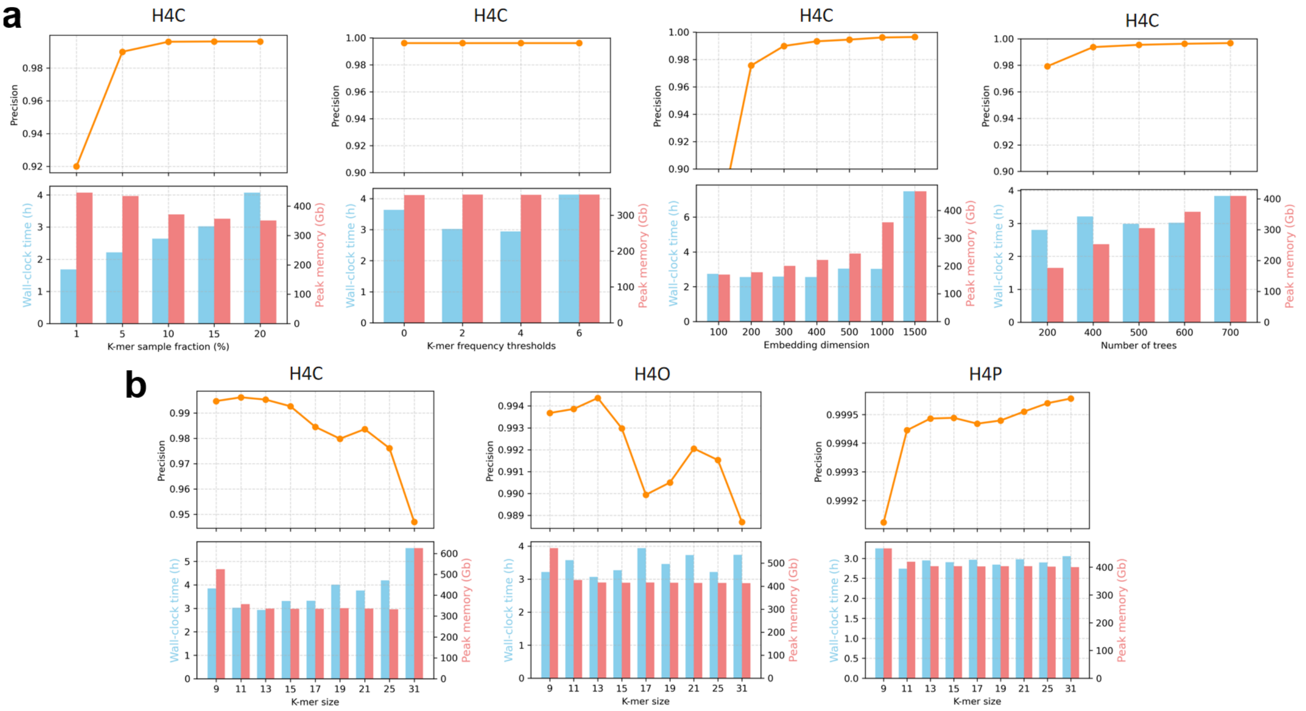

Figure S13. Impact of various parameters to Fedrann performance.
(a) Evaluation of computational efficiency parameters. The plots illustrate the impact of K-mer frequency threshold, K-mer sample fraction, embedding dimension and number of trees on precision (line plots), wall-clock time (blue bars), and peak memory (red bars). (b) Performance across k-mer sizes for different sequencing platforms. The benchmarking was conducted on genome-wide datasets from CycloneSEQ (H4C), ONT (H4O), and PacBio (H4P) platforms.

Supplementary tables

Table S1. Summary of real datasets used in this study.

Dataset	Sample	Region	Platform	Reference size (Mb)	Read count	N50 (kb)
H1C	*H. sapiens* (HG002)	IGK	CycloneSEQ G400-ER	3.92	2,506	41.91
H1O	*H. sapiens* (HG002)	IGK	ONT R10	3.92	2,701	22.97
H1P	*H. sapiens* (HG002)	IGK	PacBio HiFi	3.92	2,624	13.48
H2C	*H. sapiens* (HG002)	HLA	CycloneSEQ G400-ER	5.75	11,176	40.67
H2O	*H. sapiens* (HG002)	HLA	ONT R10	5.75	12,576	23.16
H2P	*H. sapiens* (HG002)	HLA	PacBio HiFi	5.75	12,872	13.42
H3C	*H. sapiens* (HG002)	Chromosome 22	CycloneSEQ G400-ER	51.32	76,601	41.25
H3O	*H. sapiens* (HG002)	Chromosome 22	ONT R10	51.32	81,891	22.78
H3P	*H. sapiens* (HG002)	Chromosome 22	PacBio HiFi	51.32	85,993	13.43
H4C	*H. sapiens* (HG002)	whole genome	CycloneSEQ G400-ER	3,117.29	5,127,109	40.67
H4O	*H. sapiens* (HG002)	whole genome	ONT R10	3,117.29	6,226,490	23.06
H4P	*H. sapiens* (HG002)	whole genome	PacBio HiFi	3,117.29	6,061,187	13.48
C1O	*C. elegans* (strain N2)	whole genome	ONT R9	100.29	221,513	19.02
D1O	*D. melanogaster* (BDGP genome strain)	whole genome	ONT R9	143.73	281,881	23.92

Table S2. Summary of dimensionality reduction methods used in this study. Custom Python implementation of SimHash was used as no existing libraries suitable for analyzing biological sequences were found.

Method	Implementation	Version
scBiMapping	`scBiMapping`	v0.1.0
UMAP	`umap-learn`	v0.5.3
PCA	`sklearn.decomposition.PCA`	v1.2.2
Gaussian Random Projection	`sklearn.random_projection.GaussianRandomProjection`	v1.2.2
Sparse Random Projection	`sklearn.random_projection.SparseRandomProjection`	v1.2.2
Spectral Embedding	`snapatac2.tl.spectral`	v2.1.0
SimHash	Custom Python implementation	–

Table S3. Summary of k-nearest neighbor search methods used in this study.

Method	Implementation	Version	Parameter
brute-force search	`sklearn.neighbors.NearestNeighbors`	v1.2.2	n_jobs = 64
HNSW	`hnswlib`	v0.7.0	M = 512, ef_construction = 200, set_ef = 50, set_num_threads = 64
PQ	`faiss`	v1.8.0	omp_set_num_threads = 64, index param = "PQ128x8"
IVF-PQ	`faiss`	v1.8.0	omp_set_num_threads = 64, M = 128, nlist = 1024,<br>nbits_per_idx = 8, nprobe = 300
RPF	`rpforest`	v1.6	leaf_size = 50, no_trees = 100
NNDescent	`pynndescent`	v0.5.12	n_jobs = 64, n_trees = 600, leaf_size = 200, index_n_neighbors = 50

Table S4. Fedrann parameters used for benchmarking.

Dataset	Feature extraction	Dimensionality reduction	ANN search
H4C	k-mer size = 11; k-mer sample fraction = 0.15	SRP (embedding dimension = 1000)	NNDescent (number of trees = 600)
H4O	k-mer size = 13; k-mer sample fraction = 0.15	SRP (embedding dimension = 1000)	NNDescent (number of trees = 600)
H4P	k-mer size = 31; k-mer sample fraction = 0.15	SRP (embedding dimension = 1000)	NNDescent (number of trees = 600)
C1O	k-mer size = 21; k-mer sample fraction = 0.10	SRP (embedding dimension = 1000)	NNDescent (number of trees = 600)
D1O	k-mer size = 21; k-mer sample fraction = 0.15	SRP (embedding dimension = 2000)	NNDescent (number of trees = 600)

Table S5. Command-line arguments of various tools used for benchmarking.

Tool	Dataset platform	Arguments
xRead	CycloneSEQ/ONT	-k 19 -w 40 -p 2 -t 64
xRead	PacBio	-k 19 -w 40 -p 2 -t 64
MECAT2	CycloneSEQ/ONT/PacBio	-t 64
BLEND	CycloneSEQ/ONT	-x ava-ont -t 64
BLEND	PacBio	-x ava-hifi -t 64
wtdbg2	CycloneSEQ/ONT	-p 0 -k 15 -AS 2 -s 0.05 -t 64
wtdbg2	PacBio	-p 21 -k 0 -AS 4 -K 0.05 -s 0.5 -L 1000 -t 64
minimap2	CycloneSEQ/ONT	-x ava-ont -t 64
minimap2	PacBio	-x ava-pb -t 64
MHAP	CycloneSEQ/ONT/PacBio	–repeat-weight 0.9 –repeat-idf-scale 10 -k 16 –store-full-id –num-hashes 768 –num-min-matches 20 –threshold 0.73 –filter-threshold 0.0000001 –ordered-sketch-size 1536 –ordered-kmer-size 12 –min-olap-length 500

Table S6. *De novo* assembly statistics of Fedrann-Shasta and Shasta

Method	Fedrann-Shasta	Shasta	Fedrann-Shasta	Shasta
Dataset	H4C	H4C	H4O	H4O
Total assembly length (Gb)	2.84	2.85	2.81	2.80
N50 (Mb)	44.45	45.07	17.07	5.73
NGA50 (Mb)	8.00	8.23	5.71	3.46
Largest contig length (Mb)	138.00	137.99	97.89	29.09
K-mer QV	35.74	35.71	37.51	37.58
K-mer completeness (%)	95.56	95.75	95.42	94.94
BUSCO completeness (%)	98.10	98.30	97.80	97.00

Table S7. Performance of Fedrann under various CPU and memory limitations.
Benchmarks were conducted on dataset H4C within Docker containers to simulate hardware constraints. RAM: random access memory. Prec.: precision. HDD: hard disk drive. SSD: solid state drive.

Threads	Memory Limit	Swap Medium	Wall-clock Time (h)	Peak RAM (GB)	Prec. (k=6)	Prec. (k=12)	Prec. (k=18)
64	2 TB	HDD	3.02	357.45	99.62%	99.44%	99.25%
64	256 GB	HDD	15.69	250.73	99.62%	99.44%	99.24%
32	230 GB	SSD	14.87	228.10	99.62%	99.44%	99.25%
64	128 GB	HDD	Failed	—	—	—	—

Click here to access/download;Figure;algorithm_overview.png

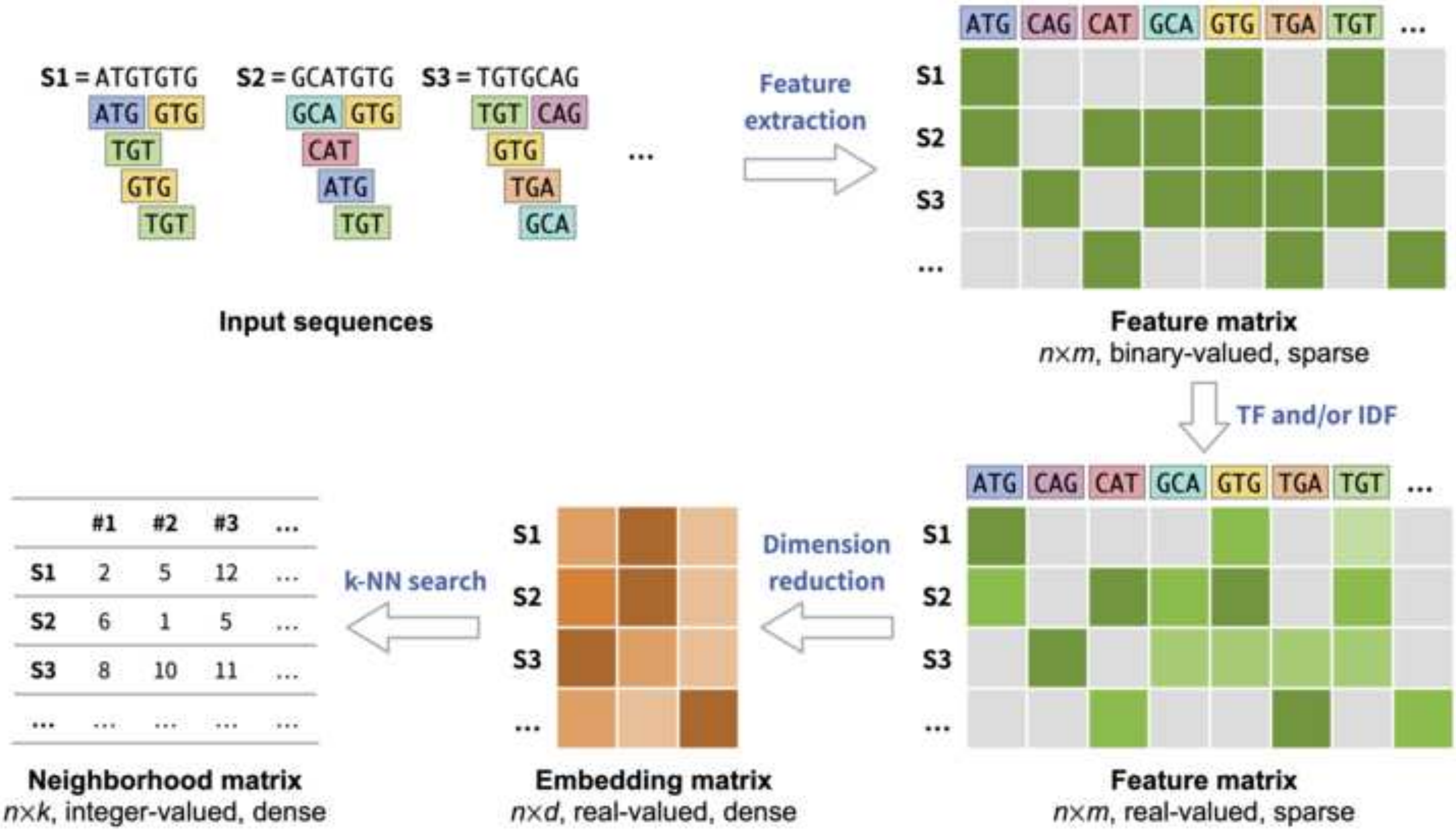

Click here to access/download;Figure;ann_main.png

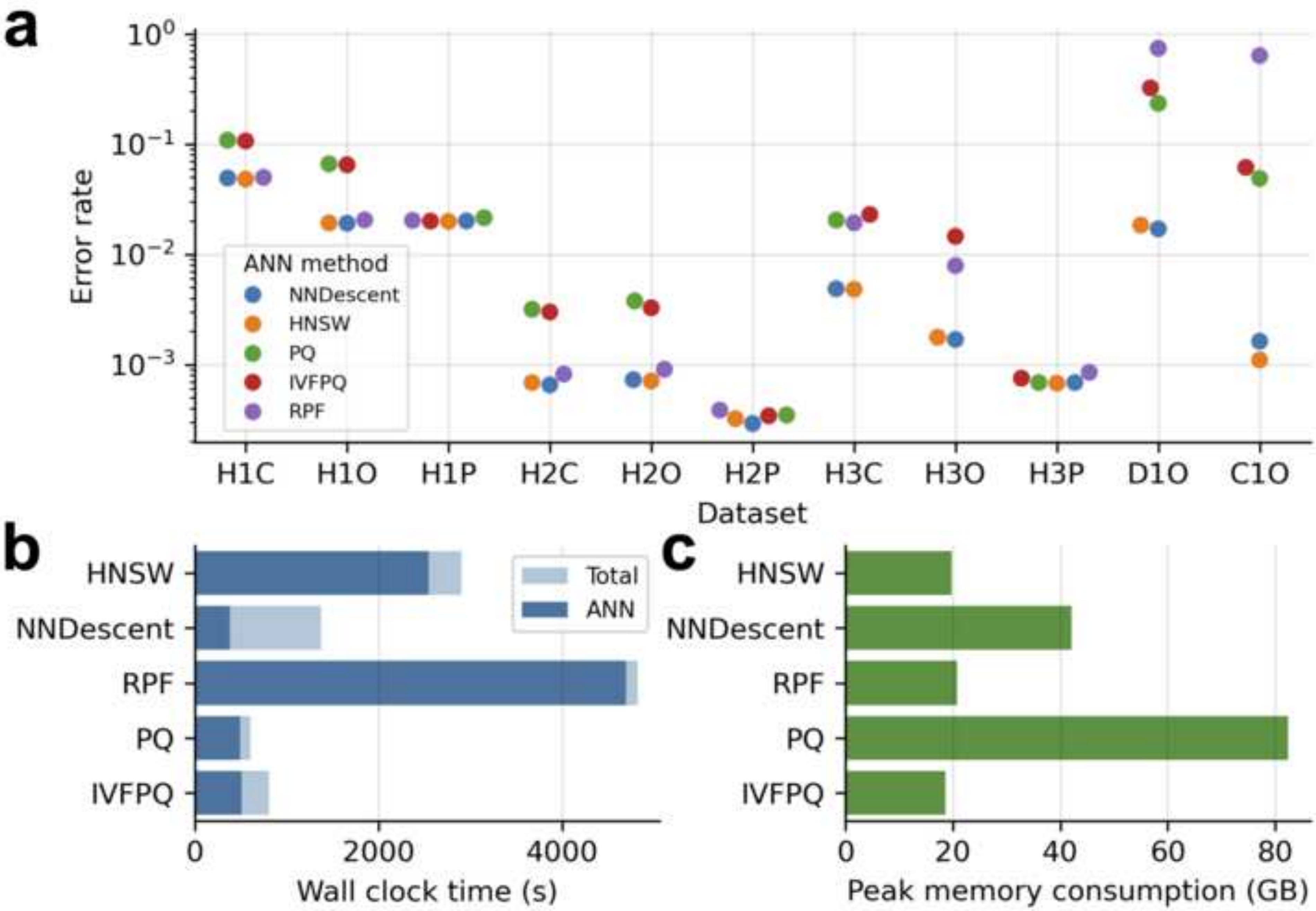

Click here to access/download;Figure;CF_DF_correlation.png

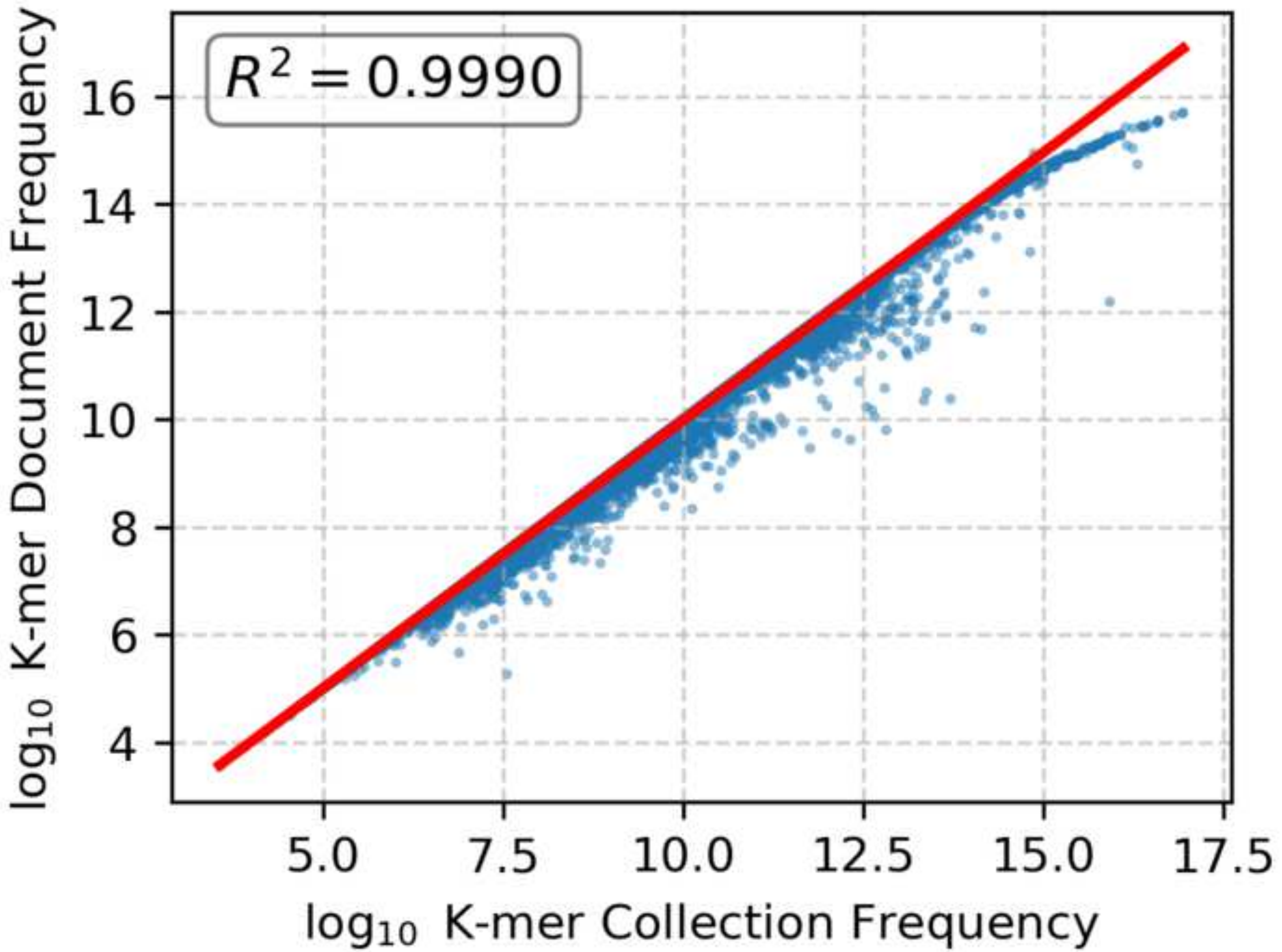

Click here to access/download;Figure;dimenality_reduction_main.png

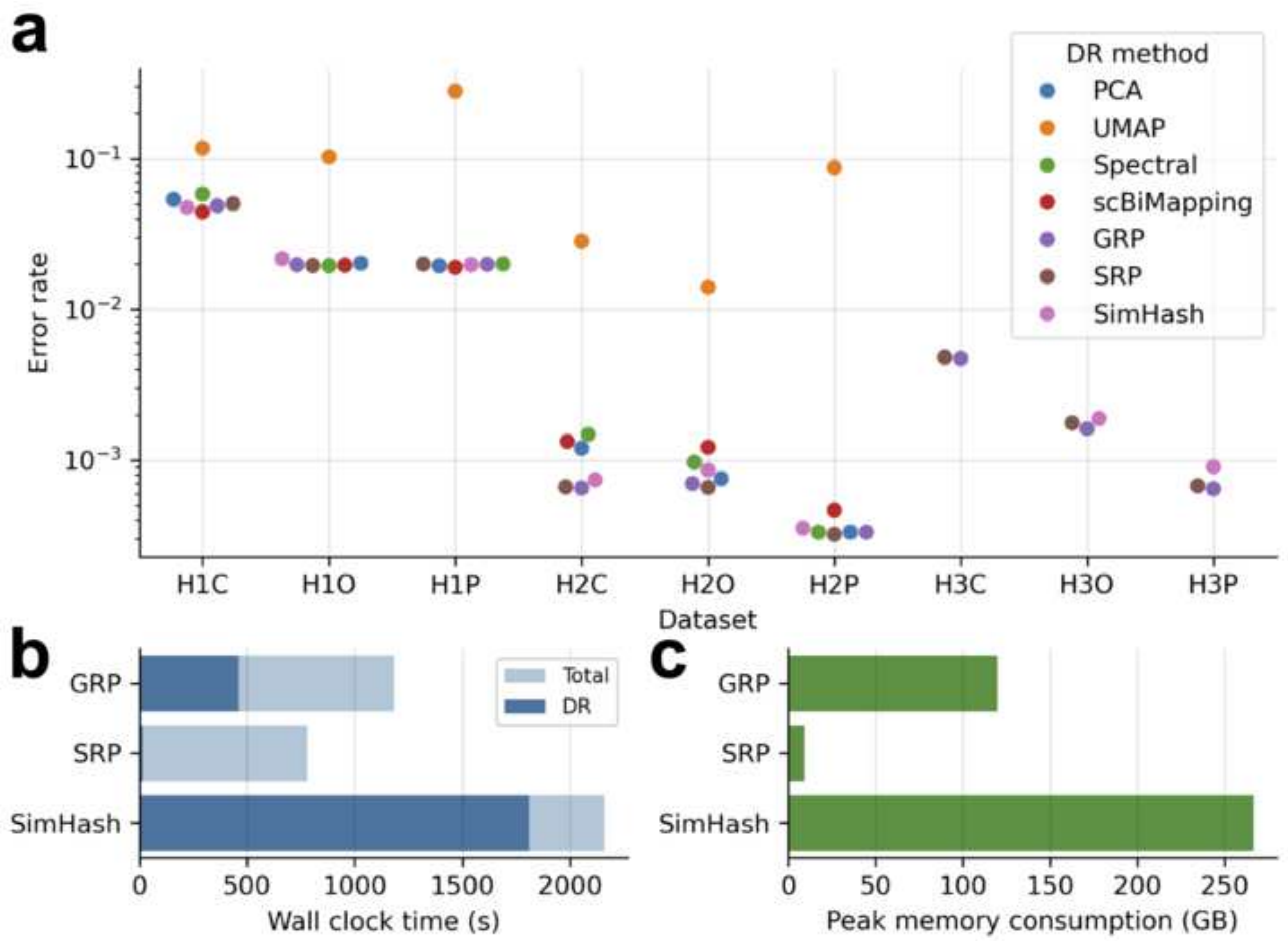

Click here to access/download;Figure;distance_correlation.png

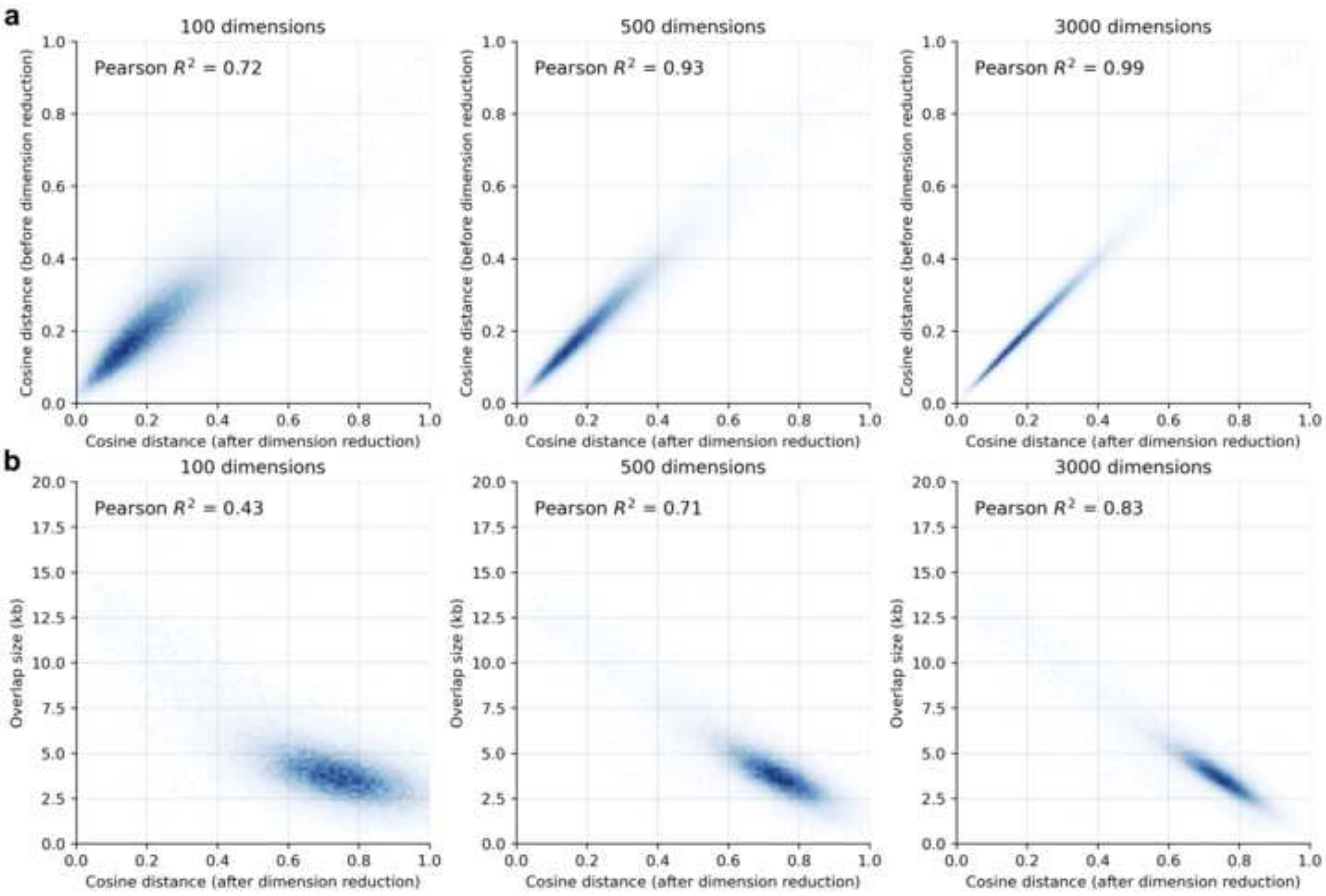

Click here to access/download;Figure;embedding_dimension_detail.png

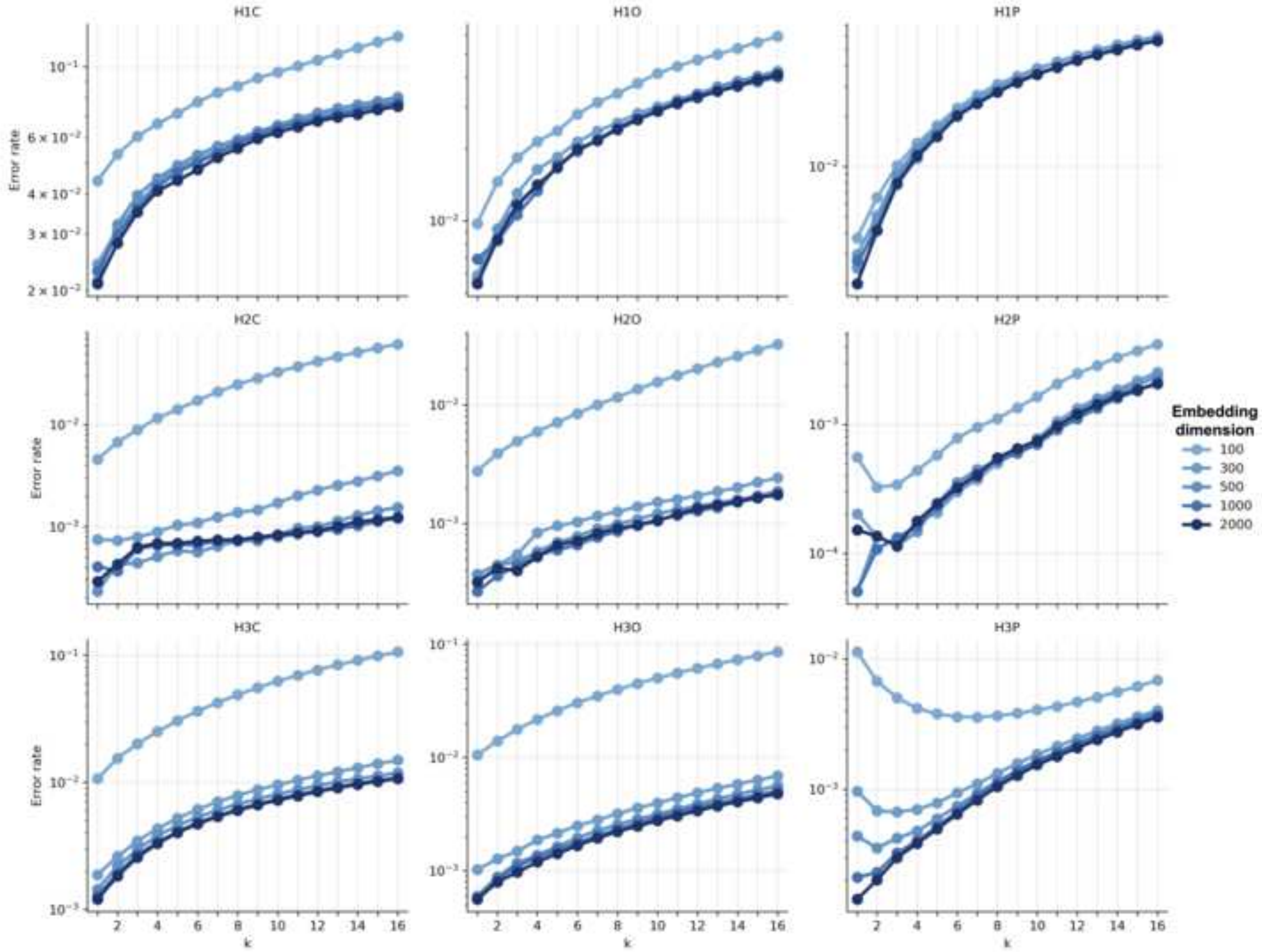

Click here to access/download;Figure;embedding_dimension_error_rate.png

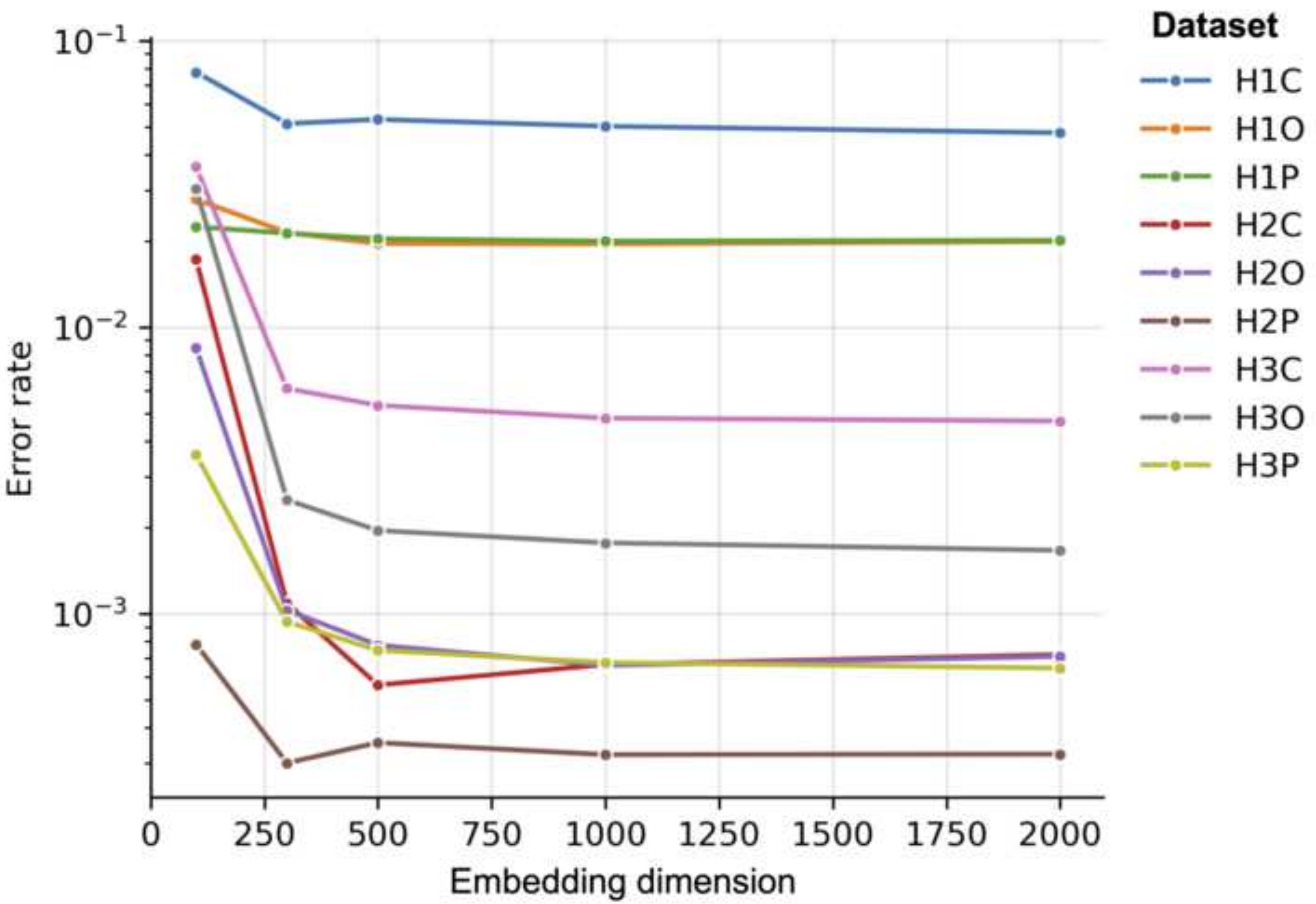

Click here to access/download;Figure;evaluation_pipeline.png

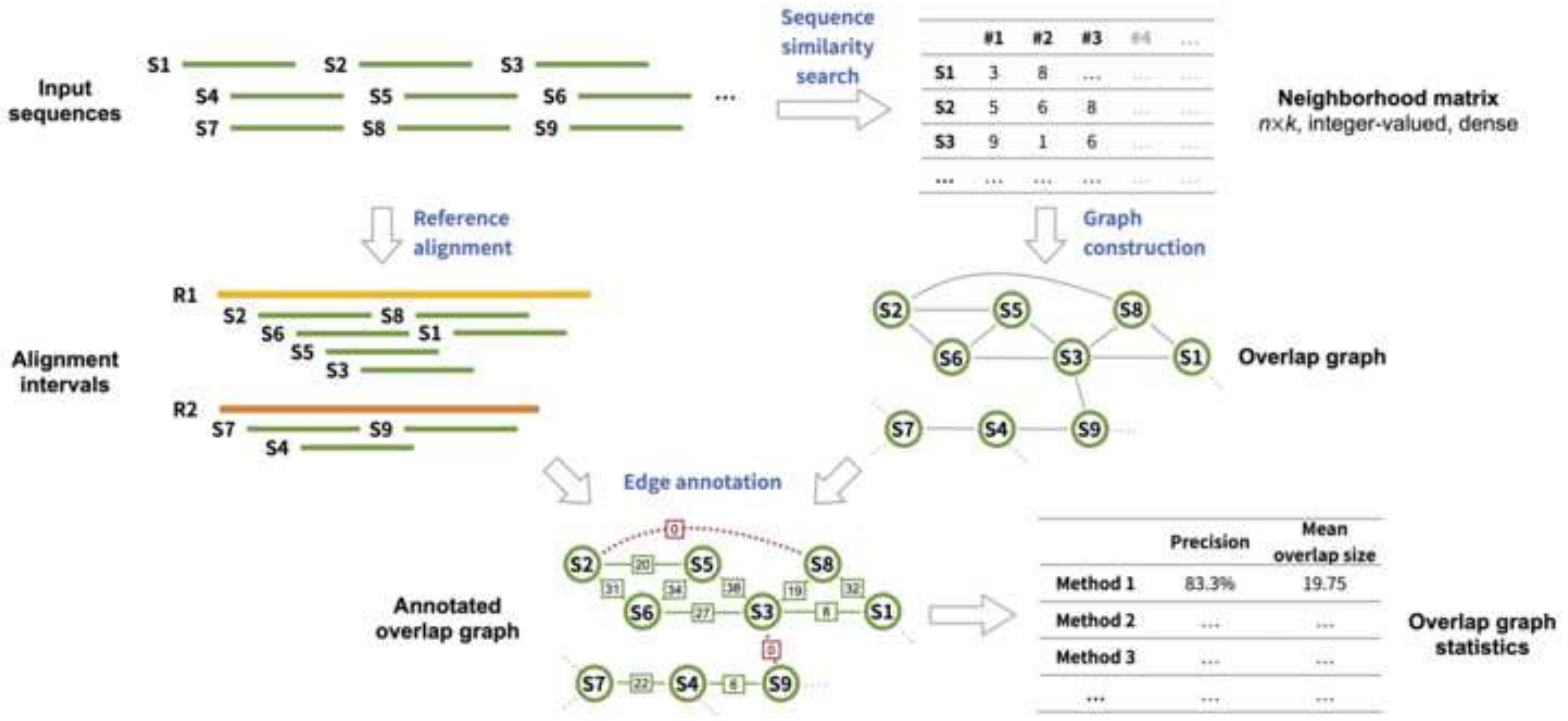

Click here to access/download;Figure;feature_extraction.png

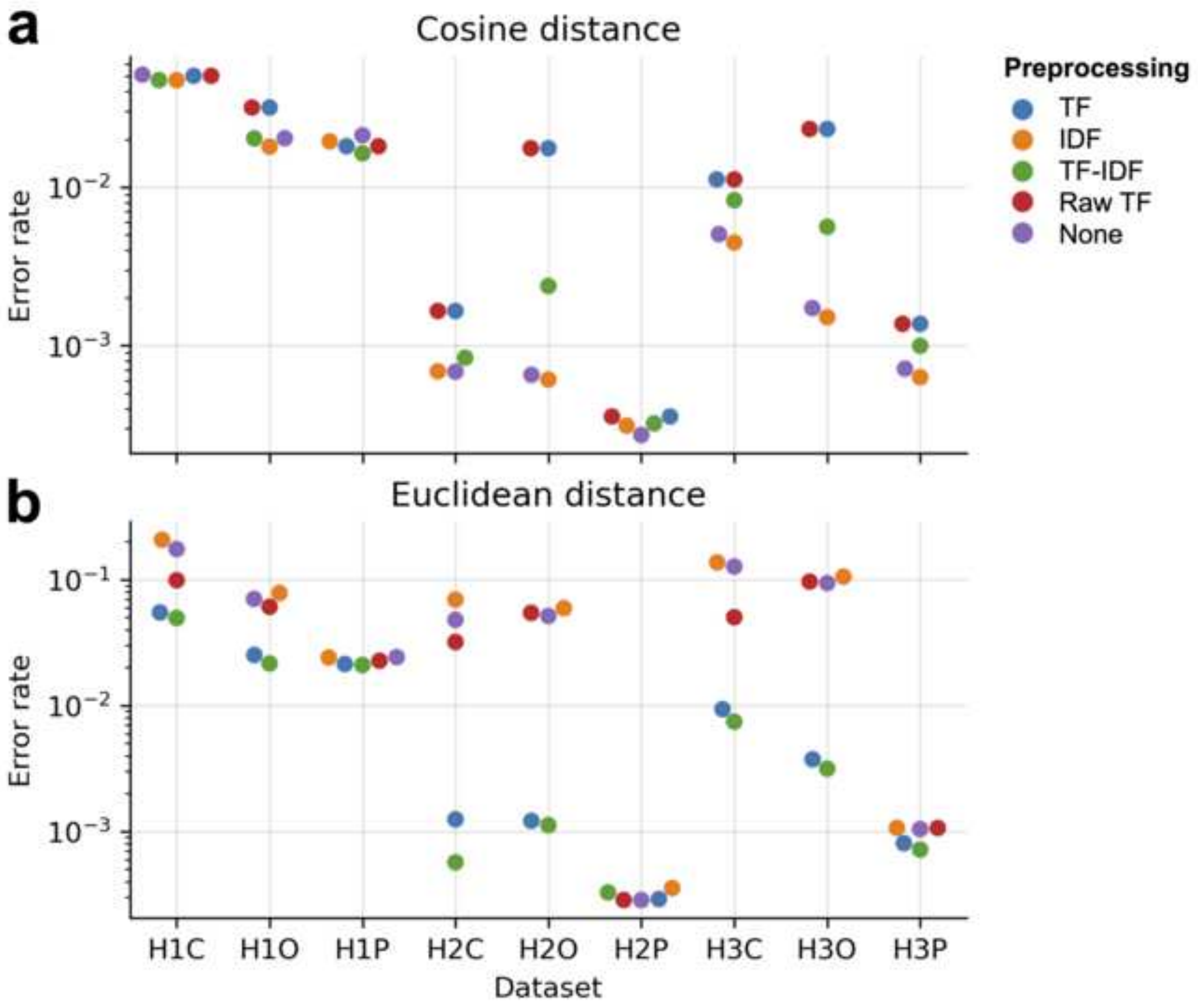

Click here to access/download;Figure;fedrann_workflow.png

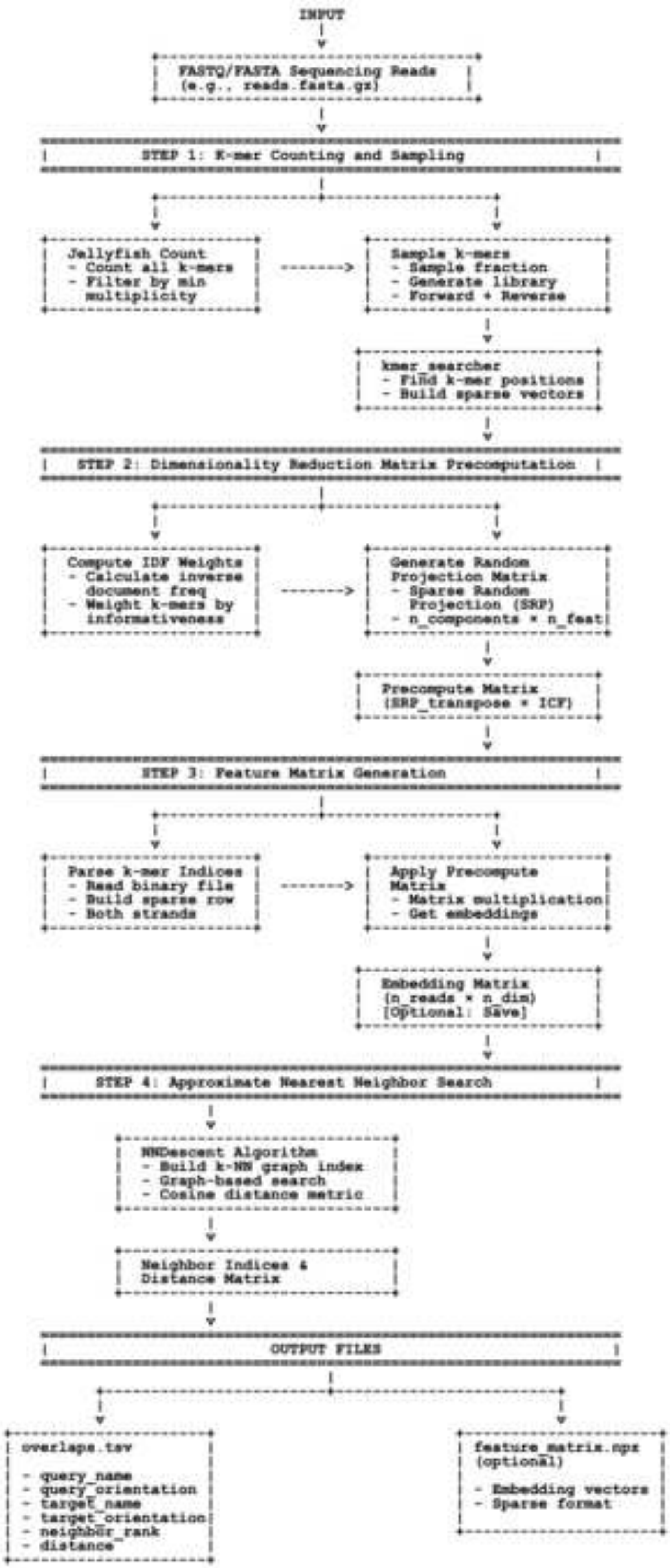

Click here to access/download;Figure;Fedrann-Shasta.png

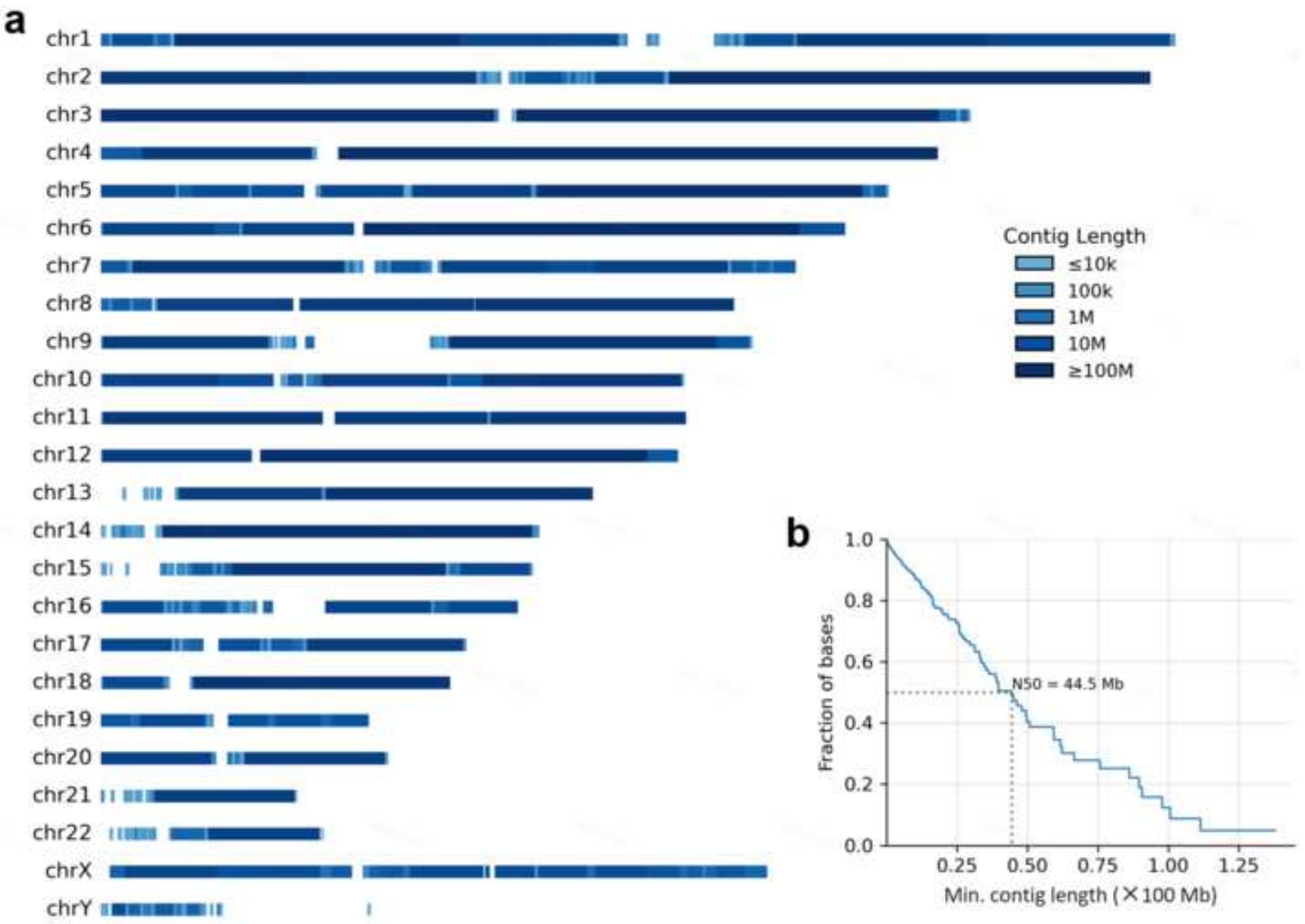

Click here to access/download;Figure;genome_benchmarking.png

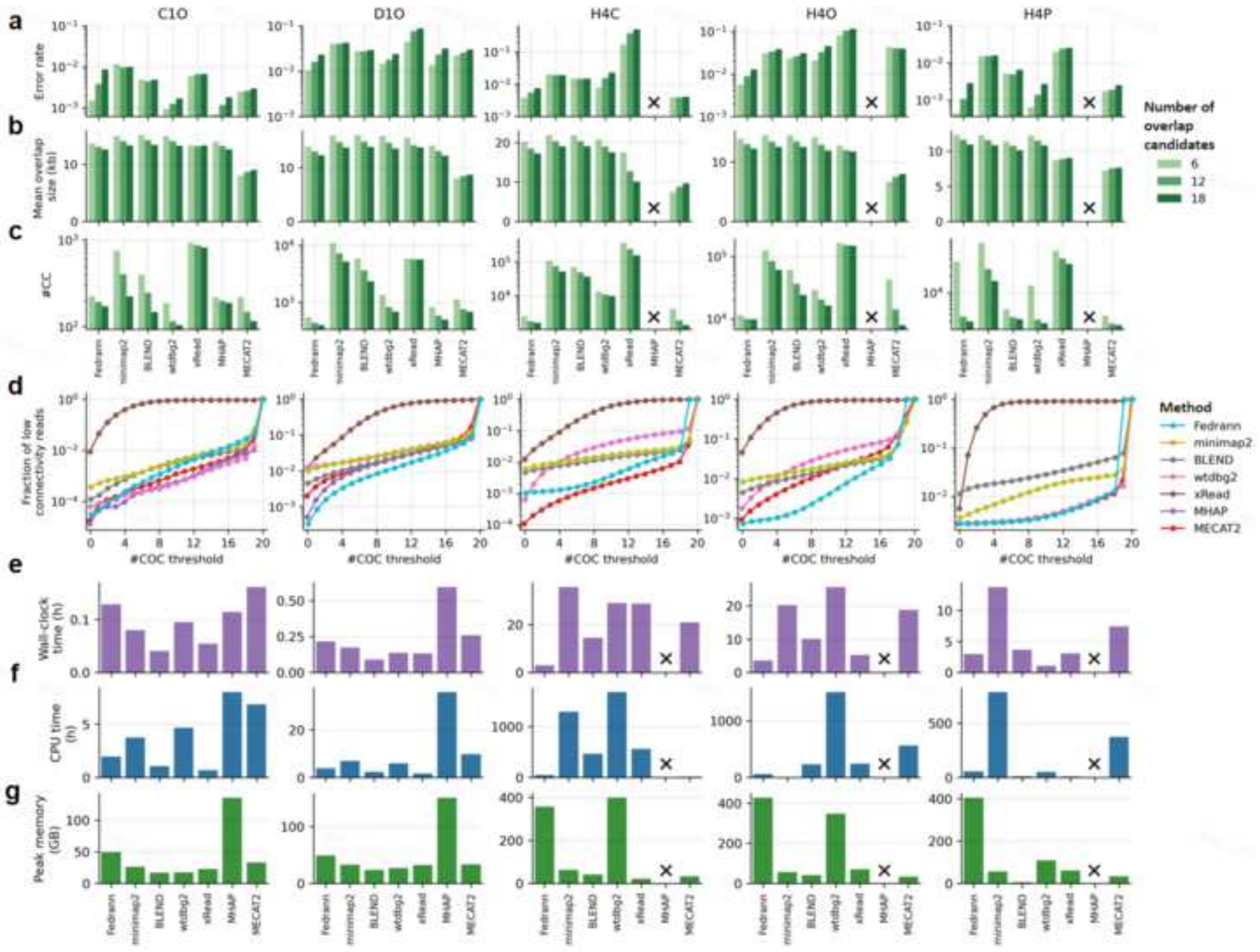

Click here to access/download;Figure;HLA_dot_plot.png

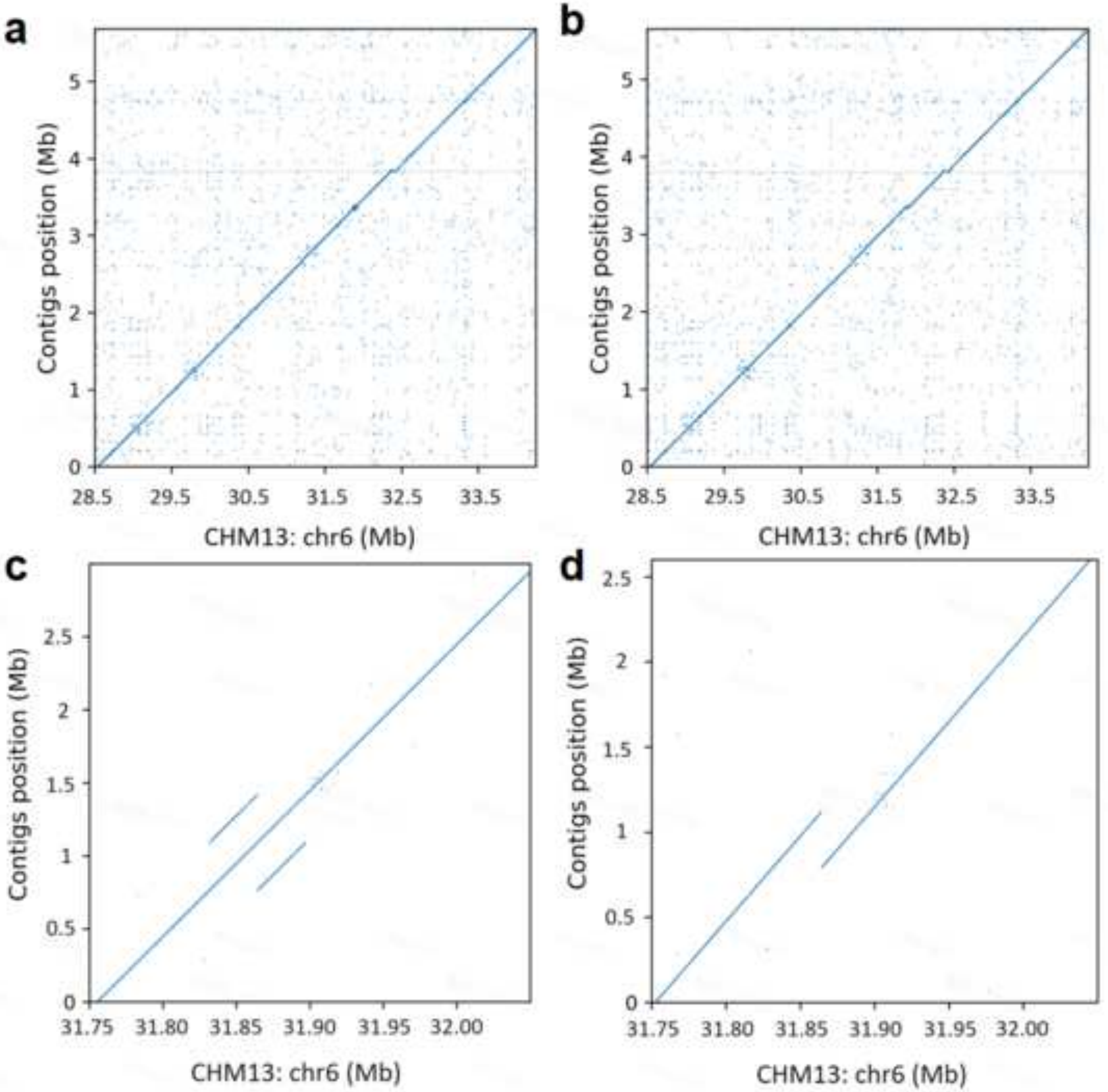

Click here to access/download;Figure;length_depth_accuracy.png

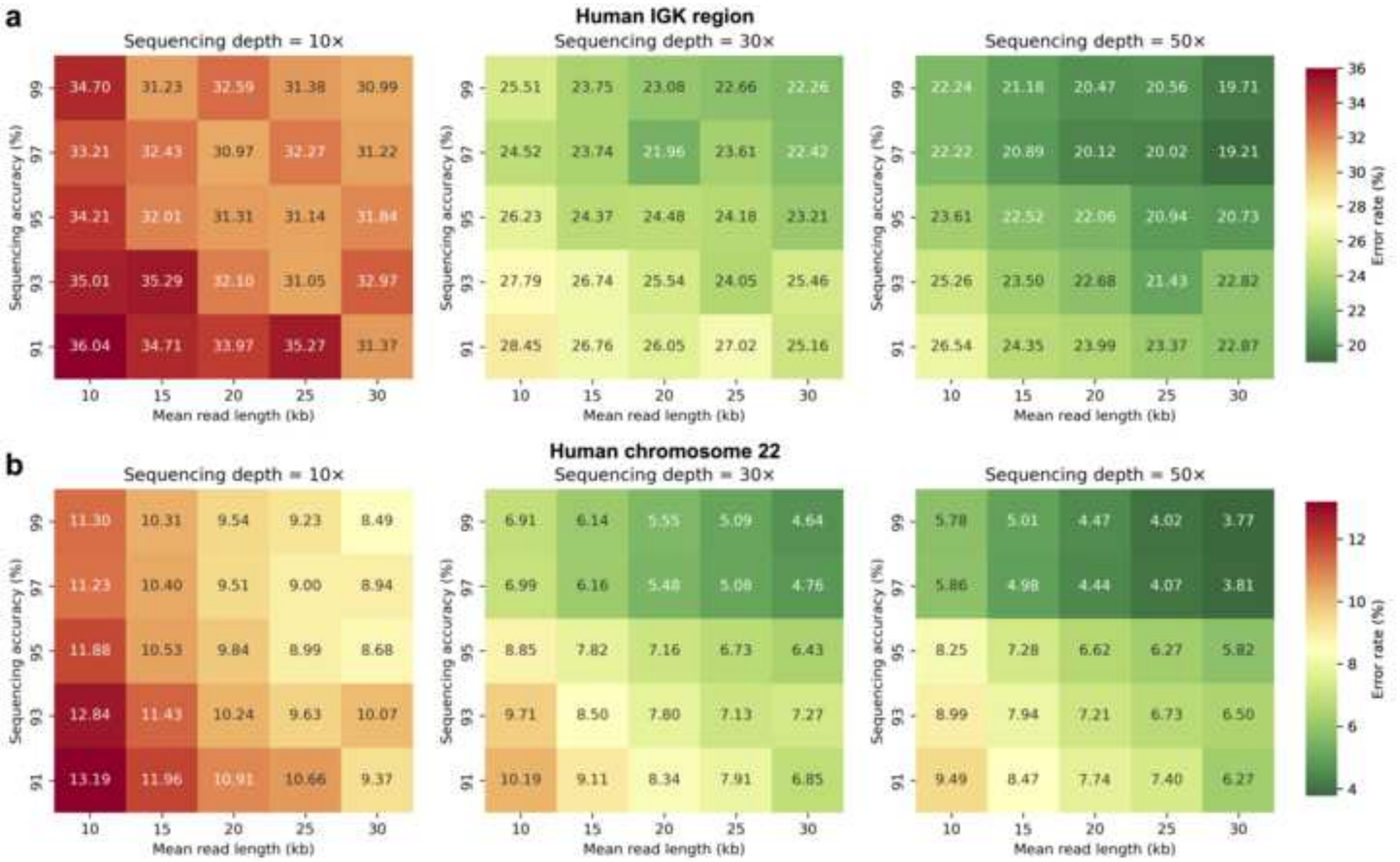

Click here to access/download;Figure;parameter_tuning.png

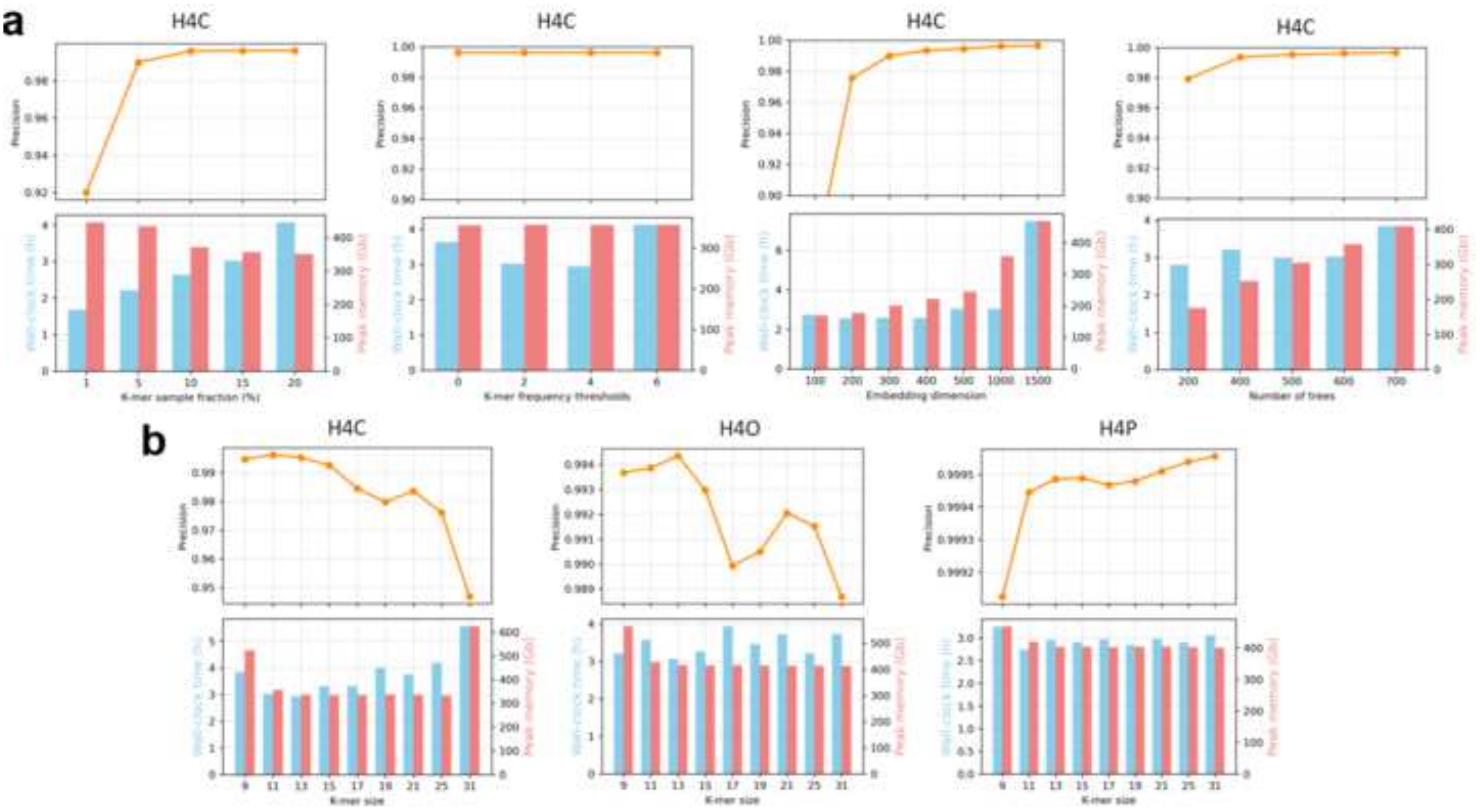

Click here to access/download;Figure;preprocessing_box_plot.png

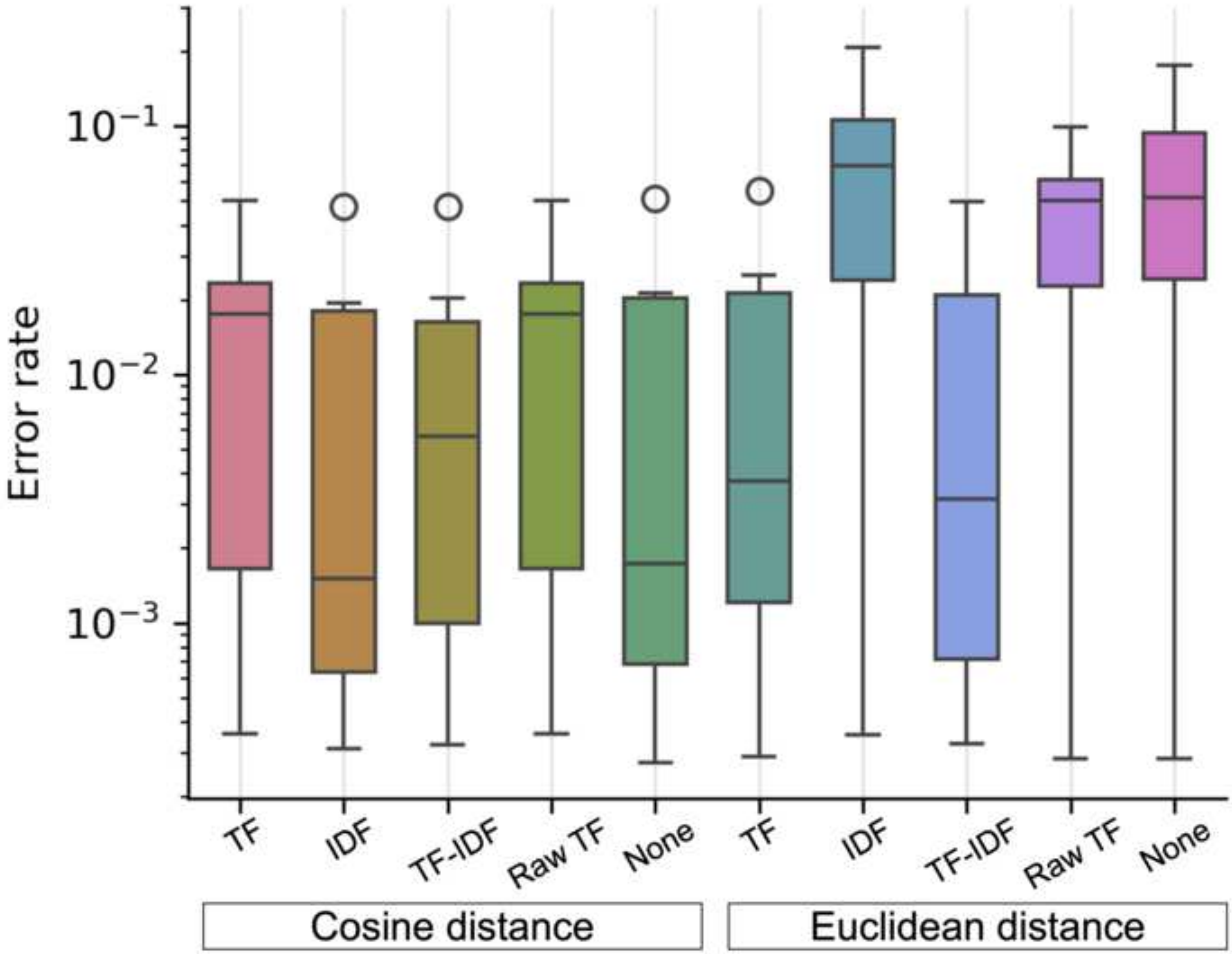

Click here to access/download;Figure;preprocessing_error_rate.png

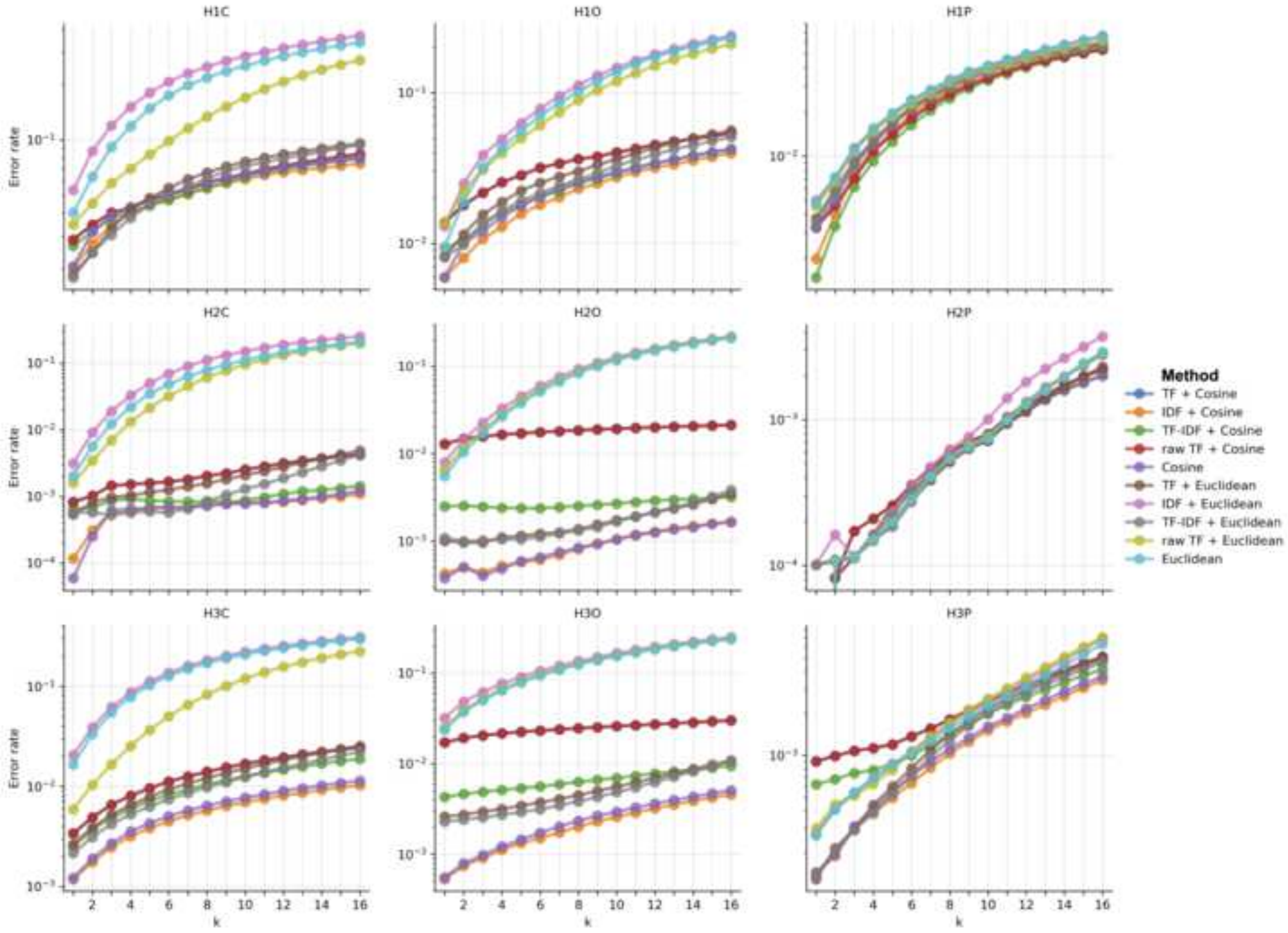

Click here to access/download;Figure;preprocessing_main.png

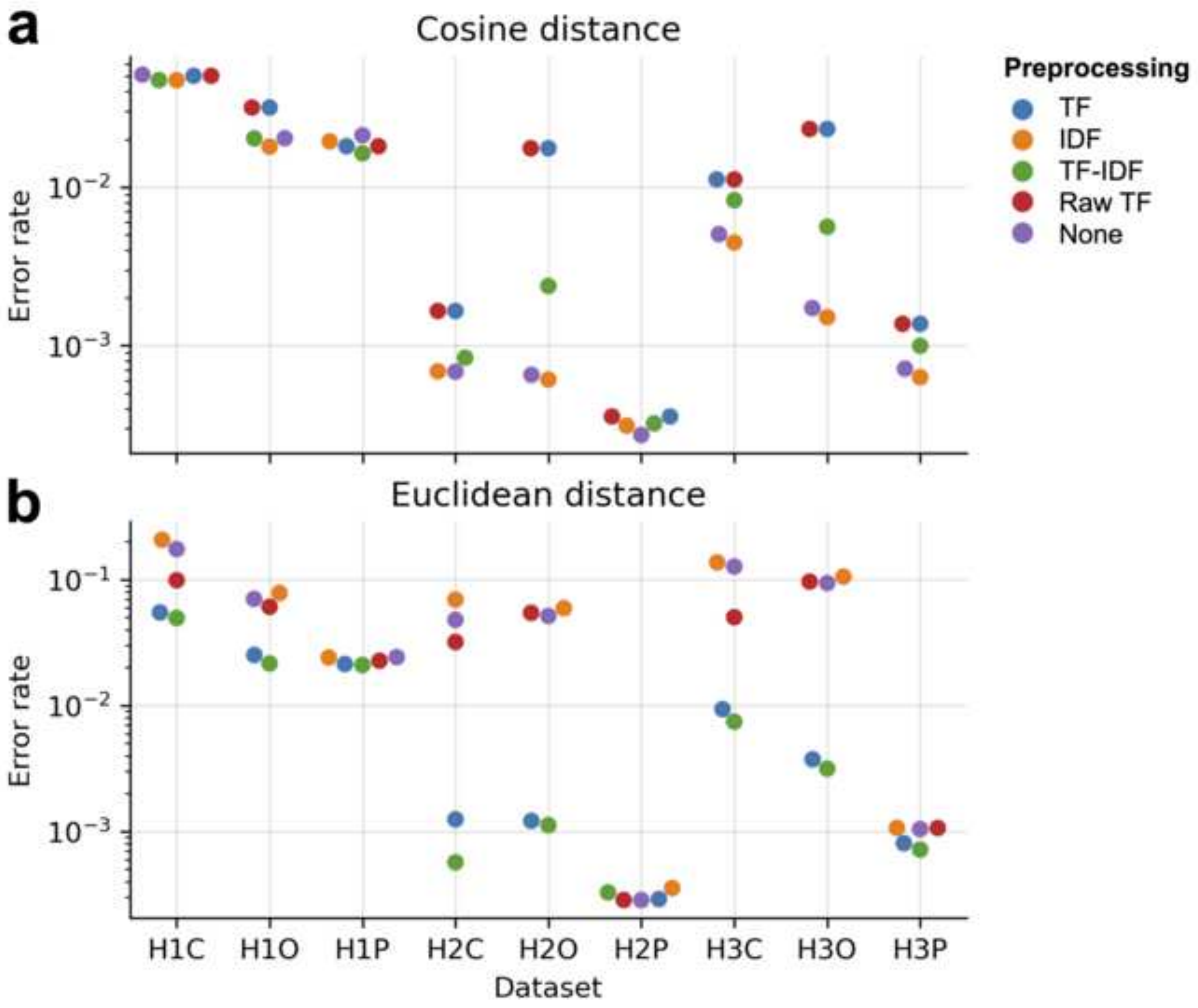

Click here to access/download;Figure;recall-precision.png

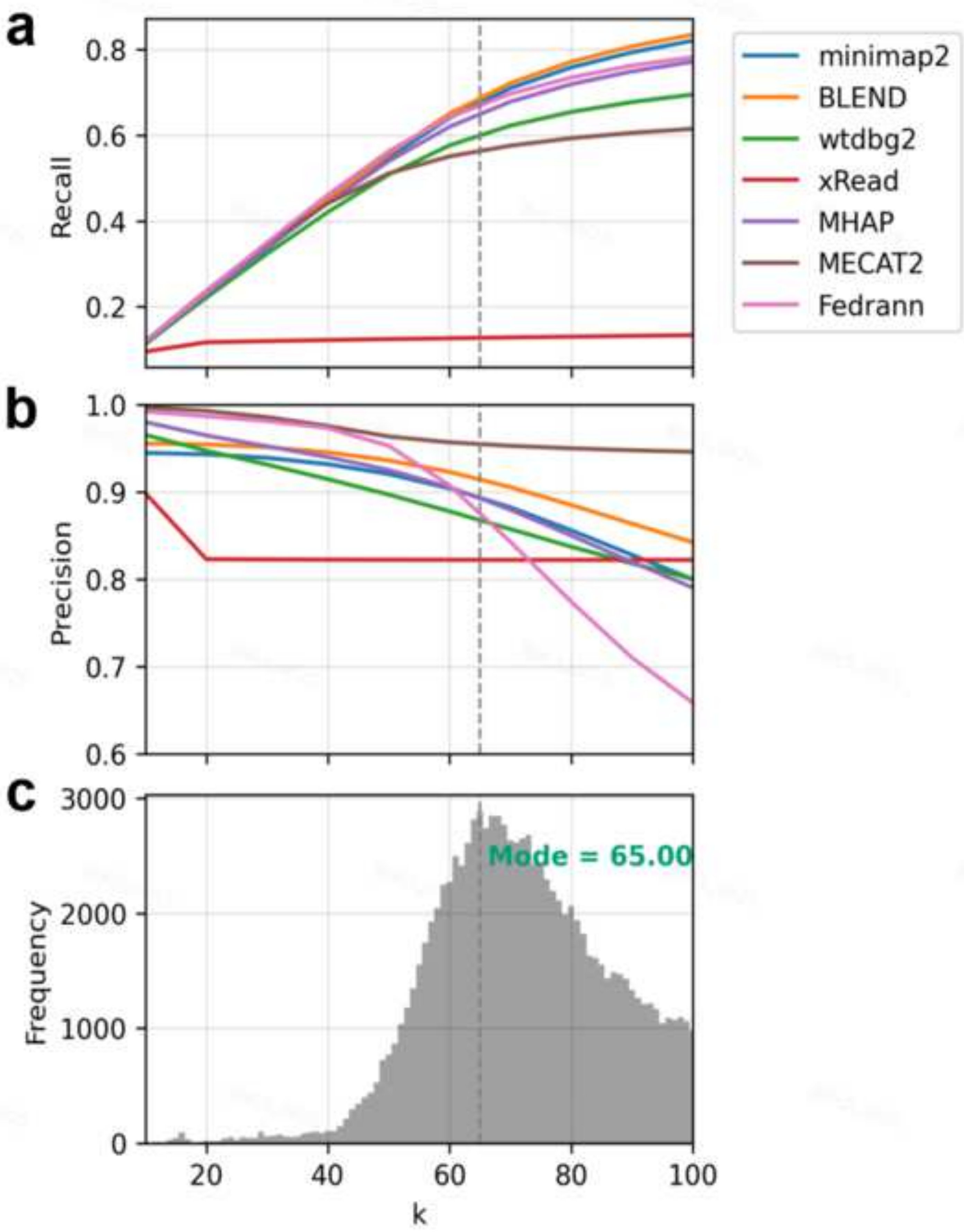

Click here to access/download;Figure;stratified_recall.png

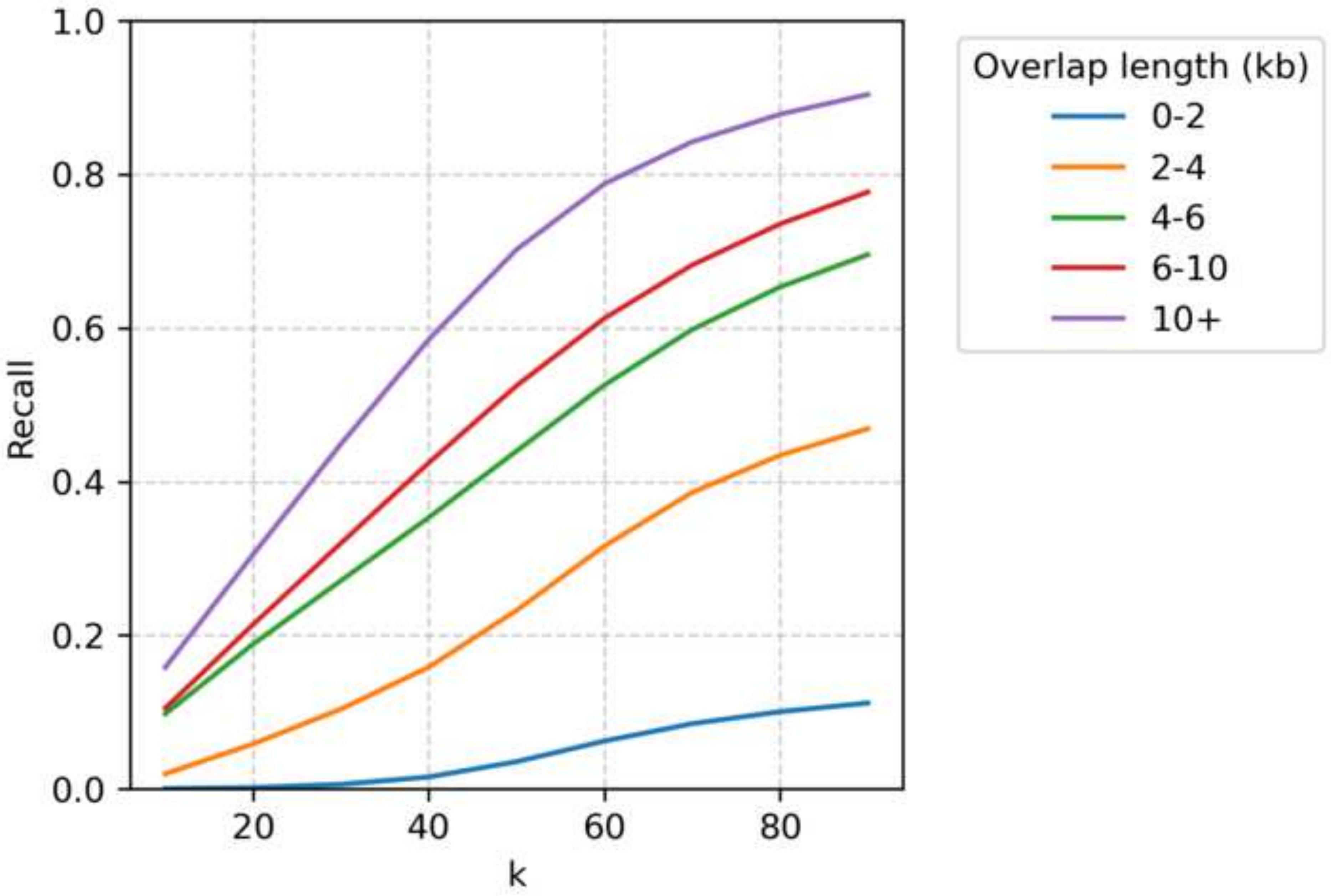

Personal Cover

Click here to access/download;Personal Cover;Fedrann Cover Letter(1).pdf

Dear Editor,

On behalf of my co-authors, I am pleased to submit the revised version of our manuscript, "**FEDRANN: effective long-read overlap detection based on dimensionality reduction and approximate nearest neighbors**". We would like to express our sincere gratitude to the reviewers for their insightful feedback, which has significantly strengthened the quality and practical utility of our work.

In response to the reviewers' primary concerns, we have implemented several major updates to the manuscript and the Fedrann framework:

- **Memory Optimization**: We have substantially refactored the Fedrann workflow from a monolithic architecture to an incremental, batch-based design. This, combined with the fusion of weighting and projection matrices, has reduced the peak memory requirement for human genome datasets by approximately 50%, bringing it within the capabilities of standard high-memory compute nodes. In addition, we demonstrate that by leveraging NVMe-based SSD swap space, Fedrann remains a viable tool for memory-constrained environments without compromising assembly accuracy
- **End-to-End Assembly Validation**: To address the gap between overlap detection and final assembly quality, we developed the **Fedrann-Shasta pipeline**. Our benchmarks on human datasets (CycloneSEQ and ONT) demonstrate that Fedrann generates high-quality assembly contigs with metrics comparable or superior to the native Shasta pipeline, particularly in resolving complex, repeat-rich regions such as the HLA locus.
- **Comprehensive Parameter Guidance**: We have added detailed "Application Notes" and a systematic calibration guide (Supplementary Note 1) to assist users in selecting optimal k-mer sizes and sampling fractions for various sequencing technologies and genomic complexities.

We have updated the Results, Discussion, and Methods sections and added several new supplementary figures and tables to provide full transparency regarding our design choices, benchmarking fairness, and precision-recall trade-offs.

We believe these revisions address all reviewer comments and further establish Fedrann as a

robust, innovative framework for the bioinformatics community. Thank you for your continued consideration of our manuscript.

Sincerely,

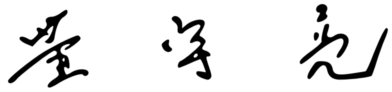

Yuliang Dong

More clear pdf version of "Response to reviewers comments" Click here to access/download;Personal Cover;Response to reviewers comments.pdf

FEDRANN: effective long-read overlap detection based on dimensionality reduction and approximate nearest neighbors

Response to reviewers' comments

Reviewer #1

Comment 1.1

Summary

This paper presents FEDRANN, a novel approach to overlap detection in long-read genome assembly that combines feature extraction, dimensionality reduction (DR), and approximate nearest neighbor (ANN) search. The authors systematically evaluate a range of design choices and implement the best-performing pipeline (IDF-SRP-NNDescent) as an open-source tool, Fedrann. Benchmarking against state-of-the-art tools (minimap2, MECAT2, wtdbg2, BLEND, xRead, MHAP) shows that Fedrann achieves competitive or superior accuracy and overlap graph quality across multiple sequencing platforms (ONT, PacBio HiFi, CycloneSEQ), while maintaining reasonable runtime. The conceptual framing of overlap detection as a k-NN search problem is both innovative and potentially influential.

Major Strengths

1. Novel conceptual framework: Framing overlap detection as a k-NN search problem,
2. drawing analogies from single-cell analysis, is creative and opens new algorithmic possibilities for genome assembly.
3. Systematic evaluation: The authors carefully assess multiple feature extraction, DR, and ANN methods before converging on the optimal pipeline.
4. Strong empirical results: Fedrann demonstrates high accuracy and graph quality, often outperforming established tools while remaining runtime-efficient.

We thank the reviewer for these positive comments.

Comment 1.2

Major Concerns and Recommendations

1. Memory consumption is a critical limitation

Fedrann requires >700 GB RAM for human genome datasets, which makes the tool impractical for most research labs and cost-prohibitive for cloud use.

While acknowledged, this limitation is somewhat downplayed. In its current state, Fedrann may be restricted to only very high-resource environments.

Recommendation: Either (a) demonstrate initial results of memory-reduction strategies (e.g., shared memory, memory-mapped structures, GPU acceleration), or (b) more prominently highlight this as a key limitation restricting practical adoption.

We thank the reviewer for raising this important concern regarding memory consumption and practical usability. We agree that memory usage is a critical factor for the adoption of Fedrann and that this limitation required both technical improvements and clearer discussion in the manuscript.

In response, we have substantially refactored the Fedrann workflow to reduce memory usage. The revised implementation replaces the original monolithic feature matrix construction with an incremental, batch-based design. Feature construction and dimensionality reduction are now performed on fixed-size batches of reads. A key optimization is the fusion of IDF weighting and Sparse Random Projection into a single matrix multiplication, based on our observation that both operations are linear. The resulting weighted projection matrix is constructed once and shared across worker processes, eliminating intermediate feature matrices and reducing redundant memory usage. These changes accelerated computation and reduced the peak memory requirement for the H1C human genome dataset from over 700 GB to approximately 357 GB (about a 50% reduction), bringing Fedrann within the capabilities of standard high-memory compute nodes. As detailed in our response to **Comment 2.2**, the utilization of system swap space further enables Fedrann to process human genome datasets on systems with less than 256 GB physical memory, albeit at the cost of increased computational time.

- We added **Supplementary Figure S7** and a new section in the **Results** titled "Efficient implementation of the IDF-SRP-NNDescent pipeline" to describe the algorithmic and architectural optimizations developed for Fedrann.

- We updated the "Fedrann enables high-precision, time-efficient overlap detection on large genomes" section in **Results** with the latest benchmarking results showing reduced time and memory usage of Fedrann.

- We rewrote the "Fedrann implementation" section in **Methods** to provide details on the updated Fedrann architecture and workflow.

- The **Discussion** section was revised to more clearly state the high memory requirement of the tool. We wrote: "Despite these strengths, the overall memory consumption of Fedrann remains higher than that of most existing overlap detection tools benchmarked, highlighting an important direction for future optimization through more memory-efficient data representations, shared-memory designs, or alternative implementations of ANN methods."

Comment 1.3

> 2. Incomplete evaluation at the assembly pipeline level
>
> The paper evaluates overlap graph quality but does not show results on final genome assemblies.
>
> This is a critical gap: improved overlaps must ultimately lead to better assemblies (NGA50, BUSCO scores, base accuracy). Without this, the utility of Fedrann remains uncertain.
>
> Recommendation: Include at least one end-to-end assembly experiment, comparing standard metrics with and without Fedrann overlaps. Even a single human or model genome dataset would provide crucial evidence.

We appreciate the reviewer's suggestion to evaluate Fedrann at the assembly pipeline level. We fully agree that the ultimate measure of an overlap detector's utility is its impact on the quality of the final genome assembly, including metrics such as N50, BUSCO scores, and base accuracy.

In response, we developed the Fedrann-Shasta pipeline by modifying the Shasta assembler to ingest Fedrann's output for overlap graph construction. We then performed *de novo* assembly of the human genome using datasets from CycloneSEQ (H4C) and

Oxford Nanopore (H4O) and benchmarked the results against the native Shasta pipeline. Our results demonstrate that Fedrann yields high-quality assembly contigs with metrics that are comparable (H4C) or significantly better (H4O) than those of the standard Shasta pipeline.

Nonetheless, we noted that integrating external overlap results into existing assembly frameworks presents inherent technical hurdles. As discussed in the manuscript, many current assemblers rely on opaque, in-memory data structures that tightly couple the discovery and assembly stages. Because this integration is currently constrained by the internal architecture of established tools, we consider these results a successful proof-of-concept rather than the upper limit of Fedrann's potential. We hope that Fedrann's transparent data structures will simplify such efforts for the community in the future. Furthermore, we are committed to developing a purpose-built assembler optimized specifically for Fedrann's output, which will be the focus of our next manuscript.

- We added **Supplementary Figures S11**, **S12** and **Supplementary Table S6** to describe the assembly results generated with the Fedrann-Shasta pipeline and compare them with the standard Shasta results. These results are summarized in a new "The Fedrann-Shasta pipeline generates high-quality human whole-genome assembly contigs" section in **Results**.
- We expanded the **Discussion** to address the technical hurdles of integrating Fedrann into existing assembly frameworks and to emphasize its utility as a modular, transparent building block for future assembly algorithms.
- We added two sections in the **Methods** describing the implementation of Fedrann-Shasta and the evaluation of *de novo* genomic assemblies, respectively.

Comment 1.4

> 3. Unclear treatment of k-mer size
>
> The manuscript does not specify the k-mer length (k) used for feature extraction, even though k-mer size critically determines sensitivity/specificity of overlaps:

Smaller k (e.g., 15): more sensitive but prone to spurious matches.

Larger k (e.g., 31): more specific but may miss overlaps in shorter or error-prone reads.

The authors mention filtering low-frequency k-mers and sampling 10–15% of the

alphabet, but do not justify frequency thresholds or sampling fractions.

Recommendation: Explicitly state the k-mer size used, justify parameter choices, and discuss their impact on overlap accuracy.

We fully agree with the reviewer that parameters such as *k*-mer size and sampling fractions are pivotal to the sensitivity and specificity of overlap detection. Fedrann utilizes a transparent parameter space where most variables exhibit predictable linear trade-offs: increasing parameter values generally enhances accuracy at the cost of increased memory consumption and execution time. We have updated the manuscript to provide comprehensive details and systematic guidance on parameter selection.

- **Supplementary Table S4** was been updated to include the specific *k*-mer sizes utilized for each dataset in this study.
- We added **Supplementary Figure S13** describing the impact of various parameters to Fedrann performance. These results were summarized in a new "Application notes" section in **Results**.
- We added **Supplementary Note 1** to provide detailed guidelines to assist users in selecting optimal parameters, including *k*-mer sizes and sampling fractions, based on their specific datasets.

Comment 1.5

Minor Comments

Figures S4–S6 (embedding dimension analysis) could be better explained in the main text with more intuitive interpretation.

We thank the reviewer for this insightful suggestion. We agree that providing a more intuitive explanation of the embedding dimension is crucial for understanding the algorithm's behavior.

We have significantly revised the relevant section to better articulate the trade-offs involved. Specifically, we now interpret the embedding dimension as a measure of "feature resolution"—where higher dimensions allow the system to better distinguish between similar but non-overlapping sequences. We have also introduced the concept of an "informational saturation point" to explain the diminishing returns observed in our sensitivity analysis, clarifying that 1,000 dimensions represent an optimal balance between structural fidelity and computational efficiency.

- We rewrote the last two paragraphs of "Sparse random projection enables scalable and accurate dimensionality reduction" in **Results** to provide intuitive interpretation on the experimental results.

> Benchmarking fairness: tools designed for high recall (e.g., minimap2, MECAT2) may be disadvantaged by post-processing into "top k" mode. Clarify this limitation in comparisons.

We appreciate the reviewer's insightful comment regarding benchmarking fairness. We acknowledge that tools like minimap2 and MECAT2 are inherently optimized for high recall by reporting all candidate overlaps above a certain quality threshold, whereas our approach (and other k-NN based methods) focuses on identifying the "top-k" most similar neighbors. However, we do not consider this a limitation of Fedrann or our benchmarking methodology. In overlap graph construction for *de novo* assembly, longer overlaps are generally more informative than shorter ones. Some assembly frameworks, such as Shasta, explicitly retain only the top k overlaps for each read during graph construction, while many other assemblers prune shorter overlaps as transitive edges during simplification. This biological context justifies a focus on identifying the most significant overlaps rather than all potential matches. In the revised manuscript, we have included additional results and discussion to examine these design differences in detail.

- To ensure a more balanced view, we added **Supplementary Figure S10**, which demonstrates the changes in precision and recall for each benchmarked tool with respect to the value of k. This analysis provides transparency regarding the inherent design of Fedrann, illustrating that the tool is optimized for high-precision overlap

detection and is unlikely to achieve exhaustive recall without a substantial sacrifice in precision.

- We added a new paragraph in the "Fedrann enables high-precision, time-efficient overlap detection on large genomes" section in **Results** to explicitly contrast threshold-based and k-NN based overlap detection and highlight the impact of different design choices on benchmarking results.

Comment 1.6

> Minor Corrections
>
> Figure 1: Step numbering is currently (1), (3), (4). Step (2) is missing

We sincerely thank the reviewer for their meticulous attention to detail. We have corrected the step numbering of Figure 1 in the updated manuscript.

- The caption of **Figure 1** was updated to fix this issue.

Comment 1.7

> Overall Recommendation
>
> This paper presents a novel and promising framework for overlap detection with strong methodological rigor and empirical results. However, two major gaps — excessive memory usage and lack of assembly-level validation — must be addressed before the work can be considered fully convincing. In addition, clarity on basic parameters such as k-mer size is necessary for robustness.

We thank the reviewer for their thoughtful evaluation. We have addressed the concerns regarding memory consumption and assembly-level validation by implementing batch-processing optimizations and developing the Fedrann-Shasta pipeline for end-to-end benchmarking. We hope these revisions and the detailed responses provided above fully address your concerns and demonstrate the robustness of the Fedrann framework.

Reviewer #2

Comment 2.1

> The authors present FEDRANN, a novel overlap detection framework that integrates feature extraction, sparse random projection-based dimensionality reduction, and approximate nearest-neighbor searching via NNDescent. The manuscript is clearly written and supported by extensive benchmarking across multiple platforms (ONT, HiFi, CycloneSEQ) and genome scales. The work introduces a notably different paradigm from traditional seed-and-extend and MinHash/LSH-based methods, and the demonstrated improvements in repeat-rich genomic regions highlight the potential value of this approach for OLC-based assembly pipelines.

We thank the reviewer for these positive comments.

Comment 2.2

> Below, I outline several concerns and suggestions for improvement:
>
> FEDRANN requires more than 700 GB of RAM to process human datasets, which far exceeds the resource demands of the comparison tools. In practice, assembly groups typically operate under <256 GB per compute node, meaning the current implementation may be unusable for many users. Improving memory efficiency is a critical issue that must be addressed.

We thank the reviewer for this pragmatic observation regarding memory constraints. We have taken this feedback seriously and implemented both algorithmic and deployment-level solutions to ensure Fedrann is accessible to a broader range of users.

Algorithmic optimization: As detailed in our response to **Comment 1.2**, we have refactored the memory architecture, transitioning from a monolithic matrix construction to an incremental batch-processing workflow. By integrating shared-memory structures for the projection matrix, we have successfully halved the peak physical memory requirement for human genome H4C datasets from >700 GB to approximately 357 GB.

Support for 256 GB memory environments: Recognizing that 256 GB RAM is a common standard for bioinformatics compute nodes, we have validated a deployment

strategy to accommodate these systems. Users can now process human whole-genome sequencing datasets on 256 GB machines by leveraging system swap space. Our benchmark tests confirm that while this mode incurs a performance penalty in terms of execution time, it does not impact the accuracy of the overlap detection or final assembly. Furthermore, we demonstrated that utilizing NVMe-based Solid State Drives (SSDs) for the swap partition significantly mitigates the latency typically associated with disk-swapping; for instance, a 32-thread SSD configuration outperformed a 64-thread HDD execution. This established workflow makes Fedrann a viable tool for memory-constrained environments without necessitating hardware upgrades.

- We have significantly refactored the Fedrann workflow to optimize execution time and memory efficiency. The corresponding **Results**, **Discussion**, and **Methods** sections have been updated to reflect these improvements, as detailed in our response to **Comment 1.2**.
- We added **Supplementary Table S7**, which provides benchmark results for Fedrann under various memory constraints. These tests confirm that Fedrann can successfully process human whole-genome datasets on 256 GB machines. These findings are now described in a new "Application notes" section within the **Results**.
- We have added **Supplementary Note 2**, which provides a comprehensive technical guide for users on how to configure system swap space to run Fedrann in memory-limited environments.

Comment 2.3

> The method involves multiple parameters that currently lack practical tuning guidance (e.g., read accuracy, kmer alphabet size, coverage depth, species complexity). The authors should provide a calibration guide with representative defaults for ONT/HiFi/CycloneSEQ and for genomes of different sizes, as well as runtime-accuracy trade-off curves for the SRP embedding dimension and ANN search settings.

We thank the reviewer for this constructive suggestion regarding the usability and transparency of our parameter settings. To better support the community in deploying our tool across various research scenarios, we have enhanced the manuscript by

providing a systematic calibration guide and empirical performance analysis. These additions offer data-driven guidance on balancing predictive accuracy with computational efficiency for different sequencing technologies and genome sizes.

- We added **Supplementary Note 1** to include a comprehensive parameter tuning guide. This guide provides recommendations for k-mer size, sampling fractions, and embedding dimensions, tailored specifically to different sequencing platforms (ONT, PacBio HiFi, and CycloneSEQ) and genomic complexities ranging from bacteria to humans.
- **Supplementary Table S4** has been updated to list the exact Fedrann parameter configurations used for every dataset in this study. These settings were empirically determined to achieve an optimal balance between precision and execution speed.
- We have added runtime-accuracy trade-off curves for the Sparse Random Projection (SRP) embedding dimension and ANN search settings in **Supplementary Figure S13**. These results are now analyzed in the "Application notes" section of **Results**, providing users with a quantitative understanding of how parameter adjustments influence computational cost and sensitivity.

Comment 2.4

FEDRANN retrieves only the top-k neighbors per read, whereas most baseline tools attempt full overlap discovery. It remains unclear whether improved precision comes at the cost of substantial recall losses. Evaluation should include overlap recall plots and precision-recall analysis stratified by overlap length.

We sincerely appreciate the reviewer's constructive suggestion regarding the evaluation of recall and precision. We agree that a comprehensive understanding of the trade-off between the "top-k" retrieval strategy and exhaustive recall is essential for validating Fedrann’s effectiveness.

In response to your guidance, we performed two new sets of experiments using the H3C dataset. First, we conducted a precision-recall analysis across a range of k values to observe how reporting depth influences performance. We found that while threshold-based tools prioritize exhaustive discovery, Fedrann’s k-NN approach is optimized for high-precision retrieval of the most significant overlaps. While Fedrann can

achieve recall comparable to exhaustive tools like minimap2 by increasing k, this results in considerable losses in precision because Fedrann is designed to report exactly k candidates, even for reads with fewer biological overlaps. Second, we stratified recall by overlap length, which confirmed that Fedrann successfully prioritizes longer, more informative overlaps. These are biologically critical for *de novo* assembly, as shorter overlaps are frequently pruned as transitive edges during graph simplification. We have updated the manuscript and supplementary materials to reflect these findings and provide transparency regarding the conditions under which different tools excel.

- We added Sup**plementary Figure S9** to illustrates the dynamic changes in recall and precision for Fedrann and existing tools as k increases.
- We added **Supplementary Figure S10**, presenting recall rates stratified by overlap length thresholds.
- We added a dedicated paragraph in the "Fedrann enables high-precision, time-efficient overlap detection on large genomes" section in **Results** to contrast threshold-based and k-NN based detection. This section now explicitly describes how recall and precision respond to changes in k, providing users with clearer expectations.

Comment 2.5

> Overlap precision and graph connectivity are useful proxies, but they do not guarantee better genome reconstruction. The evaluation should integrate FEDRANN into at least one complete OLC assembly workflow (e.g., WTDBG2, Canu, Shasta) and report standard assembly metrics (N50/NG50, BUSCO, QV, and structural variant detection). Assembly contiguity in complex immune loci (e.g., HLA, IGK) would further support claims of improved handling of repeats.

We appreciate the reviewer's suggestion to evaluate Fedrann within a complete assembly workflow and specifically to investigate complex immune loci. We agree that metrics like overlap precision are most valuable when they translate into improved genomic reconstruction.

In response, we developed the Fedrann-Shasta pipeline by modifying the Shasta

assembler to ingest Fedrann's output for overlap graph construction. We performed *de novo* assembly of the human genome using CycloneSEQ (H4C) and Oxford Nanopore (H4O) datasets and benchmarked the results against the native Shasta pipeline. Our results demonstrate that Fedrann yields high-quality assembly contigs with metrics (N50, BUSCO, and QV) that are comparable to (H4C) or significantly better than (H4O) the standard Shasta pipeline.

Furthermore, we specifically analyzed the HLA region to evaluate Fedrann's performance in repeat-rich regions. While both pipelines partitioned the HLA region into two contigs, the native Shasta assembly exhibited a collapse of a ~40 kb repeat unit, incorrectly merging two copies. This misassembly was correctly resolved in the Fedrann-Shasta assembly. This suggests that Fedrann's high-accuracy overlap detection provides the specificity required to resolve complex genomic contexts that may be prone to collapse in traditional heuristic-based pipelines.

- We added **Supplementary Figures S11**, **S12** and **Supplementary Table S6** to describe the assembly results generated with the Fedrann-Shasta pipeline and compare them with the standard Shasta results. These results are summarized in a new "The Fedrann-Shasta pipeline generates high-quality human whole-genome assembly contigs" section in **Results**.
- We expanded the **Discussion** to address the technical hurdles of integrating Fedrann into existing assembly frameworks and to emphasize its utility as a modular, transparent building block for future assembly algorithms.
- We added two sections in **Methods** describing the implementation of Fedrann-Shasta and *de novo* assembly evaluation, respectively.
