# Supplementary material for: FEDRANN: effective long-read overlap detection based on dimensionality reduction and approximate nearest neighbors
Source: Gigascience. 2026 May 8;15:giag048. doi: 10.1093/gigascience/giag048 (PMC13201080; doi:10.1093/gigascience/giag048)
Supplement: giag048_Authors_Response_To_Reviewer_Comments_original_submission [file giag048_authors_response_to_reviewer_comments_original_submission.pdf]

Author's Response To Reviewer Comments

Please note that a professionally formatted version of our response has been uploaded as a separate PDF file titled "Response to reviewers' comments". > We kindly suggest referring to that PDF for a more convenient reading experience.

FEDRANN: effective long-read overlap detection based on dimensionality reduction and approximate nearest neighbors
Response to reviewers’ comments

Reviewer #1

Comment 1.1

Summary
This paper presents FEDRANN, a novel approach to overlap detection in long-read genome assembly that combines feature extraction, dimensionality reduction (DR), and approximate nearest neighbor (ANN) search. The authors systematically evaluate a range of design choices and implement the best-performing pipeline (IDF-SRP-NNDescent) as an open-source tool, Fedrann. Benchmarking against state-of-the-art tools (minimap2, MECAT2, wtdbg2, BLEND, xRead, MHAP) shows that Fedrann achieves competitive or superior accuracy and overlap graph quality across multiple sequencing platforms (ONT, PacBio HiFi, CycloneSEQ), while maintaining reasonable runtime. The conceptual framing of overlap detection as a k-NN search problem is both innovative and potentially influential.
Major Strengths
1.Novel conceptual framework: Framing overlap detection as a k-NN search problem,
2.drawing analogies from single-cell analysis, is creative and opens new algorithmic possibilities for genome assembly.
3.Systematic evaluation: The authors carefully assess multiple feature extraction, DR, and ANN methods before converging on the optimal pipeline.
4.Strong empirical results: Fedrann demonstrates high accuracy and graph quality, often outperforming established tools while remaining runtime-efficient.

We thank the reviewer for these positive comments.

Comment 1.2

Major Concerns and Recommendations
1. Memory consumption is a critical limitation
Fedrann requires >700 GB RAM for human genome datasets, which makes the tool impractical for most research labs and cost-prohibitive for cloud use.
While acknowledged, this limitation is somewhat downplayed. In its current state, Fedrann may be restricted to only very high-resource environments.
Recommendation: Either (a) demonstrate initial results of memory-reduction strategies (e.g., shared memory, memory-mapped structures, GPU acceleration), or (b) more prominently highlight this as a key limitation restricting practical adoption.

We thank the reviewer for raising this important concern regarding memory consumption and practical usability. We agree that memory usage is a critical factor for the adoption of Fedrann and that this limitation required both technical improvements and clearer discussion in the manuscript.
In response, we have substantially refactored the Fedrann workflow to reduce memory usage. The revised implementation replaces the original monolithic feature matrix construction with an incremental, batch-based design. Feature construction and dimensionality reduction are now performed on fixed-size batches of reads. A key optimization is the fusion of IDF weighting and Sparse Random Projection into a single

matrix multiplication, based on our observation that both operations are linear. The resulting weighted projection matrix is constructed once and shared across worker processes, eliminating intermediate feature matrices and reducing redundant memory usage. These changes accelerated computation and reduced the peak memory requirement for the H1C human genome dataset from over 700 GB to approximately 357 GB (about a 50% reduction), bringing Fedrann within the capabilities of standard high-memory compute nodes. As detailed in our response to Comment 2.2, the utilization of system swap space further enables Fedrann to process human genome datasets on systems with less than 256 GB physical memory, albeit at the cost of increased computational time.

λWe added Supplementary Figure S7 and a new section in the Results titled "Efficient implementation of the IDF-SRP-NNDescent pipeline" to describe the algorithmic and architectural optimizations developed for Fedrann.

λWe updated the "Fedrann enables high-precision, time-efficient overlap detection on large genomes" section in Results with the latest benchmarking results showing reduced time and memory usage of Fedrann.

λWe rewrote the "Fedrann implementation" section in Methods to provide details on the updated Fedrann architecture and workflow.

λThe Discussion section was revised to more clearly state the high memory requirement of the tool. We wrote: "Despite these strengths, the overall memory consumption of Fedrann remains higher than that of most existing overlap detection tools benchmarked, highlighting an important direction for future optimization through more memory-efficient data representations, shared-memory designs, or alternative implementations of ANN methods."

Comment 1.3

2. Incomplete evaluation at the assembly pipeline level

The paper evaluates overlap graph quality but does not show results on final genome assemblies.

This is a critical gap: improved overlaps must ultimately lead to better assemblies (NGA50, BUSCO scores, base accuracy). Without this, the utility of Fedrann remains uncertain.

Recommendation: Include at least one end-to-end assembly experiment, comparing standard metrics with and without Fedrann overlaps. Even a single human or model genome dataset would provide crucial evidence.

We appreciate the reviewer's suggestion to evaluate Fedrann at the assembly pipeline level. We fully agree that the ultimate measure of an overlap detector's utility is its impact on the quality of the final genome assembly, including metrics such as N50, BUSCO scores, and base accuracy.

In response, we developed the Fedrann-Shasta pipeline by modifying the Shasta assembler to ingest Fedrann's output for overlap graph construction. We then performed de novo assembly of the human genome using datasets from CycloneSEQ (H4C) and Oxford Nanopore (H4O) and benchmarked the results against the native Shasta pipeline. Our results demonstrate that Fedrann yields high-quality assembly contigs with metrics that are comparable (H4C) or significantly better (H4O) than those of the standard Shasta pipeline.

Nonetheless, we noted that integrating external overlap results into existing assembly frameworks presents inherent technical hurdles. As discussed in the manuscript, many current assemblers rely on opaque, in-memory data structures that tightly couple the discovery and assembly stages. Because this integration is currently constrained by the internal architecture of established tools, we consider these results a successful proof-of-concept rather than the upper limit of Fedrann's potential. We hope that Fedrann's transparent data structures will simplify such efforts for the community in the future. Furthermore, we are committed to developing a purpose-built assembler optimized specifically for Fedrann's output, which will be the focus of our next manuscript.

λWe added Supplementary Figures S11, S12 and Supplementary Table S6 to describe the assembly results generated with the Fedrann-Shasta pipeline and compare them with the standard Shasta results. These results are summarized in a new "The Fedrann-Shasta pipeline generates high-quality human whole-genome assembly contigs" section in Results.

λWe expanded the Discussion to address the technical hurdles of integrating Fedrann into existing assembly frameworks and to emphasize its utility as a modular, transparent building block for future assembly algorithms.

λWe added two sections in the Methods describing the implementation of Fedrann-Shasta and the evaluation of de novo genomic assemblies, respectively.

Comment 1.4

3. Unclear treatment of k-mer size
The manuscript does not specify the k-mer length (k) used for feature extraction, even though k-mer size critically determines sensitivity/specificity of overlaps:
Smaller k (e.g., 15): more sensitive but prone to spurious matches.
Larger k (e.g., 31): more specific but may miss overlaps in shorter or error-prone reads.
The authors mention filtering low-frequency k-mers and sampling 10–15% of the alphabet, but do not justify frequency thresholds or sampling fractions.
Recommendation: Explicitly state the k-mer size used, justify parameter choices, and discuss their impact on overlap accuracy.

We fully agree with the reviewer that parameters such as k-mer size and sampling fractions are pivotal to the sensitivity and specificity of overlap detection. Fedrann utilizes a transparent parameter space where most variables exhibit predictable linear trade-offs: increasing parameter values generally enhances accuracy at the cost of increased memory consumption and execution time. We have updated the manuscript to provide comprehensive details and systematic guidance on parameter selection.
λSupplementary Table S4 was been updated to include the specific k-mer sizes utilized for each dataset in this study.
λWe added Supplementary Figure S13 describing the impact of various parameters to Fedrann performance. These results were summarized in a new "Application notes" section in Results.
λWe added Supplementary Note 1 to provide detailed guidelines to assist users in selecting optimal parameters, including k-mer sizes and sampling fractions, based on their specific datasets.

Comment 1.5

Minor Comments
Figures S4–S6 (embedding dimension analysis) could be better explained in the main text with more intuitive interpretation.

We thank the reviewer for this insightful suggestion. We agree that providing a more intuitive explanation of the embedding dimension is crucial for understanding the algorithm's behavior.
We have significantly revised the relevant section to better articulate the trade-offs involved. Specifically, we now interpret the embedding dimension as a measure of "feature resolution"—where higher dimensions allow the system to better distinguish between similar but non-overlapping sequences. We have also introduced the concept of an "informational saturation point" to explain the diminishing returns observed in our sensitivity analysis, clarifying that 1,000 dimensions represent an optimal balance between structural fidelity and computational efficiency.
λWe rewrote the last two paragraphs of “Sparse random projection enables scalable and accurate dimensionality reduction” in Results to provide intuitive interpretation on the experimental results.

Benchmarking fairness: tools designed for high recall (e.g., minimap2, MECAT2) may be disadvantaged by post-processing into “top k” mode. Clarify this limitation in comparisons.

We appreciate the reviewer's insightful comment regarding benchmarking fairness. We acknowledge that tools like minimap2 and MECAT2 are inherently optimized for high recall by reporting all candidate overlaps above a certain quality threshold, whereas our approach (and other k-NN based methods) focuses on identifying the "top-k" most similar neighbors. However, we do not consider this a limitation of Fedrann or our benchmarking methodology. In overlap graph construction for de novo assembly, longer overlaps are generally more informative than shorter ones. Some assembly frameworks, such as Shasta, explicitly retain only the top k overlaps for each read during graph construction, while many other assemblers prune

shorter overlaps as transitive edges during simplification. This biological context justifies a focus on identifying the most significant overlaps rather than all potential matches. In the revised manuscript, we have included additional results and discussion to examine these design differences in detail.
λTo ensure a more balanced view, we added Supplementary Figure S10, which demonstrates the changes in precision and recall for each benchmarked tool with respect to the value of k. This analysis provides transparency regarding the inherent design of Fedrann, illustrating that the tool is optimized for high-precision overlap detection and is unlikely to achieve exhaustive recall without a substantial sacrifice in precision.
λWe added a new paragraph in the "Fedrann enables high-precision, time-efficient overlap detection on large genomes" section in Results to explicitly contrast threshold-based and k-NN based overlap detection and highlight the impact of different design choices on benchmarking results.

Comment 1.6

Minor Corrections
Figure 1: Step numbering is currently (1), (3), (4). Step (2) is missing

We sincerely thank the reviewer for their meticulous attention to detail. We have corrected the step numbering of Figure 1 in the updated manuscript.
λThe caption of Figure 1 was updated to fix this issue.

Comment 1.7

Overall Recommendation
This paper presents a novel and promising framework for overlap detection with strong methodological rigor and empirical results. However, two major gaps — excessive memory usage and lack of assembly-level validation — must be addressed before the work can be considered fully convincing. In addition, clarity on basic parameters such as k-mer size is necessary for robustness.

We thank the reviewer for their thoughtful evaluation. We have addressed the concerns regarding memory consumption and assembly-level validation by implementing batch-processing optimizations and developing the Fedrann-Shasta pipeline for end-to-end benchmarking. We hope these revisions and the detailed responses provided above fully address your concerns and demonstrate the robustness of the Fedrann framework.

Reviewer #2

Comment 2.1

The authors present FEDRANN, a novel overlap detection framework that integrates feature extraction, sparse random projection-based dimensionality reduction, and approximate nearest-neighbor searching via NNDescent. The manuscript is clearly written and supported by extensive benchmarking across multiple platforms (ONT, HiFi, CycloneSEQ) and genome scales. The work introduces a notably different paradigm from traditional seed-and-extend and MinHash/LSH-based methods, and the demonstrated improvements in repeat-rich genomic regions highlight the potential value of this approach for OLC-based assembly pipelines.

We thank the reviewer for these positive comments.

Comment 2.2

Below, I outline several concerns and suggestions for improvement:

FEDRANN requires more than 700 GB of RAM to process human datasets, which far exceeds the resource demands of the comparison tools. In practice, assembly groups typically operate under <256 GB per compute node, meaning the current implementation may be unusable for many users. Improving memory efficiency is a critical issue that must be addressed.

We thank the reviewer for this pragmatic observation regarding memory constraints. We have taken this feedback seriously and implemented both algorithmic and deployment-level solutions to ensure Fedrann is accessible to a broader range of users.
Algorithmic optimization: As detailed in our response to Comment 1.2, we have refactored the memory architecture, transitioning from a monolithic matrix construction to an incremental batch-processing workflow. By integrating shared-memory structures for the projection matrix, we have successfully halved the peak physical memory requirement for human genome H4C datasets from >700 GB to approximately 357 GB.
Support for 256 GB memory environments: Recognizing that 256 GB RAM is a common standard for bioinformatics compute nodes, we have validated a deployment strategy to accommodate these systems. Users can now process human whole-genome sequencing datasets on 256 GB machines by leveraging system swap space. Our benchmark tests confirm that while this mode incurs a performance penalty in terms of execution time, it does not impact the accuracy of the overlap detection or final assembly. Furthermore, we demonstrated that utilizing NVMe-based Solid State Drives (SSDs) for the swap partition significantly mitigates the latency typically associated with disk-swapping; for instance, a 32-thread SSD configuration outperformed a 64-thread HDD execution. This established workflow makes Fedrann a viable tool for memory-constrained environments without necessitating hardware upgrades.
λWe have significantly refactored the Fedrann workflow to optimize execution time and memory efficiency. The corresponding Results, Discussion, and Methods sections have been updated to reflect these improvements, as detailed in our response to Comment 1.2.
λWe added Supplementary Table S7, which provides benchmark results for Fedrann under various memory constraints. These tests confirm that Fedrann can successfully process human whole-genome datasets on 256 GB machines. These findings are now described in a new "Application notes" section within the Results.
λWe have added Supplementary Note 2, which provides a comprehensive technical guide for users on how to configure system swap space to run Fedrann in memory-limited environments.

Comment 2.3

The method involves multiple parameters that currently lack practical tuning guidance (e.g., read accuracy, kmer alphabet size, coverage depth, species complexity). The authors should provide a calibration guide with representative defaults for ONT/HiFi/CycloneSEQ and for genomes of different sizes, as well as runtime-accuracy trade-off curves for the SRP embedding dimension and ANN search settings.

We thank the reviewer for this constructive suggestion regarding the usability and transparency of our parameter settings. To better support the community in deploying our tool across various research scenarios, we have enhanced the manuscript by providing a systematic calibration guide and empirical performance analysis. These additions offer data-driven guidance on balancing predictive accuracy with computational efficiency for different sequencing technologies and genome sizes.
λWe added Supplementary Note 1 to include a comprehensive parameter tuning guide. This guide provides recommendations for k-mer size, sampling fractions, and embedding dimensions, tailored specifically to different sequencing platforms (ONT, PacBio HiFi, and CycloneSEQ) and genomic complexities ranging from bacteria to humans.
λSupplementary Table S4 has been updated to list the exact Fedrann parameter configurations used for every dataset in this study. These settings were empirically determined to achieve an optimal balance between precision and execution speed.
λWe have added runtime-accuracy trade-off curves for the Sparse Random Projection (SRP) embedding dimension and ANN search settings in Supplementary Figure S13. These results are now analyzed in the "Application notes" section of Results, providing users with a quantitative understanding of how parameter adjustments influence computational cost and sensitivity.

Comment 2.4

FEDRANN retrieves only the top-k neighbors per read, whereas most baseline tools attempt full overlap discovery. It remains unclear whether improved precision comes at the cost of substantial recall losses. Evaluation should include overlap recall plots and precision-recall analysis stratified by overlap length.

We sincerely appreciate the reviewer's constructive suggestion regarding the evaluation of recall and precision. We agree that a comprehensive understanding of the trade-off between the "top-k" retrieval strategy and exhaustive recall is essential for validating Fedrann's effectiveness.
In response to your guidance, we performed two new sets of experiments using the H3C dataset. First, we conducted a precision-recall analysis across a range of k values to observe how reporting depth influences performance. We found that while threshold-based tools prioritize exhaustive discovery, Fedrann's k-NN approach is optimized for high-precision retrieval of the most significant overlaps. While Fedrann can achieve recall comparable to exhaustive tools like minimap2 by increasing k, this results in considerable losses in precision because Fedrann is designed to report exactly k candidates, even for reads with fewer biological overlaps. Second, we stratified recall by overlap length, which confirmed that Fedrann successfully prioritizes longer, more informative overlaps. These are biologically critical for de novo assembly, as shorter overlaps are frequently pruned as transitive edges during graph simplification. We have updated the manuscript and supplementary materials to reflect these findings and provide transparency regarding the conditions under which different tools excel.
λWe added Supplementary Figure S9 to illustrates the dynamic changes in recall and precision for Fedrann and existing tools as k increases.
λWe added Supplementary Figure S10, presenting recall rates stratified by overlap length thresholds.
λWe added a dedicated paragraph in the "Fedrann enables high-precision, time-efficient overlap detection on large genomes" section in Results to contrast threshold-based and k-NN based detection. This section now explicitly describes how recall and precision respond to changes in k, providing users with clearer expectations.

Comment 2.5

Overlap precision and graph connectivity are useful proxies, but they do not guarantee better genome reconstruction. The evaluation should integrate FEDRANN into at least one complete OLC assembly workflow (e.g., WTDBG2, Canu, Shasta) and report standard assembly metrics (N50/NG50, BUSCO, QV, and structural variant detection). Assembly contiguity in complex immune loci (e.g., HLA, IGK) would further support claims of improved handling of repeats.

We appreciate the reviewer's suggestion to evaluate Fedrann within a complete assembly workflow and specifically to investigate complex immune loci. We agree that metrics like overlap precision are most valuable when they translate into improved genomic reconstruction.
In response, we developed the Fedrann-Shasta pipeline by modifying the Shasta assembler to ingest Fedrann's output for overlap graph construction. We performed de novo assembly of the human genome using CycloneSEQ (H4C) and Oxford Nanopore (H4O) datasets and benchmarked the results against the native Shasta pipeline. Our results demonstrate that Fedrann yields high-quality assembly contigs with metrics (N50, BUSCO, and QV) that are comparable to (H4C) or significantly better than (H4O) the standard Shasta pipeline.
Furthermore, we specifically analyzed the HLA region to evaluate Fedrann's performance in repeat-rich regions. While both pipelines partitioned the HLA region into two contigs, the native Shasta assembly exhibited a collapse of a ~40 kb repeat unit, incorrectly merging two copies. This misassembly was correctly resolved in the Fedrann-Shasta assembly. This suggests that Fedrann's high-accuracy overlap detection provides the specificity required to resolve complex genomic contexts that may be prone to collapse in traditional heuristic-based pipelines.
λWe added Supplementary Figures S11, S12 and Supplementary Table S6 to describe the assembly results generated with the Fedrann-Shasta pipeline and compare them with the standard Shasta results. These results are summarized in a new "The Fedrann-Shasta pipeline generates high-quality human whole-genome assembly contigs" section in Results.
λWe expanded the Discussion to address the technical hurdles of integrating Fedrann into existing

assembly frameworks and to emphasize its utility as a modular, transparent building block for future assembly algorithms.
λWe added two sections in Methods describing the implementation of Fedrann-Shasta and de novo assembly evaluation, respectively.

Cookie Preference Center

We use cookies which are necessary to make our site work. We may also use additional cookies to analyze, improve and personalize our content and your digital experience. For more information, see our Cookie Policy.

You may choose not to allow some types of cookies. However, blocking some types may impact your experience of our site and the services we are able to offer. See the different category headings below to find out more or change your settings.

Allow all

Manage Consent Preferences

Strictly Necessary Cookies

Always active

These cookies are necessary for the website to function and cannot be switched off in our systems. They are usually only set in response to actions made by you which amount to a request for services, such as setting your privacy preferences, logging in or filling in forms. You can set your browser to block or alert you about these cookies, but some parts of the site will not then work.

Functional Cookies

These cookies enable the website to provide enhanced functionality and personalisation. They may be set by us or by third party providers whose services we have added to our pages. If you do not allow these cookies then some or all of these services may not function properly.

Performance Cookies

These cookies allow us to count visits and traffic sources so we can measure and improve the performance of our site. They help us to know which pages are the most and least popular and see how visitors move around the site.

Targeting Cookies

These cookies may be set through our site by our advertising partners. They may be used by those companies to build a profile of your interests and show you relevant adverts on other sites. If you do not allow these cookies, you will experience less targeted advertising.

Cookie List

Clear

Apply Cancel

Consent Leg.Interest

Confirm my choices

Powe
