# Supplementary material for: FEDRANN: effective long-read overlap detection based on dimensionality reduction and approximate nearest neighbors
Source: Gigascience. 2026 May 8;15:giag048. doi: 10.1093/gigascience/giag048 (PMC13201080; doi:10.1093/gigascience/giag048)
Supplement: giag048_Reviewer_2_Report_original_submission — Reviewer 2 -- 11/24/2025 [file giag048_reviewer_2_report_original_submission.pdf]

Reviewer Report

Title: FEDRANN: effective long-read overlap detection based on dimensionality reduction and approximate nearest neighbors

Version: Original Submission **Date:** 11/24/2025

Reviewer name: Reviewer 2

Reviewer Comments to Author:

The authors present FEDRANN, a novel overlap detection framework that integrates feature extraction, sparse random projection-based dimensionality reduction, and approximate nearest-neighbor searching via NNDescent. The manuscript is clearly written and supported by extensive benchmarking across multiple platforms (ONT, HiFi, CycloneSEQ) and genome scales. The work introduces a notably different paradigm from traditional seed-and-extend and MinHash/LSH-based methods, and the demonstrated improvements in repeat-rich genomic regions highlight the potential value of this approach for OLC-based assembly pipelines. Below, I outline several concerns and suggestions for improvement:(1) FEDRANN requires more than 700 GB of RAM to process human datasets, which far exceeds the resource demands of the comparison tools. In practice, assembly groups typically operate under <256 GB per compute node, meaning the current implementation may be unusable for many users. Improving memory efficiency is a critical issue that must be addressed.(2) The method involves multiple parameters that currently lack practical tuning guidance (e.g., read accuracy, k-mer alphabet size, coverage depth, species complexity). The authors should provide a calibration guide with representative defaults for ONT/HiFi/CycloneSEQ and for genomes of different sizes, as well as runtime-accuracy trade-off curves for the SRP embedding dimension and ANN search settings.(3) FEDRANN retrieves only the top-k neighbors per read, whereas most baseline tools attempt full overlap discovery. It remains unclear whether improved precision comes at the cost of substantial recall losses. Evaluation should include overlap recall plots and precision-recall analysis stratified by overlap length.(4) Overlap precision and graph connectivity are useful proxies, but they do not guarantee better genome reconstruction. The evaluation should integrate FEDRANN into at least one complete OLC assembly workflow (e.g., WTDBG2, Canu, Shasta) and report standard assembly metrics (N50/NG50, BUSCO, QV, and structural variant detection). Assembly contiguity in complex immune loci (e.g., HLA, IGK) would further support claims of improved handling of repeats.

Level of Interest

Please indicate how interesting you found the manuscript: Choose an item.

Quality of Written English

Please indicate the quality of language in the manuscript: Choose an item.

Declaration of Competing Interests

Please complete a declaration of competing interests, considering the following questions:

- Have you in the past five years received reimbursements, fees, funding, or salary from an organisation that may in any way gain or lose financially from the publication of this manuscript, either now or in the future?
- Do you hold any stocks or shares in an organisation that may in any way gain or lose financially from the publication of this manuscript, either now or in the future?
- Do you hold or are you currently applying for any patents relating to the content of the manuscript?
- Have you received reimbursements, fees, funding, or salary from an organization that holds or has applied for patents relating to the content of the manuscript?
- Do you have any other financial competing interests?
- Do you have any non-financial competing interests in relation to this paper?

If you can answer no to all of the above, write 'I declare that I have no competing interests' below. If your reply is yes to any, please give details below.

I declare that I have no competing interests.

I agree to the open peer review policy of the journal. I understand that my name will be included on my report to the authors and, if the manuscript is accepted for publication, my named report including any attachments I upload will be posted on the website along with the authors' responses. I agree for my report to be made available under an Open Access Creative Commons CC-BY license (http://creativecommons.org/licenses/by/4.0/). I understand that any comments which I do not wish to be included in my named report can be included as confidential comments to the editors, which will not be published.

Choose an item.

To further support our reviewers, we have joined with Publons, where you can gain additional credit to further highlight your hard work (see: https://publons.com/journal/530/gigascience). On publication of this paper, your review will be automatically added to Publons, you can then choose whether or not to claim your Publons credit. I understand this statement.

Yes Choose an item.
